# ARID5A RNA-binding coordinates microglial defense and ferroptosis in iPSC-derived models

**DOI:** 10.1038/s41467-026-76131-0

**Published:** 2026-08-12

**Authors:** Danielle M. Schafer, Steven M. Blue, Shuhao Xu, Orel Mizrahi, Maya L. Gosztyla, Lena Street, Katherine L. Rothamel, Hema Kopalle, Samuel B. Landry, Elijah Khalil F. Rosales, Rebecca H. Tien, Sara Elmsaouri, Nicole G. Coufal, Marko Jovanovic, Gene W. Yeo

**Affiliations:** 1 Department of Cellular and Molecular Medicine, University of California San Diego, La Jolla, CA USA; 2 Sanford Consortium for Regenerative Medicine, University of California San Diego, La Jolla, CA USA; 3 Institute for Genomic Medicine, University of California San Diego, La Jolla, CA USA; 4 Department of Biological Sciences, Columbia University, New York, NY USA; 5 Center for RNA Technologies and Therapeutics, University of California San Diego, La Jolla, CA USA; 6 Department of Pediatrics, University of California San Diego, La Jolla, CA USA; 7 Salk Institute for Biological Studies, La Jolla, CA USA; 8 Sanford Laboratories for Innovative Medicines, La Jolla, CA USA; 9 Sanford Stem Cell Institute, University of California San Diego, La Jolla, CA USA

**Keywords:** Microglia, RNA splicing, Neuroimmunology

## Abstract

RNA-binding proteins (RBPs) are key regulators of gene expression that shape cellular function in health and disease. However, the roles of RBPs in immune cells within the central nervous system (CNS) remain poorly understood. Here, we identify ARID5A as an RBP highly expressed in microglia and uncover its RNA-mediated regulatory functions using integrated multi-omics analyses of its RNA, DNA, and protein interactions. ARID5A regulates the splicing and translation of its RNA targets, many of which are integral to lysosomal, immune, and iron metabolism pathways. We confirm the functional relevance of this ARID5A-dependent RNA regulatory network by demonstrating that ARID5A modulates lysosomal activity, cytokine secretion, iron accumulation, and ferroptosis in iPSC-derived microglia. We further demonstrate that knockdown of microglial ARID5A reduces neuronal ferroptosis in co-cultures, underscoring the interconnected nature of these pathways. Moreover, in microglia harboring the TREM2-T66M mutation, ARID5A depletion restores dysregulated lysosomal and metabolic functions. Our results highlight the importance of protein-RNA interactions in regulating microglial cell biology.

## Abstract (web summary)

RNA-binding proteins shape gene expression, but their roles in brain immune cells are unclear. Here, the authors show that ARID5A regulates RNA processing in microglia, linking immune and lysosomal pathways to iron-driven death in neurodegeneration.

## Introduction

RNA-binding proteins (RBPs) govern all stages of the RNA life cycle, and their binding activity is influenced by both cell type and environmental factors^1,2^. Over 2000 RBPs contain conventional RNA-binding domains (RBDs)^1,3^, and our deep learning model predicted an additional 1700 RBPs with non-canonical binding domains^3^, indicating more widespread RNA-binding capacity than initially recognized. Systematic profiling efforts generated transcriptome-wide RNA-protein interaction maps for over 350 RBPs^4^, but many RBPs, especially those with cell-type-specific functions, remain uncharacterized. Dysregulation of RBPs has been linked to neurodegeneration^5^, though non-neuronal RBPs are an open area of research.

Microglia, the brain’s resident macrophages, survey the CNS and respond to pathogenic signals. In the healthy brain, microglia efficiently phagocytose amyloid beta (Aβ), while in Alzheimer’s Disease (AD), Aβ aggregates into plaques that overwhelm microglial clearance capacity, resulting in neuroinflammation^6^. While many RBPs were upregulated in microglial activation signatures^6–8^, their RNA-binding activity and functions remain unexplored.

We identified ARID5A as an RBP enriched in microglial activation and disease-associated signatures. While ARID5A has been shown to regulate immune transcripts in the periphery^9–12^, its role in microglia was unknown. Here, we discover that ARID5A regulates the splicing and translation of transcripts integral to lysosomal, immune, and iron metabolism pathways. We validated these RNA-based regulatory functions with parallel functional assays and delineated iron metabolism as the primary mechanism by which ARID5A modulated ferroptosis. Finally, we demonstrated that ARID5A depletion ameliorated neurodegenerative phenotypes in TREM2-T66M mutant microglia. Taken together, our findings reinforce the interconnection between lysosomal, immune, and iron metabolism pathways and emphasize the importance of RBP-RNA interactions in neurodegeneration.

## Results

### ARID5A is a microglial RBP

**Fig. 1:**
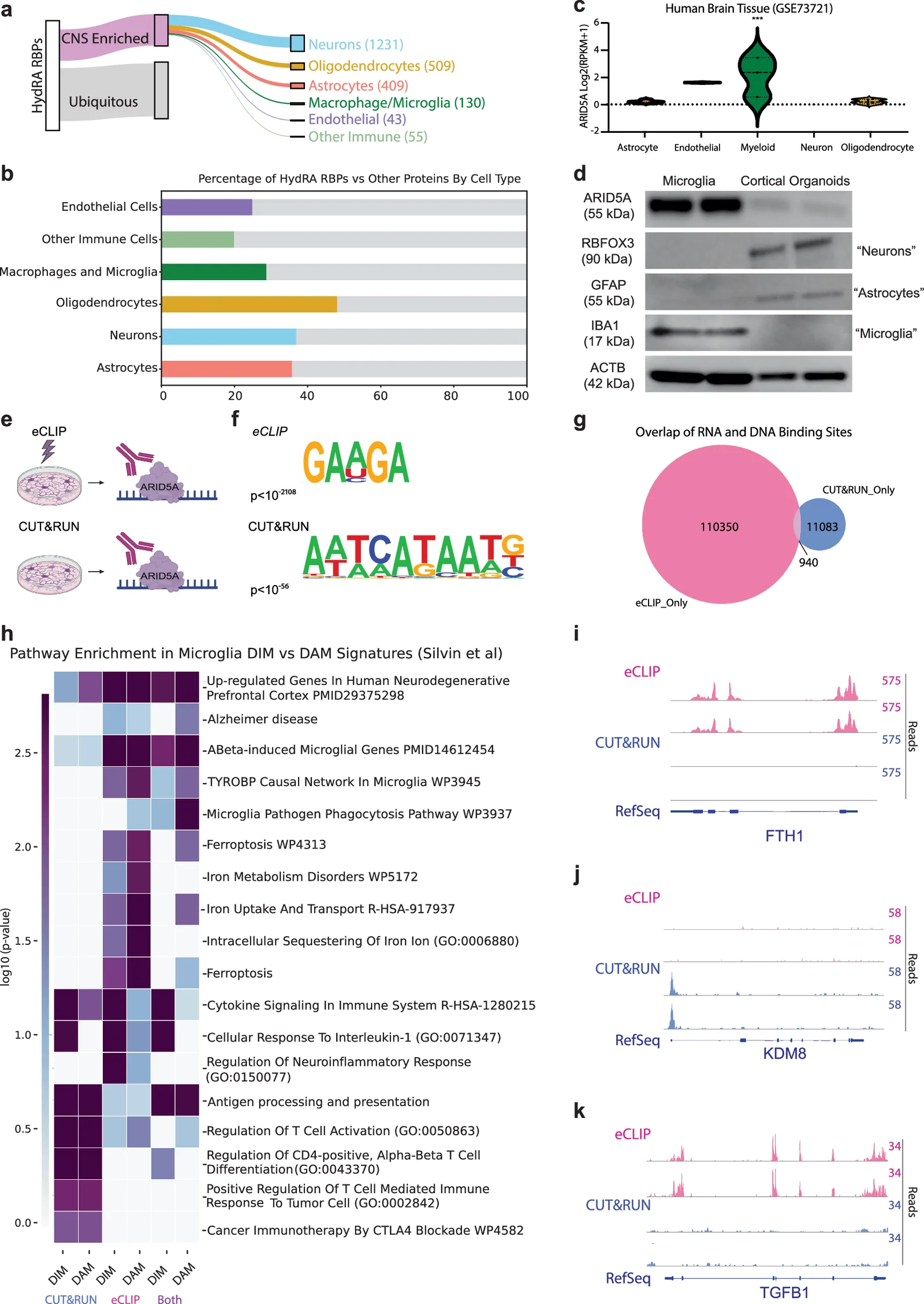
ARID5A is enriched in myeloid cells and binds iron transcripts at the RNA level and immune transcripts at both the DNA and RNA levels. **a** Sankey plot of HydRA predicted RBPs classified by Human Protein Atlas (HPA) cell type annotations. **b** Barplot of percentages of all genes predicted as HydRA RBPs by cell type. **c** Violin plot of *ARID5A* mRNA levels across human CNS cell types (GSE73721). Significance measured by one-way ANOVA with Tukey’s test, *p* < 0.001. **d** Western blotting of iPSC-derived microglia and cortical organoids for ARID5A and markers of CNS cell types. **e** Schematic of experiments for RNA (eCLIP) and DNA (CUT&RUN) binding performed in THP-1 macrophages. Created in BioRender. Blue, S. (2026) https://BioRender.com/od3oemv. **f** Motifs enriched in ARID5A-RNA interactions (GAAGA, *p* < 10^−^^2108^) and ARID5A-DNA interactions (AATA rich, *p* < 10^−^^56^) as calculated by HOMER (one-tailed Fisher’s exact test). **g** Venn diagram depicting the overlap of RNA-binding and DNA-binding sites. Jaccard index = 0.0077. **h** Heatmap of enriched pathways within the Silvin DIM and Silvin DAM signatures for DNA (blue), RNA (pink), and DNA/RNA-bound genes (purple). Significance calculated by Enrichr. **i** Genome browser tracks depicting coverage of RNA binding (pink) and DNA binding (blue) on the RNA-only bound gene *FTH1*. **j** Genome browser tracks depicting coverage of DNA-only bound gene *KDM8*. **k** Genome browser tracks depicting coverage of DNA/RNA-bound gene *TGFB1*.

We classified RBPs according to Human Protein Atlas (HPA) CNS cell type annotations to identify microglial RBPs. The HPA organized 17,862 genes into 56 clusters in the human brain^13,14^, which we summarized into seven major cell type groups: “Neurons,” “Astrocytes,” “Oligodendrocytes,” “Macrophages and Microglia,” “Endothelial,” “Other Immune,” and “Ubiquitous” (Supplementary Data 1). We analyzed experimentally-defined and predicted RBPs from HydRA, a deep learning model informed by sequence features and protein-protein interaction (PPI) networks^3^. The majority were “Ubiquitous” (Fig. 1a), as expected^2,4^, while several RBPs were categorized to specific CNS populations consistent with previous reports: RBFOX3 (NeuN) regulates neuronal-specific transcripts^15^, TLR7 binds viral RNAs in immune cells^16^, and BRD7, while not previously described as an RBP, binds DNA in oligodendrocytes^17^ (Fig. 1b, Supplementary Fig. 1a).

We analyzed publicly available RNA sequencing datasets to prioritize RBPs expressed in disease-relevant microglial signatures and found the DNA/RNA-binding protein ARID5A (Supplementary Fig. 1b) was expressed in the “Activated”^7^ and “terminally inflammatory microglia (TIM)”^8^ signatures (Supplementary Fig. 1c), the latter of which was identified in microglia surrounding amyloid plaques in a mouse model of familial AD^8^. Taken together, these signatures suggest *ARID5A* as a defining pro-inflammatory gene in AD; however, its function in microglia is unknown.

Next, ARID5A expression was validated in human brain datasets and induced pluripotent stem cell (iPSC) derived models. *ARID5A* was enriched at the RNA level in myeloid cells (microglia and macrophages), compared to other CNS cell types^18^ (Fig. 1c) and at the protein level in iPSC-derived microglia in comparison to cortical organoids, which do not contain microglia (Fig. 1d). To confirm the RNA-binding capability of ARID5A^10,11,19^ and HydRA prediction (Supplementary Fig. 1d), we performed an immunoprecipitation assay in THP-1 cells differentiated to macrophages^20^. Lysates were treated with RNase, and bound RNA was biotinylated and visualized. Streptavidin-HRP signal decreased in a UV crosslinking- and RNase-dependent manner, confirming ARID5A-RNA-binding capacity (Supplementary Fig. 1e). Motivated by these findings, ARID5A-RNA interactions were profiled to identify binding targets and regulatory mechanisms.

### ARID5A binds immune transcripts at the CDS and suggests a potential role in splicing

Studies implicate ARID5A in splicing^19^, stability^11,12^ and translation^10,21^, though it is unclear how ARID5A regulates its RNA targets in human myeloid cells. A subset of ARID5A RNA targets was identified in murine peritoneal macrophages^12^, and transcriptome-wide crosslinking and immunoprecipitation (CLIP) in P19 cells revealed ARID5A pre-mRNA interactions^19^. Here, we performed enhanced CLIP^22,23^ (eCLIP) to extend these interactions in THP-1 macrophages (Fig. 1e). Skipper-defined^24^ ARID5A binding targets were enriched in immune-associated transcripts (Supplementary Fig. 2a), consistent with its known RNA-binding targets^10–12,21^.

ARID5A binding patterns were compared to those of RBPs characterized in the ENCODE database^4^ to identify potential mechanisms of RNA regulation. ARID5A predominantly bound the coding sequence (CDS) of its RNA targets (Supplementary Fig. 2b, c), which can be indicative of regulation through splicing, stability, or translation^25^. Binding was also detected in splice sites, introns, and untranslated regions (UTR) (Supplementary Fig. 2b). ARID5A showed a preference for the GAAGA motif (Fig. 1f), which has been observed in splicing-associated proteins^26–33^ (Supplementary Fig. 2d). Its binding repertoire was most similar to splicing proteins BUD13 and SRSF7 and stability-associated proteins UPF1^9^, IGF2BP1, and IGF2BP2^34^ (Supplementary Fig. 2e), indicating that ARID5A may affect its RNA substrates through splicing, as well as stability/translation.

### ARID5A binds RNA at the ARID domain and intrinsically disordered regions (IDR)

As HydRA predicted that ARID5A binds RNA at both the domain and the IDR (Supplementary Fig. 1d), we deleted the ARID domain. Alphafold2^35^ modeling of ARID domain deletion resulted in decreased predicted Local Distance Difference Test (pLDDT) scores (Supplementary Fig. 2f), which have been associated with increased IDR^36^. IDRs bind RNA with high affinity^3^, suggesting that additional structural disorder may increase binding, even though domain-specific binding events may be lost. In addition to a lower pLDDT score, the ARID domain deletion model exhibited fewer alpha helices (Supplementary Fig. 2g). RNA-dependent HRP-Streptavidin signal persisted in HEK293T cells transfected with full-length or ARID domain-deleted ARID5A, indicating that domain deletion did not ablate RNA binding (Supplementary Fig. 2h). Domain-dependent RNA-binding targets were identified by RNA-immunoprecipitation sequencing (RIP-seq) performed in THP-1 macrophages expressing full-length or ARID domain-deleted ARID5A. 887 transcripts were bound only upon domain deletion, consistent with increased IDR (Supplementary Fig. 2i), while 397 transcripts were detected in the initial eCLIP and full-length domain RIP-Seq, but absent upon domain deletion. These transcripts were enriched in DNA repair and transcription pathways (Supplementary Fig. 2j), indicative of RNA-level regulation of transcription machinery at the ARID domain. Overall, these data were consistent with our HydRA prediction and other studies^19^.

### ARID5A DNA and RNA-binding repertoires suggest both distinct and cooperative regulation

ARID family members are transcription factors that bind “AATA” sequences within DNA^19,37^. We performed Cleavage Under Targets and Release Using Nuclease (CUT&RUN)^38^ to identify ARID5A-DNA-binding sites (Fig. 1e) and detected a motif harboring the “AATA” consensus sequence (*p* < 10^−^^56^) (Fig. 1f). In contrast, our top eCLIP motif (*p* < 10^−^^2108^) was purine-rich. Very little overlap was observed at the binding site level, however 2473 transcripts were found to contain distinct RNA and DNA-binding sites (Fig. 1g, Supplementary Fig. 3a). A higher percentage of genes were unique to RNA binding (~80%) than to DNA binding (~60%) (Supplementary Fig. 3b). While this could be due to differences in method sensitivity, differences in binding affinities or additional factors may influence ARID5A-nucleic acid interactions. Overall, these observed differences in motif preferences, binding sites, and bound genes suggest ARID5A regulation of DNA and RNA occurs through distinct mechanisms.

While ARID5A was expressed in “Activation” and “TIM” signatures, all binding repertoires were enriched for ‘Silvin_DIM’ and ‘Silvin_DAM’ signatures (Supplementary Fig. 3c). “Disease inflammatory macrophages” (DIM) were proposed to promote AD pathology, whereas “Disease associated microglia” (DAM) were proposed to combat it^6^. This was surprising given the minimal overlap between DNA and RNA-binding repertoires at the binding site and gene level. Pathway enrichment analysis was performed to investigate differences between the binding repertoires within these two signatures. Immune pathways were enriched in both DNA and RNA-bound repertoires, while iron-related pathways such as “Ferroptosis” were enriched primarily in the RNA-bound repertoire (Fig. 1h). Ferroptosis, an iron-related cell death pathway, is proposed to be driven by microglia in AD^39^, and iron metabolism genes, such as *FTH1*, are part of the DAM signature^6,7,40^. *FTH1* was bound exclusively at the RNA level (Fig. 1i), indicating ARID5A-RNA mediated regulation of iron metabolism. Conversely, *KDM8* was solely DNA-bound, indicating ARID5A-mediated regulation independent of its RNA interactions (Fig. 1j). Immune pathways may be cooperatively regulated as demonstrated by dual DNA/RNA binding of the cytokine transcript *TGFB1* (Fig. 1k). Collectively, these findings suggest ARID5A-RNA interactions may specifically govern ferroptosis, whereas immune pathways may be regulated by both DNA and RNA interactions.

### ARID5A PPI networks are enriched in splicing machinery and neurodegeneration-associated proteins

**Fig. 2:**
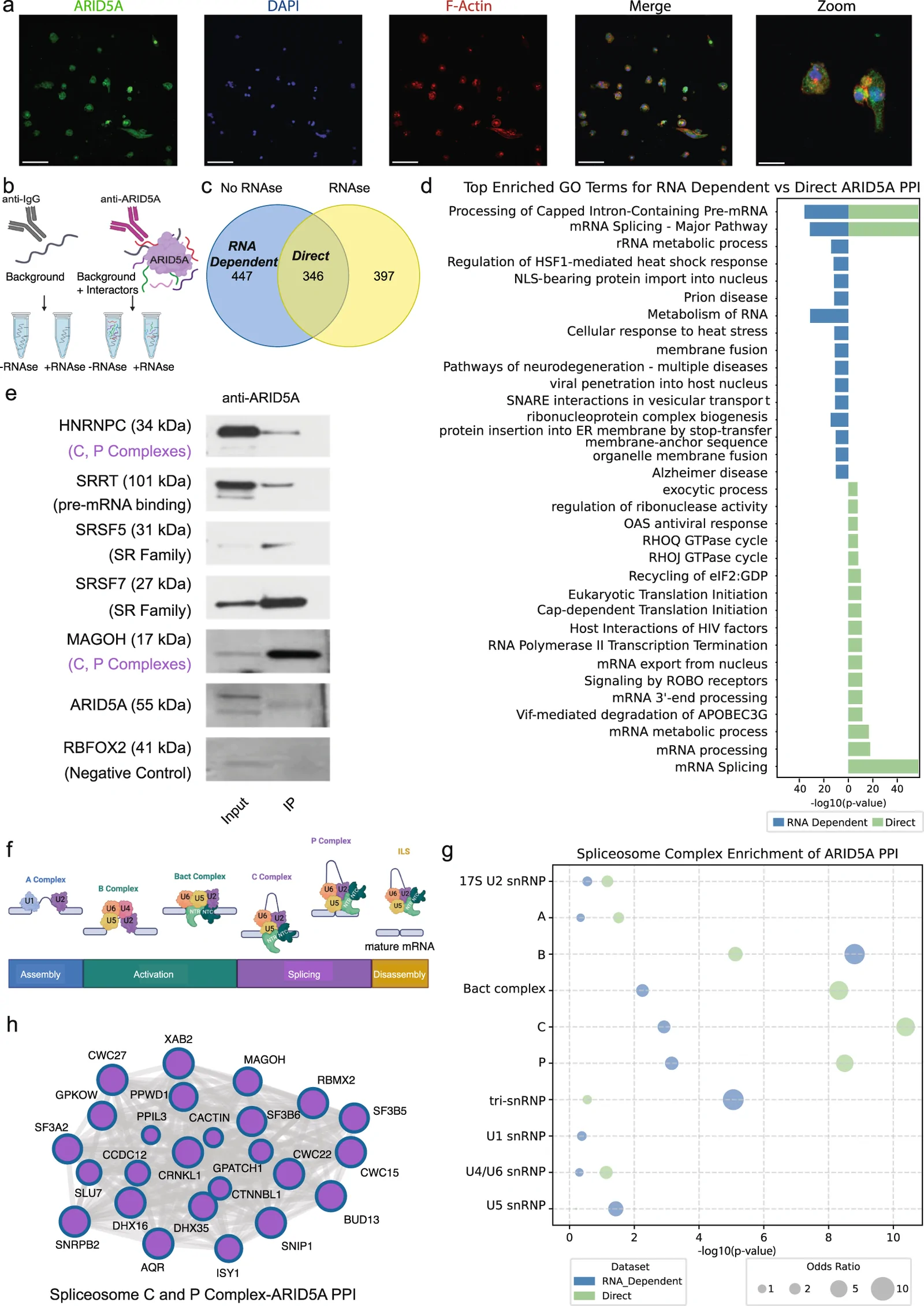
ARID5A interacts with spliceosome machinery at the protein level. **a** Immunofluorescence of ARID5A localization within the nucleus (DAPI) and cytoplasm (F-actin) in THP-1 macrophages (×20, scale 100 μm and 25 μm). **b** Schematic of workflow for affinity-purified mass spectrometry (AP-MS) experiment performed in the presence and absence of RNAse in THP-1 macrophages. Created in BioRender. Blue, S. (2026) https://BioRender.com/a0w64sk. **c** Venn diagram depicting the number of proteins identified as either direct interactions (present regardless of RNAse treatment) or RNA-dependent (present only in RNAse-absent conditions. **d** Barplot depicting MCODE pathway enrichment analysis for direct (green) vs RNA-dependent (blue) ARID5A PPI networks. MCODE clustering and pathway enrichment were performed using Metascape (one-tailed Fisher’s exact test). **e** Co-immunoprecipitation for ARID5A validating the PPI with splicing-associated proteins. RBFOX2 was included as a negative control. **f** Schematic showing complexes formed throughout the four stages of spliceosome regulation of RNA: Assembly (blue), Activation (green), Splicing (purple), and Disassembly (gold). Created in BioRender. Blue, S. (2026) https://BioRender.com/v1zv3x0. **g** Bubble plot depicting −log10(*p*-value) enrichment of ARID5A direct (green) and RNA-dependent (blue) PPIs with spliceosome complexes based on SpliceosomeDB annotations. Odds ratio for each complex is represented by bubble size. Significance and odds ratios were calculated by one-tailed Fisher’s exact test. **h** Protein-protein interaction network of C and P complex spliceosome proteins detected in the ARID5A PPI network generated by Cytoscape. Edges were generated using Metascape based on known interactions. Data are provided as a Source Data file.

Nuclear and cytoplasmic localization of ARID5A (Fig. 2a) indicated splicing and translation/stability regulation, respectively, so we profiled ARID5A-PPIs to further investigate ARID5A-mediated RNA regulation. We immunoprecipitated ARID5A from lysates treated with and without RNase to identify direct (present in both conditions) and RNA-dependent (absent in RNase treatment) protein interactions in THP-1 macrophages (Fig. 2b, Supplementary Fig. 4a-b). We identified 346 direct ARID5A PPIs and 447 RNA-dependent interactors (Fig. 2c) and both PPI networks were enriched for splicing-related pathways (Fig. 2d, Supplementary Fig. 4c-d). We validated the interaction between ARID5A and multiple splicing proteins through co-immunoprecipitation (Fig. 2e, Supplementary Fig. 4e). Consistent with its cytoplasmic localization, ARID5A directly interacted with translation-associated proteins (Fig. 2d). In the RNA-dependent PPI, “Alzheimer’s Disease” and “Pathways of neurodegeneration-multiple diseases” were enriched (Fig. 2d) and included AD-associated proteins VAPB^41^ and GSK3B^42,43^ (Supplementary Fig. 4f–h), which are also implicated in ferroptosis^44,45^. Consistent with ARID5A binding iron transcripts, these PPIs further suggest a role for ARID5A in ferroptosis.

Due to the prevalence of ARID5A-PPIs with spliceosome machinery, we performed enrichment analysis to determine whether ARID5A interacts with protein complexes pertaining to specific stages of splicing, broadly delineated as spliceosome assembly, activation, splicing, and disassembly^46,47^ (Fig. 2f). ARID5A-PPIs were enriched for the C and P complexes (Fig. 2g), which are active during the splicing stage. This included spliceosome-associated BUD13 and EJC protein MAGOH (Fig. 2h), which was further validated as an ARID5A PPI (Fig. 2e). Taken together, ARID5A interactions with spliceosome proteins and its preference for an RNA motif bound by splicing factors implicate ARID5A in alternative splicing.

### ARID5A knockdown results in differential splicing of *GBA* and alters Aβ induced GCase activity and lysosomal response

**Fig. 3:**
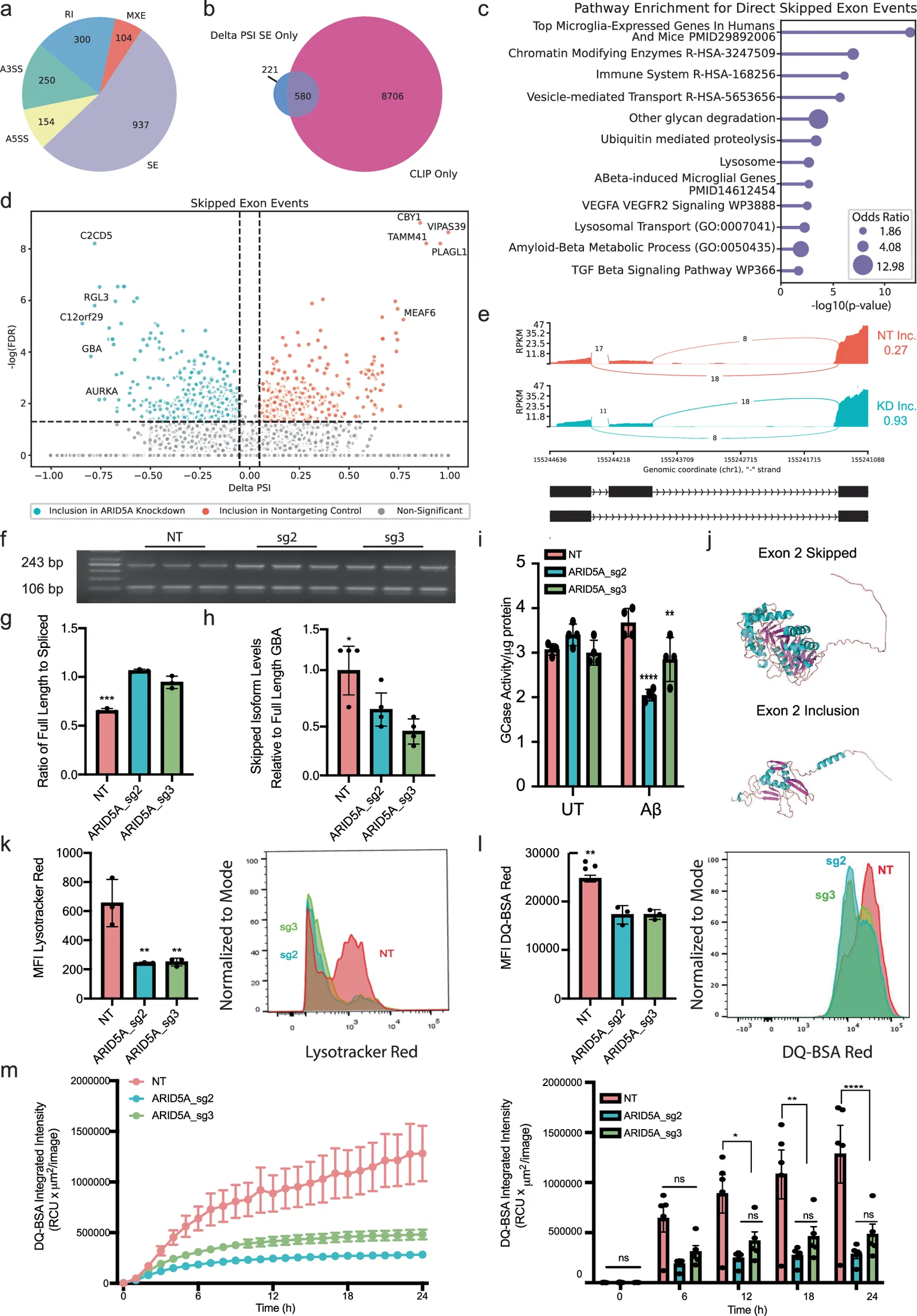
Knockdown of ARID5A results in differential splicing of lysosomal transcripts and altered GCase and lysosome activity. **a** Differential splicing events in control vs ARID5A knockdown iTF microglia (Skipped exons = SE, mutually exclusive exons = MXE, retained introns = RI, alternative usage of 5’/3’ splice sites = A5SS/A3SS. **b** Overlap of genes bound at the RNA level and those containing ARID5A-dependent SE events. Jaccard index = 0.0610. **c** Stick/dot plot of pathway enrichment for SE events. Significance and odds ratios were calculated by Enrichr (one-tailed Fisher’s exact test). **d** Volcano plot of genes containing differential SE events represented by delta percent spliced in (PSI). Negative values indicate exon inclusion in knockdown (blue) and positive values indicate exon inclusion in control (red). An additional significance threshold was set at Delta PSI ≥ |0.05| and −log(FDR) ≥ 1.3. **e** Sashimi plot of *GBA* exon 2 inclusion for NT (red) and knockdown (blue). **f** Gel electrophoresis depicting skipping (106 bp) and inclusion (243 bp) of exon 2. **g** Ratio of full length to spliced quantified by band intensity in (**f**). Significance measured by unpaired two-tailed *t*-test, ***p < 0.001, *n* = 3 independent biological replicates. **h** Abundance of skipped isoform relative to full-length *GBA* quantified by RT-qPCR. Significance measured by unpaired two-tailed *t*-test, *p < 0.05, *n* = 4 independent biological replicates. **i** GCase activity in untreated and 24 h Aβ-treated iTF microglia. Significance measured by two-way ANOVA with Tukey’s test for multiple corrections, **p < 0.01, ****p < 0.0001, *n* = 4 independent biological replicates. **j** Protein structures predicted for *GBA* transcript with exon 2 skipping or inclusion, colored by secondary structure (helices = blue, sheets = pink, strands = coral). **k** Median fluorescence intensity (MFI) of Lysotracker Red in microglia quantified by flow cytometry. Significance measured by one-way ANOVA with Tukey’s test, *p* < 0.01. *n* = 3 independent biological replicates. **l** MFI of DQ-BSA Red in microglia. Significance measured by one-way ANOVA with Tukey’s test, *p* < 0.01, *n* = 3 independent biological replicates. **m** DQ-BSA integrated intensity during Aβ treatment. Significance measured by two-way ANOVA with Tukey’s test, *p < 0.05, **p < 0.01, ****p < 0.0001, *n* = 5 independent biological replicates. For all bar graphs, data are presented as mean values ± SD and provided as a Source Data file.

ARID5A-dependent splicing events were profiled in microglia differentiated from hiPSCs through inducible transcription factor expression (iTF) and CRISPRi-mediated depletion (Supplementary Fig. 5a, b). ARID5A depletion differentially spliced 1745 genes, primarily through exon skipping (SE) (Fig. 3a). 72% of SE events were detected in ARID5A-bound transcripts (Fig. 3b), and included “chromatin modifying enzymes,” consistent with ARID5A’s proposed role in mediating RNA and chromatin interactions^19^. Microglial transcripts and functional pathways such as “Amyloid-beta metabolic process,” “Lysosome,” and “TGF beta signaling” were also enriched (Fig. 3c). ARID5A knockdown resulted in differential exon inclusion within lysosomal transcripts such as *GBA*, which encodes for β-glucocerebrosidase (GCase) (Fig. 3d–h). GCase activity influences alpha-synuclein metabolism^48^, and its dysregulation contributes to Gaucher’s Syndrome^49^ and Parkinson’s Disease (PD)^50^. Given the role of GCase and enrichment of “Amyloid-beta metabolic process,” we hypothesized that differential splicing of *GBA* may lead to altered GCase activity, particularly under Aβ treatment. We incubated lysates from both untreated and fibrillated Aβ-treated (Supplementary Fig. 5c) microglia with a fluorescent substrate to quantify activity. GCase activity was significantly dampened in knockdown microglia under Aβ treatment (Fig. 3i), consistent with a reported 20–80% decrease in GCase activity in PD patients^51^. To assess if changes in activity could arise from aberrant translation, we used AlphaFold2 to model GCase protein with the inclusion or skipping of *GBA* exon 2^35^. Exon 2 inclusion introduced a premature stop codon (Supplementary Fig. 6a, b), resulting in a truncated GCase variant predicted by ProtParam to be unstable (Supplementary Fig. 6a, b). Truncation resulted in the loss of the helices required to form the TIM barrel domain and a beta-sandwich, structures which enable GCase to interact with its substrates^52^ (Fig. 3j). Predicted by Ensembl to be noncoding, the truncated version was not detected via Western blot with canonical anti-GBA antibodies (Supplementary Fig. 6c, d). Collectively, these findings demonstrate how differential splicing regulation can lead to functional consequences in the event of ARID5A depletion.

Consistent with reduced GCase activity, Aβ-induced lysosomal activity was dampened in ARID5A-depleted microglia as measured by Lysotracker and DQ-BSA (Fig. 3k, l). Twelve hours into Aβ treatment DQ-BSA levels, a probe that fluoresces upon lysosomal degradation, were significantly reduced and plateaued in ARID5A-depleted microglia, indicating a potentially slowed rate of lysosome acidification (Fig. 3m). No changes in phagocytosis rate, capacity, or cell death were observed, and lysosomes were detected by LAMP1 staining in ARID5A-depleted microglia (Supplementary Fig. 5d–h). These results support our finding that these changes in lysosomal activity could be due to ARID5A-mediated alternative splicing of *GBA*, or other lysosomal enzymes such as protease *CTSB*, rather than cell death, phagocytic capacity, or loss of lysosomes. While decreased lysosome function can be detrimental^53^, the degree was not severe enough to trigger cell death. In sum, ARID5A depletion resulted in alternative splicing of lysosomal transcripts and dampened Aβ-induced lysosomal activity.

### Depletion of ARID5A leads to enhanced translation of the defense response and increased ISGylation and cytokine secretion

**Fig. 4:**
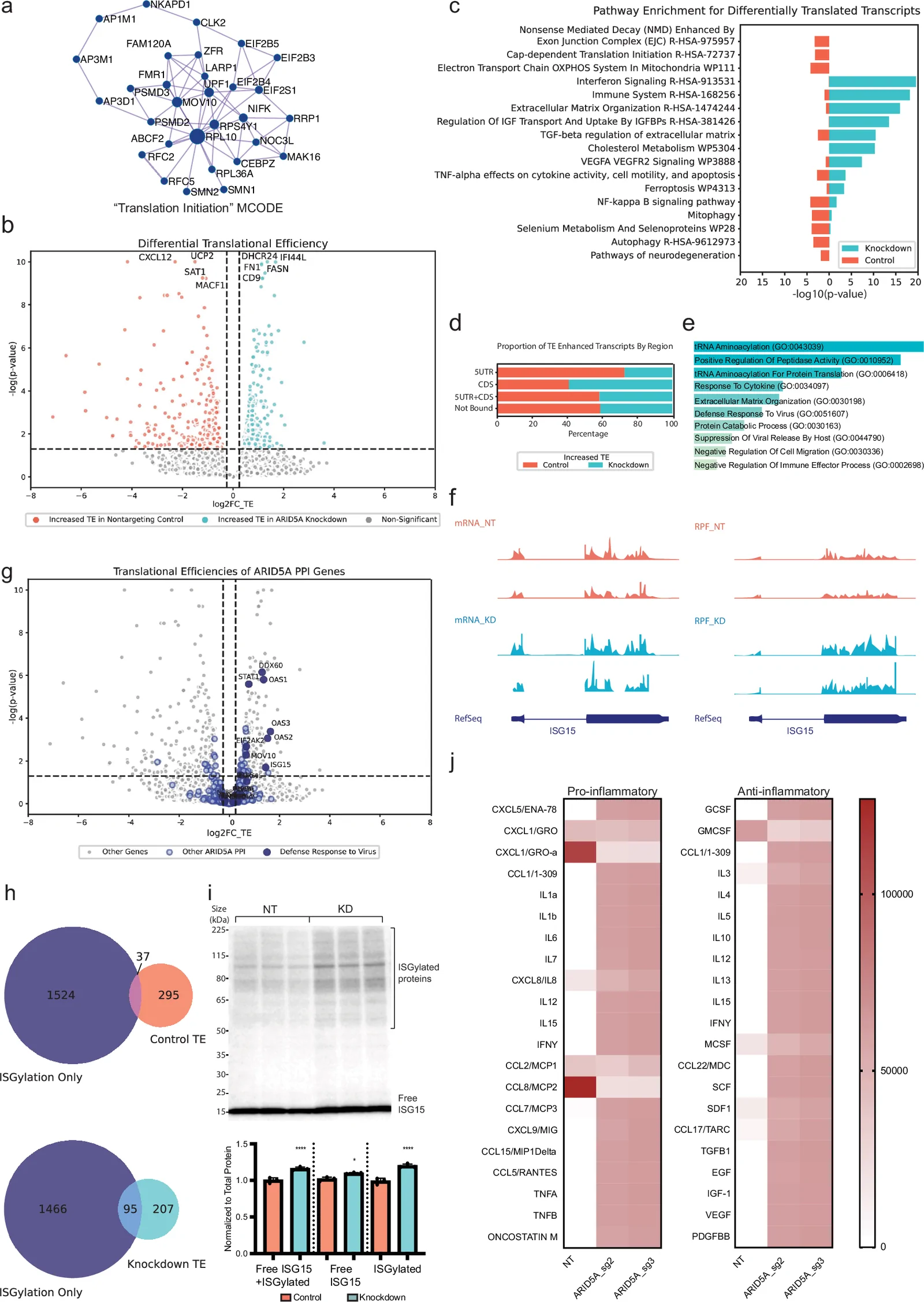
Knockdown of ARID5A results in increased translation of defense response transcripts and enhances ISGylation and cytokine secretion. **a** MCODE “Translation Initiation” cluster from ARID5A PPI networks. **b** Volcano plot of differentially translated transcripts in iTF microglia represented by log2FC of translational efficiency (TE). Negative TE values indicate better translation in the non-targeting control (red), whereas positive TE values indicate better translation in ARID5A knockdown (blue). Dotted lines indicate significance thresholds of Delta TE ≥ |0.25| and *p*-value < 0.05. Significance calculated by the negative binomial model and Benjamini–Hochberg false discovery rate for multiple corrections. **c** Barplot of pathway enrichment for differentially translated transcripts in control (red) vs knockdown (blue) as represented by −log10(*p*-value). Significance calculated by Enrichr. **d** Barplot depicting ratios of control to knockdown TE across 5’UTR and CDS CLIP binding regions. **e** Barplot depicting significant terms of CDS-bound transcripts with enhanced TE upon knockdown. Significance was determined by Enrichr. **f** Genome browser tracks of mRNA input and ribosome-protected RNA fragments (RPF) for control (red) and knockdown (blue) for *ISG15*. **g** Volcano plot depicting TE values of ARID5A PPI transcripts (light blue). Transcripts that are part of the defense response are shown in navy. **h** Venn diagrams depicting overlap of known ISGylation targets and transcripts with enhanced TE in control cells (red) [Jaccard index = 0.0199] and with enhanced TE in knockdown cells (blue) [Jaccard index = 0.0537]. **i** Western blotting of free ISG15 and ISGylated proteins in control and knockdown iTF microglia with quantification normalized to total protein levels. Significance measured by one-way ANOVA with Tukey’s test, *p < 0.05, ****p < 0.0001, *n* = 3 independent biological replicates. Data are presented as mean values ± SD. **j** Heatmap of cytokine secretion in Aβ-treated microglia quantified by cytokine array and normalized to positive control intensity. Significance measured by two-way ANOVA with Tukey’s test, *p* < 0.0001 and shown in Supplementary Fig. 8, with all cytokines significant except CCL2/MCP1 and CXCL1/GRO. Data are provided as a Source Data file.

As ARID5A interacted with many translation-associated proteins (Fig. 4a), we performed ribosome profiling in ARID5A knockdown iTF microglia and quantified changes in translational efficiency (TE). A similar number of genes had enhanced TE in control (332) as in knockdown (302) (Fig. 4b), but were enriched in different pathways (Fig. 4c). ARID5A depletion promoted translation of transcripts implicated in cytokine signaling, notably interferon pathways (Fig. 4c). Overlap of TE values with ARID5A RNA-binding sites showed less efficient translation of transcripts bound at the 5’UTR compared to CDS-bound transcripts upon knockdown (Fig. 4d, Supplementary Fig. 7a). Enhanced translation of CDS-bound transcripts upon knockdown, together with widespread ARID5A–CDS binding (Supplementary Fig. 2b), suggests that ARID5A may suppress translational programs to fine-tune cellular state, consistent with its role in promoting inflammation^12^. In total, 50% of transcripts differentially translated were bound at the 5′UTR and/or CDS, and these CDS sites were enriched for immune pathways (Fig. 4e). Interferon and TNF alpha related pathways were more significantly enriched among indirectly regulated (unbound) translation targets, whereas direct (bound) targets were associated with “VEGFA VEGFR2 Signaling” and “TGF-beta regulation of the extracellular matrix” (Supplementary Fig. 7b). Translation of unbound transcripts may be mediated by ARID5A PPI partners (Figs. 2d, 4a), which was supported by the enrichment of differentially translated transcripts within the ENCODE RNA-binding profiles of translation-associated proteins. This included IGF2BP2, FXR2 and multiple EIF proteins (Supplementary Fig. 7c), suggesting that ARID5A may regulate translation of some transcripts indirectly through interactions with other RBPs.

*ISG15*, a key interferon gene, showed increased TE after ARID5A knockdown without eCLIP binding, indicating its translation regulation may be mediated by an ARID5A PPI partner (Fig. 4f). Indeed, ISG15 and other defense response proteins were detected by AP-MS within “RHO GTPase” and “OAS Antiviral” MCODE clusters (Supplementary Fig. 7d, e). Furthermore, these proteins tended to have significantly higher TE upon knockdown (Fig. 4g), consistent with reports that ISG15 can alter translation by modifying proteins in a process known as ISGylation^54,55^. Defense response proteins can be involved in ISGylation and ISGylation targets themselves^55^, so we compared our differentially translated transcripts with a list of established ISGylation targets^55^ and identified 95 overlapping ISGylation hits in the knockdown TE dataset compared to 37 targets in the control dataset (Fig. 4h). ISGylation and the defense response enable the cell to selectively respond^56,57^, suggesting that ARID5A knockdown leads to increased TE of the defense response directly or through its PPI network. ISG15, or its other pathway members, self-regulate and/or co-regulate, which may contribute to higher TE levels. This was supported by increased free ISG protein and ISGylation signal in ARID5A-depleted microglia (Fig. 4i) and recapitulated in HeLa cells (Supplementary Fig. 7f). Collectively, these data provide strong evidence that ARID5A regulated ISGylation, likely through the modulation of ISG15 translation efficiency.

ARID5A depletion enhanced the TE of cytokine signaling transcripts and, as expected, increased Aβ-induced secretion of VEGF, IGF, and TGFB1 (Fig. 4j, Supplementary Fig. 7g–i). Pro-inflammatory cytokines bound by ARID5A at the RNA level, including IL1B, IL8, and TNF, were also elevated. Enhanced secretion of TGFB1 and TE of “TGF-beta regulation of extracellular matrix” suggested microglial migration would be similarly upregulated, as TGF-beta induces microglial motility through cytoskeletal rearrangement^58^. Microglial migration to Aβ increased upon ARID5A knockdown (Supplementary Fig. 7j, k), in agreement with upregulated secretion of TGFB1 and TE of its signaling pathway. Collectively, these data demonstrated ARID5A depletion may elicit an activation response or “priming” in microglia, a mechanism by which myeloid cells can better respond to a previously encountered stressor or stress response^59^. Primed macrophages have shown resistance to cell death, whereas fibroblasts lack the same resilience when subjected to death-inducing compounds following stress^60^. As transcripts were translationally profiled in unstimulated conditions, the enhancement of cytokine translation may indicate a primed state, similar to a stress response triggered by Aβ. This was consistent with increased Aβ-induced migration and cytokine secretion upon knockdown, as well as the dampened lysosomal response to Aβ (Fig. 3k–m). These results were in agreement with previous reports of cytokine-treated macrophages exhibiting dampened lysosomal activity despite upregulation of lysosomal transcripts and proteins^61^.

Beyond immune signaling, ISGylation regulates additional processes including lysosomal function^57^. As expected, pathway analysis of ISGylation targets with ARID5A-dependent TE showed enrichment of “Defense Response to Virus,” “ISG15 Antiviral Mechanism,” and “Positive Regulation of Cytokine Production” in ARID5A-depleted microglia (Supplementary Fig. 8a). In line with ARID5A regulation of lysosomal activity, “Lysosomal Transport” was also enriched and included *GRN*, a known AD risk gene^62^. “Ferroptosis” was similarly enriched due to increased TE of ferroptosis-suppressors such as *AIFM2*^63^, which was consistent with higher TE of the ferroptosis-inducer *SAT1* observed in control microglia^64^ (Fig. 4b). Collectively, ISGylation highlights a potential hub connecting lysosomal and immune pathways, as well as ferroptosis regulation.

### Knockdown of ARID5A decreases the sensitivity to ferroptosis in microglia and co-cultured neurons

**Fig. 5:**
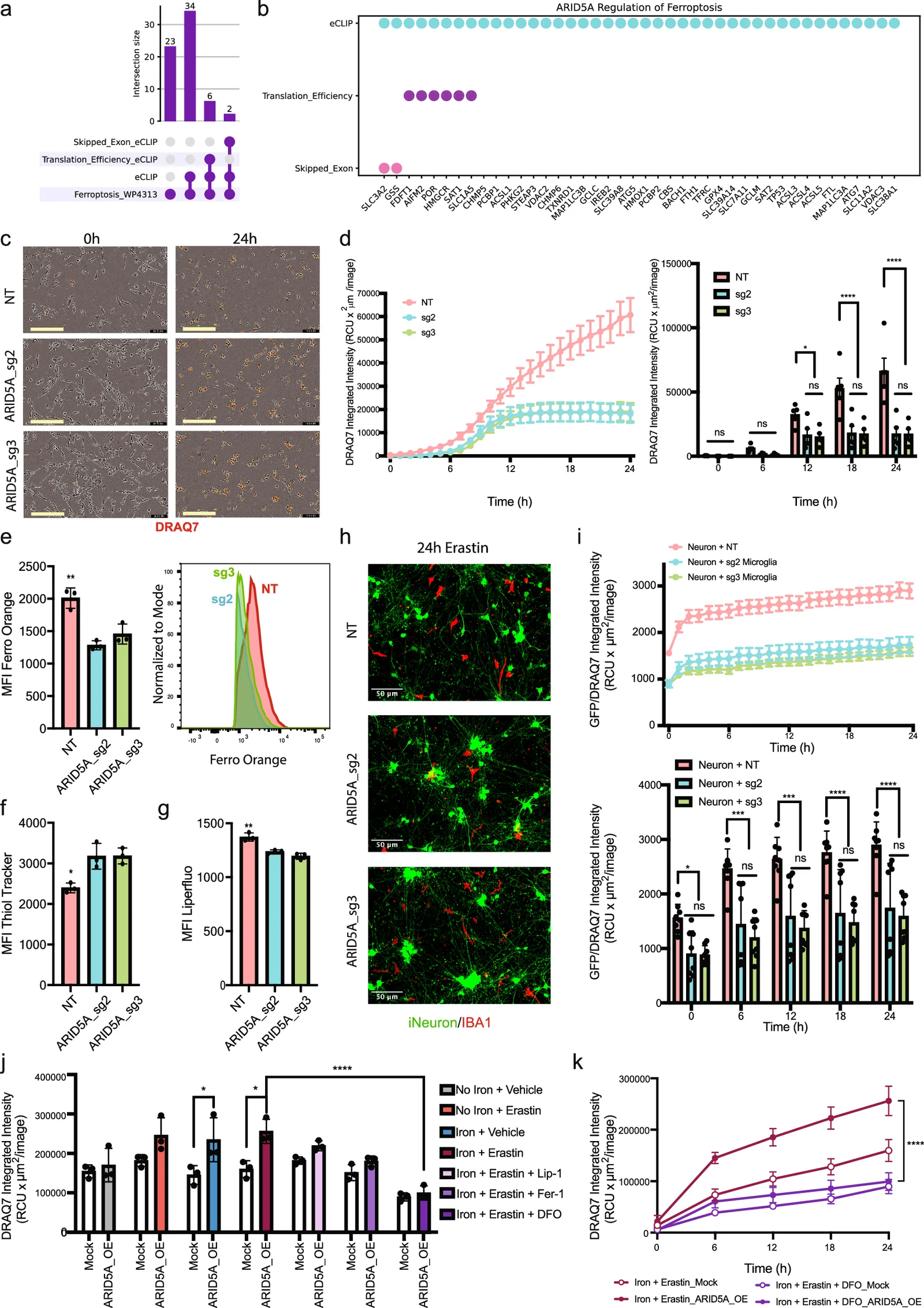
ARID5A sensitizes cells to erastin-induced ferroptosis. **a** UpSet plot depicting the intersection of ARID5A-bound, differentially translated, and differentially spliced transcripts with “Ferroptosis WP4313.” **b** Dot plot of intersecting “Ferroptosis (WP4313) genes” with bound transcripts (turquoise), differentially translated transcripts (purple), and differentially spliced via exon skipping (pink). **c** Incucyte images (×20, scale 200 μm) of erastin-treated iTF microglia at 0, 12, and 24 h labeled with DRAQ7 (red). **d** Timecourse of DRAQ7 integrated intensity during erastin treatment with barplot quantifying integrated intensity at 0, 6, 12, 18, and 24 h. Significance measured by two-way ANOVA with Tukey’s test, *p < 0.05, ****p < 0.0001, *n* = 5 independent biological replicates. **e** Median fluorescence intensity (MFI) of FerroOrange in microglia quantified by flow cytometry. Significance measured by one-way ANOVA with Tukey’s test, *p* < 0.01, *n* = 3 independent biological replicates. **f** MFI of ThiolTracker (GSH) in microglia quantified by flow cytometry. Significance measured by one-way ANOVA with Tukey’s test, *p* < 0.05, *n* = 3 independent biological replicates. **g** MFI of Liperfluo (lipid peroxidation) in microglia quantified by flow cytometry. Significance measured by one-way ANOVA with Tukey’s test, *p* < 0.05, *n* = 3 independent biological replicates. **h** Immunofluorescent staining of induced NGN2 neurons (iNeurons) tagged with GFP co-cultured with iTF-microglia and treated with erastin for 24 h. Microglia were labeled with IBA1 (red) (×20, scale 50 μm). **i** Timecourse of DRAQ7 integrated intensity in iNeurons co-cultured with iTF microglia during erastin treatment. Barplot quantifying integrated intensity of neurons (GFP+). Significance measured by two-way ANOVA with Tukey’s test, *p < 0.05, ***p < 0.001, ****p < 0.0001, *n* = 8 independent biological replicates. **j** Quantification of DRAQ7 integrated intensity in HeLa cells transfected with or without an ARID5A overexpression vector after 24 h of designated treatments. Significance measured by two-way ANOVA with Tukey’s test, *p < 0.05, ****p < 0.0001, *n* = 3 independent biological replicates. **k** Timecourse of DRAQ7 integrated intensity in HeLa cells with or without ARID5A overexpression treated with iron and erastin (pink) or iron, erastin, and iron chelator DFO (purple). Significance calculated by two-way ANOVA with Tukey’s test, ****p < 0.0001, *n* = 3 independent biological replicates. For all bar graphs, data are presented as mean values ± SD and provided as a Source Data file.

ARID5A-RNA interactions were enriched for iron-related transcripts (Fig. 1h), and 42 of the 65 ferroptosis pathway genes were bound with eight differentially regulated by splicing or translation (Fig. 5a, b). Based on the directional shift in TE of ferroptosis-inducers vs suppressors, we hypothesized that ARID5A knockdown would attenuate ferroptosis. Microglia were treated with the ferroptosis-inducer erastin, and cell death was quantified over 24 h using DRAQ7. ARID5A-depleted microglia DRAQ7 levels were significantly reduced and plateaued at 12 h (Fig. 5c, d), indicating attenuation of ferroptosis. This was further evident in morphology, as ARID5A-depleted microglia retained ramified morphology at later stages of erastin treatment relative to control (Fig. 5c). Increased iron accumulation, lipid peroxidation, and concurrently reduced GSH levels were subsequently measured to confirm ferroptotic cell death^45^. ARID5A-depleted microglia exhibited reduced levels of iron accumulation, increased GSH levels, and decreased lipid peroxidation (Fig. 5e–g), confirming ARID5A knockdown attenuated ferroptosis.

Given the ability of microglia to sensitize other cells to ferroptosis^39^, ARID5A-knockdown microglia were co-cultured with iNeurons and treated with erastin (Fig. 5h). Cell death was reduced in iNeurons co-cultured with ARID5A knockdown microglia compared to those co-cultured with control microglia (Fig. 5i), indicative of potential cross-talk between microglial iron and immune pathways to mitigate neuronal ferroptosis. Ferroptosis is driven by iron-dependent lipid peroxidation and requires coordination of iron metabolism and antioxidant pathways^65^. To determine whether ARID5A acts primarily through iron metabolism or antioxidant pathways, ARID5A was overexpressed in HeLa cells treated with erastin in the presence or absence of iron. Cells were co-treated with either the iron chelator DFO or the antioxidant inhibitors ferrostatin-1 and liproxstatin-1^39^. ARID5A overexpression significantly increased cell death in both iron+erastin and iron-only conditions (Fig. 5j). While antioxidant pathway modulation had little effect, iron chelation markedly reduced ferroptosis nearly to wild-type levels (Fig. 5j). In iron+erastin-treated cells, ARID5A overexpression continuously increased cell death, but co-treatment with DFO blunted this effect (Fig. 5k, Supplementary Fig. 8b, c). Collectively, these results indicate ARID5A sensitizes cells to ferroptosis through iron metabolism.

Iron metabolism is regulated through the binding of iron-responsive elements (IREs), which are conserved stem loop structures within the 5’UTR, 3’UTR, and CDS regions of mRNA. Based on our findings that ARID5A binds iron-related transcripts and regulates ferroptosis in an iron-dependent manner, we examined ARID5A RNA-binding sites for IREs. All human 5’UTR, CDS, and 3’UTR FASTA sequences were run through Searching for Iron-Responsive Elements (SIREs)^66^ to identify predicted IREs and intersected with ARID5A RNA-binding sites. 466 predicted overlapping sites were identified, of which 286 mapped to either CDS (34.3%), exon (39.2%) or UTR regions (26.6%) (Supplementary Fig. 8d). Transcripts containing overlapping IRE and ARID5A RNA-binding sites were enriched for immune pathways such as “IL-17 signaling pathway” and “ISG15 Antiviral Mechanism”(Supplementary Fig. 8e). ARID5A regulation of IL-17 was consistent with other studies^10,21^, whereas enrichment in “ISG15 Antiviral Mechanism” further bridges the connection between immune signaling and iron metabolism. ARID5A depletion resulted in differential TE of ISGylated targets that regulate ferroptosis, and conversely, transcripts that regulate ISGylation contained IREs within ARID5A RNA-binding sites. This included *PPM1B*, a target and regulator of ISGylation^67^. ISGylation of *PPM1B* downregulates NF-kappa B activity^68^ consistent with increased ISGylation and downregulated TE of the NF-kappa B signaling pathway upon ARID5A knockdown (Fig. 4c, h, i, Supplementary Fig. 7f). The IRE of *PPM1B* contains the CAGUGG sequence, which overlapped the ARID5A binding site and was detected in the top five significant RNA-binding motifs (Supplementary Fig. 8f–h). Collectively, the identification of IREs within RNA-binding sites integrated ARID5A’s regulation of the immune response and iron metabolism.

### Modulation of ARID5A restores microglial response to Aβ in TREM2-T66M microglia

**Fig. 6:**
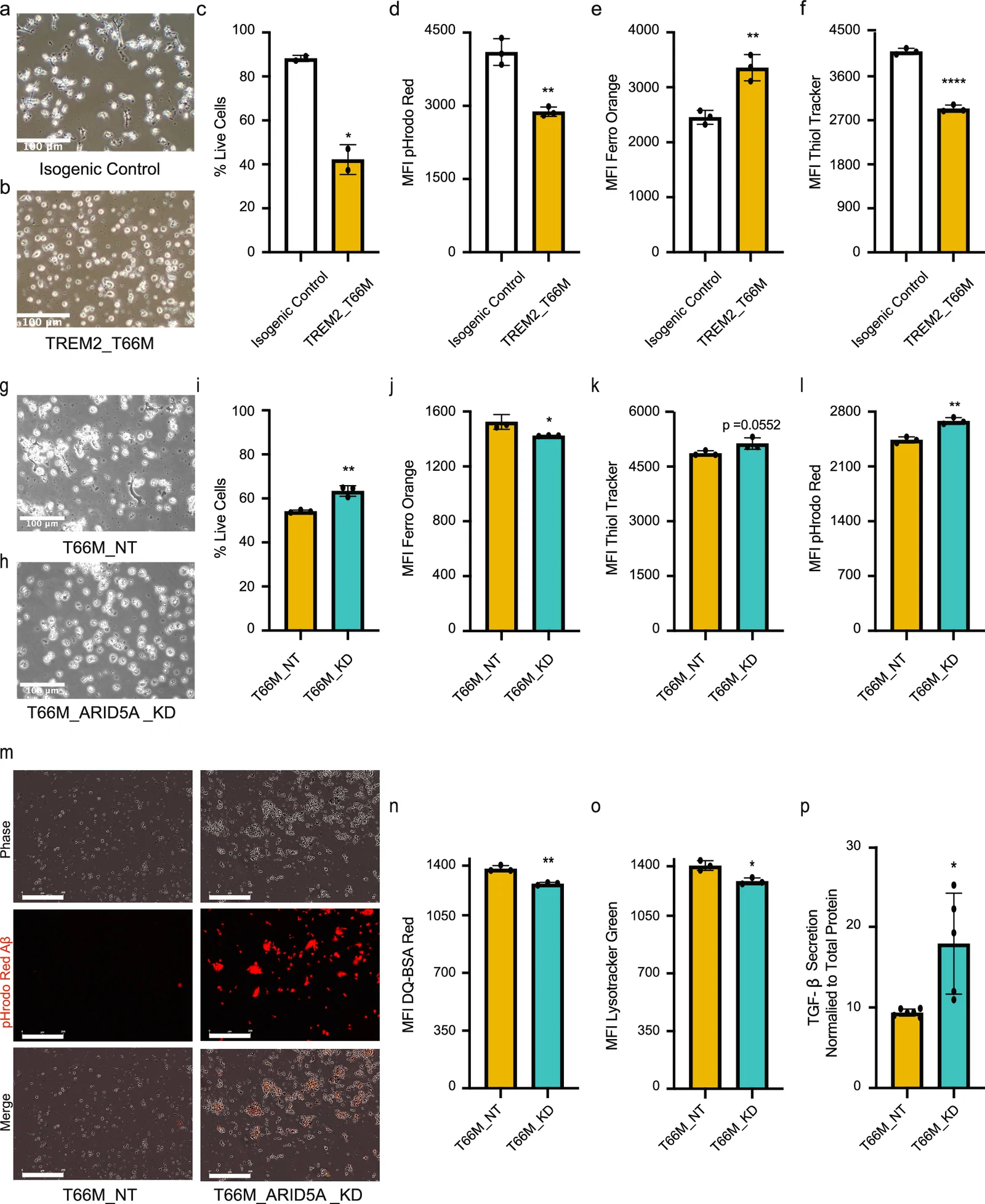
Knockdown of ARID5A in TREM2 T66M mutant microglia restores microglial response to Aβ. Brightfield images (×40, scale 100 μm) of **a** isogenic control and **b** T66M mutant iMGs at Day 30. **c** Percentage of live cells as measured by Zombie Violet and quantified by flow cytometry. Significance measured by unpaired two-tailed *t*-test, *p* < 0.05, *n* = 2 independent biological replicates. **d** MFI of pHrodo red following 24 h treatment of pHrodo red labeled Aβ. Significance measured by unpaired two-tailed *t*-test, *p* < 0.01, *n* = 3 independent biological replicates. **e** MFI of FerroOrange following Aβ treatment. Significance measured by unpaired two-tailed *t*-test, *p* < 0.01, *n* = 3 independent biological replicates. **f** MFI of ThiolTracker following Aβ treatment. Significance measured by unpaired two-tailed *t*-test, *p* < 0.0001, *n* = 3 independent biological replicates. Brightfield images (×40, scale 100 μm) of **g** non-targeting shRNA and **h** ARID5A shRNA knockdown T66M mutant microglia at D30. **i** Percentage of live cells measured with Zombie Violet. Significance measured by unpaired two-tailed *t*-test, *p* < 0.01, *n* = 3 independent biological replicates. **j** MFI of FerroOrange following Aβ treatment. Significance measured by unpaired two-tailed *t*-test, *p* < 0.05, *n* = 3 independent biological replicates. **k** MFI of ThiolTracker following Aβ treatment. Significance measured by unpaired two-tailed *t*-test, *p* = 0.0552, *n* = 3 independent biological replicates. **l** MFI of pHrodo red following 24 h treatment of pHrodo red labeled Aβ. Significance measured by unpaired two-tailed *t*-test, *p* < 0.01, *n* = 3 independent biological replicates. **m** Incucyte images (×20, scale 200 μm) of microglia after 24 h treatment of pHrodo red labeled Aβ. **n** MFI of DQ-BSA red following Aβ treatment. Significance measured by unpaired two-tailed *t*-test, *p* < 0.01, *n* = 3 independent biological replicates. **o** MFI of Lysotracker Green following Aβ treatment. Significance measured by unpaired two-tailed *t*-test, *p* < 0.05, *n* = 3 independent biological replicates. **p** Quantification of TGF-β secretion by ELISA in untreated control (NT) and knockdown (KD) T66M microglia Significance measured by unpaired two-tailed *t*-test, *p* < 0.05, *n* = 5–6 independent biological replicates. For all bar graphs, data are presented as mean values ± SD and provided as a Source Data file.

Dysregulation of immune, lysosomal, and iron metabolism is prevalent in neurodegeneration^69–71^. We hypothesized that modulation of ARID5A would be most impactful in a neurodegenerative disease variant with these phenotypes and thus evaluated the role of ARID5A in TREM2 T66M mutant microglia. *TREM2* was identified as a prominent microglial AD risk gene, and many variants have been broadly implicated in neurodegeneration^72,73^. The T66M variant is characterized by rampant neuroinflammation, dysregulated iron metabolism^74^, and deficits in microglial activity and phagocytic capability^72,74–76^. Consistent with chronic neuroinflammation, T66M microglia upregulated ARID5A protein by approximately 50% (Supplementary Fig. 9a, b) and exhibited a persistent ameboid morphology accompanied by decreased viability (Fig. 6a–c). While cell morphology appeared apoptotic, Caspase 3/7 levels were unchanged (Supplementary Fig. 9c). To determine if the cells were ferroptotic, microglia were treated with Aβ and assayed for iron accumulation, GSH production, and lipid peroxidation. Phagocytosis was also measured to confirm consistency with previous reports^75,76^. Cells were treated with Aβ instead of erastin to elicit a more disease-relevant response, as iron deposits have been correlated with Aβ plaques^77^. As expected, Aβ phagocytosis was reduced in T66M microglia (Fig. 6d). Consistent with ferroptosis, iron accumulation was increased, and GSH levels were decreased in T66M microglia (Fig. 6e, f). No changes in lipid peroxidation were observed (Supplementary Fig. 9d). Taken together, these findings expand on phagocytic deficits and identify ferroptosis as a phenotype in T66M microglia.

Based on our data in healthy cells, we hypothesized that knockdown of ARID5A may decrease Aβ-induced iron accumulation. T66M microglia were treated with an ARID5A-targeted shRNA or non-targeting control (Supplementary Fig. 9e, f). Upon ARID5A knockdown, cells appeared more viable based on increased brightness and a less ameboid morphology (Fig. 6g, h). This was accompanied by an increase in cell viability (Fig. 6i), but no significant difference in Caspase 3/7 levels (Supplementary Fig. 9g). Knockdown of ARID5A reduced iron accumulation (Fig. 6j); however, it had no significant effect on GSH levels (Fig. 6k) or lipid peroxidation (Supplementary Fig. 9h). This is in agreement with our findings that iron chelation mitigated the effects of ARID5A overexpression in HeLa cells, whereas inhibitors targeting antioxidant pathways did not (Fig. 5j, k). While phagocytosis was unchanged upon ARID5A knockdown in healthy cells (Supplementary Fig. 5d–f), phagocytosis increased upon ARID5A knockdown in T66M microglia (Fig. 6l, m). This discrepancy between healthy and disease microglia suggests that restoration of phagocytosis may be due to ARID5A modulation of lysosomal or immune pathways rather than direct modulation of phagocytosis genes. Despite increased phagocytosis, DQ-BSA and Lysotracker levels were reduced (Fig. 6n, o). This was consistent with our observations in healthy cells, which similarly showed enhanced immune signaling accompanied by dampened lysosomal activity (Figs. 3, 4).

ARID5A knockdown in healthy cells enhanced translation of TGF-beta–related transcripts, increased TGF-beta secretion, and promoted migration toward Aβ. Since TGF-beta drives microglial ramification, morphology changes in ARID5A-depleted T66M microglia may be due to enhanced TGF-beta secretion. Aβ-induced secretion of TGF beta was significantly increased nearly two-fold higher in ARID5A knockdown T66M microglia (Fig. 6p). Taken together, our findings in T66M microglia are consistent with those in healthy microglia. In both models, ARID5A knockdown led to reduced iron accumulation, dampened lysosomal activity, and increased secretion of TGF-beta. The reduction in iron accumulation in concert with increased cell viability was similarly observed in our erastin-induced ferroptosis model (Fig. 5). Increased cytokine production in parallel with decreased lysosome activity was consistent with our findings in healthy microglia and previous studies^61^. Increased phagocytosis of Aβ and a shift to a more ramified morphology were only observed in ARID5A-depleted T66M microglia. This discrepancy can be explained by the severe deficits in morphology and phagocytosis that are hallmarks of the T66M mutation, and thus these phenotypes would not be as evident in healthy cells. Finally, secretion of TGF-beta was enhanced upon ARID5A knockdown in both healthy and T66M microglia, and as such, may be driving these changes in morphology and phagocytosis, ultimately allowing the cells to respond more effectively to Aβ.

### ARID5A as a central modulator of immune, lysosomal, and iron metabolism networks in neurodegeneration at the RNA level

**Fig. 7:**
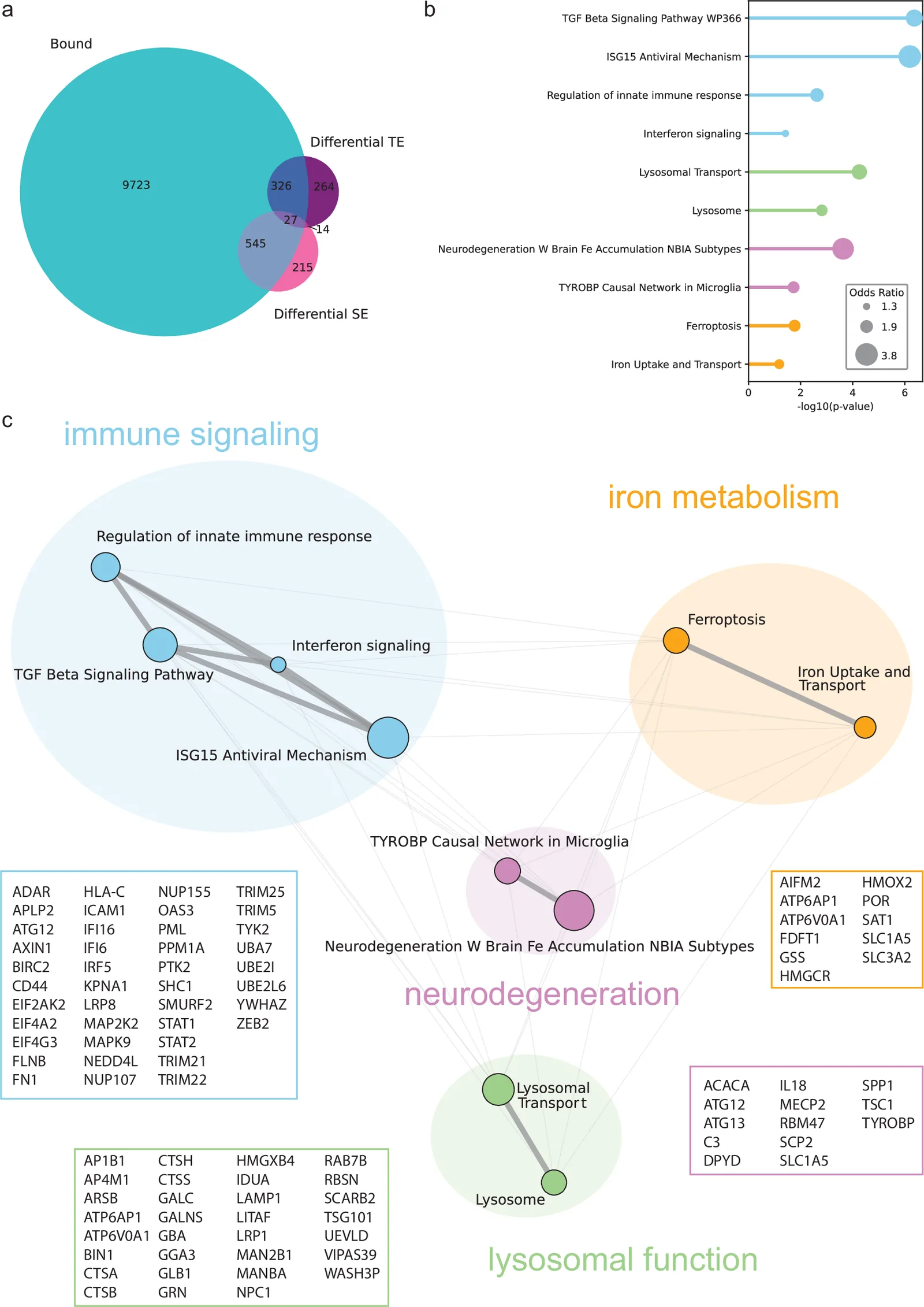
ARID5A regulates transcripts of highly interconnected immune, iron, lysosomal and neurodegeneration pathways. **a** Venn diagram depicting overlap of all ARID5A-bound transcripts with transcripts with differentially skipped exons (SE) and differential translation efficiencies (TE) upon ARID5A knockdown. **b** Pathway enrichment of ARID5A targets regulated at the RNA level (bound, differential TE, and/or differential SE). Pathways are categorized as either involved in immune signaling (blue), lysosomal function (green), neurodegeneration (pink), or iron metabolism (orange). Significance and odds ratios determined by Enrichr (one-tailed Fisher’s exact test). **c** Network diagram of pathways regulated by ARID5A at the RNA level. Connections represent the overall Jaccard index of shared genes between pathways, and nodes represent the odds ratio of enrichment for ARID5A RNA-regulated transcripts (bound, differential TE, and/or differential SE) within each pathway. Boxes show specific transcripts within each pathway found to be direct splicing and/or translation targets of ARID5A.

ARID5A knockdown altered splicing and translation of immune, lysosomal, and iron-metabolism transcripts (Fig. 7a, b), with parallel shifts in functional assay readouts. These pathways are highly interconnected and contribute to neurodegenerative phenotypes (Fig. 7c). Several transcripts associated with AD, PD and ALS were bound and regulated by ARID5A through RNA-based mechanisms (Supplementary Fig. 10a–f). The lysosomal gene *GRN*, known to be protective against AD and regulate immune signaling, was bound and had enhanced TE upon ARID5A knockdown (Fig. 7c, Supplementary Fig. 10d). Additionally, lysosomal transcript *GBA*, a key risk factor in PD, was bound and alternatively spliced upon ARID5A knockdown (Figs. 3, 7c, Supplementary Fig. 10e). The transcript *SQSTM1* (encoding p62), implicated as a negative regulator of ferroptosis^45^ and ALS risk gene, was bound by ARID5A and had higher TE upon ARID5A knockdown (Supplementary Fig. 10f). While several bound transcripts were regulated by splicing or translation, other bound transcripts may be governed by other mechanisms of RNA metabolism such as stability or RNA modifications. This included AD risk genes *APOE* and *TREM2* and ALS risk genes *VCP* and *ATXN2* (Supplementary Fig. 10d, f, g). Collectively, these data highlight the enrichment for ARID5A RNA-regulated targets in immune, lysosomal, and iron pathways that are highly interconnected and dysregulated in neurodegeneration.

## Discussion

We mapped ARID5A RNA, DNA, and protein interactions and demonstrated the significance of ARID5A-RNA interactions in regulating lysosomal activity, immune signaling, and iron metabolism through extensive molecular profiling and functional phenotyping in healthy and TREM2 mutant microglia. We expanded ARID5A-RNA interactions^12,19,21^ to human myeloid cells and, in parallel, profiled its DNA and protein interactions. ARID5A-RNA interactions were enriched for iron-related pathways, whereas dual-bound DNA/RNA transcripts were enriched for immune pathways (Fig. 1), underscoring the importance of profiling RNA vs DNA interactions. Following identification of splicing and translation-associated PPI partners (Fig. 2, Supplementary Fig. 4), ARID5A was confirmed to regulate splicing and translation of its RNA substrates, particularly those enriched in lysosomal, immune, and iron metabolism pathways. Integration of these datasets pinpointed cellular functions governed by ARID5A-dependent RNA regulation and confirmed by functional readouts of lysosomal, immune and iron signaling. ARID5A-dependent alternative splicing of lysosomal transcript *GBA* was observed alongside changes in its enzyme activity and lysosomal function (Fig. 3, Supplementary Figs. 5, 6). Immune transcripts, including *ISG15*, were differentially translated upon ARID5A knockdown, leading to enhanced ISGylation and cytokine secretion (Fig. 4, Supplementary Fig. 7). Multiple mechanisms of ARID5A-dependent RNA regulation of iron metabolism were identified including differential translation, splicing, and the presence of IREs within ARID5A RNA-binding sites (Fig. 5a, b, Supplementary Fig. 8). ARID5A modulated sensitivity to ferroptosis in both microglia and HeLa cells, primarily through iron metabolism (Fig. 5). We further demonstrated that microglial ARID5A depletion reduced ferroptosis in co-cultured neurons, indicating a convergence of iron metabolism and immune signaling pathways.

Our study demonstrated ARID5A regulation of lysosomal, immune, and iron signaling, and emphasized the interconnected nature of these pathways. This was consistent with a growing body of work demonstrating mutual regulation between all three functional hubs. ARID5A knockdown enhanced TE of ISGylation targets implicated in ferroptosis, and reciprocally IREs were detected within ARID5A RNA-binding sites in transcripts that regulate ISGylation (Supplementary Fig. 8), consistent with studies connecting iron and immune signaling^78^. Many lysosomal transcripts differentially spliced by ARID5A (Figs. 3, 7c) also regulate immune signaling. Dysregulation of *GBA* alters cytokine secretion^79^ and cathepsins, a family of lysosomal proteases, regulate the immune response^80^. In macrophages, enhanced cytokine secretion dampened lysosomal function^61^, which was consistent with our findings (Figs. 3, 4). Lysosomes traffic iron and activation of lysosomal iron was pinpointed as an initiator of ferroptosis in cancer cells^81^. Collectively, our data suggest regulation not of three discrete pathways, but rather regulation of a highly interconnected network and points to ARID5A-dependent RNA regulation as a nexus (Fig. 7c).

The importance of examining lysosomal, immune, and iron metabolism pathways as a network rather than discrete hubs is underscored in neurodegeneration (Fig. 7c, Supplementary Fig. 10). ARID5A depletion in TREM2-T66M mutant microglia modulated iron metabolism, immune signaling, and lysosomal activity, as well as ameliorated known phagocytic and cell viability deficits (Fig. 6). ARID5A protein levels were elevated in T66M microglia (Fig. 6, Supplementary Fig. 9), consistent with ARID5A expression in the periphery during chronic inflammation^12^. T66M microglia viability was significantly decreased, but Caspase 3/7 levels were unchanged (Supplementary Fig. 9). This led us to explore iron accumulation as a potential contributing factor to cell death. Indeed, Aβ-induced iron accumulation was increased alongside dampened GSH levels in T66M microglia. Consistent with our findings in healthy microglia, ARID5A depletion significantly reduced iron accumulation, increased cell viability (Fig. 6) and, strikingly, phagocytosis was restored. Lysosomal activity readouts were consistent with those in healthy microglia, so we hypothesized that the changes in phagocytosis may be driven by immune signaling. This was supported by multiple lines of data in our healthy microglia, in which ARID5A depletion enhanced TE and secretion of cytokines as well as migration to Aβ (Fig. 4, Supplementary Fig. 7). Further, ribosome profiling was performed in unstimulated conditions, suggesting a potential priming response. This was evident by increased ramification of ARID5A-depleted T66M microglia at baseline and subsequent increased secretion of TGF-beta, which in turn were consistent with our ribosome profiling data (Fig. 4) and TGF-beta remodeling the extracellular matrix to promote microglial ramification^58^. Collectively, our data indicate that modulation of an RBP can reshape functional networks dysregulated in disease.

Overall, we provide extensive insights into the molecular functions of ARID5A and the effects of its depletion in healthy and TREM2-T66M mutant microglia. While these findings provide foundational rationale for exploring ARID5A-dependent RNA regulation further, additional experiments are required to address limitations of this study. It is still unknown how T66M mutation leads to its phenotypes, and while we propose amelioration was due to increased TE of immune signaling transcripts, other mechanisms are possible. Dysregulated soluble TREM2 levels in T66M microglia are proposed to be due to impaired accessibility of the TREM2 domain^76,82^, which in turn could be regulated by ARID5A-TREM2 interactions at the RNA level (Supplementary Fig. 10) and have downstream effects. Despite these limitations, our findings advance our understanding of ARID5A as a microglial RBP in health and disease and provide the groundwork for future exploration of modulating cell-type enriched protein-RNA interactions in neurodegeneration.

## Methods

### Annotation of candidate RBP cell type, binding and signatures

Candidate RBPs were defined with a HydRA score of greater than or equal to 0.8^3^. Cell-type annotation was assigned based on the Human Protein Atlas^13,14^, and further binned into the categories “Neurons,” “Astrocytes,” “Oligodendrocytes,” “Macrophages and Microglia,” “Endothelial Cells,” “Other Immune Cells,” and “Ubiquitous.” Percentage of RBPs was determined by dividing the number of HydRA predicted RBPs over the total number of cell-type-defining genes (Supplementary Data 1). Candidate RBPs were annotated as DNA binding, RNA binding, or DNA/RNA binding based on GO annotations “DNA binding” (GO:0003677) and “RNA binding” (GO:0003723). Microglial signatures were obtained from the following publications: Silvin et al.^6^, Olah et al.^7^, and Millet et al.^8^. Macrophage/Microglial RBPs were intersected with all signatures to determine whether any of the HydRA RBPs were part of these signatures.

### Validation of ARID5A mRNA expression

ARID5A expression was validated as myeloid-specific in the human brain using Myeloid Landscape 2 with dataset GSE73721^83^. Log2 (RPKM + 1) values were plotted for all cell types (Myeloid, Astrocyte, Endothelial, Neuron, and Oligodendrocyte).

### THP-1 culture and differentiation

All cell types were cultured in humidified incubators at 37 °C, 5% CO_2_ and routinely assayed for mycoplasma contamination. Naive THP-1 cells were maintained in RPMI 1640 (Gibco, #11875093) supplemented with 10% fetal bovine serum (Gibco, #A5256701), 1X GlutaMAX (Gibco, #35050061), 1X MEM Nonessential Amino Acids (Gibco, #11140050), 1X penicillin-streptomycin (Gibco, #10378016) and 0.05 mM 2-mercaptoethanol (Gibco, #21985023). THP-1s were passaged to maintain a density less than 1 × 10^6^ cells per mL. For differentiation^20^, THP-1 cells were plated at a density of 1 × 10^6^ cells per mL onto tissue culture treated plates in maintenance media supplemented with 10 nM phorbol 12-myristate 13-acetate (STEMCELL Technologies, #74044) for 24 h. After 24 h, cells were checked for adherence and media was replaced with maintenance media. Cells were allowed to recover for 48 h prior to experiments.

### iPSC culture and maintenance

WTC11 (Coriell Institute, #GM2526) and KOLF2.1J (Jackson Laboratory, #JIPSC001000) were the human induced pluripotent stem cells (hiPSCs) used in this study. iPSCs were cultured in StemFlex (Gibco, #A3349401) and routinely passaged with Gentle Cell Dissociation Reagent (STEMCELL Technologies, #100-1077) onto 1x Cultrex (R&D Systems, #BME001-05) coated plates prior to differentiation.

### Differentiation of microglia from iPSCs (iMGs)

Microglia were differentiated as previously described^84–86^. In brief, iPSCs were dissociated into clumps and plated at a 1:40 dilution in StemFlex (Gibco, #A3349401) containing 10 µM ROCK inhibitor (Tocris, #1254). After 24 h, media was changed to StemFlex. After an additional 24 h, media was changed to STEMdiff Hematopoietic Kit media A (STEMCELL Technologies, #05310). Cells were further differentiated with media changes per the manufacturer’s instructions. On day 12 and day 14, suspension hematopoietic progenitors were plated onto Cultrex-coated (R&D Systems, #BME001-05) plates in microglia differentiation media (DMEM/F-12 [Gibco, #11320033], 1X GlutaMAX [Gibco, #35050061], 1X MEM Nonessential Amino Acids [Gibco, #11140050], 1X penicillin-streptomycin [Gibco, #10378016], 0.5X N-2 [Gibco, #17502048], 1X B-27 [Gibco, #17504044], 2X insulin-transferrin-selenium [Gibco, #41400045], 400 µM monothioglycerol [Millipore Sigma, #M1753], 5 µg/mL insulin solution [Sigma, #19278], 100 ng/mL IL-34 [Peprotech, #200-34], 50 ng/mL TGF-β1 [Peprotech, #100-25C], and 25 ng/mL M-CSF [Peprotech, #300-25]) for 27 days. On day 28, microglia were re-plated for experiments onto fresh Cultrex-coated plates in microglia maturation media (differentiation media plus 100 ng/mL CX3CL1 [Peprotech, #300-31] and 100 ng/mL CD200 [Novoprotein, #C311]). Experiments were performed on day 30.

### Differentiation of cortical organoids from iPSCs

Cortical organoids were generated based on the protocol from Qian et al.^87^. In brief, iPSC colonies were grown to 70% confluency and dissociated with 1.5 mg/mL collagenase (Thermo Fisher, 17104019) for 60 min at 37 °C. Colonies were washed with DMEM/F12 (Gibco, #11320033) and transferred to a conical tube. Once colonies gravity-settled, DMEM/F12 was aspirated and colonies were gently transferred to an ultra-low attachment dish containing embryoid body (EB) formation media (DMEM/F12 [Gibco,#1132003], 1X GlutaMAX [Gibco, #35050061], 1X MEM Nonessential Amino Acids [Gibco, #11140050], 20% Knockout Serum Replacement [Gibco, #10828028], 0.05 mM 2-mercaptoethanol [Gibco, #21985023], 2 µM dorsopmorphin [Tocris, 309310], and 2 µM A83 [Tocris, #2939]) plus ROCK inhibitor (Tocris, #1254). Media was changed to fresh EB media without ROCK inhibitor on day 3. On day 5, media was changed to neural induction media (DMEM/F-12 [Gibco, #11320033], 1X GlutaMAX [Gibco, #35050061], 1X MEM Nonessential Amino Acids [Gibco, #11140050], 1X penicillin-streptomycin [Gibco, #10378016], 1X N-2 [Gibco, #17502048], 1X B-27 [Gibco, #17504044], 10 µg/mL heparin [Tocris, #2812], 1 mM CHIR9920 [Tocris, #442310], 1 mM SB431542 [Tocris, #161410]). On day 7, organoids were embedded into 18 μL droplets of Cultrex (R&D Systems, #BME001-05) for 30 min at 37 °C and returned to neural induction media. On day 12, organoids were transitioned to differentiation media (DMEM/F-12 [Gibco, #11320033], 1X GlutaMAX [Gibco, #35050061], 1X MEM Nonessential Amino Acids [Gibco, #11140050], 1X penicillin-streptomycin [Gibco, #10378016], 1X N-2 [Gibco, #17502048], 1X B-27 [Gibco, #17504044], 0.05 mM 2-mercaptoethanol [Gibco, #21985023], and 2.5 µg/mL insulin solution [Sigma, #19278]) and placed on an orbital shaker. Excess Cultrex was gently removed on day 20, and cortical organoids were cultured until day 60.

### Western blotting

Cells were lysed in lysis buffer (50 mM Tris-HCl pH 7.4, 100 mM NaCl, 1% NP-40 [Igepal CA-630], 0.1% SDS, 0.5% sodium deoxycholate, 1X protease inhibitor cocktail III [Millipore Sigma, #535140], and 1X phosphatase inhibitor cocktail [CST, #5870]) on ice for 15 min and sonicated for 5 min for 30 s on, 30 s off. Lysates were centrifuged at 15,000×*g* for 10 min at 4 °C to pellet debris, and supernatants were then transferred to a clean tube. Total protein concentration was quantified using the Pierce BCA Protein Assay Kit (Thermo Scientific, #23225). For gel electrophoresis, equal amounts of protein were loaded onto 4–12% Bis-Tris gels (Invitrogen, #NP0321BOX) and subsequently transferred to PVDF membrane stacks (Invitrogen, #IB24002) using an iBlot 2. Membranes were blocked in 5% milk in TBST solution for 60 min at room temperature. Primary antibodies (see Table 1: Antibodies) were diluted in 5% milk in TBST and probed overnight at 4 °C. Secondary antibodies (see Table 1: Antibodies) were diluted at 1:2000 in 5% milk in TBST and probed for 120 min at room temperature. Membranes were rinsed in TBST and developed with ECL (Thermo Scientific, #32106 and Thermo Scientific, #34095) on film. Total protein was validated prior to blocking using the Licor Revert 700 Total Protein Stain and Wash Kit (Licor, #926-11015). Band intensity was normalized to total protein intensity in ImageJ for quantification. All samples represented are independent biological replicates.

**Table 1:** Antibodies

Antibodies	Product information	Dilution	Application
anti-ARID5A	Atlas Antibodies, #HPA023879-100UL Lot 000024429	Assay dependent	Western blotting (1:1000), immunoprecipitation (5–10 µg), immunofluorescence (1:100)
anti-Beta Actin	Cell Signaling Technology, #3700S Lot 21	1:1000	Western blotting
anti-NeuN/Rbfox3	Novus Biologicals, #NBP1-92693	1:1000	Western blotting
anti-GFAP	Dako, #Z033429	1:500	Western blotting
anti-IBA1	Novus Biologicals, #NB100-1028 Lot 87C7G2P59	Assay dependent	Western blotting (1:1000), immunofluorescence (1:500)
anti-SRRT	Fortis Life Sciences, #A304-551A Lot 1	1:1000	Western blotting
anti-MAGOH	Proteintech, #68293-1-Ig (20 µL) Lot 10030681	1:1000	Western blotting
anti-RBFOX2	Fortis Life Sciences, #A300-864A Lot 4	1:1000	Western blotting
anti-HNRNPC	MBL International, #RN052PW Lot 001	1:1000	Western blotting
anti-SRSF5	MBL International, #RN082PW Lot 001	1:1000	Western blotting
anti-SRSF7	MBL International, #RN079PW Lot 001	1:1000	Western blotting
anti-GSK3B	Cell Signaling Technology, #9832S Lot 5	1:100	Immunofluorescence
anti-LAMP1	Cell Signaling Technology, #15665S	1:500	Immunofluorescence
anti-LC3B	Cell Signaling Technology, #3868 Lot 14	1:500	Immunofluorescence
anti-GBA	Cell Signaling Technology, #88162 Lot 1	1:1000	Western blotting
anti-ISG15	Proteintech, #15981-1-AP Lot 00140703	1:1000	Western blotting
anti-V5	Fortis/Bethyl, # A190-120A (RIP-seq)	Assay dependent	Immunoprecipitation, Western blotting (1:1000)
anti-V5	Cell Signaling Technology, # 80076 Lot 4	1:1000	Western blotting
HRP anti-rabbit secondary	Cell Signaling Technology, #7074 Lot 34	1:2000	Western blotting
IRDye 800 anti-mouse IgG secondary	LI-COR Biosciences, #926-32210 Lot D50121-08	1:2000	Western blotting
Goat anti-Rabbit IgG (H + L) Highly Cross-Adsorbed Secondary Antibody Alexa Fluor 594	Invitrogen, #A27034 Lot 2446823	1:250	Immunofluorescence
Goat anti-Mouse IgG (H + L) Highly Cross-Adsorbed Secondary Antibody, Alexa Fluor 647	Invitrogen, #A31570 Lot 2064098	1:250	Immunofluorescence
Donkey anti-Goat IgG (H + L) Cross-Adsorbed Secondary Antibody, Alexa Fluor 633	Invitrogen, #A21082 Lot 1889311	1:250	Immunofluorescence
Alexa Fluor 488 Phalloidin	Invitrogen, #A12379 Lot 2615834	165 nM	Overnight (4 °C)
TrueBlot anti-rabbit IgG HRP	Rockland Immunochemicals, #18-8816-33 Lot 50455	1:2000	Western blotting for immunoprecipitation

### Visualization of nucleic acid binding with biotin-labeled RNA

THP-1 cells were differentiated into macrophages in biological duplicates and UV crosslinked. Pellets were lysed on ice for 15 min and treated with a low (4 U/µL) and high (33.3 U/µL) concentration of RNase I (Invitrogen, #AM2294). RNA was immunoprecipitated overnight using an ARID5A antibody (Table 1: Antibodies). Immunoprecipitated samples were subjected to low and high salt washes and a biotinylated cytidine (Bis) phosphate (Jena Bioscience, #NU-1706-BIO) was ligated on. Samples were run on a 4–12% Bis-Tris gel (Invitrogen, #NP0321BOX) and transferred to a nitrocellulose membrane at 30 V overnight at 4 °C. Membranes were developed per the manufacturer’s instructions using the Chemiluminescent Nucleic Acid Detection Module kit (Thermo Scientific, #89880).

### Preparation and sequencing of eCLIP libraries

eCLIP was performed as previously described^22,23^. Two replicates were generated and crosslinked. UV crosslinked pellets were lysed on ice for 5 min and then sonicated and treated with RNase I (Invitrogen, #AM2294) to fragment RNA. Two percent of the sample was saved for a size match input (SMInput), and the rest was immunoprecipitated overnight at 4 °C with the ARID5A antibody (Table 1: Antibodies). Immunoprecipitated RNA fragments were dephosphorylated, and an RNA adaptor was ligated onto the 3’-end. The SMInputs and immunoprecipitated samples were run on an SDS-polyacrylamide gel and transferred to a nitrocellulose membrane at 30 V overnight at 4 °C. Regions from 55 kDa (ARID5A size) to 130 kDa were excised from the membrane and treated with Proteinase K to release the RNA. The SMInputs were then subjected to dephosphorylation and 3’ RNA adaptor ligation. Both the SMInputs and immunoprecipitated samples were reverse transcribed to cDNA with SuperScript III Reverse Transcriptase (Invitrogen, #18080093). A DNA adaptor was then ligated on the 5’-end of all samples. qPCR was used to quantify libraries, which were subsequently amplified with Q5 High Fidelity PCR Master Mix (NEB, #M0492S). Sequencing was performed using the NovaSeq 6000 platform, with a targeted number of single-end reads of 30 M per sample.

### Analysis of eCLIP

Analysis of eCLIP data was performed using default settings of Skipper^24^ (available on Github [https://github.com/YeoLab/skipper]) and mapped to human genome assembly GRCh38. Reproducibly enriched windows were defined as windows containing significant enrichment of reads above the SMInput in both immunoprecipitation samples. Motifs were analyzed using HOMER and compared to existing ENCODE 3 datasets using the ATtRACT database^88^.

### Preparation, sequencing, and analysis of RIP-seq libraries

THP-1 macrophages were nucleofected with full-length ARID-domain deleted constructs with V5 tags in biological duplicate and crosslinked. UV crosslinked pellets were lysed on ice for 5 min and sonicated. Lysates were immunoprecipitated overnight at 4 °C with the V5-antibody (Table 1: Antibodies). 2% of the samples were set aside as inputs prior to washes described in *Preparation and sequencing of* *eCLIP libraries*. For the final wash, RNA was eluted from the beads using TRIzol. RNA was extracted using phenol-chloroform and libraries were prepared as described below for RNA sequencing libraries. For both conditions, gene counts were normalized with DESeq to determine IP enrichment over inputs. Pathway enrichment was performed using Enrichr.

### Preparation and sequencing of CUT&RUN libraries

CUT&RUN was performed with the Epicypher CUTANA CUT&RUN kit per manufacturer instructions (Epicypher, #14-1048). In brief, samples were crosslinked with light crosslinking (1% formaldehyde) per the manufacturer’s recommendation for immune cells. ARID5A antibody (Table 1: Antibodies) was used for immunoprecipitation, as well as the positive and negative controls included within the kit. Two independent biological replicates were used for each immunoprecipitation. All libraries were sequenced using the Novaseq 6000 with approximately 10 M paired-end reads per sample.

### Analysis of CUT&RUN

Data analysis was performed using a modified version of CUT&RUN Tools 2.0.98 as previously described^33^. In brief, adapters were removed using two rounds of trimming. Reads were aligned to human genome assembly GRCh38. PCR duplicates were removed with Picard and peaks were called using MACS2 and normalized to negative control. Peaks were then analyzed with Chipseeker and reproducible peaks were determined using BedTools. Motif analysis was performed with HOMER with the negative control as a background control.

### Integration of RNA and DNA-binding repertoires and analysis

BED files containing genomic coordinates for RNA and DNA-binding sites, respectively, were intersected using BedTools to identify overlapping binding sites. Shared genes were defined as either containing a DNA-binding site or an RNA-binding site, but not necessarily overlapping. Unique genes were calculated by dividing the number of genes found only in RNA or only in DNA by the total number of genes detected in each binding repertoire. To determine enrichment within microglial signatures, a Fisher’s exact test was performed. Pathway enrichment was performed using Enrichr^89^. Binding repertoire similarity was calculated by Jaccard index.

### Preparation of affinity purification mass spectrometry samples

Cells were differentiated as described and lysed. In brief, cell lysates were immunoprecipitated using an ARID5A antibody (Table 1: Antibodies). Two replicates were treated with RNase I and all samples were washed on beads in the following buffers: NP-40 buffer four times, wash buffer 1 (50 mM Tris [pH 7.5], 150 mM NaCl, 10 mM MgCl_2_, 0.05% NP-40, and 5% glycerol) twice, and buffer 2 (50 mM Tris [pH 7.5], 150 mM NaCl, 10 mM MgCl_2_, and 5% glycerol) twice. Beads were resuspended in trypsin buffer (2 M Urea, 50 mM Tris [pH 7.5], 5 μg/mL trypsin) to digest proteins and incubated at 37 °C for 60 min. Beads were centrifuged at 100×*g* for 30 s, and the supernatant was transferred to a new tube. The beads were washed two more times with Urea buffer (2 M Urea, 50 mM Tris [pH 7.5]), and each supernatant was subsequently added to a new tube each time. Disulfide bonds were reduced with 5 mM dithiothreitol (DTT). Cysteines were then alkylated with 10 mM iodoacetamide. Samples were digested for 16 h at 25 °C using trypsin and then acidified with 1% formic acid. Peptides were desalted on C18 StageTips as described in Rappsilber et al.^90^ and evaporated in a vacuum concentrator. Samples were then reconstituted in 3% acetonitrile/2% formic acid.

### Analysis of affinity purification mass spectrometry

LC-MS/MS analysis was performed with a Q-Exactive HF as previously described^91^. Peptides were analyzed on a Waters M-Class UPLC using a 25 cm Ion-Opticks column (1.7 µm, C18, 75 µm × 25 cm) coupled to a benchtop Orbitrap Q Exactive HF. Peptides were separated at 400 nL/min with a 120 min gradient and data were acquired in data-dependent mode. MS1 spectra were measured at 120,000, an AGC target of 3e6 and the mass range from 300 to 1800 m/z. MS2 spectra were measured with a resolution of 15,000, an AGC target of 1e5 and a mass range from 200 to 2000 m/z. MS2 isolation windows of 1.6 m/z were measured with a normalized collision energy of 25. Raw data were analyzed by Spectronaut v16.090 using the UniProt database (*Homo sapiens*, UP000005640), and MS/MS searches were performed with BGS factory settings. Global median normalized protein intensity values were used for analysis; a pseudocount of 1 was added to all values, they were log2 transformed, and interacting proteins were identified. Interacting proteins had *p*-values less than 0.05 and log2FC greater than 1 when compared to their RNase treatment matched control IPs. To identify RNA-dependent and direct interactions, no RNase and RNase treated proteins were overlapped. Proteins detected in the no RNase and absent in the RNase-treated conditions were defined as RNA-dependent and proteins that were detected in both conditions were defined as direct. MCODE analysis was performed using Metascape^92^ to identify significantly enriched terms and clusters. Enrichment within spliceosome complexes was performed using the complexes defined by SpliceosomeDB^46^ and determined by Fisher’s exact test.

### Co-immunoprecipitation of splicing proteins

Cells were lysed, and 98% of the samples were immunoprecipitated and subjected to low salt and high salt buffers, with the remaining 2% run as input controls. Samples were run on a 4–12% Bis-Tris gel (Invitrogen, #NP0321BOX) and transferred to PVDF membranes (Invitrogen, #IB24002) using an iBlot 2. Membranes were blocked with 5% milk in TBST for 60 min at room temperature and incubated with primary antibodies (Table 1: Antibodies) overnight at 4 °C. Membranes were rinsed and probed with TrueBlot (Rockland, #18-8816-33) for 120 min and developed with ECL (Thermo Scientific, #32106 and Thermo Scientific, #34095) on film.

### Immunofluorescence

Cells were fixed with 4% paraformaldehyde at room temperature for 30 min and rinsed with DPBS (Gibco, #14190144). Samples were then permeabilized with 0.1% Triton-X for 15 min, and blocked with SuperBlock (Thermo Scientific, #37515) for 60 min at room temperature. Cells were incubated with primary antibody (Table 1: Antibodies) in SuperBlock overnight at 4 °C, and rinsed with DPBS the next day. Samples were incubated in secondary antibodies (Table 1: Antibodies) diluted in SuperBlock at room temperature for 90 min, protected from light. Cells were washed with DAPI (Thermo Scientific, #62248) at 1 µg/mL for 5 min and then washed with DPBS. Cells were imaged at ×40 using a BZ-X Keyence microscope or at ×20 using the Andor Dragonfly Spinning Disk Confocal microscope.

### Cloning of CRISPRi guides

CRISPRi sgRNA lentivirus plasmids were generated as previously described^93–95^. In brief, sequences for CRISPRi guides were obtained from the CRISPRi v2 H1 library^94^. Guides were annealed and ligated into the pMK1334 backbone (Addgene, #127965). Resulting constructs were transformed onto carbenicillin-containing LB-agar plates and grown at 37 °C for 16–18 h. Single colonies were picked and grown in liquid cultures containing carbencillin with agitation for 16 h. Cultures were prepped following the manufacturer’s instructions using QIAprep Spin Miniprep kit (Qiagen, #27104) or ZymoPURE II Midiprep kit (Zymo Research, #D4200) based on liquid culture size. All constructs were validated using Plasmidsaurus whole plasmid sequencing.

### Lentivirus generation

Lenti-x HEK293T (Takara, #632180) cells were cultured in DMEM (Gibco, #11965092) and 10% FBS (Gibco, #A5256701). Cells were seeded at 60% and transfected with plasmids of interest along with third-generation helper plasmids and Opti-MEM (Gibco, #31985062). After 72 h, the media were harvested and spun down to remove debris. Supernatants were then concentrated with Lenti-X Concentrator (Takara Bio, #631232) overnight at 4 °C. Per manufacturer’s instructions, supernatants were spun down and resuspended in 1:100 of the initial volume in DMEM. Aliquots were stored at −80 °C until later transduction experiments.

### Generation of induced microglia (iTFs)

WTC11 iPSCs harboring inducible microglial transcription factors and constitutive CRISPRi machinery^93^ were transduced with CRISPRi sgRNA lentiviruses using TransIT (Mirus Bio, #MIR 6600). After 48 h, iPSCs were subjected to 1 µg/mL puromycin (Gibco, #A1113803) selection for 5 days. Cells were maintained in StemFlex (Gibco, #A3349401) and media was replenished every other day. Once a stable population was selected, cells were expanded. Induced microglia (iTFs) were generated as previously described^93^. Briefly, iPSCs were dissociated to single cells using Accutase (Gibco, #A11105-01) and plated onto Cultrex-coated poly-D-lysine precoated BioCoat plates (Corning, assorted product numbers) in Essential 8 media (Gibco, #A15169-01) containing 10 µM ROCK inhibitor (Tocris, #1254) and 2 µg/mL doxycycline hyclate (Sigma-Aldrich, #D9891) to induce microglial transcription factors on day 0. On day 2, media was replaced with iTF media (Advanced DMEM/F12 Medium [Gibco, #12634-010], 1X Antibiotic-Antimycotic [Gibco, #15240-062], 1X GlutaMAX [Gibco, #35050061], 2 µg/mL doxycycline hyclate [Sigma-Aldrich, #D9891], 100 ng/mL IL-34 [Peprotech, #200-34] and 10 ng/mL GM-CSF [Peprotech, #300-03]). On day 4, media was changed to iTF media, additionally supplemented with 50 ng/mL M-CSF (Peprotech, #300-25) 50 ng/mL TGF-β1 (Peprotech, #100-25C). This media formulation was maintained for the remainder of the differentiation with media changes on day 6 and day 8. Treatments were started on day 8 and cells were assayed on day 9. For co-cultures, iTF microglia were dissociated with TrypLE Express (Gibco, #12604013) for 5 min.

### Generation of NGN2 induced neurons (iNeurons) and co-culture with iTF microglia

Neurons were differentiated as previously described^95^. In brief, WTC11 iPSCs were maintained in StemFlex (Gibco, #A3349401) and transduced with lentiviruses for NGN2, GFP, and p2A puro. After 48 h, iPSCs were subjected to 1 µg/mL puromycin (Gibco, #A1113803) selection for 5 days. Media was changed every other day. Once a stable population was selected, cells were expanded. On day 1, iPSCs were treated with 10 µM ROCK inhibitor (Tocris, #1254) and 2 µg/mL doxycycline hyclate (Sigma-Aldrich, #D9891) to induce differentiation. The next day, media was changed to neuronal maturation media (BrainPhys Neuronal Culture Media [STEMCELL Technologies, #05790, 1X GlutaMAX [Gibco, #35050061], 1X penicillin-streptomycin [Gibco, #10378016], 1X N-2 [Gibco, #17502048], 1X B-27 [Gibco, #17504044], 1X MEM Nonessential Amino Acids [Gibco, #11140050], 0.075% sodium bicarbonate [Gibco, #25080094], 200 µM L-ascorbic acid [Sigma-Aldrich, #A0278], 20 ng/mL BDNF [Peprotech, #420-02]], 20 ng/mL GDNF [Peprotech, #450-10], 10 ng/mL NT-3 [Peprotech, #450-03], 2 µg/mL laminin [Sigma-Aldrich, #L2020], and 0.5 mM db-cAMP [STEMCELL Technologies, #100-0244]) supplemented with 2 µg/mL doxycycline hyclate (Sigma-Aldrich, #D9891). On day 3, cells were dissociated to single cells using Accutase (Gibco, #A11105-01) for 20 min and re-plated onto Cultrex-coated BioCoat plates into neuronal maturation media containing 10 µM ROCK inhibitor and 2 µg/mL doxycycline hyclate. On day 5, media was supplemented with 10 µM cytarabine/AraC (Tocris, #4520) and 2 µg/mL doxycycline hyclate to remove glial cell contamination. On day 7, cells were transitioned back to neuronal maturation media only and fed every 3–4 days. On day 14, day 8 iTF microglia were plated onto iNeurons in neuronal maturation media supplemented with iTF microglial factors (2 µg/mL doxycycline hyclate [Sigma, #D9891], 100 ng/mL IL-34 [Peprotech, #200-34] and 10 ng/mL GM-CSF [Peprotech, # 300-03], 50 ng/mL M-CSF [Peprotech, #300-25] and 50 ng/mL TGF-β1 [Peprotech, #100-25C]). On day 16, cells were subjected to treatments and collected on day 17.

### Treatments

Cells were treated as indicated with either Aβ or erastin. To form aggregates, Aβ 1-42 (Anaspec, #AS-20276) was incubated at 37 °C for 48 h at 300 rpm. Aggregation was validated by Thioflavin T incorporation. Cells were treated with 3.3 µg/mL of amyloid beta for 24 h. For phagocytosis experiments, 1 mg of Aβ was labeled with pHrodo red (Invitrogen, #P36600) at 37 °C for 60 min at 500 µM in 0.1 M sodium bicarbonate buffer (pH 8.4). Labeled protein was spun down at 13,000×*g* for 5 min at 4 °C and rinsed with DPBS (Gibco, #14190144) twice. Aβ was resuspended in media and added to cells as described above. For erastin treatments, erastin (Selleck Chemicals, #S7242) was resuspended in DMSO and diluted in media at a final concentration of 10 µM. Cells were treated for 24 h and a corresponding vehicle control was included by adding equivalent volumes of DMSO (0.03%).

### Transwell migration

D8 iTF microglia were seeded onto the apical layer of a 24-well transwell plate (Corning, CLS3422) and incubated with 3.3 µg/mL of amyloid beta for 24 h. At the end of the incubation, a cotton swab was used to remove nonmigratory cells from the apical membrane before fixation with cold methanol. Inserts were stained with 1% crystal violet for 20 min and rinsed three times with ddH_2_O. Crystal violet-stained cells were lysed in 10% acetic acid with shaking for 30 s and quantified on a plate reader at 595 nm. These absorbances were compared to a standard curve of cells plated to correspond absorbance with known cell number.

### Cytokine arrays

Cytokine secretion was quantified using the 42 target Abcam Human Cytokine Antibody Array (Abcam, #ab133997). After 24 h of Aβ treatment, media were collected from two independent biological replicates and spun down at 15,000×*g* at 4 °C for 10 min to pellet cell debris. Media was transferred to a new tube and stored at −80 until further processing. Samples were thawed on ice and incubated with cytokine dot arrays overnight at 4 °C. Membranes were rinsed and developed per the manufacturer’s instructions. Pixel intensity was quantified using ImageJ and normalized to the positive control. Significance was determined by two-way ANOVA with Tukey’s test for multiple corrections using GraphPad Prism.

### Incucyte live cell imaging

Cells were plated onto Cultrex-coated CellBIND plates (Corning, #3340) and imaged in the Incucyte S3 every 60 min with four images acquired per well over the course of 24 h. Images were captured at ×20 in the phase and red channels (pHrodo red [Invitrogen, #P36600], DRAQ7 [Abcam, #ab109202] and DQ-BSA [Invitrogen, #D12051]). Image analysis was performed using Incucyte S3 analysis modules to identify cells and count fluorescent-positive cells. Integrated intensity was calculated by normalizing fluorescence to phase cell count, and standard deviation was calculated by image per condition. Each condition included three independent biological replicates. Significance was measured by two-way ANOVA with Tukey’s test for multiple corrections or unpaired two-tailed *t*-test using GraphPad Prism.

### Flow cytometry-live cell dyes

Cells were labeled with dyes as described (Table 2: Live cell dyes) in FluoroBrite DMEM (Gibco, #A1896701) and prepared in biological triplicate. Samples were then rinsed twice with DMEM and collected in DMEM containing Zombie Violet (BioLegend, #423133) at a 1:1000 dilution and placed on ice. Cells were analyzed on a BD Fortessa x20 and gated based on an unstained control. Samples were gated for cells, single cells, live cells, and then populations of interest. Approximately 10,000 events per sample were collected. Median fluorescence intensity was quantified using FlowJo. All statistical analysis was performed using GraphPad Prism, and significance was determined by one-way ANOVA with Tukey’s test for multiple corrections.

**Table 2:** Live cell dyes

Dye name	Product information	Concentration	Incubation time
FerroOrange	Cell Signaling Technology, #36104	1 µM	30 min
LysoTracker Red DND-99, special packaging	Invitrogen, #L7528	50 nM	5 min
LysoTracker Green, DND-26, special packaging	Invitrogen, #L7526	50 nM	5 min
ThiolTracker Violet	Invitrogen, #T10095	10 nM	30 min
Zombie Violet	BioLegend, #423133	1:1000	15 min
DRAQ7	Abcam, #ab109202	3 µM	15 min
DQ-BSA Red, special packaging	Invitrogen, #D12051	10 µg/mL	24 h
pHrodo Red, succinimidyl ester	Invitrogen, #P36600	500 µM	60 min
Incucyte Caspase-3/7 Dye for Apoptosis	Sartorious, #4440	5 µM	24 h
Liperfluo Lipid Peroxidation	Dojindo, #L248-10	2 µM	30 min

### Protein modeling

FASTA sequences (full mRNA) for *GBA* transcripts with and without exon 2 were downloaded from Ensembl. Sequences were uploaded to ExPASy Translate^96^ to generate translated protein sequences. Frame 3 was selected as the appropriate reading frame based on Uniprot annotation of the canonical GBA protein. ProtParam was run to determine additional protein characteristics including stability. Protein sequences were then uploaded to AlphaFold2 using ColabFold^35^ and run through default settings. The top seed PDB file for each *GBA* transcript was visualized in Pymol. For ARID5A modeling, Uniprot sequences with and without the ARID domain were uploaded to ColabFold and run through default settings.

### GCase activity

Following the treatments described above, cells were washed with DPBS and lysed in lysis buffer. Lysates were sonicated as described above and spun down to remove debris. Total protein was quantified by BCA (Thermo Scientific, #23225). Equal amounts of protein from four independent biological replicates were brought to equivalent volumes of lysate and resuspended in McIlvaine’s Buffer (0.2 M citrate/phosphate buffer, pH 5.4) containing 0.25% Triton X-100, 22 mM of the activator sodium taurocholate hydrate (Sigma, #86339), and 5 mM of the fluorescent substrate 4-Methylumbelliferyl β-d-glucopyranoside (Sigma-Aldrich, #M363). 100 μL of samples were loaded in triplicate to a 96-well assay plate and incubated for 60 min at 37 °C protected from light. The reaction was terminated using a stop solution (25 μL of 0.25 M glycine/NaOH, pH 10.4) and liberated 4-methylumbelliferone was measured at λex = 365 nm/λe on a FLx800 spectrofluorimeter (BioTek). Fluorescence was normalized to total protein and significance was determined by one-way ANOVA with Tukey’s test for multiple corrections using GraphPad Prism.

### Splicing gel validation of GBA exon skipping

Three wells per condition of iTF microglia were lysed for 5 min in 300 µL of TRIzol reagent (Invitrogen, #15596026). RNA isolation was performed using the Direct-zol RNA Microprep Kit (Zymo Research, #R2060) per manufacturer’s instructions. The optional DNase step was performed for each extraction to ensure removal of DNA contamination. Total RNA was quantified on the Agilent Tapestation 4150. RNA was converted to cDNA using the ProtoScript II First Strand cDNA Synthesis Kit (NEB, #E6560L). cDNA was amplified by primers flanking exon 2 using GoTaq Green (Promega, #M7122) and subsequently run on a 3% agarose gel for 45 min at 95 V. Band intensity was calculated in ImageJ to quantify the ratio of full length (243 bp) to spliced (106 bp) transcript. Significance was measured by unpaired two-tailed *t*-test in GraphPad Prism.

### Splicing qPCR validation of GBA exon skipping

cDNA from iTF microglia was prepared as described for the splicing gel validation above. RT-qPCR was performed using SYBR green and a primer set independent of the splicing gel primers to validate exon skipping.

### RNA sequencing library preparation

iTF microglia were lysed for 5 min in 300 µL of TRIzol reagent (Invitrogen, #15596026). RNA was isolated using chloroform phase-separation as previously described^97^. Total RNA and quality were measured using Agilent Tapestation 4150, and samples with a RIN > 9.5 were selected for library preparation. RNA was normalized to 100 ng and prepared using the Illumina Stranded mRNA Prep, Ligation kit per manufacturer’s instructions. Libraries were sequenced using the Illumina NovaSeq 6000 at a depth of 50 million paired-end reads.

### Differential splicing analysis

Reads were mapped using STAR alignment and converted to BAM files. Differential splicing events were detected using rMATS 4.0.2^98^. Significant splicing events were defined as absolute value of inclusion level differences greater than 5% and an FDR less than 1%. Skipped exon events were overlapped with eCLIP binding sites found in the CDS, CDS start/stop sites, splice sites, and intron regions to determine direct skipped exon genes. Pathway enrichment was performed using Enrichr. Sashimi plots of skipped exon events were generated using the rmats2sashimi plot package.

### Ribosome profiling and analysis

Cells for ribosome profiling were harvested as previously described^99^. In brief, cultured cells in 15 cm were treated with 100 μg/mL cycloheximide for 1 min at 37 °C, followed by two washing steps with ice-cold PBS supplemented with 100 μg/mL cycloheximide. Then, cells were lysed with lysis Buffer (12.5 mM Tris, pH 7.0; 7.5 mM Tris, pH 8.0; 150 mM NaCl; 5 mM MgCl_2_; 100 μg/mL cycloheximide; 1 mM DTT; 1% (v/v) Triton X-100; 20 U/mL DNase), which were then collected and centrifuged 16,000×*g* for 10 min at 4 °C.

Cell lysate was treated with 250 U RNase I (Thermo #AM2295) at 25 °C for 45 min. The digestion was quenched with 200 U SUPERase Inhibitor (Thermo #AM2694) and transferred to 4 °C. Samples were loaded on sucrose solution (34% sucrose, 20 mM Tris pH 7.5, 150 mM NaCl, 5 mM MgCl_2_, 1 mM dithiothreitol and 100 μg mL^−1^ CHX) and spun for 1 h at 100,000 rpm in a TLA-110 rotor (Beckman) at 4 °C. Then, the polysome pellet was resuspended in 1 mL TRIzol (Thermo #15596026), and RNA was extracted following the manufacturer’s protocol by chloroform-based separation. 8 µg of total RNA was used for loading, followed by gel extraction of 28–34 nt mRNA fragments using ladder fragments with 15% UREA-TBE gel for ribosomal footprints. Ribosome profiling libraries were then processed as described^99,100^.

For analysis, sequenced reads were aligned as previously described^101^. Briefly, IDT linker (CTGTAGGCACCATCAAT) and poly(A) sequences were removed. Then, the remaining reads were aligned to rRNA. Reads that failed to align after the previous process were used for the alignment for GRCh38 human sequence using Bowtie v.1.1.229, and a maximum of two mismatches per read were allowed. Unaligned reads were used for alignment to sequences that span splice junctions. The ribosome P site was calculated using the 5′ end of the reads using the canonical ORFs with +12 for reads that were 28–29 bp and +13 for reads that were 30–33 bp. Direct translational events were defined as present in the 5’UTR and/or CDS eCLIP bound genes.

### Genome browser visualization

Tracks of genomic data were visualized using Integrated Genomics Viewer (IGV) and input as BAM (eCLIP/CUT&RUN) or bigwig (RiboSeq) files.

### Generation of shRNA knockdown iMGs

MISSION lentiviral shRNA plasmids targeting ARID5A (TRCN0000157147) and a non-targeting control (SHC002V) were transformed and miniprepped. Lentiviruses were generated as described. iPSCs from the iNDI consortium harboring a homozygous T66M TREM2 mutation (Jackson Laboratory, JIPSC003084) were transduced for 48 h with non-targeting and ARID5A shRNA. Cells were selected as described and dissociated to single cells. Single clones were selected and further expanded. Microglia (iMGs) were differentiated as described.

### ELISA

Conditioned media were collected from iTF non-targeting control and ARID5A knockdown microglia and T66M non-targeting control and knockdown ARID5A iMGs at day 30. Media was spun down to remove debris and transferred to a new tube. Samples were processed and measured for absorbance using the LEGEND Max Total TGF-β1 kit (Biolegend, #436707) and LEGEND Max Total TNF-alpha (Biolegend, #430207). Absorbance was measured on a Tecan Infinite m200 Pro at 450 nm and 570 nm. Background values (570 nm) were subtracted from sample values (450 nm) and concentrations were determined using the standard curve and a 4PL calculation. Concentrations were normalized to total protein measured by BCA (Thermo Scientific, #23225) derived from the corresponding plated cells. Significance was determined by unpaired two-tailed *t*-test using GraphPad Prism.

### Neurodegeneration risk gene overlap analysis

ALS Risk genes were defined by ALS Association, PD risk genes by a previously published dataset^102^, and AD genes by the Alzheimer’s Disease Sequencing Project (ADSP). ADSP “Loci” and “Causal genes” were merged and intersected with overlapping ARID5A eCLIP data, skipped exons, and translated transcripts. Overlapping ARID5A skipped exons were determined as present in relevant eCLIP regions (CDS, CDS start/stop, introns, and splice sites), and rMATS SE analysis and overlapping translation events were determined as present in the relevant eCLIP regions (5’UTR and CDS) and ribosome profiling.

### Statistics

The statistical tests used for each experiment are described in each section of the “Methods.” In brief, two-tailed *t*-tests were performed for comparisons between two groups, one-way ANOVA with Tukey’s test for multiple corrections for comparisons between three or more groups, and two-way ANOVA with Tukey’s test for multiple corrections for comparisons between three or more groups in which there were two independent variables (i.e., cell line and treatment condition). For enrichment of gene signatures, a one-tailed Fisher’s exact test was performed. Enrichr and Graphpad Prism were used to run statistical tests. The tests used to determine significance and odds ratios for programs such as Skipper, RMATS, and HOMER are further described in the original publications.

### Reporting summary

Further information on research design is available in the Nature Portfolio Reporting Summary linked to this article.

## Supplementary information

Supplementary Information

Description of Additional Supplementary Files

Supplementary Data 1: Cell-type annotation of HydRA RBPs based on the Human Protein Atlas

Reporting Summary

Transparent Peer Review file

## Source data

Source data

## Data Availability

Raw data and processed sequencing files are available on GEO (GSE290625, GSE290765, GSE290755, and GSE328331). Proteomics data is available on MassIVE (MSV000097123). Source data are provided with this paper.
