# Supplementary material for: ARID5A RNA-binding coordinates microglial defense and ferroptosis in iPSC-derived models
Source: Nat Commun. 2026 Aug 12;17:9687. doi: 10.1038/s41467-026-76131-0 (PMC13558697; doi:10.1038/s41467-026-76131-0)

*Supplementary Figures*

a

| | Ubiquitous | Neurons | Oligodendrocytes | Astrocytes | Macrophages and Microglia | Endothelial Cells | Other Immune Cells |
|---|---|---|---|---|---|---|---|
| HydRA RBPs | 5984 | 1231 | 509 | 409 | 130 | 43 | 55 |
| Ex. RBP | FUS | RBFOX3 | BRD7* | ALDH1L1* | TLR7 | CDH5* | CD2* |

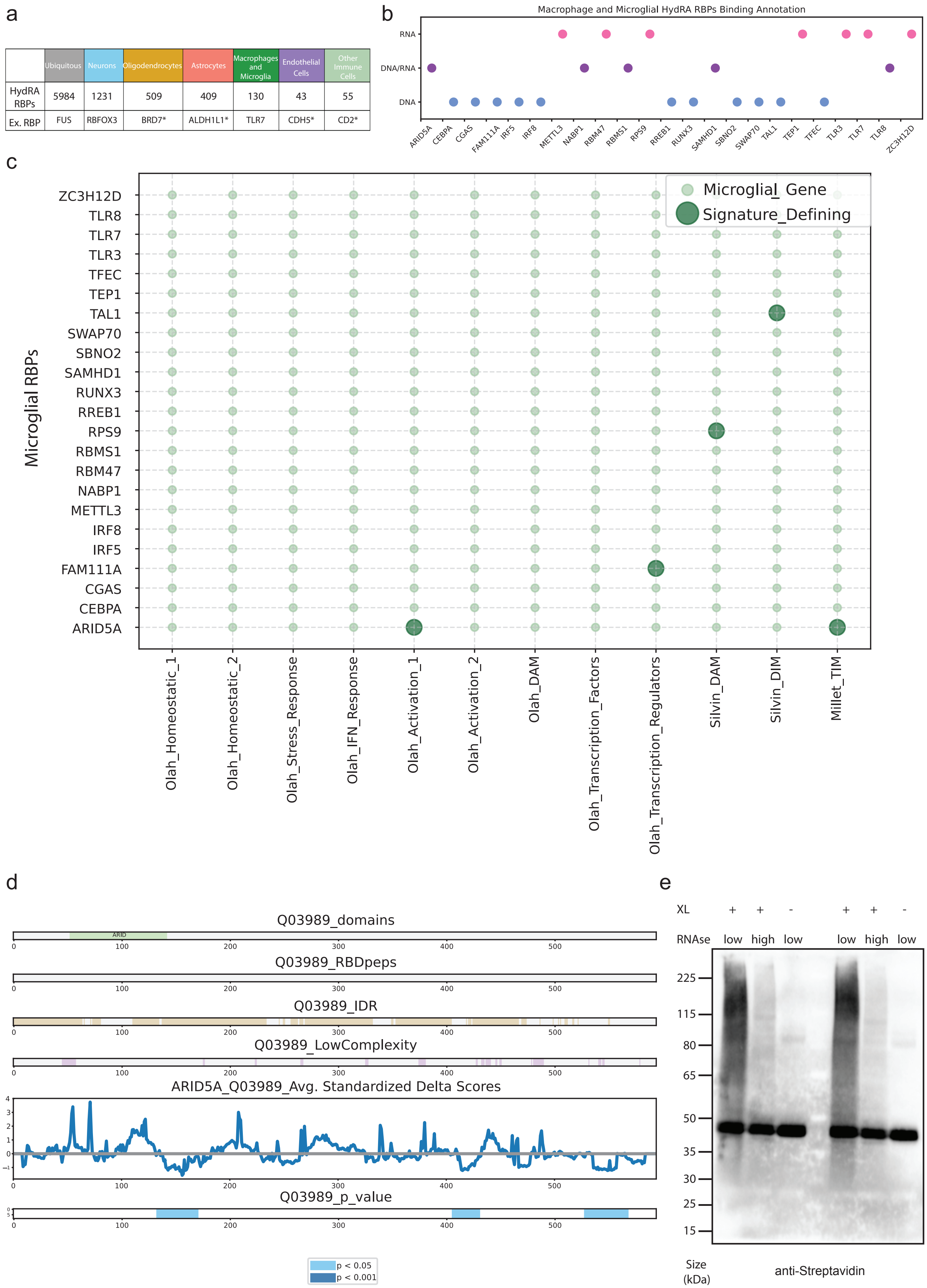

**Supplementary Figure 1 Characterization of cell type-specific RNA and DNA binding proteins and HydRA prediction and validation of ARID5A. a.** Table of number and examples of cell type RBPs. * indicates known cell markers but not previously described as DNA or RNA binding proteins. **b.** Dot plot of Macrophage/Microglial RBPs with annotated binding. **c.** Presence of Macrophage/Microglial RBPs in microglial transcriptomic signatures derived from published datasets (Olah et al. 2020, Silvlin et al. 2022, Millet et al. 2024) **d.** HydRA scoring of factors such as IDR and complexity to determine significant areas of predicted RNA binding for ARID5A. **e.** Immunoprecipitation of ARID5A in THP-1 macrophages treated with low and high concentrations of RNAse in crosslink and no crosslink conditions. A biotinylated cytidine is ligated and samples are then probed with anti-streptavidin as a readout of nucleic acid binding.

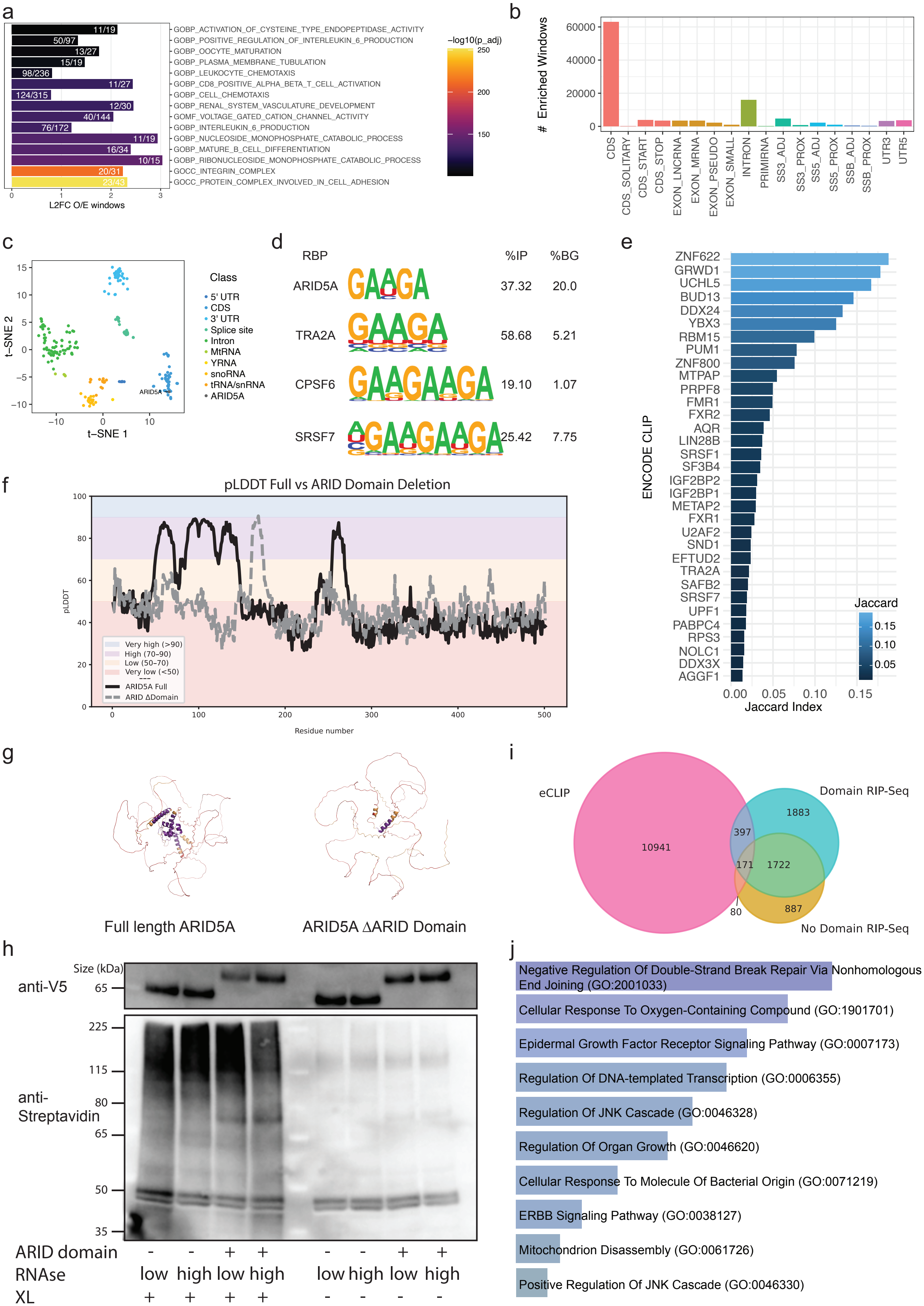

a
11/19
50/97
13/27
15/19
98/236
11/27
124/315
12/30
40/144
76/172
11/19
16/34
10/15
20/31
23/43
GOBP_ACTIVATION_OF_CYSTEINE_TYPE_ENDOPEPTIDASE_ACTIVITY
GOBP_POSITIVE_REGULATION_OF_INTERLEUKIN_6_PRODUCTION
GOBP_OOCYTE_MATURATION
GOBP_PLASMA_MEMBRANE_TUBULATION
GOBP_LEUKOCYTE_CHEMOTAXIS
GOBP_CD8_POSITIVE_ALPHA_BETA_T_CELL_ACTIVATION
GOBP_CELL_CHEMOTAXIS
GOBP_RENAL_SYSTEM_VASCULATURE_DEVELOPMENT
GOMF_VOLTAGE_GATED_CATION_CHANNEL_ACTIVITY
GOBP_INTERLEUKIN_6_PRODUCTION
GOBP_NUCLEOSIDE_MONOPHOSPHATE_CATABOLIC_PROCESS
GOBP_MATURE_B_CELL_DIFFERENTIATION
GOBP_RIBONUCLEOSIDE_MONOPHOSPHATE_CATABOLIC_PROCESS
GOCC_INTEGRIN_COMPLEX
GOCC_PROTEIN_COMPLEX_INVOLVED_IN_CELL_ADHESION
0 1 2 3
L2FC O/E windows
−log10(p_adj)
250
200
150
b
# Enriched Windows
60000
40000
20000
0
CDS
CDS_SOLITARY
CDS_START
CDS_STOP
EXON_LNCRNA
EXON_MRNA
EXON_PSEUDO
EXON_SMALL
INTRON
PRIMIRNA
SS3_ADJ
SS3_PROX
SS5_ADJ
SS5_PROX
SSB_ADJ
SSB_PROX
UTR3
UTR5
c
t-SNE 2
t-SNE 1
15
10
5
0
−5
−10
−10 0 10
ARID5A
Class
5' UTR
CDS
3' UTR
Splice site
Intron
MtRNA
YRNA
snoRNA
tRNA/snRNA
ARID5A
d
RBP
%IP
%BG
ARID5A
GAAGA
37.32
20.0
TRA2A
GAAGA
58.68
5.21
CPSF6
GAAGAAGA
19.10
1.07
SRSF7
AGAAGAAGA
25.42
7.75
e
ZNF622
GRWD1
UCHL5
BUD13
DDX24
YBX3
RBM15
PUM1
ZNF800
MTPAP
PRPF8
FMR1
FXR2
AQR
LIN28B
SRSF1
SF3B4
IGF2BP2
IGF2BP1
METAP2
FXR1
U2AF2
SND1
EFTUD2
TRA2A
SAFB2
SRSF7
UPF1
PABPC4
RPS3
NOLC1
DDX3X
AGGF1
ENCODE CLIP
Jaccard
0.15
0.10
0.05
0.00 0.05 0.10 0.15
Jaccard Index
f
pLDDT Full vs ARID Domain Deletion
pLDDT
100
80
60
40
20
0
0 100 200 300 400 500
Residue number
Very high (>90)
High (70–90)
Low (50–70)
Very low (<50)
ARID5A Full
ARID ΔDomain
g
Full length ARID5A
ARID5A ΔARID Domain
h
Size (kDa)
anti-V5
65
225
115
80
65
50
35
anti-
Streptavidin
ARID domain - - + + - - + +
RNAse low high low high low high low high
XL + + + + - - - -
i
eCLIP
Domain RIP-Seq
No Domain RIP-Seq
10941
1883
397
171
1722
80
887
j
Negative Regulation Of Double-Strand Break Repair Via Nonhomologous End Joining (GO:2001033)
Cellular Response To Oxygen-Containing Compound (GO:1901701)
Epidermal Growth Factor Receptor Signaling Pathway (GO:0007173)
Regulation Of DNA-templated Transcription (GO:0006355)
Regulation Of JNK Cascade (GO:0046328)
Regulation Of Organ Growth (GO:0046620)
Cellular Response To Molecule Of Bacterial Origin (GO:0071219)
ERBB Signaling Pathway (GO:0038127)
Mitochondrion Disassembly (GO:0061726)
Positive Regulation Of JNK Cascade (GO:0046330)

**Supplementary Figure 2 ARID5A binds the CDS and purine rich motifs; and binds RNA at both the ARID domain and IDR.** **a.** Barplot of gene sets enriched in ARID5A eCLIP over ENCODE eCLIP datasets**. b.** Barplot showing number of enriched reproducible windows across different transcript regions derived from ARID5A eCLIP. **c.** TSNE plot of ARID5A and ENCODE RBPs clustered by preferred transcript binding regions. **d.** Motif enrichment calculated by Homer depicting purine rich motif binding by ARID5A compared to ENCODE RBPs **e.** Barplot of Jaccard indexes indicating degree of overlap between ARID5A and ENCODE RBPs at the gene level. **f.** Alphafold2 predicted Local Distance Difference Test (pLDDT) scores by residue for full length ARID5A protein (black) and ARID5A protein without the ARID domain (gray). Scores indicate confidence as very low (red, <50) low (orange, 50-70), high (purple, 70-90), or very high (blue, >90). **g.** Alphafold2 predicted structures for full length ARID5A and ARID-domain deleted ARID5A. Structures are colored by pLDDT scores as indicated in panel f. **h.** Immunoprecipitation of ARID5A-V5 in low and high concentrations of RNAse in crosslink and no crosslink conditions of HEK293T cells transfected with either constructs containing full length ARID5A or ARID domain deletion. A biotinylated cytidine is ligated and samples are then probed with anti-streptavidin as a readout of nucleic acid binding. **i.** Venn diagram depicting overlap of THP-1 macrophage eCLIP with RIP-seq performed on THP-1 macrophages nucleofected with either full length ARID5A (Domain) or ARID domain deleted ARID5A (No Domain). **j.** Pathway enrichment of GO biological terms for transcripts detected in the Domain RIP-seq and eCLIP, but not in the No Domain RIP-seq. Significance determined by Enrichr.

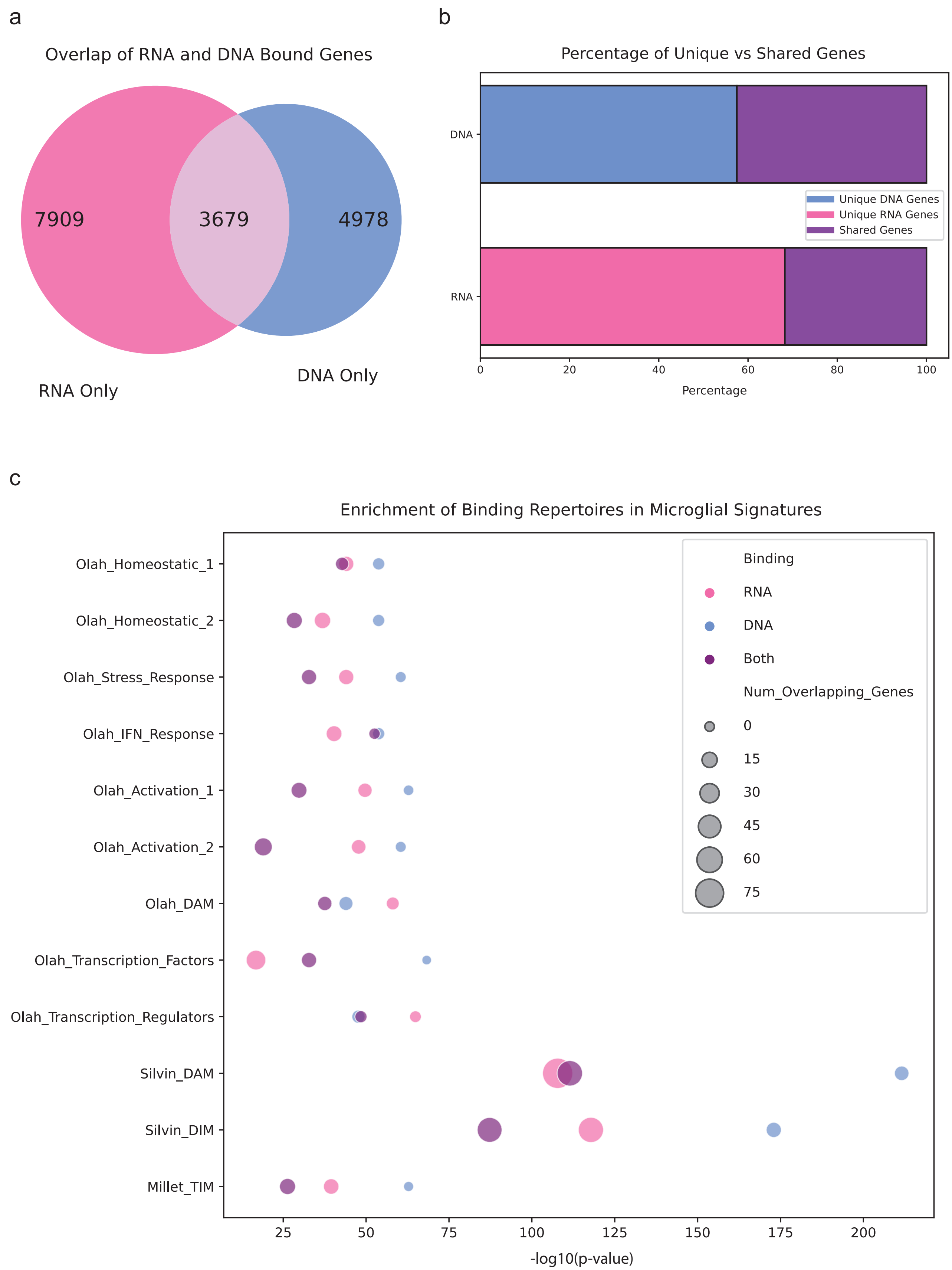


**Supplementary Figure 3 ARID5A DNA vs RNA bound transcripts. a.** Venn diagram depicting overlap of genes containing an RNA binding and a DNA binding site Jaccard index =0.1623. **b.** Barplot of percentage of unique vs shared genes for DNA (blue) and RNA (pink) binding repertoires. **c.** Bubble plot depicting -log10(p-value) enrichment in microglial signatures for DNA bound genes (blue), RNA bound genes (pink), and DNA/RNA bound genes (purple). Number of overlapping genes is represented by dot size. Significance determined by Fisher's exact test.

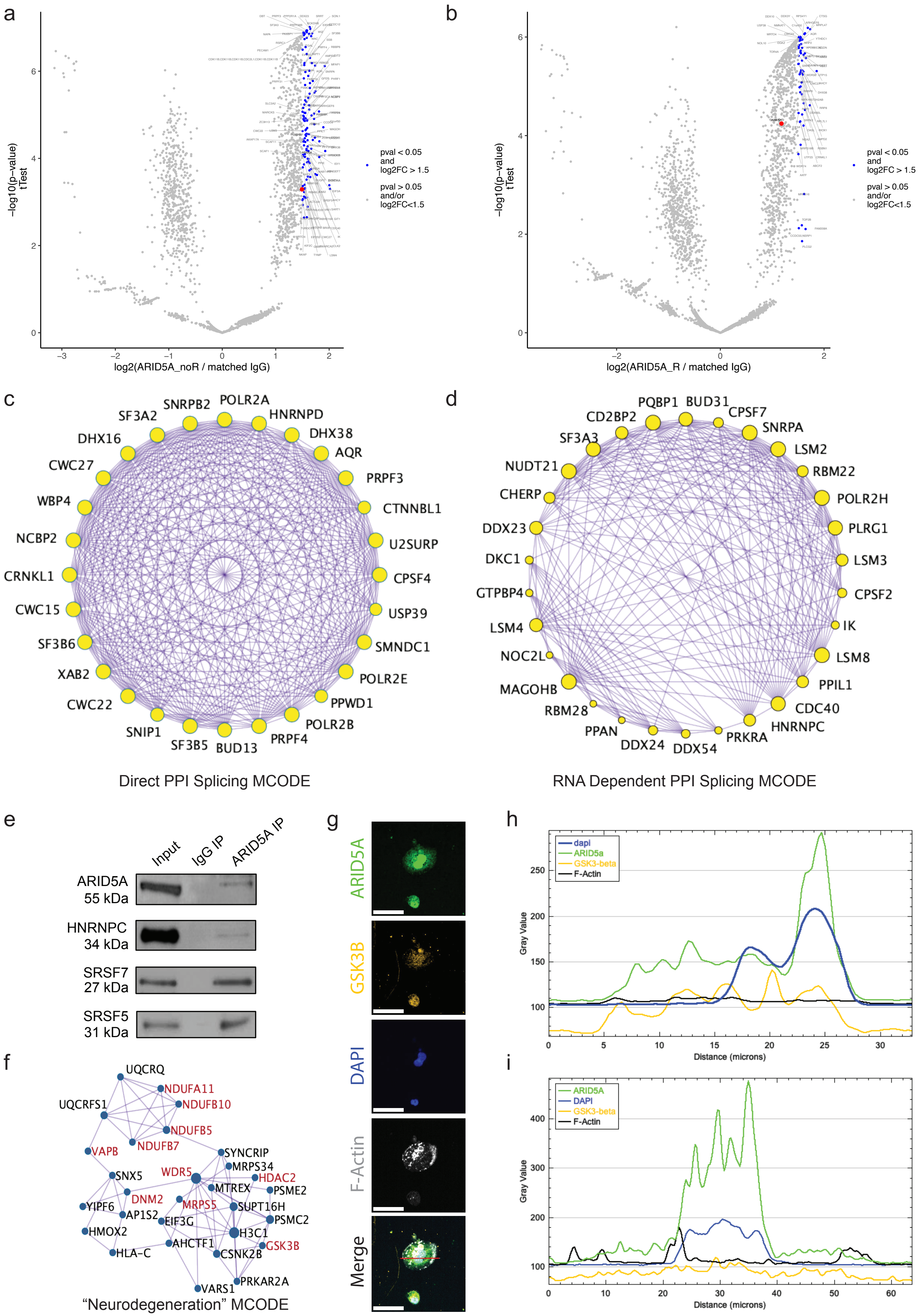
a
−log10(p−value) tTest
log2(ARID5A_noR / matched IgG)
pval < 0.05 and log2FC > 1.5
pval > 0.05 and/or log2FC<1.5
b
−log10(p−value) tTest
log2(ARID5A_R / matched IgG)
pval < 0.05 and log2FC > 1.5
pval > 0.05 and/or log2FC<1.5
c
SNRPB2
POLR2A
HNRNPD
SF3A2
DHX38
DHX16
AQR
CWC27
PRPF3
WBP4
CTNNBL1
NCBP2
U2SURP
CRNKL1
CPSF4
CWC15
USP39
SF3B6
SMNDC1
XAB2
POLR2E
CWC22
PPWD1
SNIP1
POLR2B
SF3B5
BUD13
PRPF4
Direct PPI Splicing MCODE
d
PQBP1
BUD31
CD2BP2
CPSF7
SNRPA
SF3A3
LSM2
NUDT21
RBM22
CHERP
POLR2H
DDX23
PLRG1
DKC1
LSM3
GTPBP4
CPSF2
LSM4
IK
NOC2L
LSM8
MAGOHB
PPIL1
RBM28
CDC40
PPAN
HNRNPC
DDX24
PRKRA
DDX54
RNA Dependent PPI Splicing MCODE
e
Input
IgG IP
ARID5A IP
ARID5A 55 kDa
HNRNPC 34 kDa
SRSF7 27 kDa
SRSF5 31 kDa
f
UQCRQ
NDUFA11
NDUFB10
UQCRFS1
NDUFB5
NDUFB7
VAPB
SYNCRIP
MRPS34
SNX5
WDR5
HDAC2
MTREX
PSME2
DNM2
YIPF6
MRPS5
SUPT16H
AP1S2
EIF3G
PSMC2
HMOX2
H3C1
AHCTF1
GSK3B
HLA-C
CSNK2B
PRKAR2A
VARS1
“Neurodegeneration” MCODE
g
ARID5A
GSK3B
DAPI
F-Actin
Merge
h
dapi
ARID5a
GSK3-beta
F-Actin
Gray Value
Distance (microns)
i
ARID5A
DAPI
GSK3-beta
F-Actin
Gray Value
Distance (microns)

**Supplementary Figure 4 ARID5A interacts with splicing and neurodegeneration associated proteins in the nucleus and cytoplasm. a.** Volcano plot of log2FC enrichment of ARID5A bound proteins normalized to IgG control in the absence of RNAse treatment. Significant interactions are represented in blue with ARID5A depicted as the red dot. **b.** Volcano plot of log2FC enrichment of ARID5A bound proteins normalized to IgG control in the presence of RNAse treatment. Significant interactions are represented in blue with ARID5A depicted as the red dot. **c.** "Splicing" MCODE cluster of interactions enriched in the direct ARID5A-PPI network. MCODE clustering and significance performed by Metascape. **d.** "Splicing" MCODE cluster of interactions enriched in the RNA-dependent ARID5A-PPI network. MCODE clustering and significance performed by Metascape. **e.** Co-immunoprecipitation of ARID5A or IgG control validating PPI splicing proteins. **f.** "Neurodegeneration" MCODE cluster of interactions enriched in the RNA-dependent ARID5A-PPI network. AD associated proteins are highlighted in red. MCODE clustering and significance performed by Metascape. **g.** Immunofluorescence staining of ARID5A (green) and GSK3β (gold) in THP-1 macrophages (20x, scale 50 μm). DAPI (blue) and F-actin (gray) were used to stain the nucleus and cytoskeleton respectively. Red lines are drawn through the merge to show ROI measured. **h.** Quantification of fluorescent intensities across each channel for the cell on the left shown in the merge composite. **i.** Quantification of fluorescent intensities across each channel for the cell on the right in the merge composite.

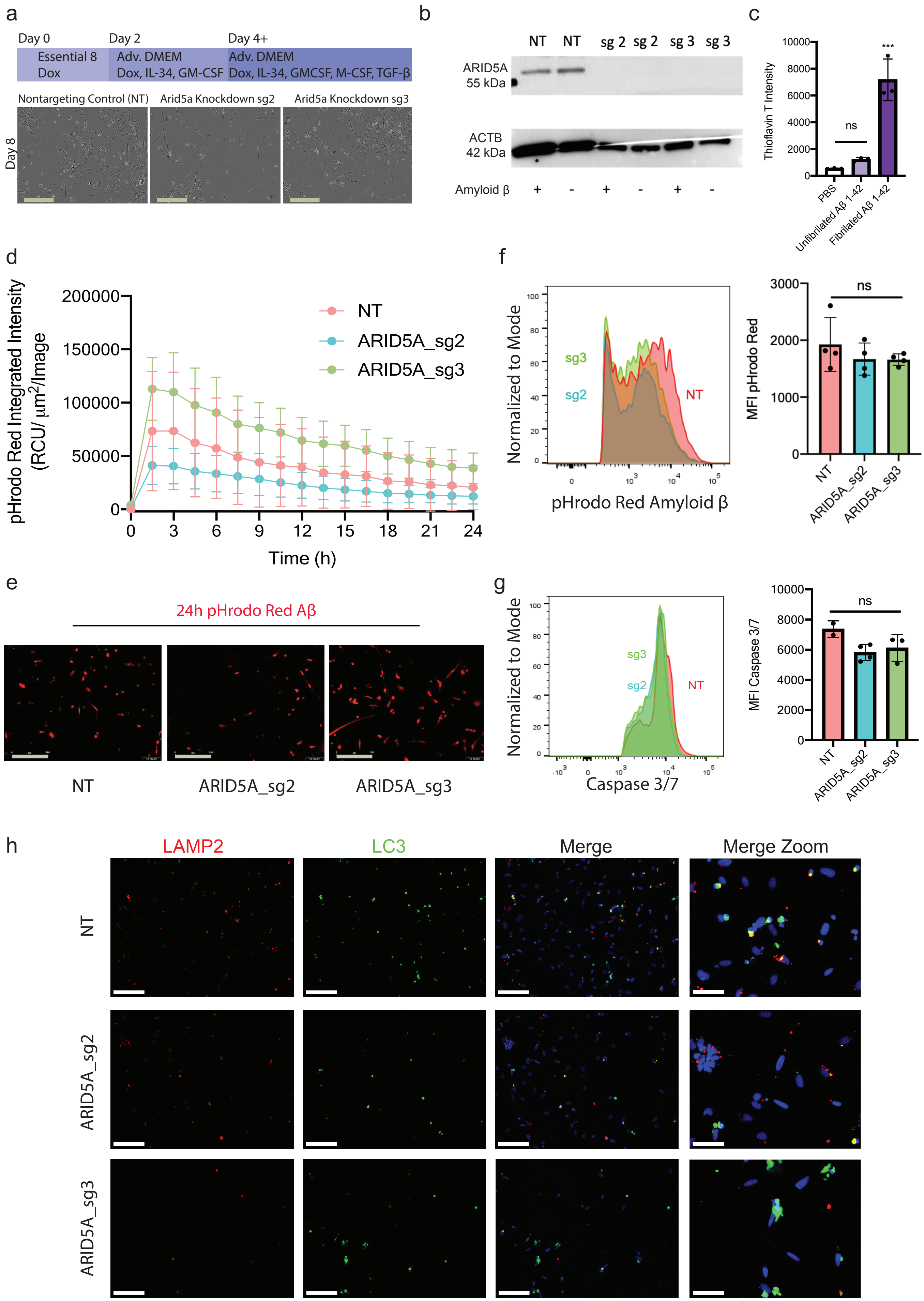
a
Day 0
Day 2
Day 4+
Essential 8
Dox
Adv. DMEM
Dox, IL-34, GM-CSF
Adv. DMEM
Dox, IL-34, GMCSF, M-CSF, TGF-β
Nontargeting Control (NT)
Arid5a Knockdown sg2
Arid5a Knockdown sg3
Day 8
b
NT NT sg 2 sg 2 sg 3 sg 3
ARID5A
55 kDa
ACTB
42 kDa
Amyloid β + - + - + -
c
Thioflavin T Intensity
ns
***
PBS
Unfibrilated Aβ 1-42
Fibrilated Aβ 1-42
d
pHrodo Red Integrated Intensity
(RCU/ µm2/Image
NT
ARID5A_sg2
ARID5A_sg3
Time (h)
e
24h pHrodo Red Aβ
NT
ARID5A_sg2
ARID5A_sg3
f
Normalized to Mode
sg3
sg2
NT
pHrodo Red Amyloid β
MFI pHrodo Red
ns
NT
ARID5A_sg2
ARID5A_sg3
g
Normalized to Mode
sg3
sg2
NT
Caspase 3/7
MFI Caspase 3/7
ns
NT
ARID5A_sg2
ARID5A_sg3
h
LAMP2
LC3
Merge
Merge Zoom
NT
ARID5A_sg2
ARID5A_sg3

**Supplementary Figure 5 ARID5A knockdown cells maintain lysosomes and knockdown does not result in altered phagocytosis or apoptosis under Aβ treatment**. **a.** Schematic of iTF Microglia differentiation from iPSCs containing a doxycycline-inducible transcription factors system and CRISPRi machinery. Incucyte images (20x, scale 200 μm) of nontargeting control (NT), ARID5A-targeting CRISPRi guide 2 (sg2) and guide 3 (sg3) on Day 8. **b.** Western blot of ARID5A and β-actin in untreated and Aβ-treated conditions. **c.** Thioflavin T intensity validating Aβ aggregates represent fibrillated amyloid protein compared to PBS and unfibrilated control. Significance determined by one-way ANOVA with Tukey's test for multiple corrections, $p<0.001$, n=3 independent biological replicates. **d.** Timecourse of pHrodo red integrated intensity throughout treatment with 24h of pHrodo-red labeled Aβ. **e.** Incucyte images (20x, scale 100 μm) of red fluorescence depicting phagocytosis of pHrodo-red labeled Aβ at 24 hours. **f.** Median fluorescence intensity (MFI) of pHrodo red in microglia quantified by flow cytometry. Significance measured by one-way ANOVA with Tukey's test for multiple corrections, $p<0.05$, n=4 independent biological replicates. **g.** MFI of Caspase 3/7 in microglia following Aβ treatment (24h) quantified by flow cytometry. Significance measured by one-way ANOVA with Tukey's test for multiple corrections, $p<0.05$, n=3 independent biological replicates. **h.** Immunofluorescence staining for lysosomal marker LAMP1 (red) and autophagy marker LC3 (green) following 24h Aβ treatment (20x, scale 200 and 75 μm). Data provided as a Source Data file.

a Exon 2 Skipped ENST00000327247.9, 536 a.a

P R A A F L A A A G G R A A S C L Q S L T A R G P S L Q S R P G A F A G C G G I S
F C L R L G L L F L F A D V D P L S F R D S G T P V V F S S S N D P E G Met E F S
S P S R E E C P K P L S R V S I Met A G S L T G L L L L Q A V S W A S G A R P C I P
K S F G Y S S V V C V C N A T Y C D S F D P P T F P A L G T F S R Y E S T R S G R
R Met E L S Met G P I Q A N H T G T G L L L T L Q P E Q K F Q K V K G F G G A Met
T D A A A L N I L A L S P P A Q N L L L K S Y F S E E G I G Y N I I R V P Met A S C D
F S I R T Y T Y A D T P D D F Q L H N F S L P E E D T K L K I P L I H R A L Q L A Q
R P V S L L A S P W T S P T W L K T N G A V N G K G S L K G Q P G D I Y H Q T W
A R Y F V K F L D A Y A E H K L Q F W A V T A E N E P S A G L L S G Y P F Q C L G
F T P E H Q R D F I A R D L G P T L A N S T H H N V R L L Met L D D Q R L L L P H
W A K V V L T D P E A A K Y V H G I A V H W Y L D F L A P A K A T L G E T H R L F
P N T Met L F A S E A C V G S K F W E Q S V R L G S W D R G Met Q Y S H S I I T N
L L Y H V V G W T D W N L A L N P E G G P N W V R N F V D S P I I V D I T K D T F
Y K Q P Met F Y H L G H F S K F I P E G S Q R V G L V A S Q K N D L D A V A L Met
H P D G S A V V V V L N R S S K D V P L T I K D P A V G F L E T I S P G Y S I H T Y
L W R R Q Stop W S R Y S R R H W A Q P G H Stop R D R V S S H A V C D Stop R
G H S R A S V S L Q R R K P R G N G L G D S L S P L G G A R G W R P L E K D Q
Stop A P V S P Q P P C L C E H A L C A A C F G N W A W V Q A Stop G E L T V R T
N T R S G L R V R K R R D Stop E S W A Q N W R L F V F P G D A E L G P W S S S
V S I R A E A L K Q Q R V C P G T Q Met I P Met A P A R K N G S S Stop R R K C L S P

b Exon 2 Inclusion ENST00000493842.5, 227 a.a

G L H P A F R V L L R G A P V S S P A Q A P L Q A A V G F R F A S G W G C C F S
S P T Stop R R G F T Met L V R L V S N S Stop P R D P P A S A C Q T A V I I G V S
H R A R P T I I I I F N V D P L S F R D S G T P V V F S S S N D P E G Met E F S S P
S R E E C P K P L S R V S I Met A G S L T G L L L L Q A V S W A S G A R P C I P K S
F G Y S S V V C V C N A T Y C D S F D P P T F P A L G T F S R Y E S T R S G R R
Met E L S Met G P I Q A N H T G T G L L L T L Q P E Q K F Q K V K G F G G A Met T
D A A A L N I L A L S P P A Q N L L L K S Y F S E E G I G Y N I I R V P Met A S C D F
S I R T Y T Y A D T P D D F Q L H N F S L P E E D T K L K I P L I H R A L Q L A Q R
P V S L L A S P W T S P T W L K T N

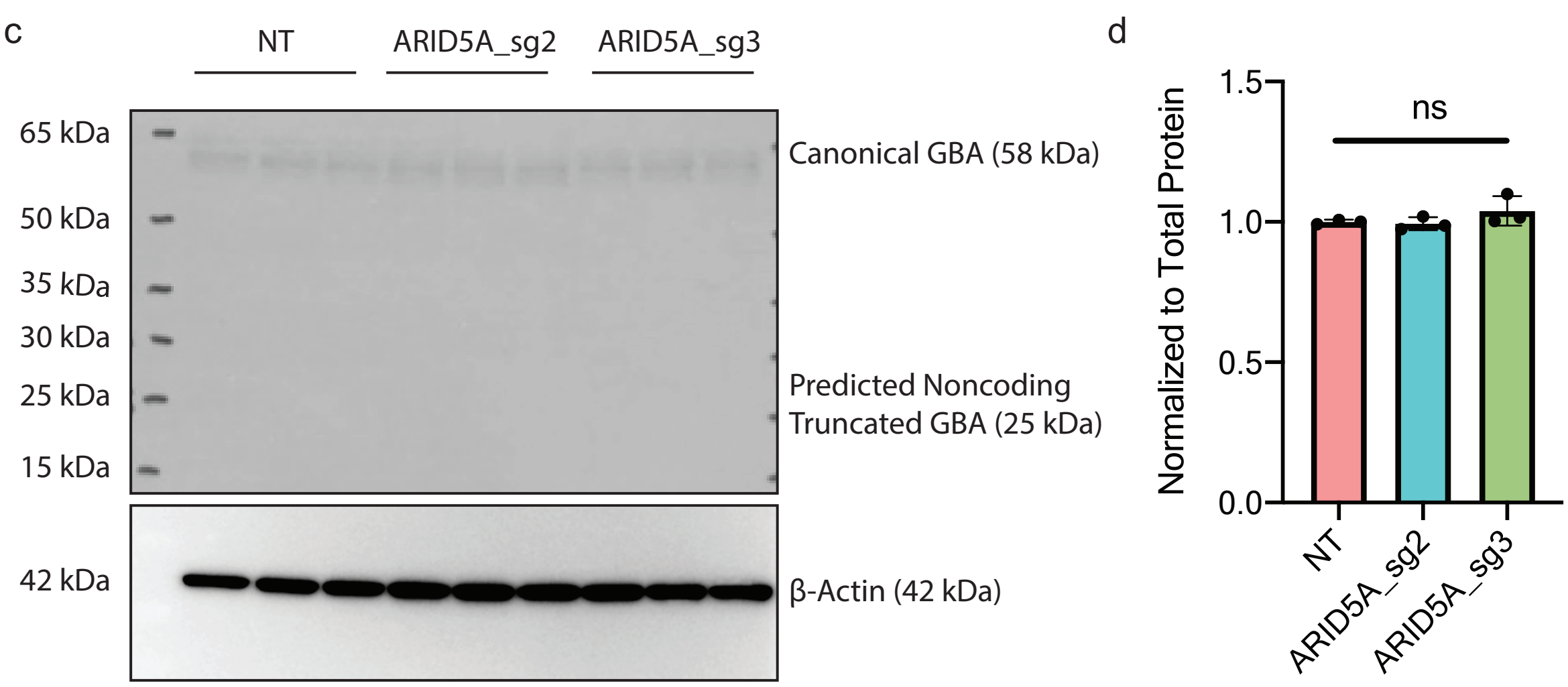


**Supplementary Figure 6 Knockdown of ARID5A results in two different protein sequences for GBA. a.** ExPASy predicted protein sequence based on canonical transcript for *GBA* in which exon 2 is skipped. The TIM barrel domain is comprised of 8 helices (blue) and beta sandwich (pink) and normally generates a 536 a.a. long protein that is stable. Stop codons are in red. **b.** ExPASy predicted protein sequence based on exon 2 inclusion, resulting in a truncated (227 a.a.) and unstable protein lacking key structural features. A premature stop codon is also present in exon 2 inclusion, upstream of the truncated protein. **c.** Western blot for GBA and β-actin in WT and ARID5A knockdown iTF microglia. Canonical GBA detected at 58 kDa, predicted noncoding truncated variant not detected at 25 kDa. **d.** Barplot depicting quantification of canonical GBA normalized to total protein. Significance measured by one-way ANOVA with Tukey's test for multiple corrections.

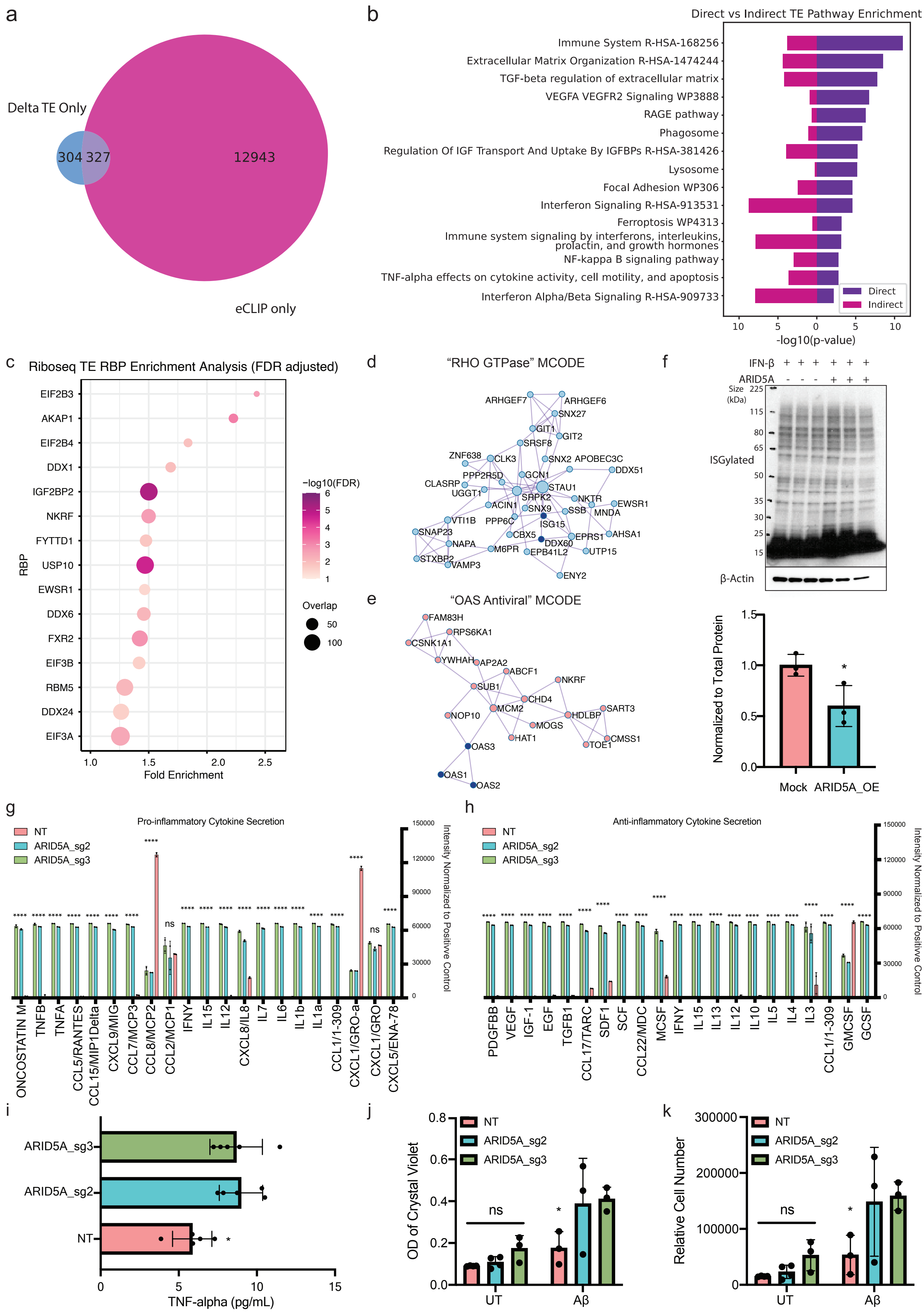
a
Delta TE Only
304
327
12943
eCLIP only
b
Direct vs Indirect TE Pathway Enrichment
Immune System R-HSA-168256
Extracellular Matrix Organization R-HSA-1474244
TGF-beta regulation of extracellular matrix
VEGFA VEGFR2 Signaling WP3888
RAGE pathway
Phagosome
Regulation Of IGF Transport And Uptake By IGFBPs R-HSA-381426
Lysosome
Focal Adhesion WP306
Interferon Signaling R-HSA-913531
Ferroptosis WP4313
Immune system signaling by interferons, interleukins, prolactin, and growth hormones
NF-kappa B signaling pathway
TNF-alpha effects on cytokine activity, cell motility, and apoptosis
Interferon Alpha/Beta Signaling R-HSA-909733
Direct
Indirect
-log10(p-value)
c
Riboseq TE RBP Enrichment Analysis (FDR adjusted)
EIF2B3
AKAP1
EIF2B4
DDX1
IGF2BP2
NKRF
FYTTD1
USP10
EWSR1
DDX6
FXR2
EIF3B
RBM5
DDX24
EIF3A
RBP
Fold Enrichment
−log10(FDR)
Overlap
d
"RHO GTPase" MCODE
ARHGEF7
ARHGEF6
SNX27
GIT1
GIT2
SRSF8
ZNF638
CLK3
SNX2
APOBEC3C
DDX51
PPP2R5D
GCN1
CLASRP
UGGT1
STAU1
SRPK2
NKTR
EWSR1
ACIN1
SNX9
SSB
MNDA
VTI1B
PPP6C
ISG15
SNAP23
CBX5
EPRS1
AHSA1
NAPA
DDX60
M6PR
EPB41L2
UTP15
STXBP2
VAMP3
ENY2
e
"OAS Antiviral" MCODE
FAM83H
RPS6KA1
CSNK1A1
YWHAH
AP2A2
ABCF1
NKRF
SUB1
CHD4
MCM2
SART3
NOP10
HDLBP
MOGS
HAT1
CMSS1
TOE1
OAS3
OAS1
OAS2
f
IFN-β
ARID5A
Size (kDa)
ISGylated
β-Actin
Normalized to Total Protein
Mock
ARID5A_OE
g
Pro-inflammatory Cytokine Secretion
NT
ARID5A_sg2
ARID5A_sg3
Intensity Normalized to Positive Control
ONCOSTATIN M
TNFB
TNFA
CCL5/RANTES
CCL15/MIP1Delta
CXCL9/MIG
CCL7/MCP3
CCL8/MCP2
CCL2/MCP1
IFNY
IL15
IL12
CXCL8/IL8
IL7
IL6
IL1b
IL1a
CCL1/1-309
CXCL1/GRO-a
CXCL1/GRO
CXCL5/ENA-78
h
Anti-inflammatory Cytokine Secretion
NT
ARID5A_sg2
ARID5A_sg3
Intensity Normalized to Positive Control
PDGFBB
VEGF
IGF-1
EGF
TGFB1
CCL17/TARC
SDF1
SCF
CCL22/MDC
MCSF
IFNY
IL15
IL13
IL12
IL10
IL5
IL4
IL3
CCL1/1-309
GMCSF
GCSF
i
ARID5A_sg3
ARID5A_sg2
NT
TNF-alpha (pg/mL)
j
NT
ARID5A_sg2
ARID5A_sg3
OD of Crystal Violet
ns
UT
Aβ
k
NT
ARID5A_sg2
ARID5A_sg3
Relative Cell Number
ns
UT
Aβ

**Supplementary Figure 7 ARID5A regulates translational efficiency of transcripts through direct and indirect mechanisms to regulate cytokine secretion and cell migration. a.** Venn diagram depicting overlap of genes bound by ARID5A at the RNA level and differentially translated transcripts. Jaccard index= 0.0241. **b.** Barplot of pathway enrichment for differentially translated transcripts bound by ARID5A at the 5'UTR/CDS (Direct, purple) vs unbound (Indirect, pink) as represented by -log10(p-value). Significance calculated by Enrichr. **c.** Bubble plot depicting the enrichment of targets with upregulated TE upon ARID5A knockdown bound by RBPs that interact with ARID5A at the protein level. Binding targets were derived from ENCODE and significance was calculated by Fisher's exact test. **d.** MCODE "RHO GTPase" cluster generated by Metascape containing defense response proteins that showed enhanced translation upon ARID5A knockdown (shown in navy). **e.** MCODE "OAS Antiviral" cluster generated by Metascape containing defense response proteins that showed enhanced translation in knockdown (shown in navy). **f.** Western blot probing for ISG15 and β-actin in HeLa cells treated for 24 hours with IFN-β with or without ARID5A overexpression and quantification. Significance determined by unpaired two-tailed t test, $p<0.05$, n=3 independent biological replicates. **g.** Barplot showing intensity of pro-inflammatory cytokines secreted under 24h Aβ treatment normalized to the positive control in the cytokine array. Significance measured by two-way ANOVA with Tukey's test for multiple corrections, $p<0.0001$. **h.** Barplot showing intensity of anti-inflammatory cytokines secreted under 24h Aβ treatment normalized to the positive control in the cytokine array. Significance measured by two-way ANOVA with Tukey's test for multiple corrections, $p<0.0001$, n=2 independent biological replicates. **i.** Validation of TNF-alpha secretion by ELISA. Significance measured by one-way ANOVA with Tukey's test for multiple corrections, $p<0.05$, n=5 independent biological replicates. Barplots depicting the optical density (OD) of crystal violet (**j**) derived from cells that successfully migrated the transwell insert. Cell number (**k**) was calculated using a standard curve of cell densities and OD values. Significance measured by two-way ANOVA with Tukey's test for multiple corrections, $p<0.05$, n=3 independent biological replicates. Data provided as a Source Data file.

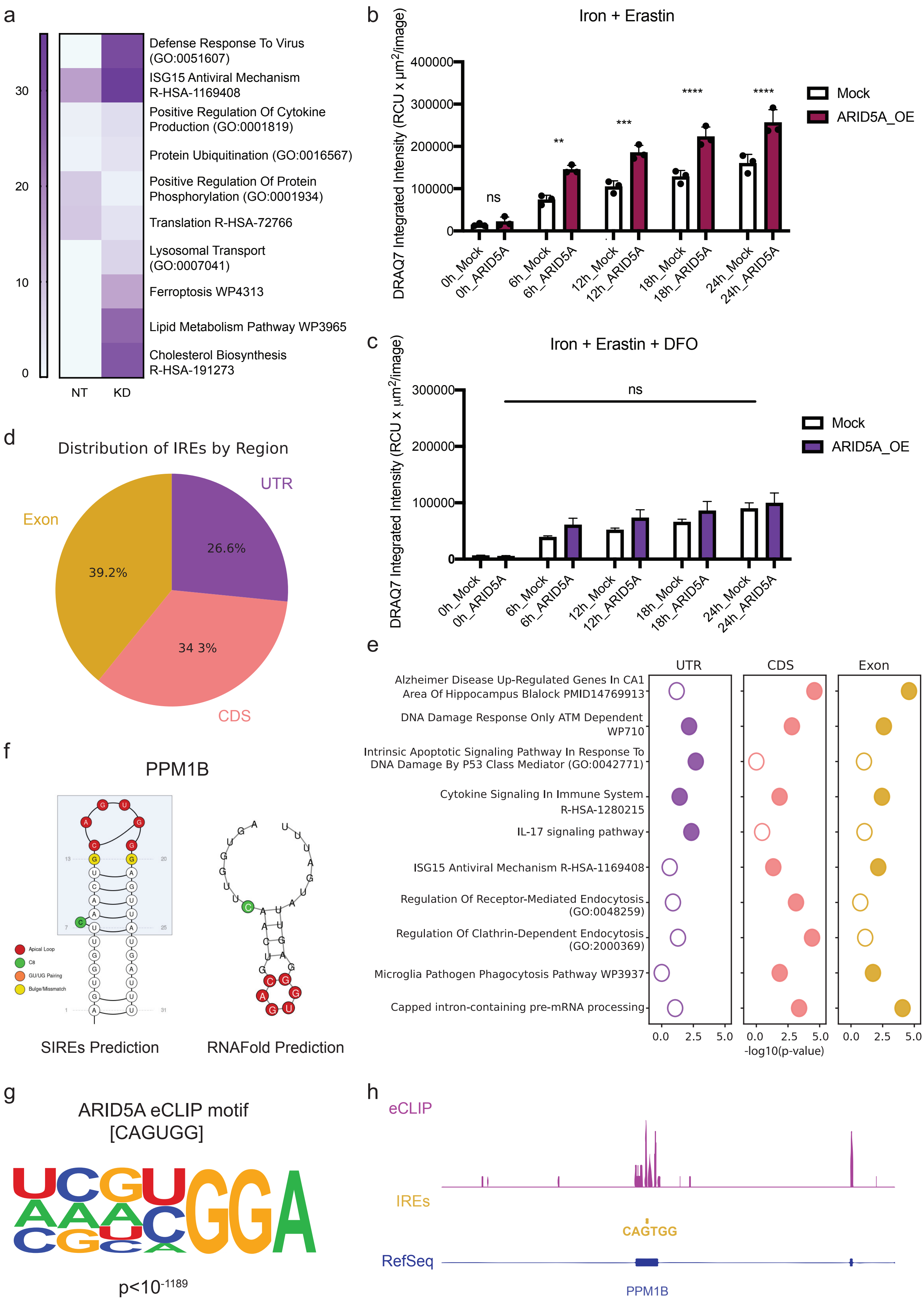
a
Defense Response To Virus (GO:0051607)
ISG15 Antiviral Mechanism R-HSA-1169408
Positive Regulation Of Cytokine Production (GO:0001819)
Protein Ubiquitination (GO:0016567)
Positive Regulation Of Protein Phosphorylation (GO:0001934)
Translation R-HSA-72766
Lysosomal Transport (GO:0007041)
Ferroptosis WP4313
Lipid Metabolism Pathway WP3965
Cholesterol Biosynthesis R-HSA-191273
30
20
10
0
NT
KD
b
Iron + Erastin
DRAQ7 Integrated Intensity (RCU x µm²/image)
400000
300000
200000
100000
0
ns
**
***
****
****
Mock
ARID5A_OE
0h_Mock
0h_ARID5A
6h_Mock
6h_ARID5A
12h_Mock
12h_ARID5A
18h_Mock
18h_ARID5A
24h_Mock
24h_ARID5A
c
Iron + Erastin + DFO
DRAQ7 Integrated Intensity (RCU x µm²/image)
300000
200000
100000
0
ns
Mock
ARID5A_OE
0h_Mock
0h_ARID5A
6h_Mock
6h_ARID5A
12h_Mock
12h_ARID5A
18h_Mock
18h_ARID5A
24h_Mock
24h_ARID5A
d
Distribution of IREs by Region
UTR
Exon
CDS
26.6%
39.2%
34 3%
e
UTR
CDS
Exon
Alzheimer Disease Up-Regulated Genes In CA1 Area Of Hippocampus Blalock PMID14769913
DNA Damage Response Only ATM Dependent WP710
Intrinsic Apoptotic Signaling Pathway In Response To DNA Damage By P53 Class Mediator (GO:0042771)
Cytokine Signaling In Immune System R-HSA-1280215
IL-17 signaling pathway
ISG15 Antiviral Mechanism R-HSA-1169408
Regulation Of Receptor-Mediated Endocytosis (GO:0048259)
Regulation Of Clathrin-Dependent Endocytosis (GO:2000369)
Microglia Pathogen Phagocytosis Pathway WP3937
Capped intron-containing pre-mRNA processing
0.0 2.5 5.0
-log10(p-value)
f
PPM1B
Apical Loop
C8
GU/UG Pairing
Bulge/Mismatch
SIREs Prediction
RNAFold Prediction
g
ARID5A eCLIP motif [CAGUGG]
p<10^-1189
h
eCLIP
IREs
CAGTGG
RefSeq
PPM1B

**Supplementary Figure 8 ISGylation and iron metabolism are interconnected pathways regulated by ARID5A at the RNA level. a.** Heatmap of pathway enrichment for translationally regulated ISGylation targets in ARID5A control (NT) and knockdown (KD) iTF microglia. Odds ratio and significance calculated by Enrichr. **b.** Timepoint quantification of integrated DRAQ7 intensity every six hours in HeLa cells transfected with an ARID5A overexpression vector and treated with iron and erastin. Significance measured by two-way ANOVA with Tukey's test for multiple corrections, $p<0.0001$, n=3 independent biological replicates. **c.** Timepoint quantification of integrated DRAQ7 intensity every six hours in HeLa cells transfected with an ARID5A overexpression vector and treated with iron, erastin, and DFO. Significance measured by two-way ANOVA with Tukey's test for multiple corrections, $p<0.05$, n=3 independent biological replicates. Data provided as a Source Data file. **d.** Distribution of iron response elements (IREs) found to overlap with ARID5A eCLIP binding sites across genomic regions. **e.** Pathway enrichment for overlapping binding/IRE sites based on genomic region. Significance calculated by Enrichr. **f.** SIREs and RNAfold prediction for overlapping IRE site in the *PPM1B* gene. **g.** Fifth most significant motif as determined by Homer in the ARID5A eCLIP. **h.** Genome browser tracks depicting coverage of ARID5A-bound transcript *PPM1B* (pink) and location of the overlapping IRE site (gold).

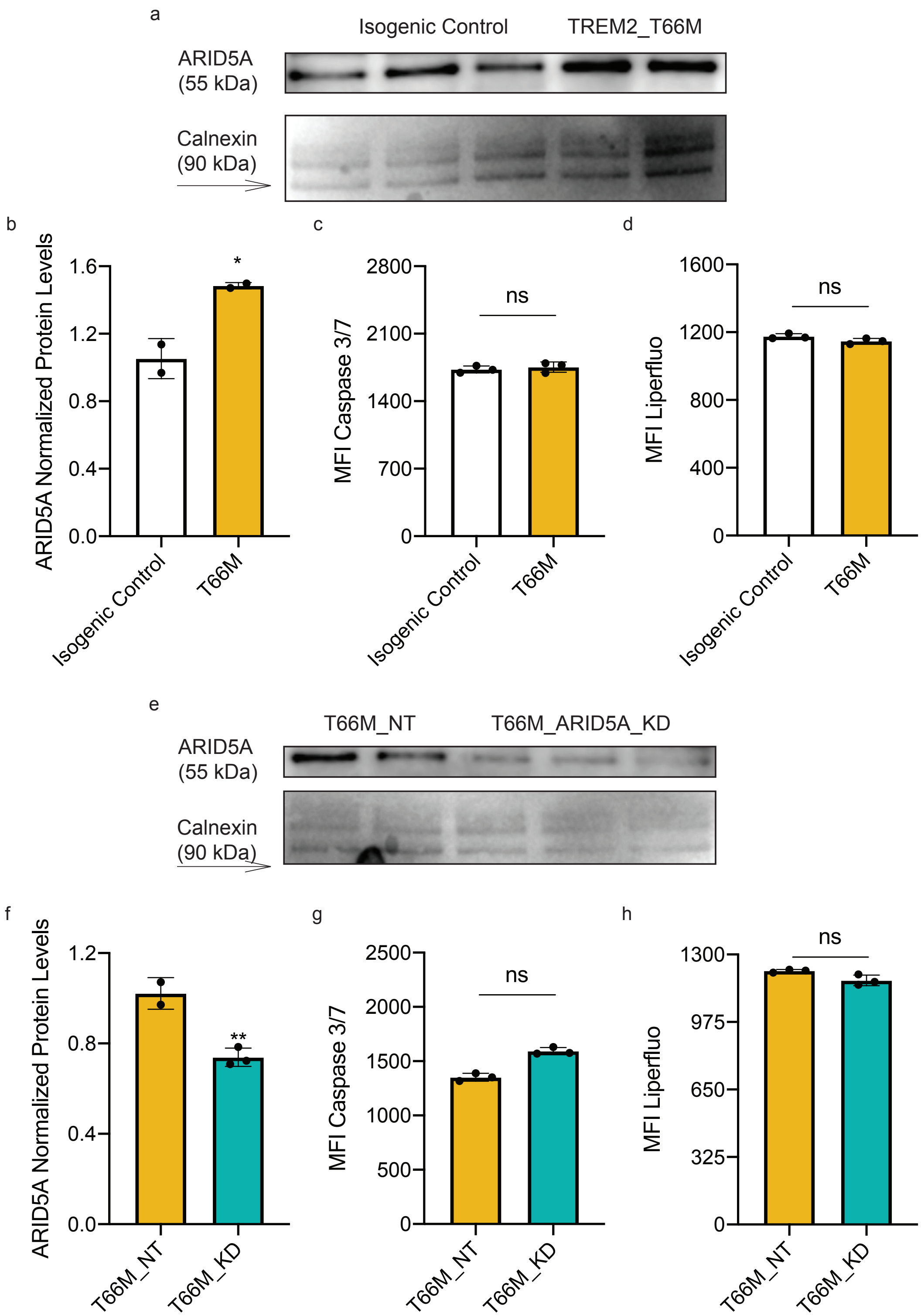


**Supplementary Figure 9. ARID5A is elevated in TREM2-T66M microglia, but knockdown does not alter apoptosis or lipid peroxidation in this model.**
**a.** Western blot of ARID5A in isogenic control and T66M mutant microglia. Calnexin is included as a loading control. **b.** Barplot of the quantification normalized to total protein, significance measured by unpaired t-test, $p<0.05$, n=2 independent biological replicates. **c.** Median fluorescence intensity (MFI) of Caspase 3/7 levels as measured by flow cytometry in isogenic and T66M mutant microglia. Significance measured by unpaired t-test, n=3 independent biological replicates. **d.** MFI of Liperfluo (lipid peroxidation) as measured by flow cytometry in isogenic vs T66M mutant microglia. Significance measured by unpaired t-test, n=3 independent biological replicates. **e.** Western blot of ARID5A in T66M mutant microglia treated with either a nontargeting (NT) shRNA control or an ARID5A-targeted shRNA (KD). Calnexin is included as a loading control. **f**. Barplot of the quantification normalized to total protein, significance measured by unpaired t-test, $p<0.01$, n=2 independent biological replicates. **g.** MFI of Caspase 3/7 levels as measured by flow cytometry in T66M microglia with or without ARID5A knockdown. Significance measured by unpaired t-test, n=3 independent biological replicates. **h.** MFI of Liperfluo (lipid peroxidation) as measured by flow cytometry in T66M microglia with or without

ARID5A knockdown. Significance measured by unpaired t-test, n=3 independent biological replicates. Data provided as a Source Data file.

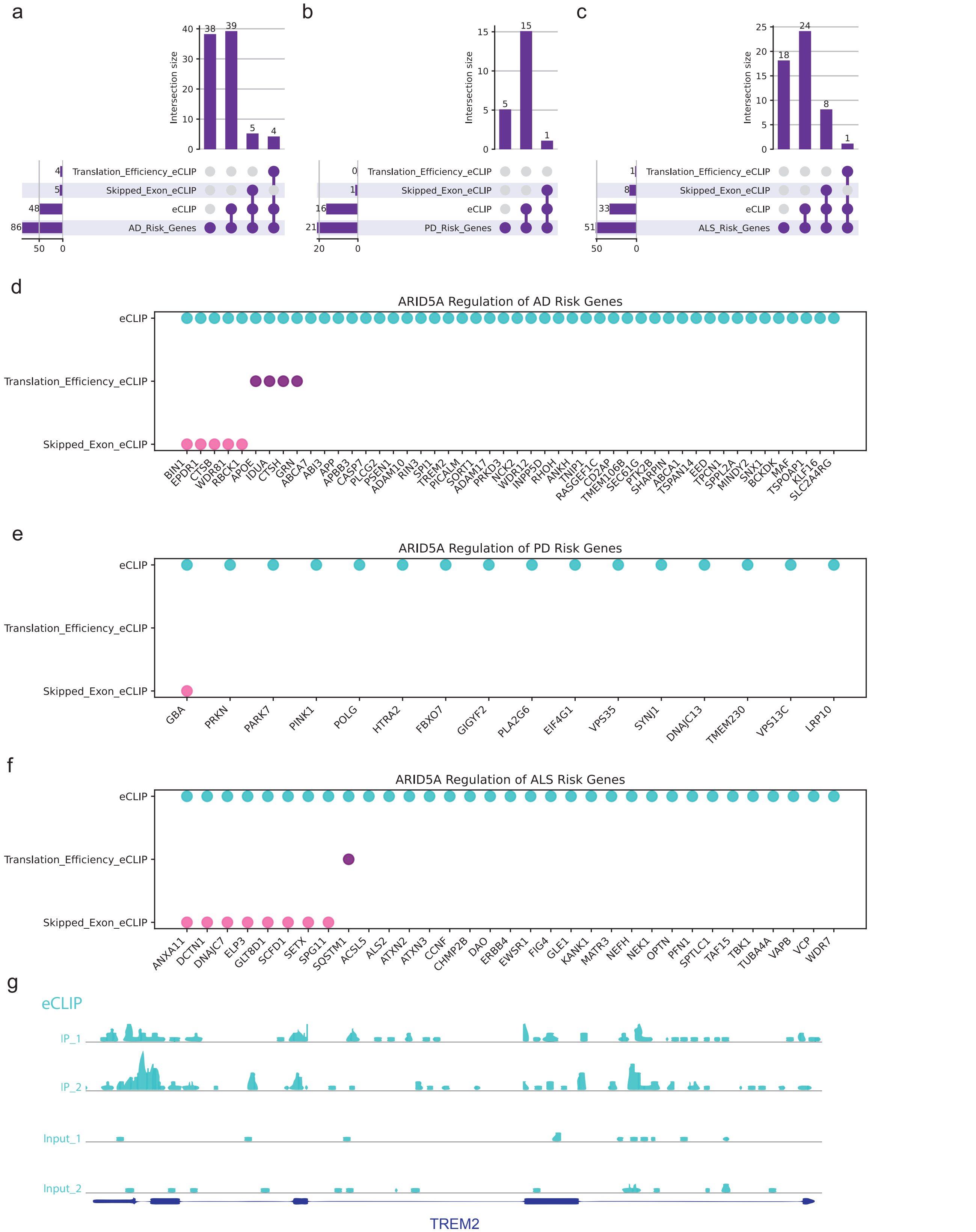


**Supplementary Figure 10 ARID5A RNA regulation of neurodegeneration risk genes**

UpSet plots depicting intersection of ARID5A-bound, differentially translated, differentially spliced transcripts with: **a.** AD risk genes, **b.** PD risk genes, **c.** ALS risk genes. Dot plots of bound transcripts (turquoise), differentially translated transcripts (purple), and

differentially spliced via exon skipping (pink) intersecting with: **d.** AD risk genes, **e.** PD risk genes, **f.** ALS risk genes. **g.** Genome browser tracks depicting coverage of ARID5A-bound transcript TREM2, an AD risk gene.

*Gating Strategy*

For all flow cytometry experiments, samples are gated sequentially as shown below. Small debris is removed with FSC-A/SSC-A (**a**), single cells are selected with FSC-H and SSC-H gates (**b, c**). Appropriate fluorescent channels (in this example PE-A) are then gated with the positive population containing less than 0.1% in the unstained control (**d**). These gates are uniformly applied across samples as shown in **e** (unstained control) and **f** (experimental example). Median fluorescence intensity is calculated with FlowJo.

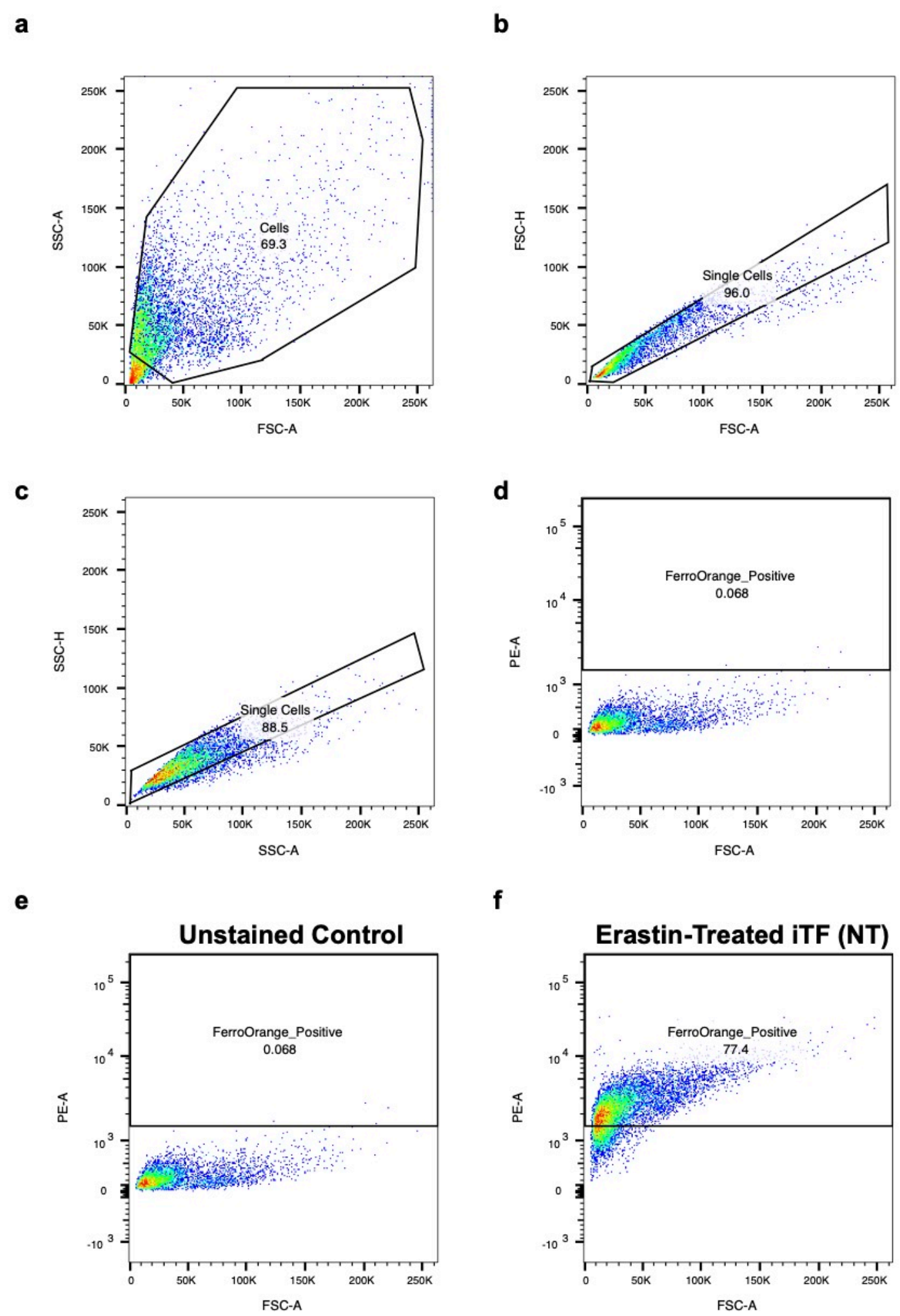

*Antibody Validation*

**Supplementary Validation of ARID5A IP Antibody Unprocessed Western Blot**

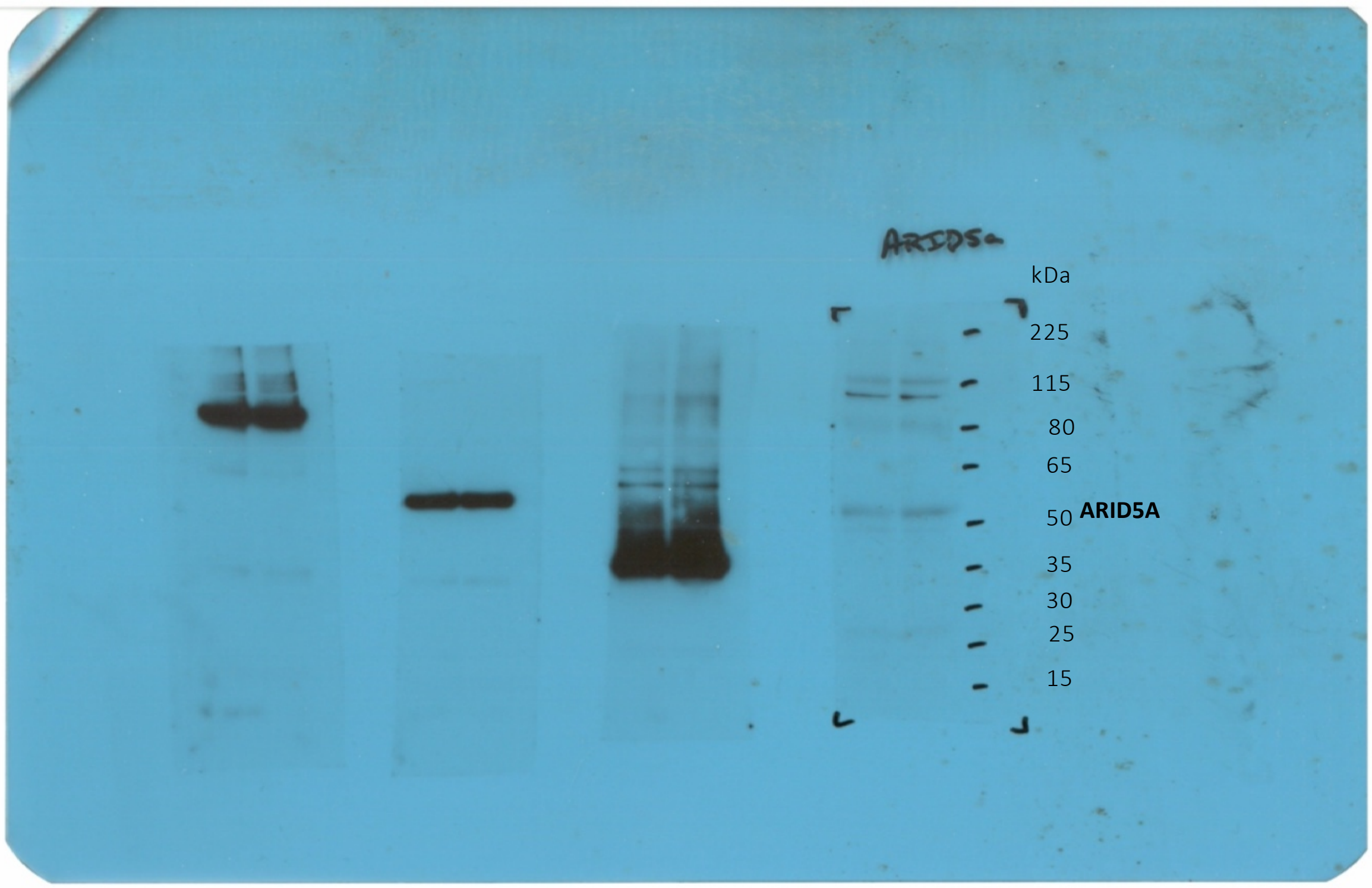

*Source Data Unprocessed Gels and Blots*

**Source Data Figure 1d Unprocessed Western Blot RBFOX3, GFAP, IBA1**

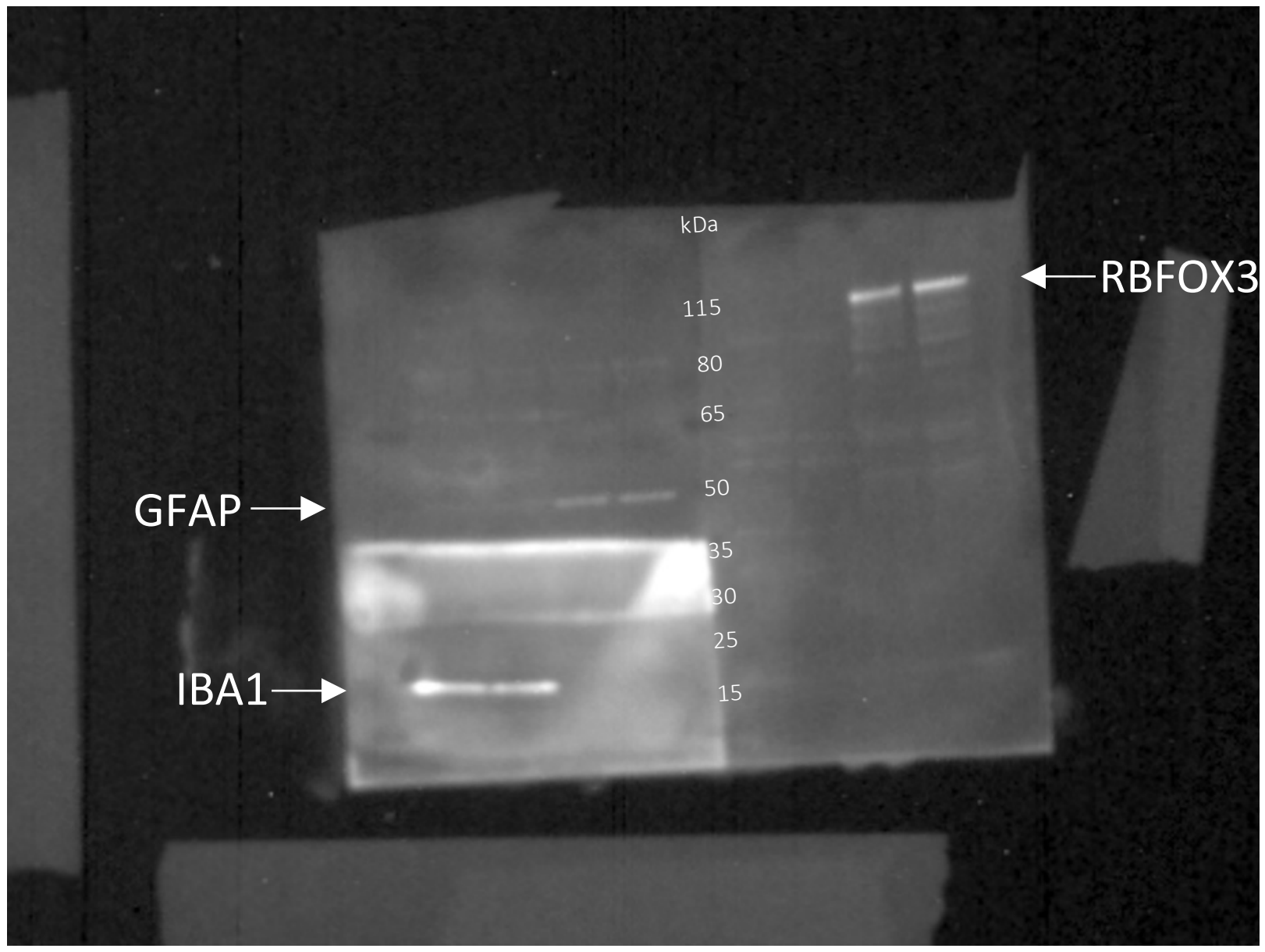


**Source Data Figure 1d Unprocessed Western Blot ARID5A**

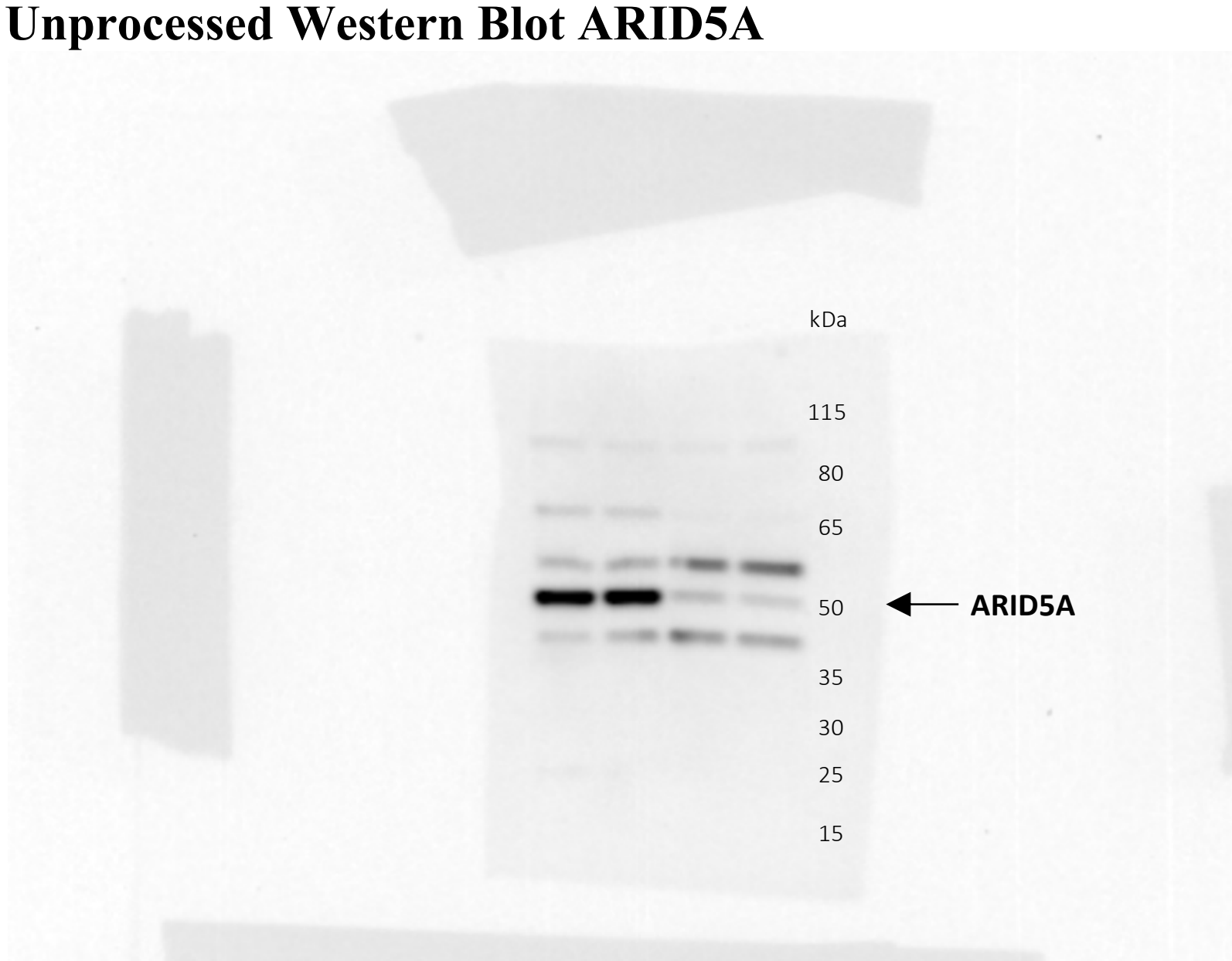


**Source Data Figure 1d Unprocessed Western Blot Beta Actin**

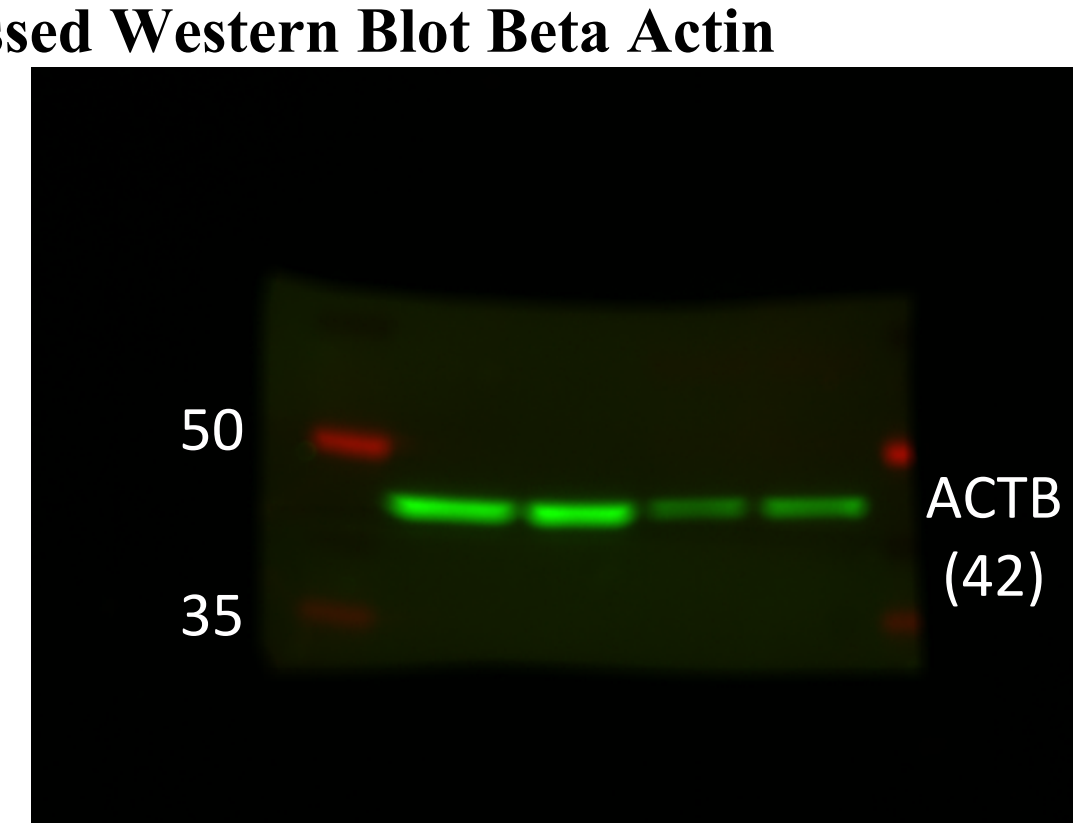

**Source Data Figure 2e Unprocessed Western Blot (SRRT, HNRNPC, SRSF5, SRSF7)**

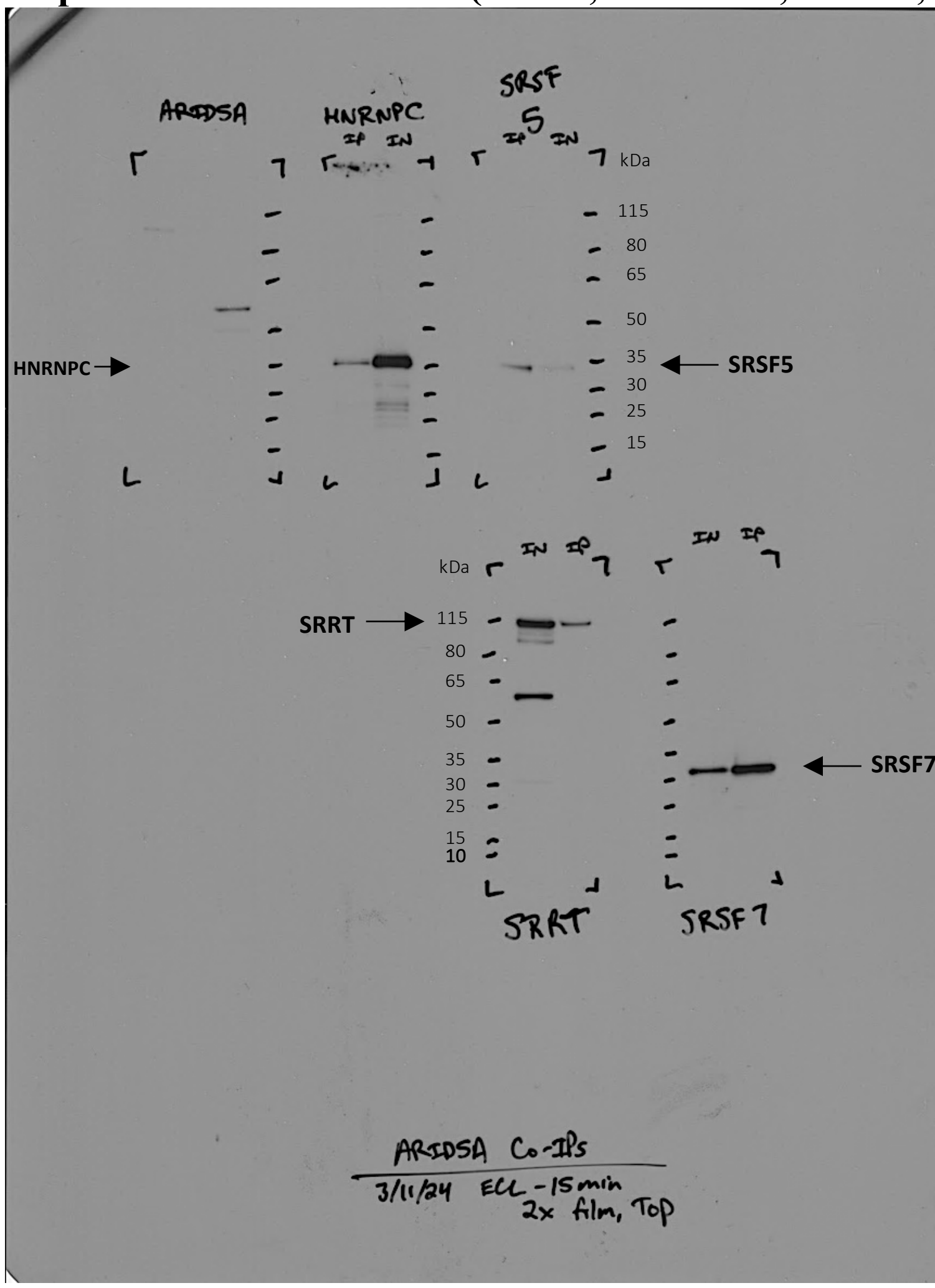


**Source Data Figure 2e Unprocessed Western Blot (MAGOH, ARID5A, RBFOX2)**

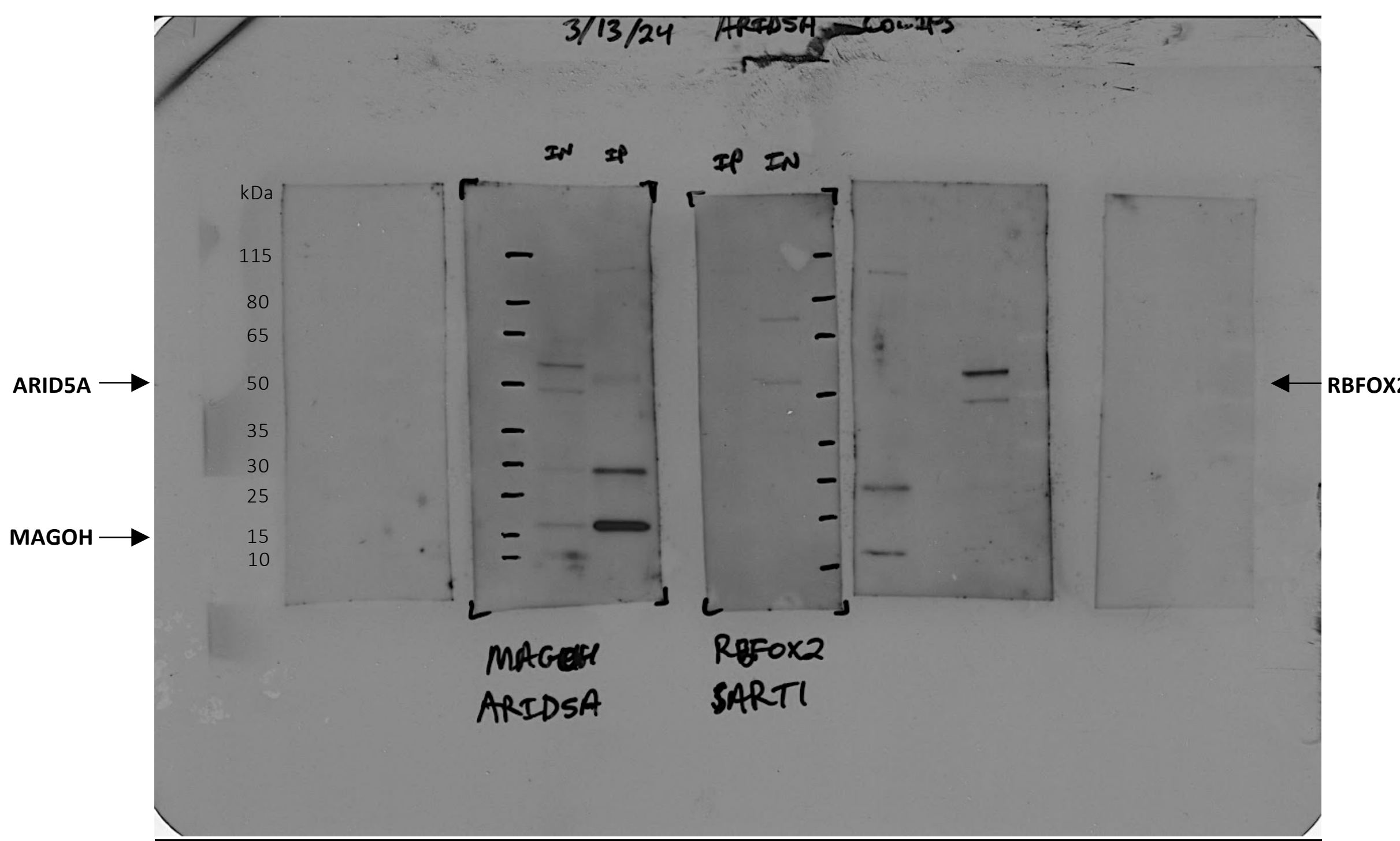

**Source Data Figure 3f Unprocessed Splicing Gel (Lowed left quadrant is GBA)**

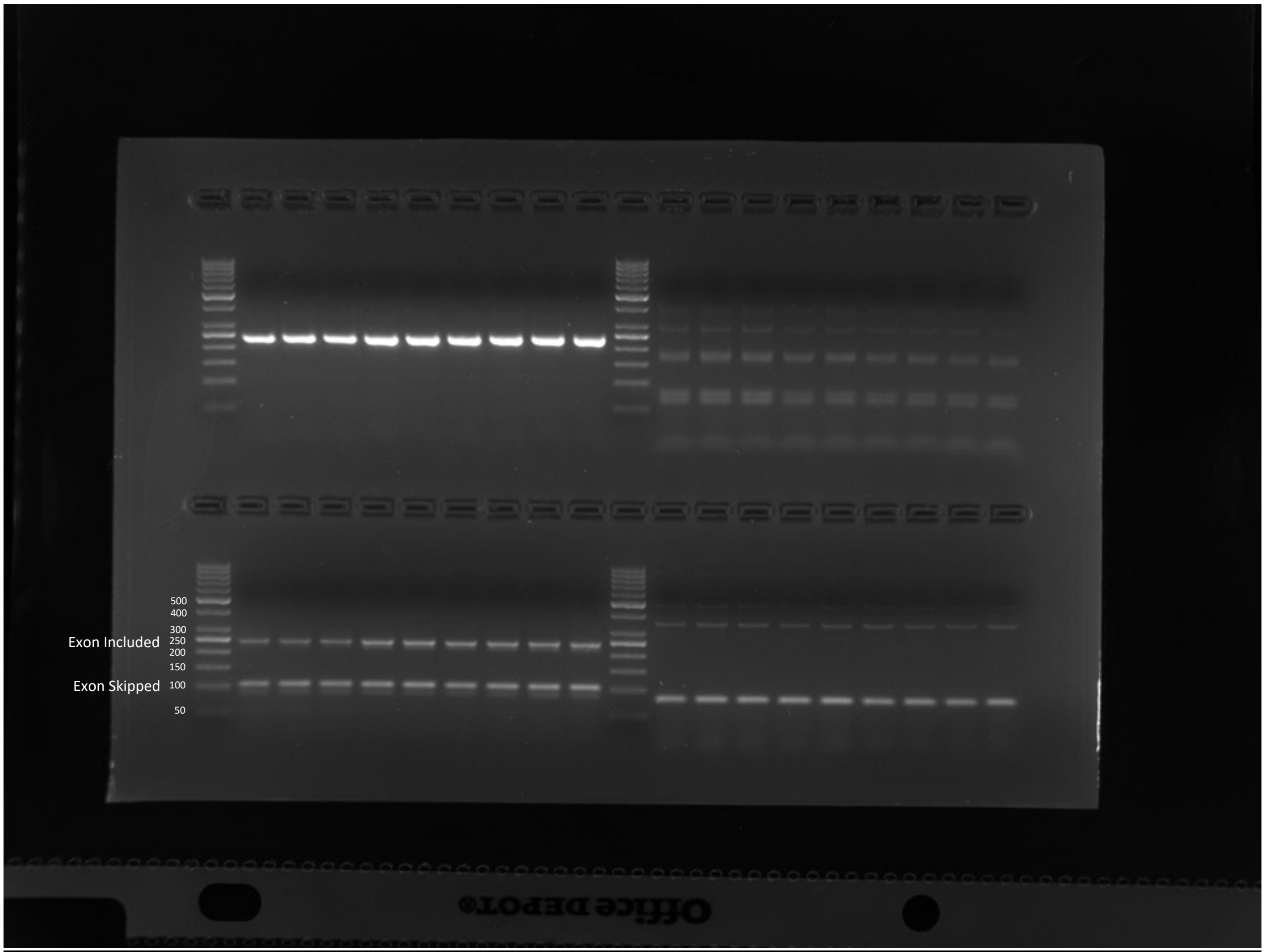

**Source Data Figure 4i Unprocessed Western Blot ISG15**

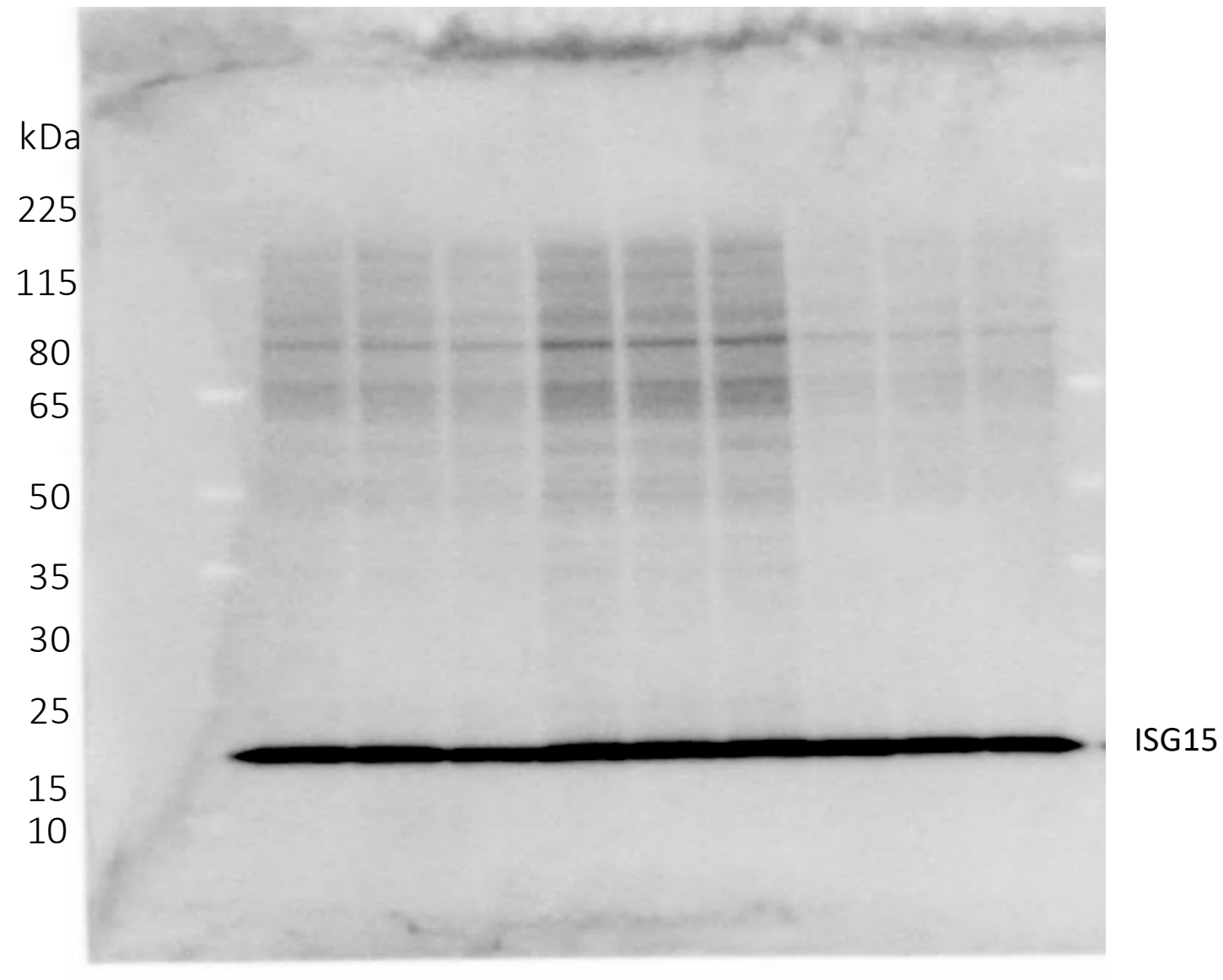


**Source Data Figure 4i Unprocessed Western Blot Beta Actin**

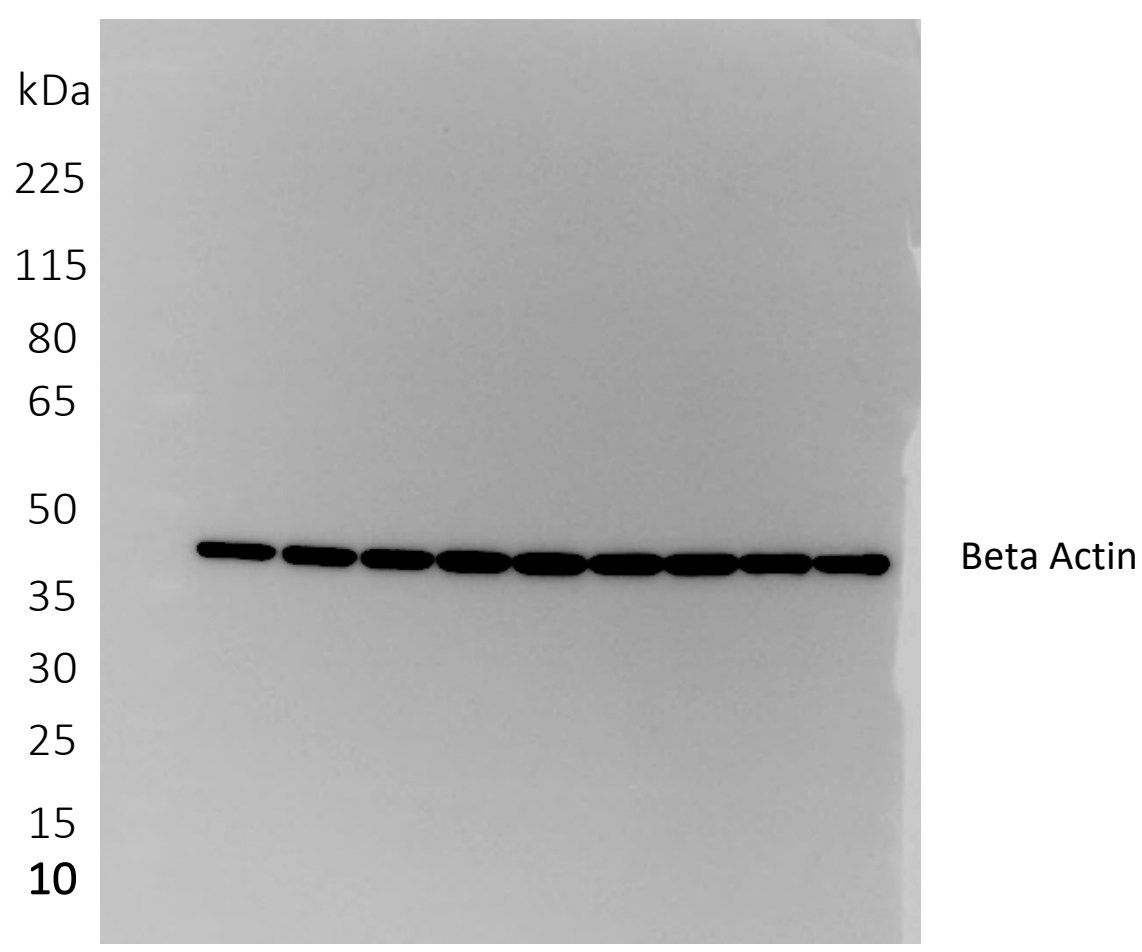


**Source Data Figure 4i Unprocessed Western Blot Total Protein**

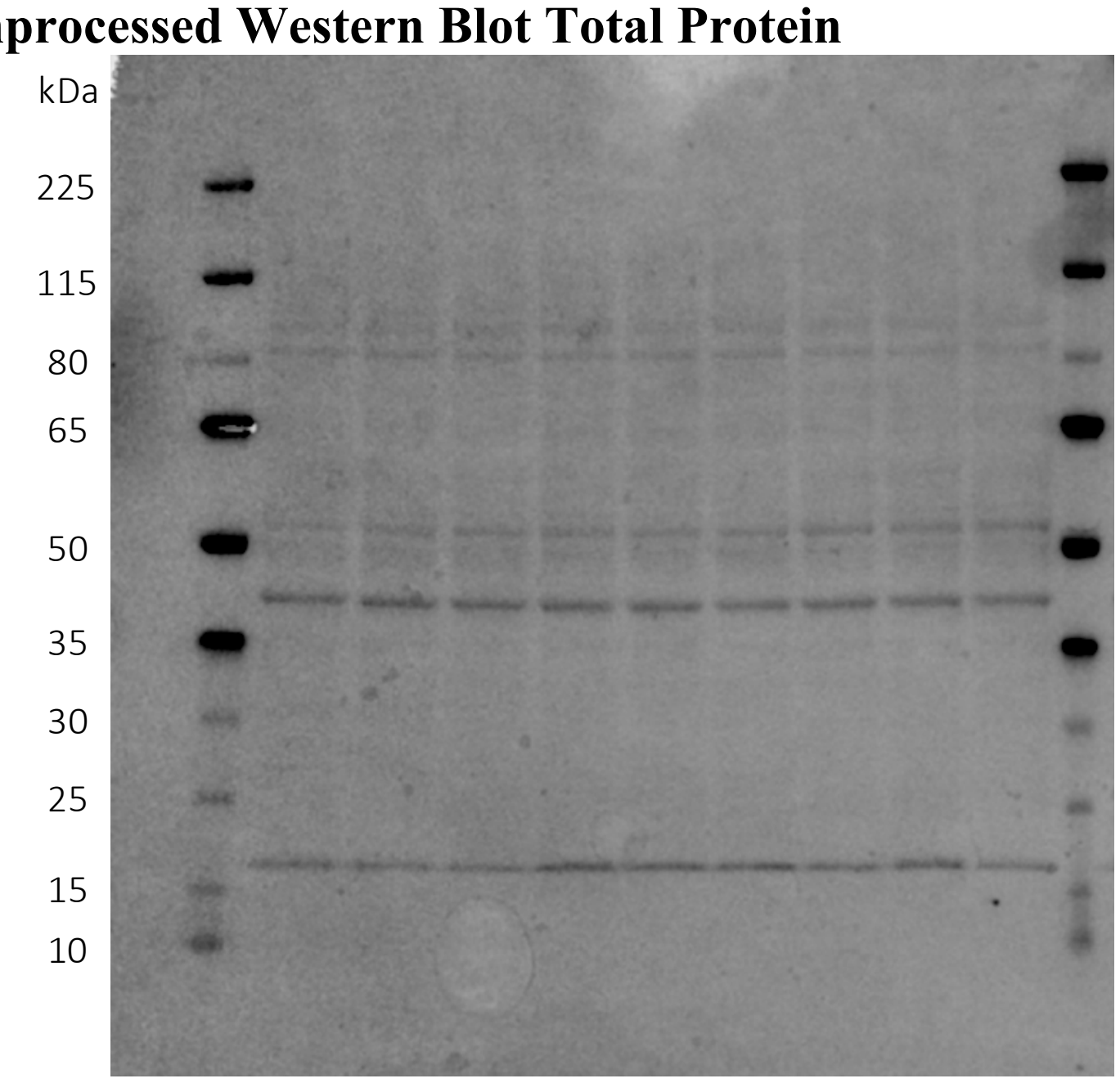

# Source Data Figure 4j Unprocessed Cytokine Array (ab133997)

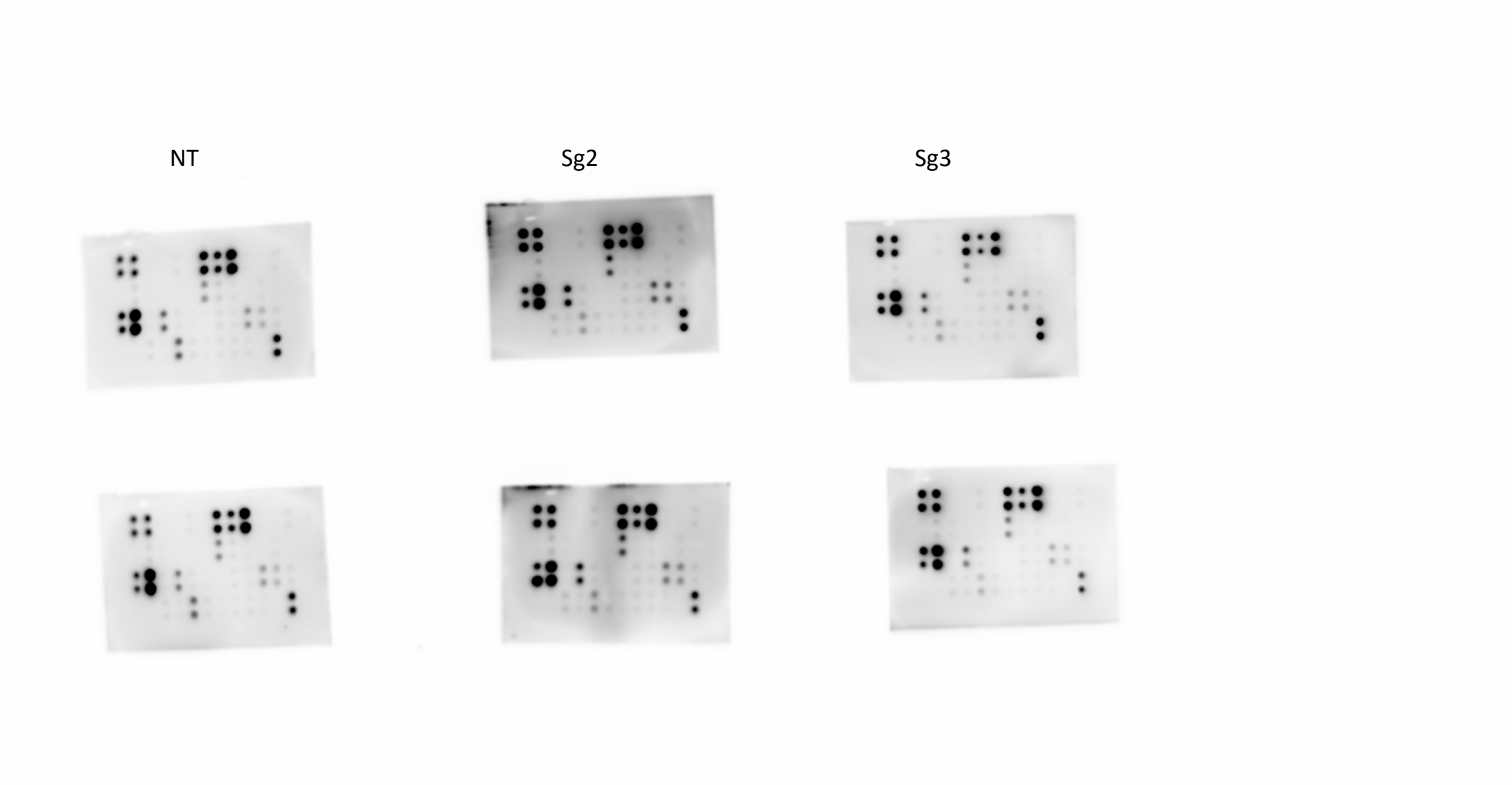

## Source Data Supplementary Figure 1e Unprocessed Western Blot Streptavidin

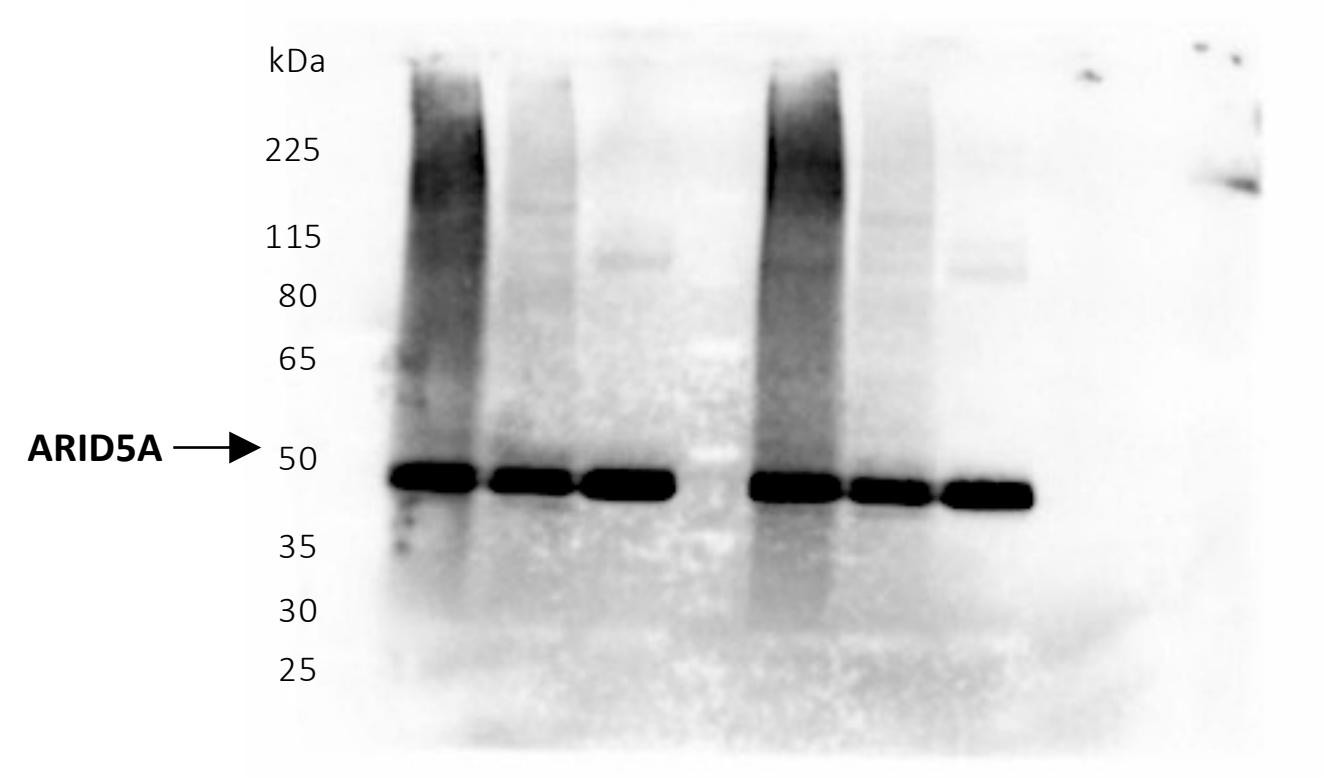


## Source Data Supplementary Figure 2h Unprocessed Western Blot Streptavidin

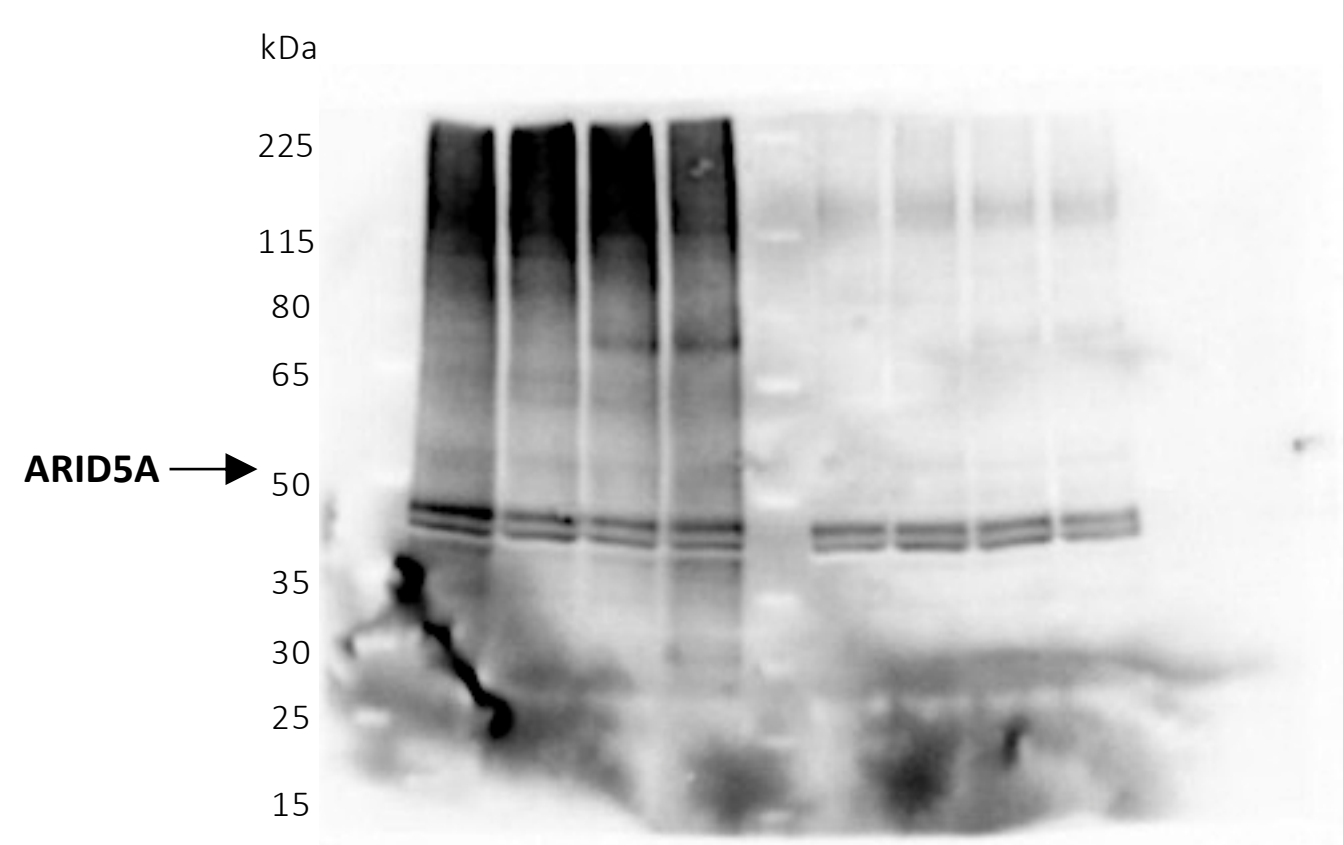


## Source Data Supplementary Figure 2h Unprocessed Western Blot V5

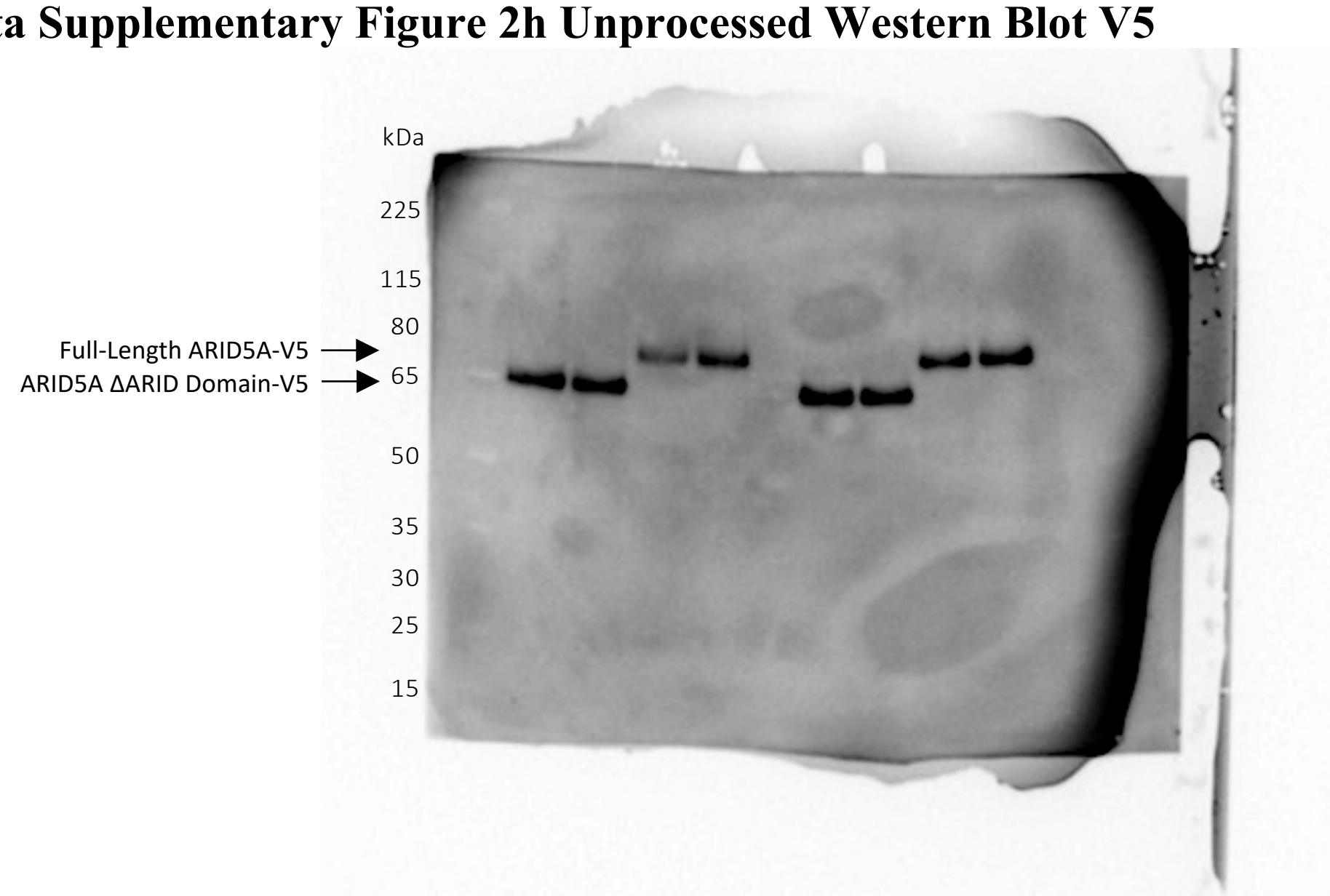


## Source Data Supplementary Figure 4e Unprocessed Western Blots

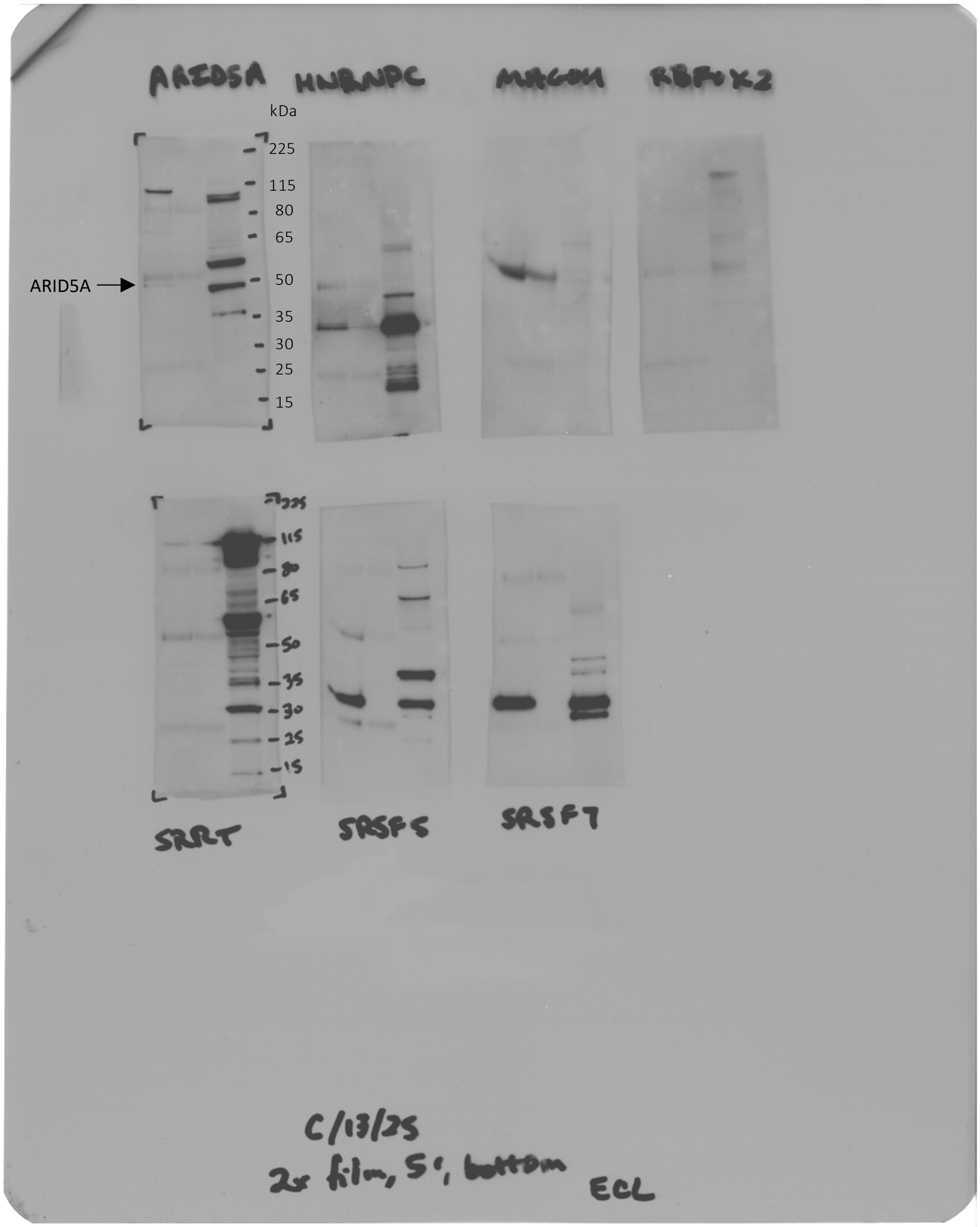
ARID5A
HNRNPC
MAGOH
RBFOX2
kDa
225
115
80
65
50
35
30
25
15
ARID5A
SRRT
SRSF5
SRSF7
2x film, 5', bottom
ECL

**Source Data Supplementary Figure 4e Unprocessed Western Blots**

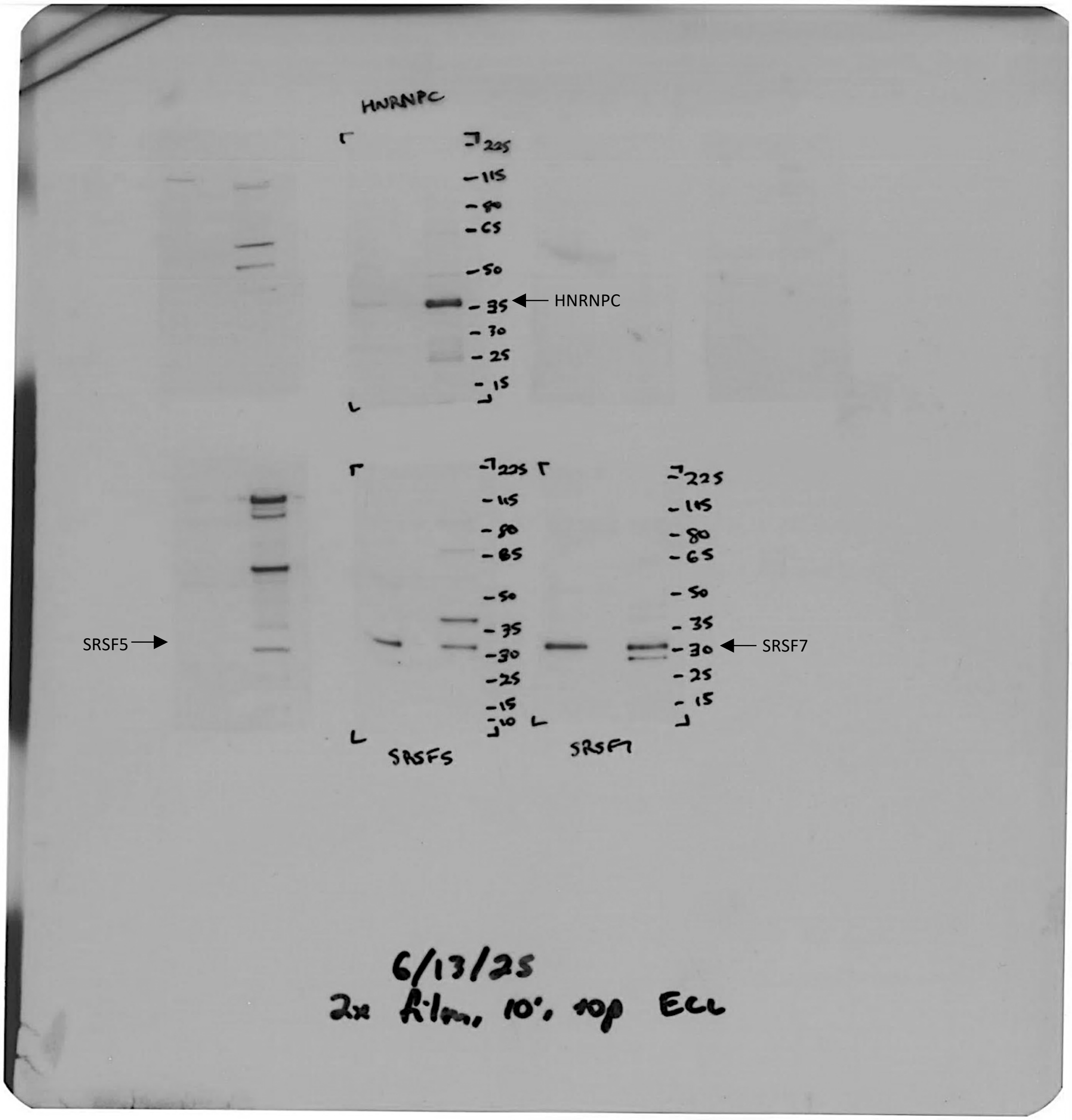

**Source Data Supplementary Figure 5b Unprocessed Western Blot Beta Actin**

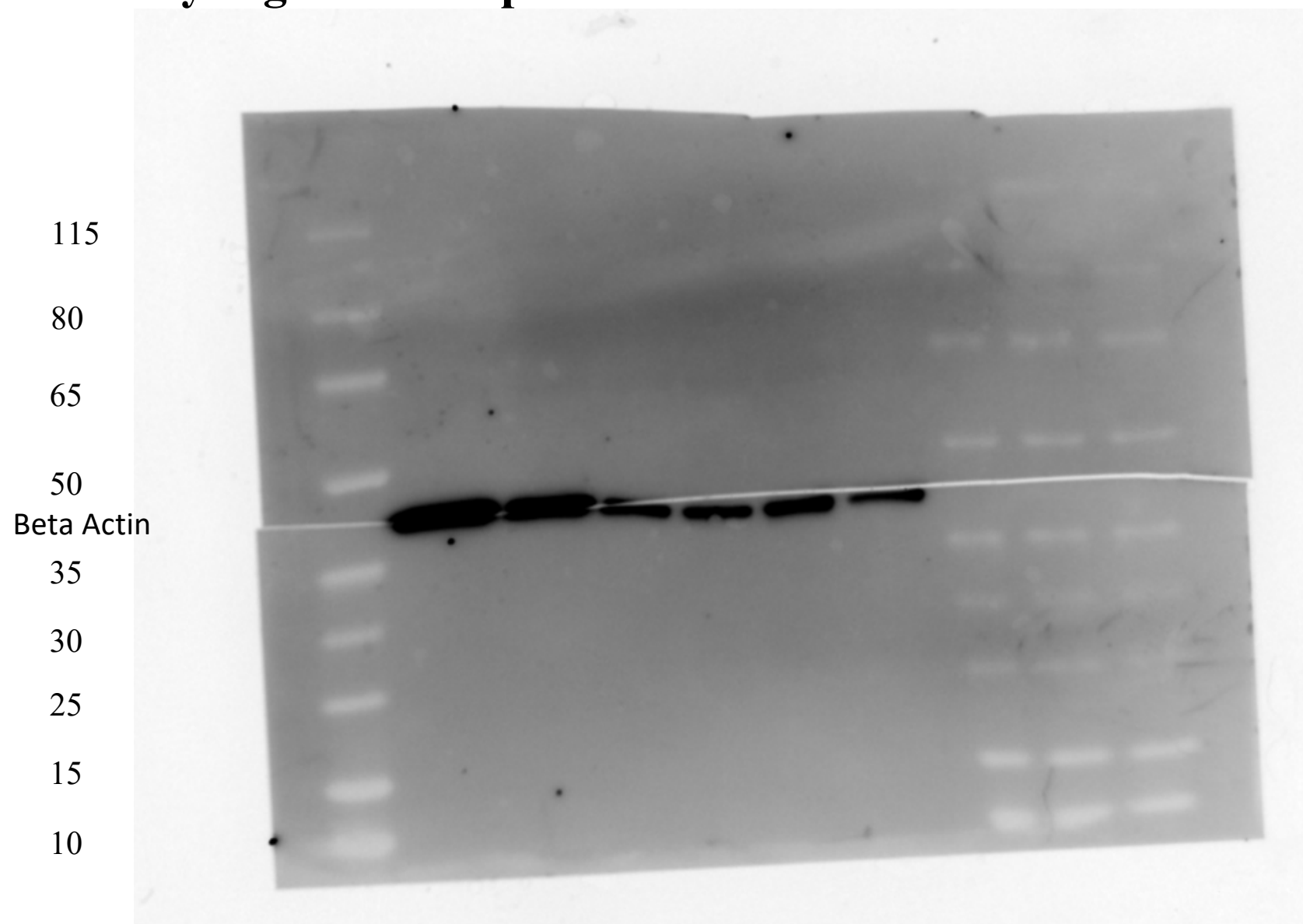


**Source Data Supplementary Figure 5b Unprocessed Western Blot ARID5A**

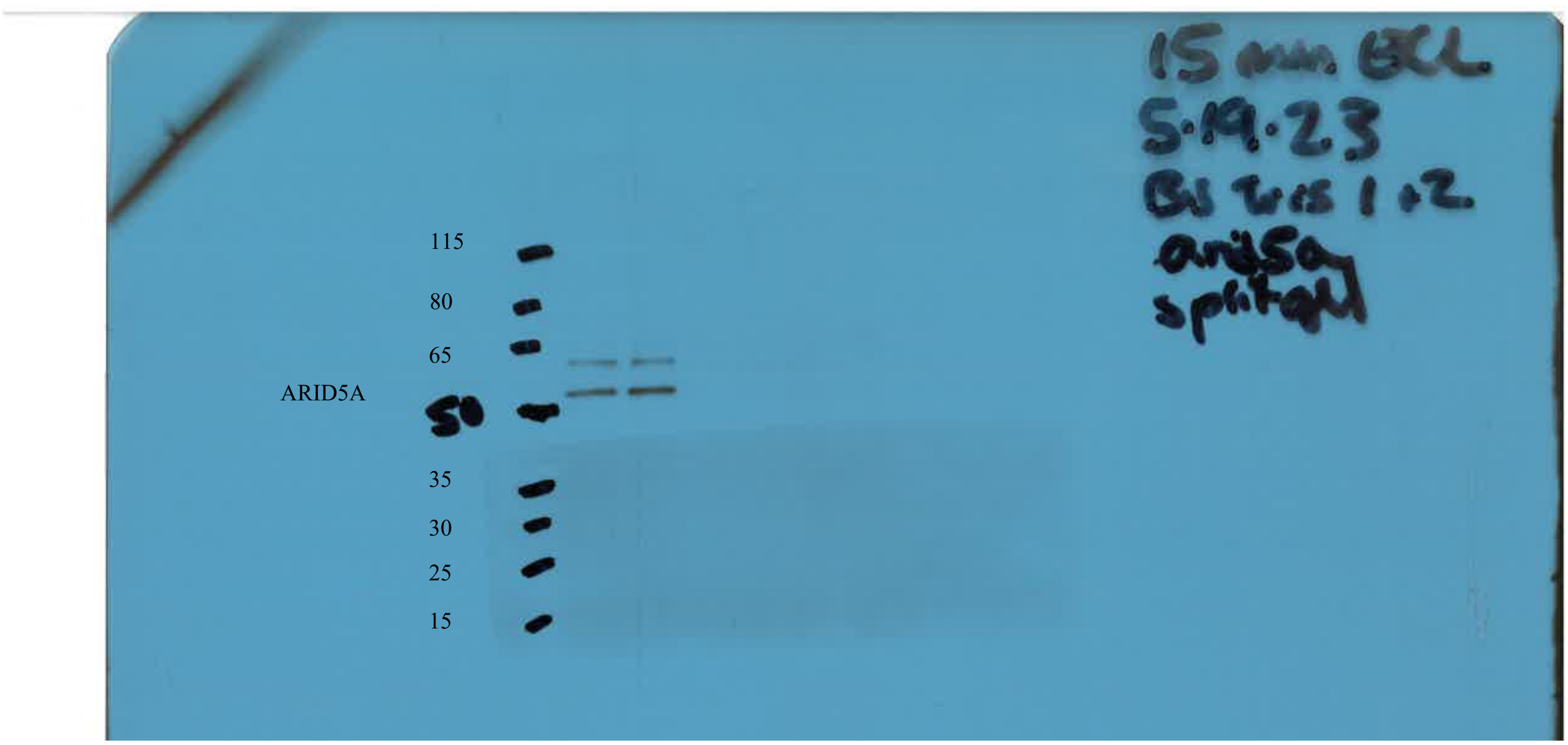

## Source Data Supplementary Figure 6c Unprocessed Western Blot Beta Actin

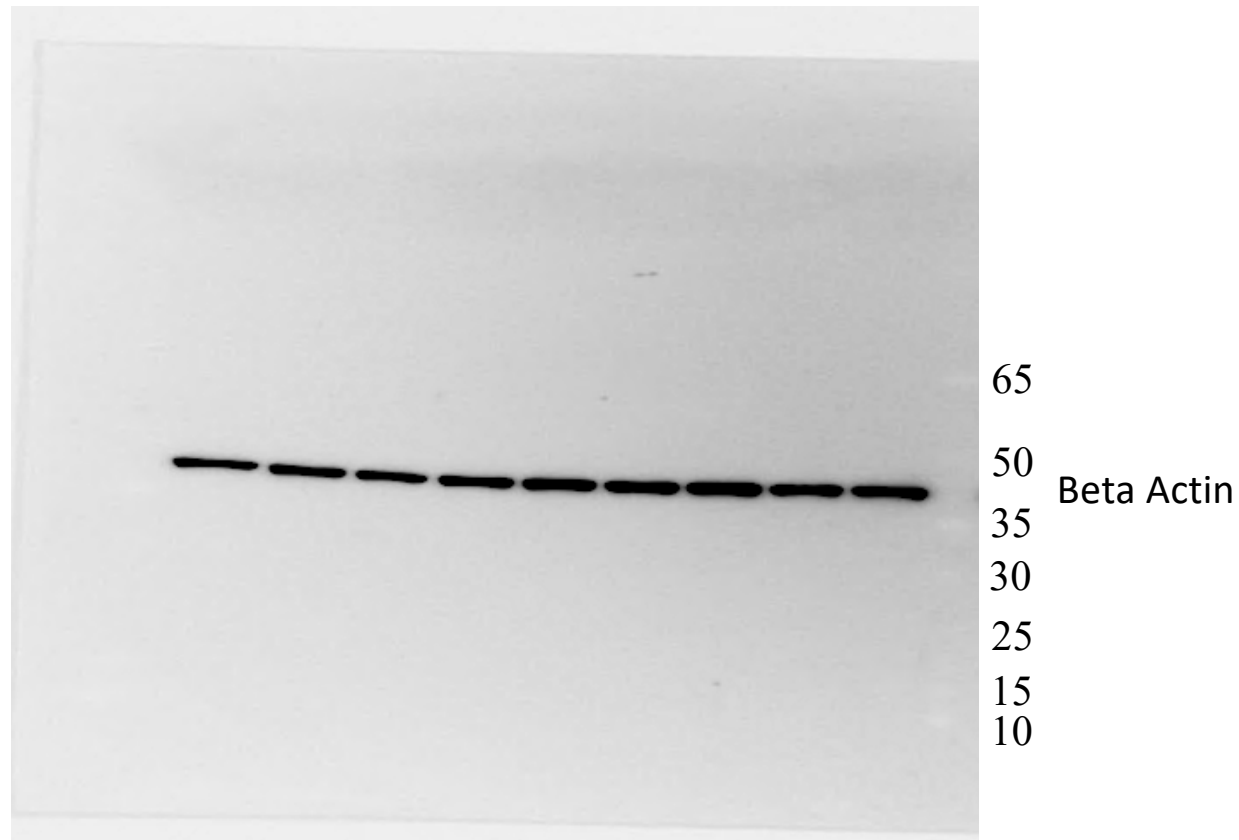


## Source Data Supplementary Figure 6c Unprocessed Western Blot GBA

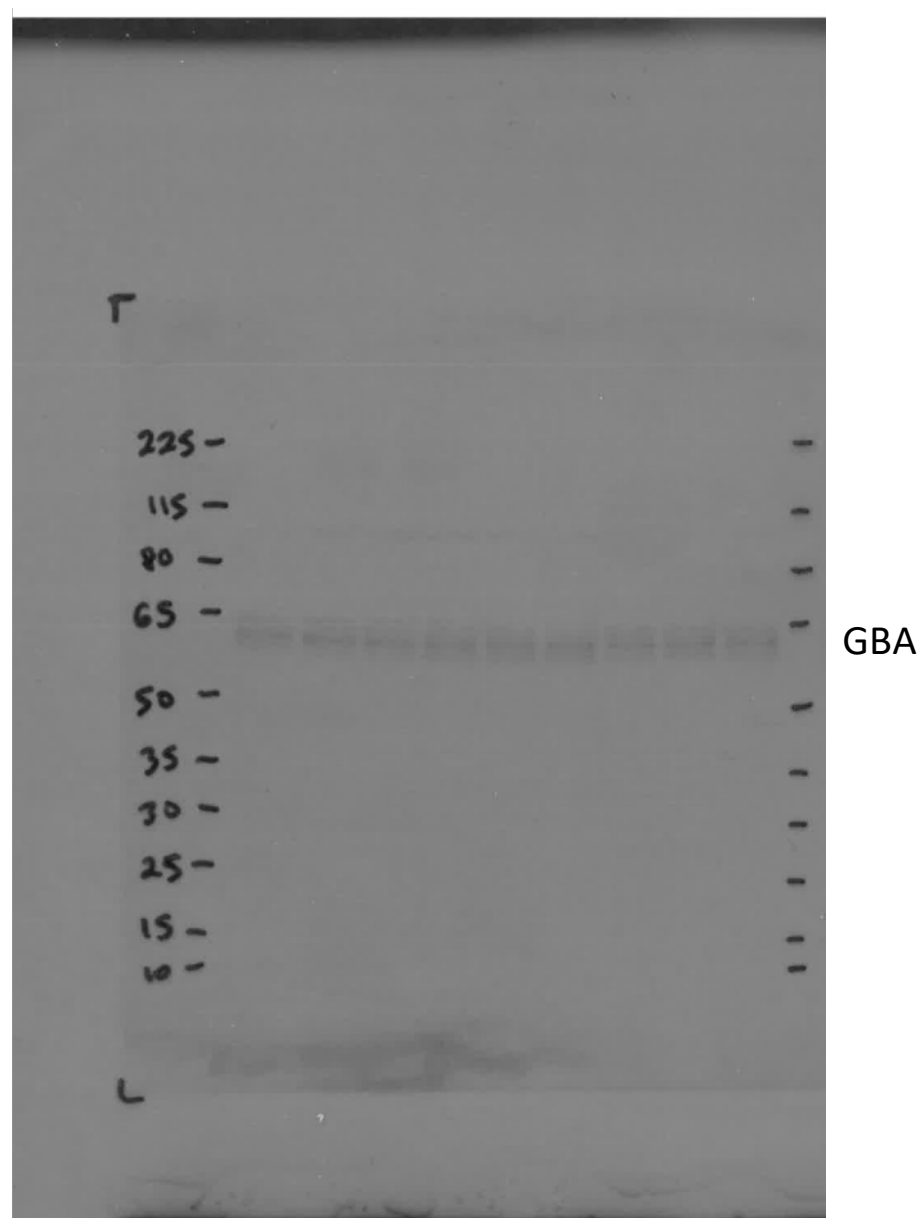


## Source Data Supplementary Figure 6c Unprocessed Western Blot Total Protein

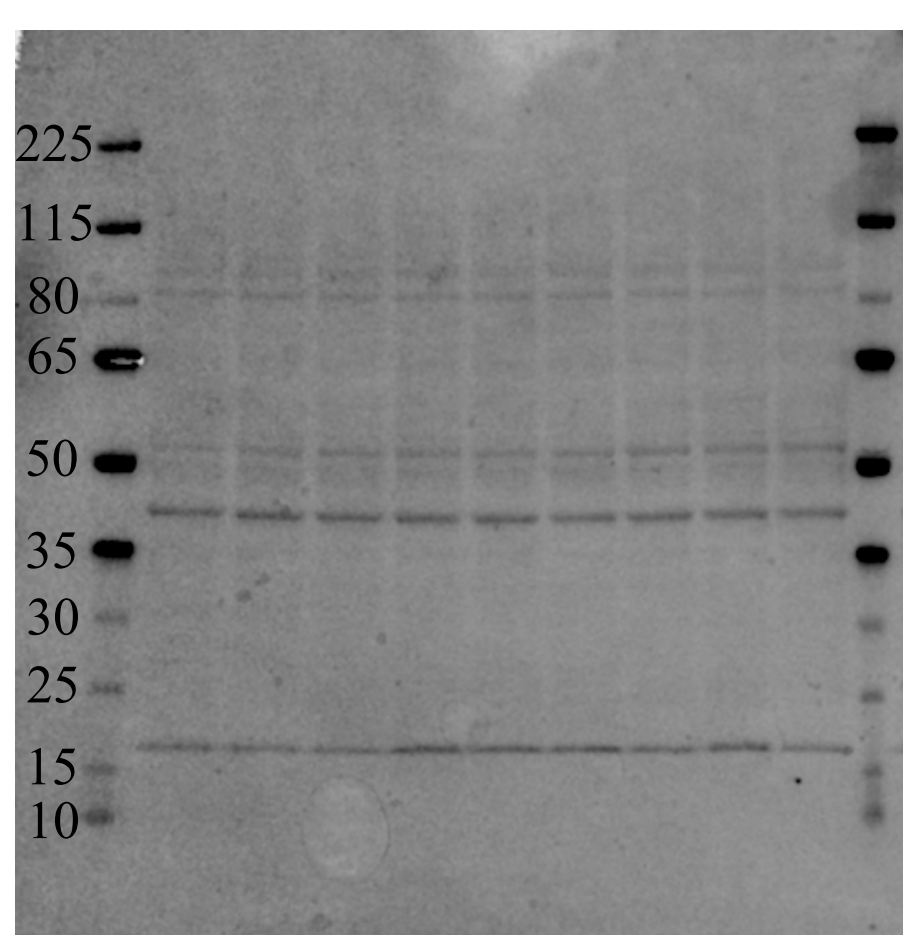

**Source Data Supplementary Figure 7f Unprocessed Western Blot ISG15**

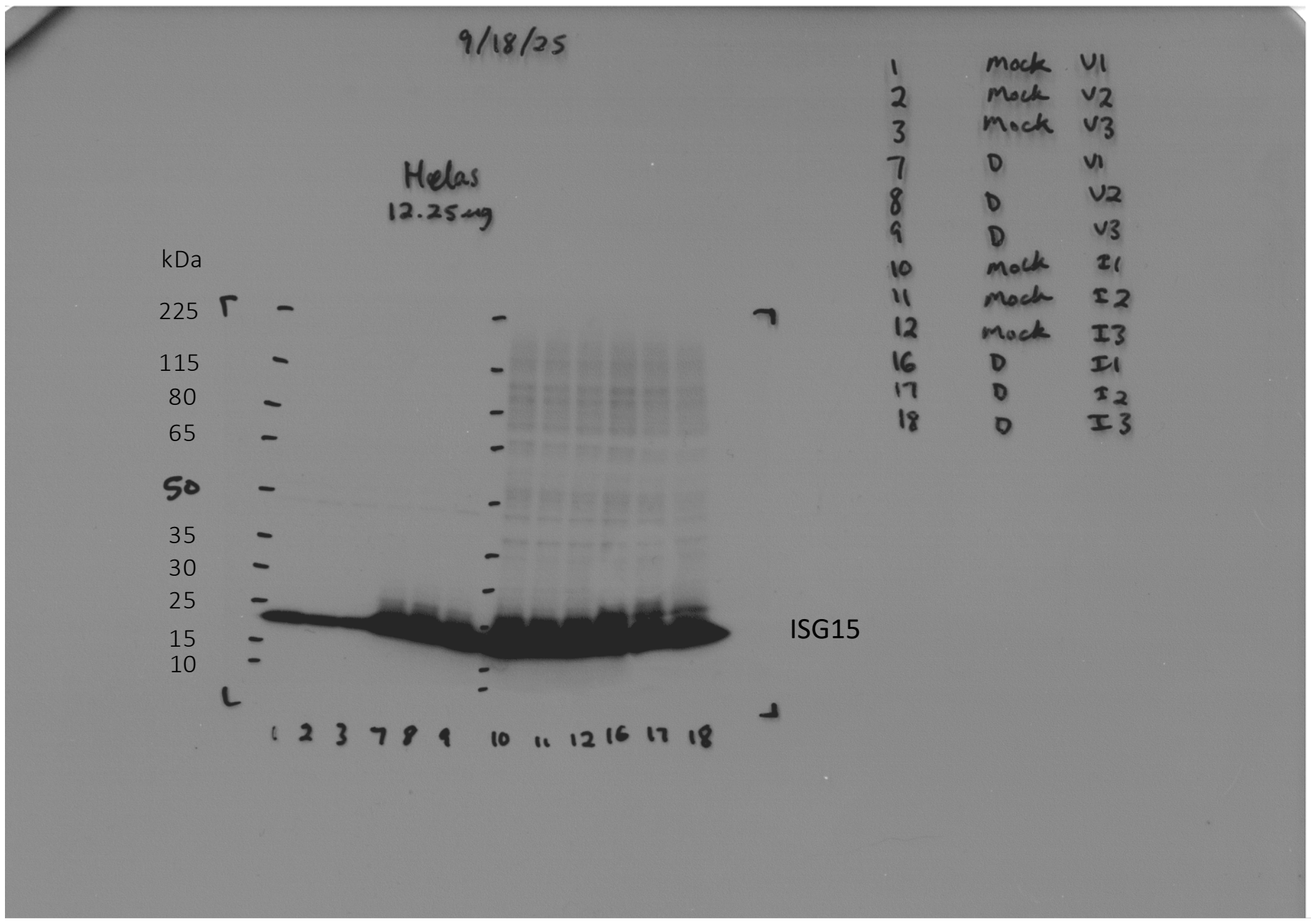


**Source Data Supplementary Figure 7f Unprocessed Western Blot Beta Actin**

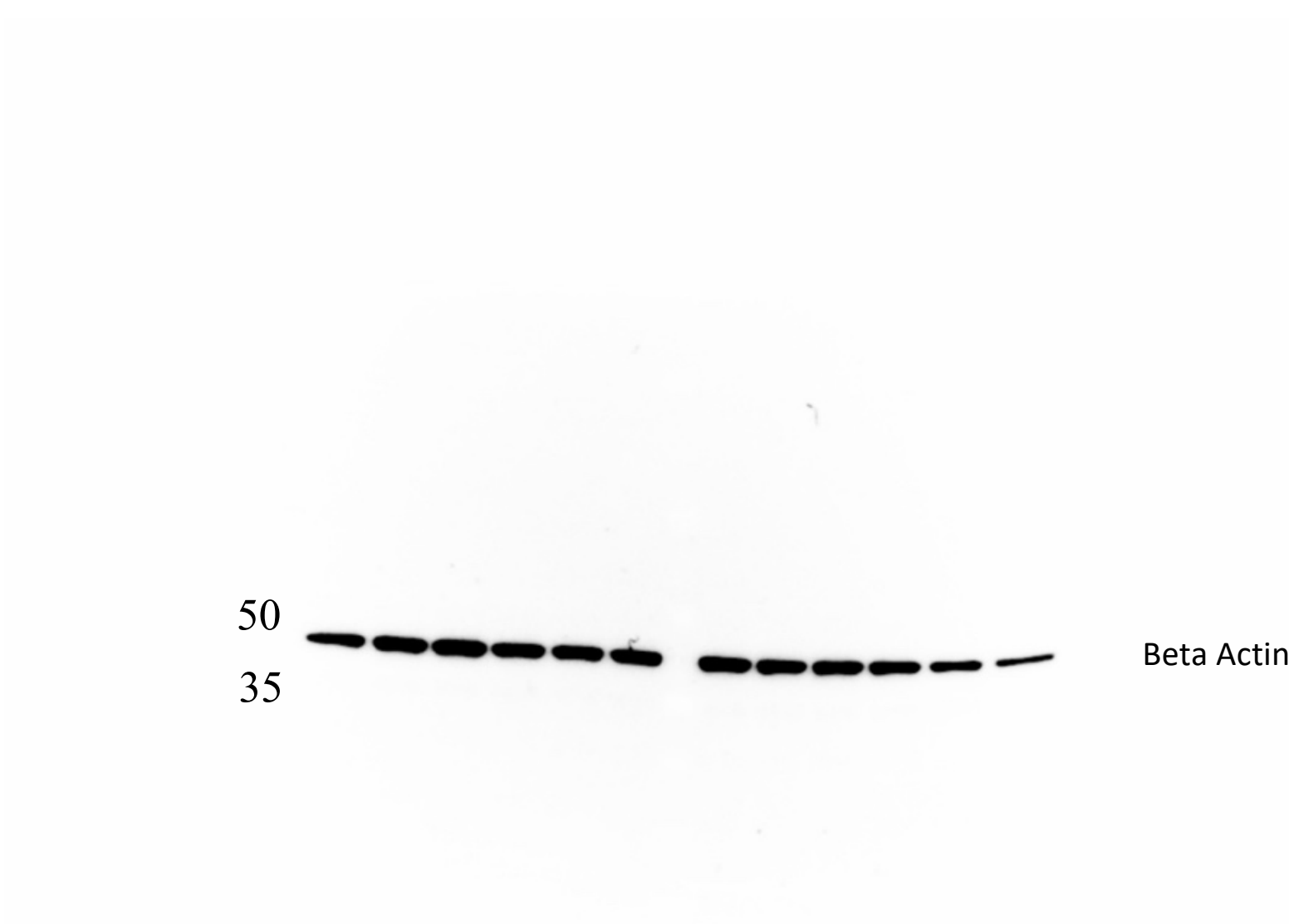


**Source Data Supplementary Figure 7f Unprocessed Western Blot Total Protein**

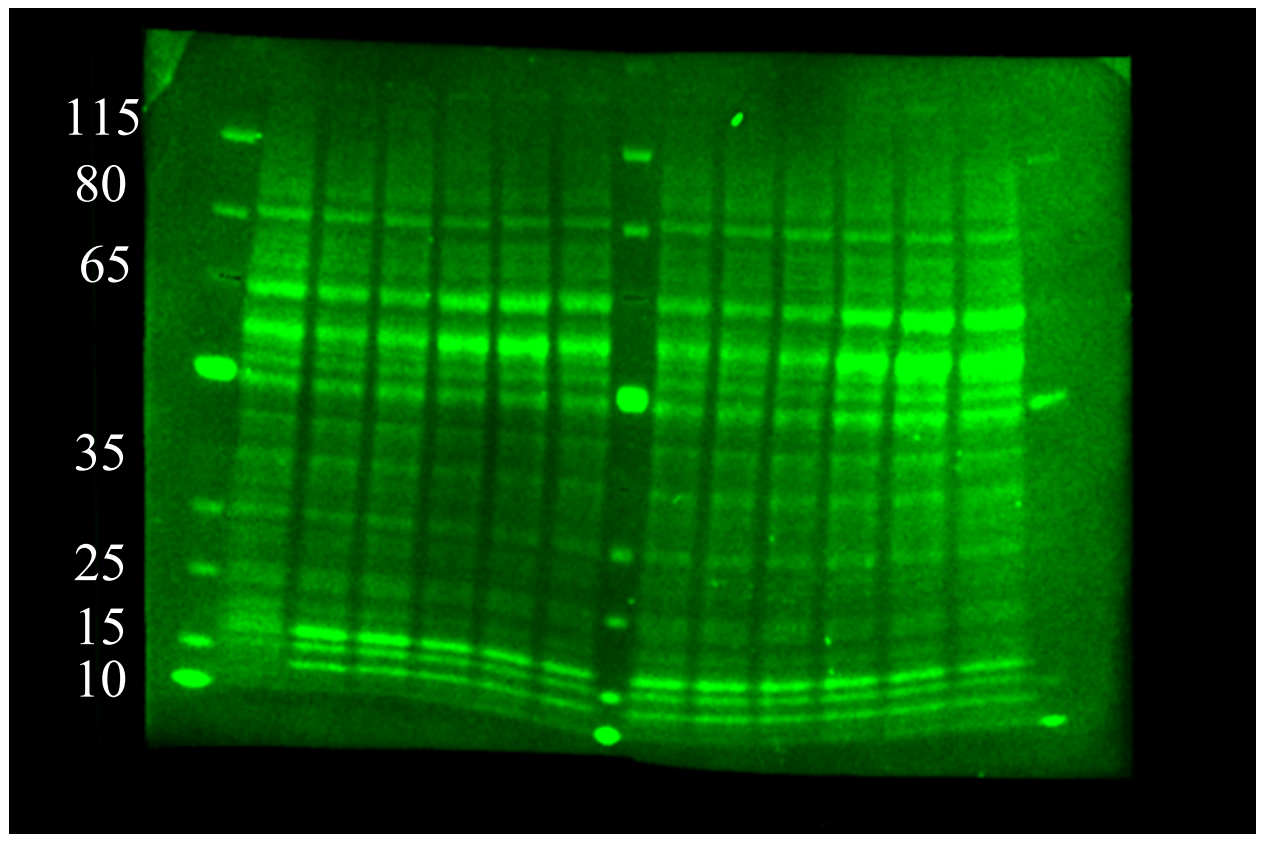
115
80
65
35
25
15
10

**Source Data Supplementary Figure 9a Unprocessed Western Blot ARID5A**

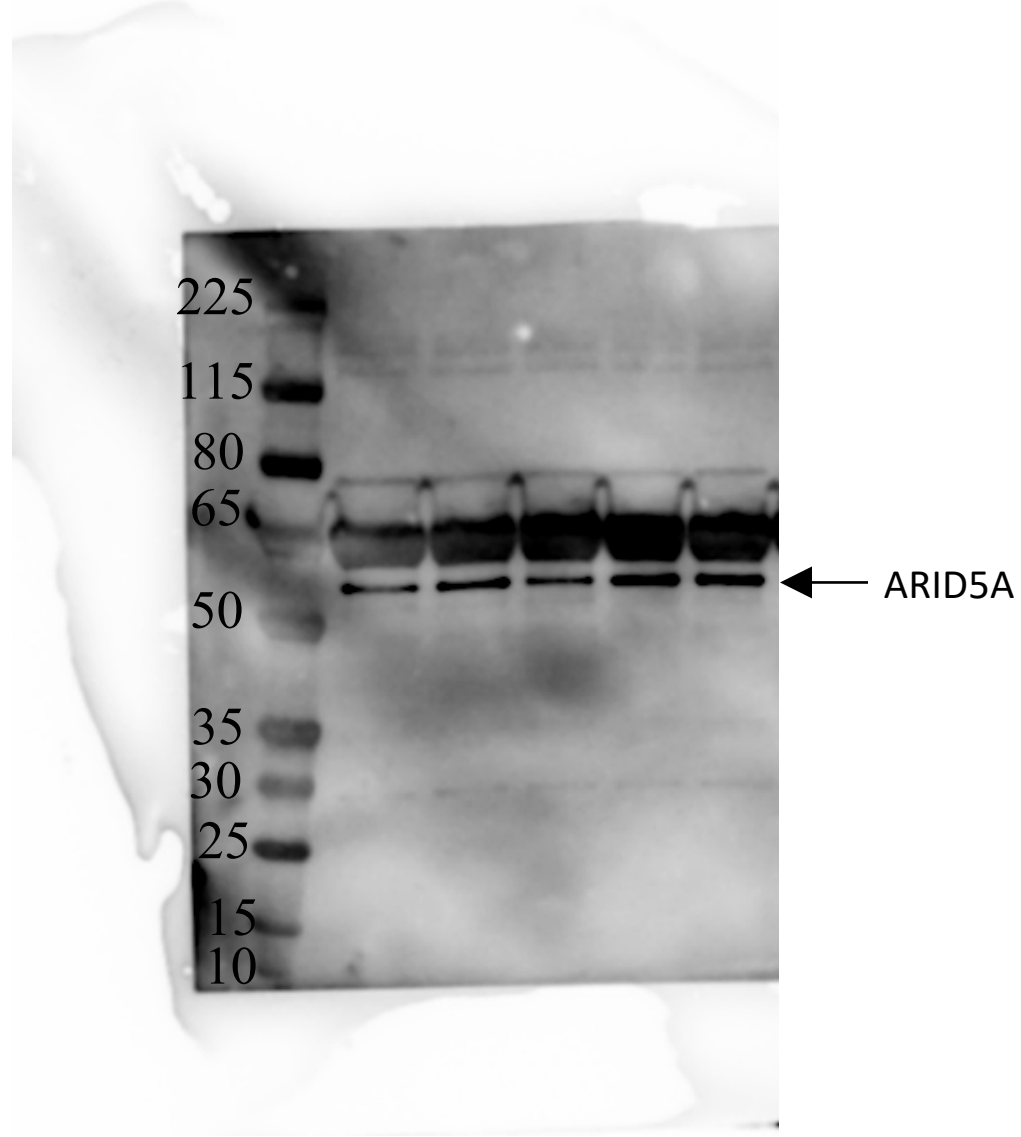


**Source Data Supplementary Figure 9a Unprocessed Western Blot Calnexin**

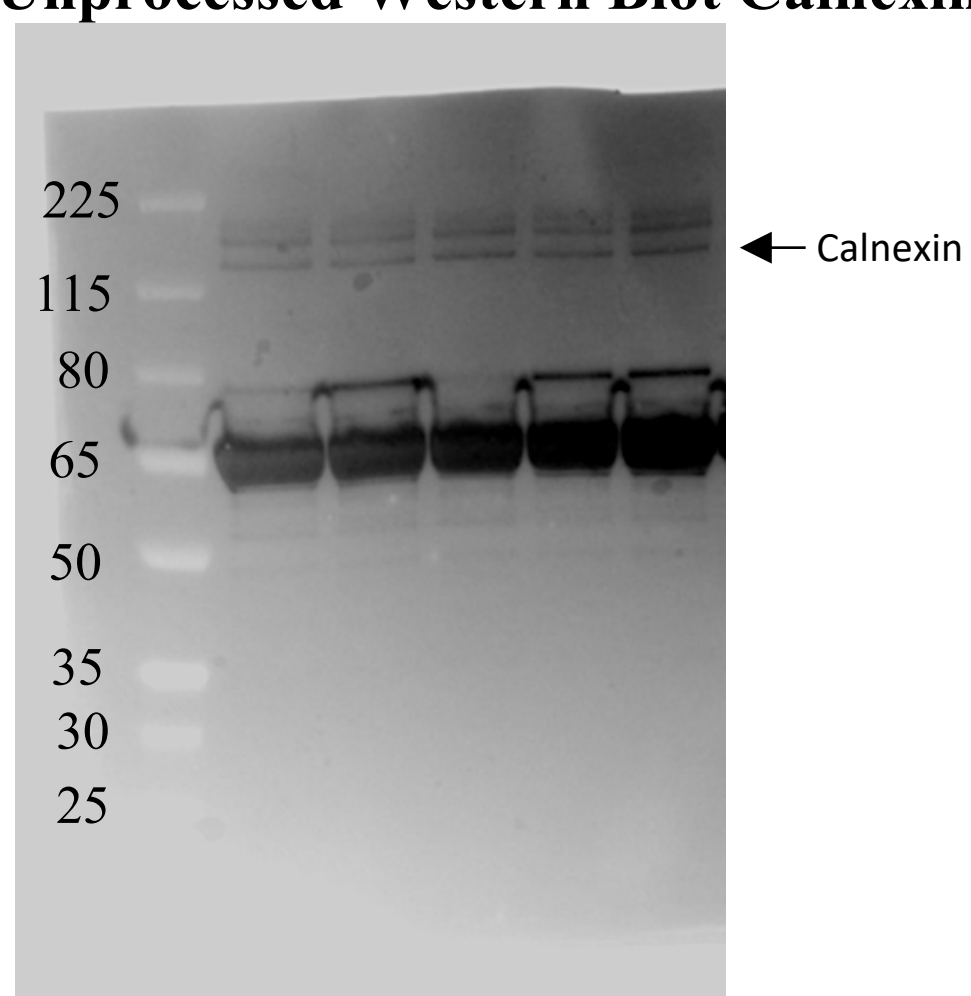


**Source Data Supplementary Figure 9a Unprocessed Western Blot Total Protein**

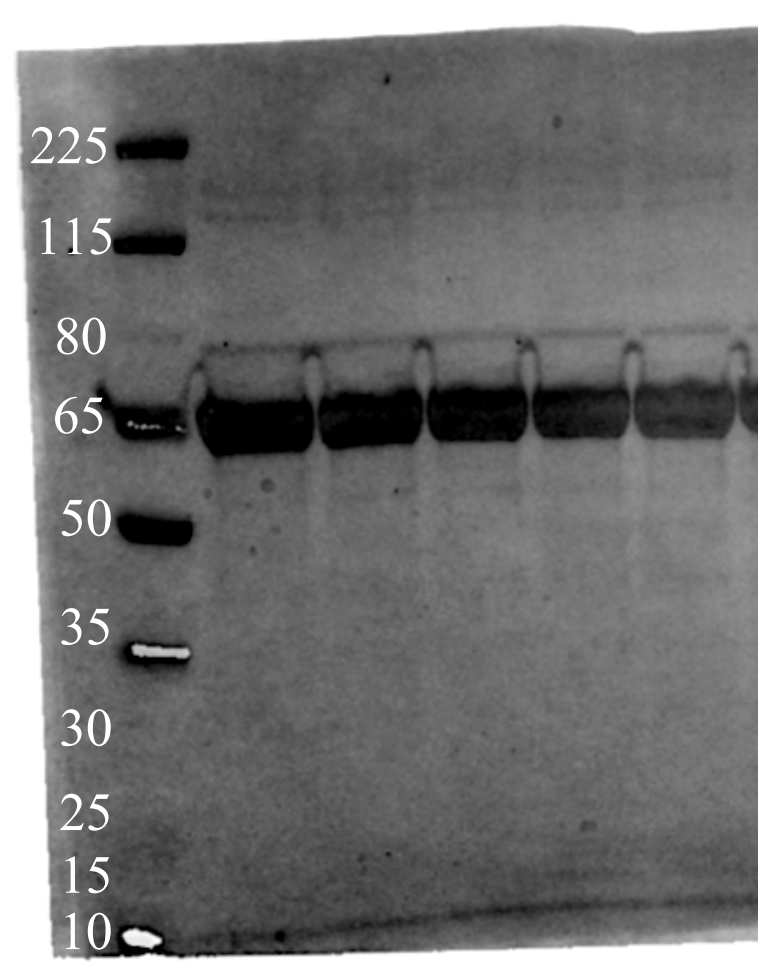

**Source Data Supplementary Figure 9e Unprocessed Western Blot ARID5A**

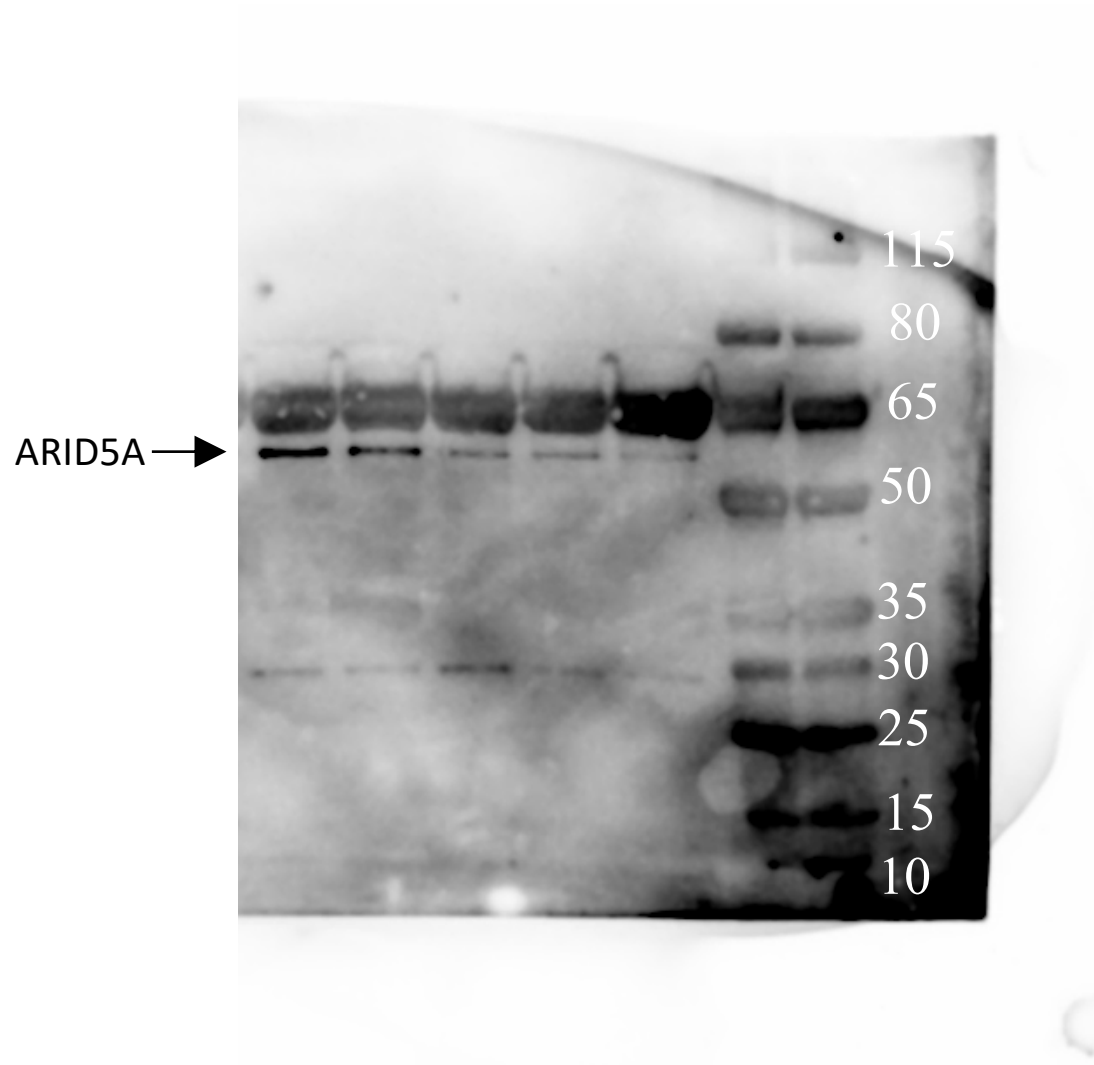


**Source Data Supplementary Figure 9e Unprocessed Western Blot Calnexin**

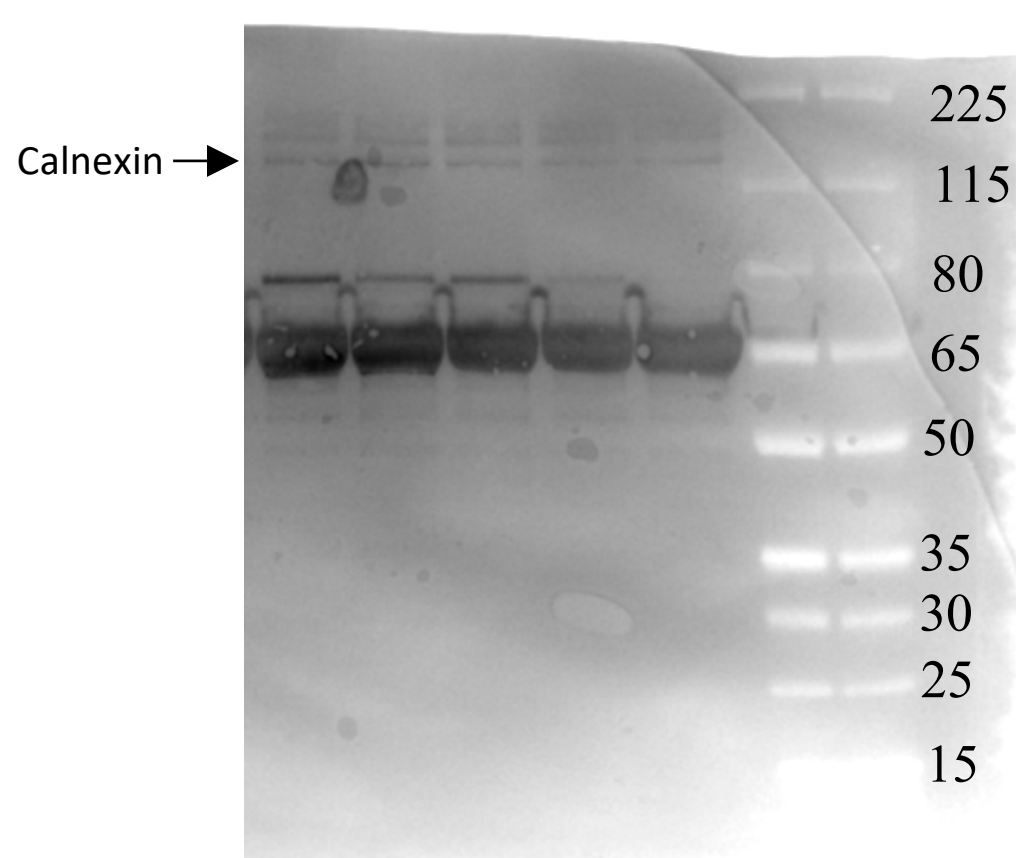


**Source Data Supplementary Figure 9e Unprocessed Western Blot Total Protein**

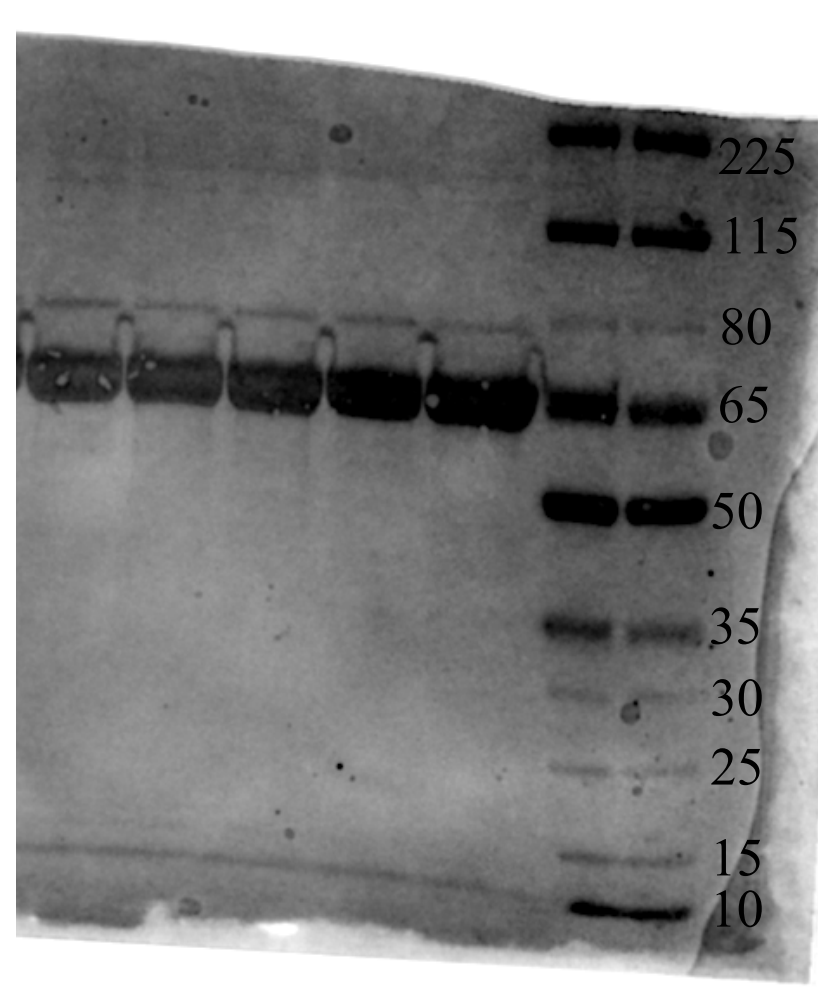

Supplement: Supplementary file 1 [file 41467_2026_76131_MOESM1_ESM.pdf]
