# Supplementary material for: ARID5A RNA-binding coordinates microglial defense and ferroptosis in iPSC-derived models
Source: Nat Commun. 2026 Aug 12;17:9687. doi: 10.1038/s41467-026-76131-0 (PMC13558697; doi:10.1038/s41467-026-76131-0)
Supplement: Supplementary file 2 [file 41467_2026_76131_MOESM2_ESM.pdf]

Supplementary Data 1 Cell-type annotation of HydRA RBPs based on the Human Protein Atlas

Table assigning HydRA RBPs as Ubiquitous, Neurons, Astrocytes, Macrophage/Microglia, Oligodendrocytes, Endothelial, or Other Immune based on Human Protein Atlas annotations.
