# Supplementary material for: ARID5A RNA-binding coordinates microglial defense and ferroptosis in iPSC-derived models
Source: Nat Commun. 2026 Aug 12;17:9687. doi: 10.1038/s41467-026-76131-0 (PMC13558697; doi:10.1038/s41467-026-76131-0)
Supplement: Supplementary file 3 [file 41467_2026_76131_MOESM4_ESM.pdf]

nature portfolio

Corresponding author(s): Gene Yeo

Last updated by author(s): Apr 30, 2026

Reporting Summary

Nature Portfolio wishes to improve the reproducibility of the work that we publish. This form provides structure for consistency and transparency in reporting. For further information on Nature Portfolio policies, see our Editorial Policies and the Editorial Policy Checklist.

Statistics

For all statistical analyses, confirm that the following items are present in the figure legend, table legend, main text, or Methods section.

n/a	Confirmed	
☐	☒	The exact sample size (*n*) for each experimental group/condition, given as a discrete number and unit of measurement
☐	☒	A statement on whether measurements were taken from distinct samples or whether the same sample was measured repeatedly
☐	☒	The statistical test(s) used AND whether they are one- or two-sided<br>*Only common tests should be described solely by name; describe more complex techniques in the Methods section.*
☐	☒	A description of all covariates tested
☐	☒	A description of any assumptions or corrections, such as tests of normality and adjustment for multiple comparisons
☐	☒	A full description of the statistical parameters including central tendency (e.g. means) or other basic estimates (e.g. regression coefficient) AND variation (e.g. standard deviation) or associated estimates of uncertainty (e.g. confidence intervals)
☐	☒	For null hypothesis testing, the test statistic (e.g. *F*, *t*, *r*) with confidence intervals, effect sizes, degrees of freedom and *P* value noted<br>*Give P values as exact values whenever suitable.*
☒	☐	For Bayesian analysis, information on the choice of priors and Markov chain Monte Carlo settings
☒	☐	For hierarchical and complex designs, identification of the appropriate level for tests and full reporting of outcomes
☒	☐	Estimates of effect sizes (e.g. Cohen's *d*, Pearson's *r*), indicating how they were calculated

Our web collection on statistics for biologists contains articles on many of the points above.

Software and code

Policy information about availability of computer code

Data collection	BD FACSDiva (8.0.2) was used for flow cytometry acquisition and Incucyte (2024B) was used for live cell imaging.
Data analysis	No custom code was generated in this manuscript. The following commercial/open-source software were used as described:<br>FlowJo(10.10): Analysis of flow cytometry as acquired on Fortessa x20<br>Incucyte (2024B): Analysis of live cell images acquired on Incucyte<br>ImageJ (1.53): Image analysis, cytokine array, and Western blot band intensity quantification<br>GraphPad Prism (8.4.3 [471]): ANOVA and t-test statistical analyses<br>Skipper: End to end eCLIP sample processing and analysis, available at https://github.com/YeoLab/skipper<br>CUT&RUN Tools (2.0.98): CUT&RUN sample processing and analysis<br>Bowtie (1.1.229): Genomic alignment for ribosome profiling<br>STAR (2.7.6a): Genomic alignment of fastq files for splicing and RIP-seq files<br>Skipper: End to end eCLIP sample processing and analysis, available at https://github.com/YeoLab/skipper<br>Samtools (1.16): Processing of SAM and BAM files throughout sequencing pipelines<br>Subread (2.0.6): Counts analysis for RIP-seq analysis<br>DESeq2 (1.34.0): Differential analysis of RIP-seq samples<br>Picard (0.1.8): Removal of PCR duplicates in CUT&RUN data<br>bowtie2 (2.3.5.1): Alignment for CUT&RUN data<br>MACS2 (2.2.7.1): Peak calling for CUT&RUN data<br>Chipseeker (1.34.0): Peak annotation

Bedtools (2.27.1): Comparison of BED files for CUT&RUN, eCLIP, and SIRES analysis
IGV (2.11.2): Visualization of genomic browser tracks
rMATS (4.3.0): Differential splicing analysis
Rmats2sashimi (3.0.0): Visualization of rMATS splicing events
Homer (4.11): Motif analysis for eCLIP and CUT&RUN data
HydRA (1.21.28): RNA binding prediction for ARID5A, available at https://github.com/Wenhao-Jin/HydRA
SIRES (3.0): Iron response element predictions, available at https://www.sires-webserver.eu/
Enrichr: Gene set enrichment analysis, available at https://maayanlab.cloud/Enrichr/
Spectronaut (16.090): Raw data analysis for AP-MS
Metascape (3.5): MCODE analysis of AP-MS processed results
Python 3.9.7: Data organization, analysis, and visualization with packages including seaborn, matplotlib, pandas, numpy, scipy, plotly, statmodels, glob, upsetplot
RStudio 1.3.1056: Visualization of R-based analyses
Jupyter Lab 4.0.4: Visualization of data analysis
Alphafold2/ColabFold (1.5): Prediction of protein structures, available at https://github.com/sokrypton/ColabFold
Pymol (3.1.1): Visualization of PDB files generated by Alphafold2/ColabFold
Cytoscape (3.10.0): Visualization of protein-protein interaction networks as defined by MCODE
ExPasy (3.0): Protparam analysis and translation of differentially spliced isoforms, available at https://www.expasy.org/

For manuscripts utilizing custom algorithms or software that are central to the research but not yet described in published literature, software must be made available to editors and reviewers. We strongly encourage code deposition in a community repository (e.g. GitHub). See the Nature Portfolio guidelines for submitting code & software for further information.

Data

Policy information about availability of data

All manuscripts must include a data availability statement. This statement should provide the following information, where applicable:

- Accession codes, unique identifiers, or web links for publicly available datasets
- A description of any restrictions on data availability
- For clinical datasets or third party data, please ensure that the statement adheres to our policy

Raw data and processed files for sequencing data are available on GEO and AP-MS data is available on MassIVE (see Supplemental Information). We reference SpliceosomeDB for spliceosome protein annotation, available at http://spliceosomedb.ucsc.edu/proteins; Uniprot (Homo sapiens, UP000005640), and Human Protein Atlas (Version 22), available at https://www.proteinatlas.org/about/download

Research involving human participants, their data, or biological material

Policy information about studies with human participants or human data. See also policy information about sex, gender (identity/presentation), and sexual orientation and race, ethnicity and racism.

Reporting on sex and gender	N/A
Reporting on race, ethnicity, or other socially relevant groupings	N/A
Population characteristics	N/A
Recruitment	N/A
Ethics oversight	N/A

Note that full information on the approval of the study protocol must also be provided in the manuscript.

Field-specific reporting

Please select the one below that is the best fit for your research. If you are not sure, read the appropriate sections before making your selection.

[x] Life sciences [ ] Behavioural & social sciences [ ] Ecological, evolutionary & environmental sciences

For a reference copy of the document with all sections, see nature.com/documents/nr-reporting-summary-flat.pdf

Life sciences study design

All studies must disclose on these points even when the disclosure is negative.

Sample size	Sample size for each experiment is provided in the figure legend and was determined by previous publications as cited in the manuscript within each method section.
Data exclusions	No data were excluded.
Replication	Experiments were performed in biological duplicate and triplicate and orthogonal methods were used to confirm key results. Many measures

	were taken to ensure reproducibility including precise replication of timing in regards to differentiations and live cell imaging/flow cytometry, aliquoting of growth factor reagents to avoid freeze thaw effects, and standardized experimental parameters such as laser intensity for data acquisition. Findings were successfully replicated over the course of two years of experiments.
Randomization	No randomization of samples was used because all experiments were conducted in tightly controlled experimental conditions in our cell models, with all modifications (treatments, genotypes) measured simultaneously.
Blinding	Blinding was not relevant to our study because data was exclusively measured by quantification determined by software rather than any subjective scoring methods.

Reporting for specific materials, systems and methods

We require information from authors about some types of materials, experimental systems and methods used in many studies. Here, indicate whether each material, system or method listed is relevant to your study. If you are not sure if a list item applies to your research, read the appropriate section before selecting a response.

Materials & experimental systems

n/a	Involved in the study
☐	☒ Antibodies
☐	☒ Eukaryotic cell lines
☒	☐ Palaeontology and archaeology
☒	☐ Animals and other organisms
☒	☐ Clinical data
☒	☐ Dual use research of concern
☒	☐ Plants

Methods

n/a	Involved in the study
☒	☐ ChIP-seq
☐	☒ Flow cytometry
☒	☐ MRI-based neuroimaging

Antibodies

Antibodies used	Due to constraints of the Reporting Summary, the entire list of antibodies used in this manuscript are included in the methods as “Table 1”.
Validation	All antibodies used for Western blot and immunofluorescence were validated by the manufacturer. V5 antibodies were validated for IP by previous publications (Schmok et al. 2024) and ARID5A was independently validated for IP as shown in the Supplemental Information.

Eukaryotic cell lines

Policy information about cell lines and Sex and Gender in Research

Cell line source(s)	KOLF2.1J and TREM2 T66M cell lines were acquired from the iNDi consortium. WTC-11 (GM25256) was purchased from Coriell. The iTF cell line prior to sgRNA introduction was acquired from the Kampmann Lab at UCSF. HeLa, and THP-1 cells were acquired from ATCC and Lenti-X HEK293T cells were acquired from Takara.
Authentication	Confirmation of microglia was validated by IBA1 staining, as shown in Figure 5. Organoids were validated as containing multiple CNS cell types by Western blotting as shown in Figure 1. THP-1s were validated as successfully differentiated to macrophages based on morphology transitions from suspension to adherent cells and subsequent tests of phagocytic capability.
Mycoplasma contamination	The cell lines used in this study were routinely and frequently tested negative for mycoplasma contamination.
Commonly misidentified lines (See ICLAC register)	None of our lines were listed in the ICLAC register version 13 (Lenti-X HEK293T cells were not listed).

Plants

Seed stocks	N/A
Novel plant genotypes	N/A
Authentication	N/A

Flow Cytometry

Plots

Confirm that:

- [x] The axis labels state the marker and fluorochrome used (e.g. CD4-FITC).
- [x] The axis scales are clearly visible. Include numbers along axes only for bottom left plot of group (a 'group' is an analysis of identical markers).
- [x] All plots are contour plots with outliers or pseudocolor plots.
- [x] A numerical value for number of cells or percentage (with statistics) is provided.

Methodology

Sample preparation	Both iPSC-derived microglia were labeled with live cell dyes at the concentrations and durations detailed in the Methods Table 2. Cells were stained in FluroBrite DMEM in the incubator until collection. Samples were washed three times with FluroBrite DMEM prior to collection. Cells were resuspended and immediately analyzed by flow cytometry. In between measurements, samples were maintained on ice and protected from light. All experiments included unstained controls to determine gating for each experiment.
Instrument	BD LSRFortessa X-20
Software	BD FACSDiva (8.0.2) was used to acquire flow cytometry measurements. Files were exported as fcs files and analyzed in FlowJo (10.10). No custom code was generated for this manuscript.
Cell population abundance	Cells were only analyzed for fluorescent signal as indicated by live cell dyes or internalization of fluorescently-tagged (pHrodo red) amyloid beta protein. Starting populations were pure and exclusively microglia. Fluorescent intensity was determined by first gating on unstained controls and applying those gates across all samples within the experiment. Median fluorescence intensity was calculated by FlowJo (10.10).
Gating strategy	Live and single cells were identified by gating FSC/SSC populations, as shown in the Gating Strategy in the Supplemental Information. All gates were determined by the unstained control samples and applied uniformly across all experimental samples.

- [x] Tick this box to confirm that a figure exemplifying the gating strategy is provided in the Supplementary Information.
