# Supplementary material for: ARID5A RNA-binding coordinates microglial defense and ferroptosis in iPSC-derived models
Source: Nat Commun. 2026 Aug 12;17:9687. doi: 10.1038/s41467-026-76131-0 (PMC13558697; doi:10.1038/s41467-026-76131-0)
Supplement: Supplementary file 4 [file 41467_2026_76131_MOESM5_ESM.pdf]

nature portfolio

Peer Review File

ARID5A RNA-binding Coordinates Microglial Defense and Ferroptosis

Corresponding Author: Professor Gene Yeo

This file contains all reviewer reports in order by version, followed by all author rebuttals in order by version.

Version 0:

Reviewer comments:

Reviewer #1

(Remarks to the Author)
The manuscript by Schafer et al presents an ambitious and multifaceted study exploring the RNA-binding protein ARID5A and its role in microglial function, combining eCLIP, RNA sequencing, ribosome profiling, protein interaction mapping with functional assays in microglia. The findings are of potential significance, particularly given the emerging interest in RBPs in their diverse regulatory roles and cell-type specific contexts. The authors have produced high quality sequencing data sets and proteomics but several methodological, interpretational, and presentation issues limit the current impact of the study. I would strongly encourage the authors to focus on clarifying their mechanistic claims, and strengthen the narrative thread across the study. With these revisions, the manuscript would constitute a valuable contribution to the field and has the potential to advance understanding of a cell type-specific RBP in neuroimmune contexts. However, it currently lacks critical validation and cohesion. Below are major and minor points organized to guide revision.

Major points:
Experimental Validation and Interpretation Gaps
• The manuscript proposes several distinct functions for ARID5A (e.g., in lysosomal regulation, splicing, ferroptosis, and immune modulation) without adequately demonstrating causality for all of these functions. It appears that this protein has three very distinct modes of action (as an DNA or RNA-binding proteins active in the nuleus and cytoplasm). While this is not unknown for RBPs, it is not clear what specific feature qualifies the protein to be involved in all of these processes (just location, modification, solely interactors?).
• Rescue experiments are essential across relevant functional assays to distinguish ARID5A-specific effects from off-target phenomena.
• Figure 3: For example, considering that nearly 9000 mRNAs were bound and 300 mRNAs appear to be dependent on ARID5A expression, the rationale for narrowing down on lysosomal function does not appear very straight-forward. If the ARID5A-mediated GBA splicing is indeed causing differential lysosomal activity, a rescue by providing an intact GBA version should be provided. This would be a necessary experiment to confirm that GBA is the key gene here.
• In addition, one should consider generating RNA or DNA-binding mutants that can disentangle the different roles. Otherwise, the paper remains somewhat descriptive.
• Figure 1: For example, can the authors see differences in iron metabolism when they generate RNA-binding deficient mutants.
• Figure 4: Given that the effect on mRNA splicing was comparatively mild (see comment below on Figure 3F), it might have implications for the translational efficiency measurements. Therefore, it would be helpful to see a validation of one or more of the candidate for the translational regulation of genes using orthogonal assays (de novo translation proteomics) or at least a Western blot to show that protein levels are affected. Also ARID5A is mainly in the nucleus, as per their microscopy imaging, how do the authors envision the mechanism of translational regulation?

Inconsistencies in eCLIP Data Interpretation
• The rationale for performing eCLIP in non-microglial cells (e.g., THP-1) is unclear given the microglial focus. A justification or data from microglia-derived cells would strengthen relevance.
• Supplementary Figure 2A: The analysis does not convincingly account for cell-type-specific transcriptomes. ENCODE libraries derived from cancer lines may not represent macrophage/microglia expression profiles, complicating interpretation of enrichment patterns. Specifically, if THP-1 macrophages just express more immune-associated transcripts compared the K563/HepG2 cells then detecting it more in the ARID5A eCLIP library would not be too surprising however not really

supporting their argument.

Key Functional Readouts and Missing Assays

• The authors claim that ARID5 regulates splicing and translation of genes critical for microglial function. However, the microglia functional assays used by themselves—cytokine secretion, lysosomal activity, and iron accumulation—do not fully support this. Key functional outputs like motility or process dynamics are missing and should be included.

• The potential separation of RNA- and DNA-binding roles is intriguing but requires direct testing (e.g., RNA-binding mutant assays or functional data from orthogonal data).

Unconvincing Evidence in Some Key Figures

• Several figures require either replication, better controls, or reinterpretation:

o Figure 2d: The authors should include an IgG control for their IP+WB experiments to show specific enrichment, as it is somewhat surprising that while ARID5A was not notably enriched proteins such as SRSF7 and MAGOH were much stronger enriched. An IgG control would be required here to ensure that these proteins do not unspecifically bind to IgG or the beads. How can these stoichiometric differences be explained? Were the IPs RNase treated or not?

o Supplementary Figure 1e: The biotinylation assay is not of very high quality. No replicate data are provided and no western blot. At the moment, the data are not very convincing evidence of RNA binding and it would be recommended to either remove this figure and statement from the paper or prepare replicate data with Western blot controlling for the IP efficiency.

o Figure 3F: It is very hard to see the differences on the gel, as both of the bands appear to change between conditions. Could the authors represent the individual clones separately in their quantification of the gel and then perform statistical testing to ensure the same number of replicates is being compared? Overall, the effect appears very mild how does this compare to the RNAseq (PSI) results from Figure 3d, where GBA splicing appears to be one of the top hits when knocking down ARID5A. Are all of the changes expected to be this mild? Would this explain very little overlap with alternative translational output?

o Figure 3i: Predicted truncation should be validated (e.g., WB or proteomics).

o Figure 4G-H: The authors propose ISGylation involvement solely on overlapping datasets without sufficient experimental backing; statistical analysis and functional validation are needed.

o Figure 4I: Cytokine secretion was assessed via cytokine array, which is semi-quantitative and depends on internal standards. For reliable data, key cytokines such as IL-1β and TNF-α should be validated by ELISA.

o Figure S5c: No clear effect on phagocytosis or degradation is shown. The claim of neuroprotection is not corroborated here. How do the authors rationalize that?

o Figure 6e: The cells appear apoptotic. These data should be interpreted with caution and ideally validated by a dedicated apoptosis assay.

o Figure 6i: The absence of LysoTracker signal in NT groups is unlikely, given microglia's phagocytic nature even in the T66M line.

o Figure 6k: Data imply that T66M_NT cells lack F-actin, which is implausible. An additional WB should be performed here to validate this or staining needs to be questioned.

Clarity for Reproducibility

• A clear description of cell types used in each experiment is missing in several figure legends and the main text, impairing interpretation of genotype-dependent effects.

• Critical experimental details are missing:

o Concentration of Aβ used.

o QC of Aβ aggregates (e.g., EM or Thioflavin T).

o Normalization approaches (e.g., in viability assays).

For example: Figure 6b: Proper comparison should be A ≤-treated vs. untreated within each genotype. This is not evident in the current graph.

o Indication of biological replicate number and presentation of individual data points across figures.

For example: The sample size in Figures 6a and 6c appears limited (possibly only n=2). I would ask the authors to show individual data points across all graphs for transparency.

Overarching Narrative and Thematic Cohesion

• The manuscript presents multiple disconnected hypotheses (lysosomal function, ferroptosis, ISGylation, etc.) without clear experimental resolution.

o Stronger integration and prioritization of mechanistic themes are necessary to clarify ARID5A's core function.

o Each line of speculation should be explicitly linked to testable hypotheses and validated findings, or the speculation should be tempered accordingly.

Minor Points

1. Literature Representation and Citations

o The abstract overstates the novelty of CNS-specific RBP functon("remain uncharacterized") and should be revised to acknowledge the growing body of literature.

2. Figure-Specific Issues

o Supplementary Figure 1c: The figure legend lacks clarity, particularly regarding ARID5A's apparent significance in only one replicate of the Olah_activation dataset.

o Supplementary Figure 4F: Show replicate data and quantification to support the reported findings.

3. Technical and Presentation Corrections

o Figure 6f–i: The method switches from flow cytometry (Fig. 6c) to Incucyte without explanation. NT groups consistently

have fewer cells, and since data are shown as RCU/µm2 (incorrectly labeled as "µM"), it likely is not normalized to cell count.
o Figure 6I: Cytokine levels should be reported in pg/mL for standardization.
o Terminology: Clarify use of "sg2" and "sg3" for shRNA knockdowns to avoid confusion with CRISPR nomenclature.

Reviewer #2

(Remarks to the Author)
This manuscript (NCOMMS-25-29083-T) demonstrated the machinery of Arid5a in microglia activation as DNA/RNA binding protein. The authors clarified that Arid5a has both activities to bind RNA and DNA directly and indirectly in microglia, although a distinct repertoire, and they suggested the potential role in splicing. In the last part, the authors identified the function of Arid5a by knockdown in microglia, and they demonstrated that knockdown of Arid5a has several functions to have regulation of splicing of neurodegenerative proteins and the alteration of Aβ-induced GCase activity and lysosome response in microglia. In addition, knockdown of Arid5a in microglia augmented the immune responses. Next, knockdown of Arid5a decreased ferroptosis by inhibition of lipid peroxidation in migroglia, indicating protective function. Last, under Aβ stress, depletion of Arid5a also exerts the protective function in TREM-T66M mutant microglia which impaired in phagocytosis and neuroinflammation. However, there are many concerns to be link the machinery of Arid5a function as RNA/DNA binding protein and the phenotypes of Arid5a knockdown microglia.

Reviewer #3

(Remarks to the Author)
In this manuscript, Schafer et al characterize the role of ARID5A in microglia. ARID5A is an RNA-binding protein that can regulate gene expression in multiple ways. Here, the authors show that ARID5A binds RNA, DNA, and proteins and regulates the expression of numerous immune genes. Knockdown of ARID5A in iPSC-derived microglia leads to altered microglia lysosomal functions, altering their engulfment capacity and stress response. Finally, ARID5A can attenuate the phenotypes observed in the TREM2 mutant line.

The regulation of microglia transcriptional signature is central to their capacity to adapt to the brain and carry out their functions rapidly. Microglia represent 5-10% of all brain cells, so cell type-specific analysis has been extremely challenging to carry out, and little is known about the regulation of gene expression in a cell type-specific manner and the regulation of defined microglia states in disease. Therefore, this manuscript is important, and few other groups have tried to tackle this question before. Moreover, the authors aim to bridge the gap between the transcriptional signature and microglia functions, which is essential to evaluate the implications of ARDI5A. This manuscript is unique and important to the field, but some questions must be answered before publication.

• Identification of ARID5A: The authors perform extensive analysis to pinpoint the importance and specificity of ARID5A; however, they do not provide details about their analyses. A supplementary table with more information on Figures 1A and B is necessary to understand the importance of ARID5A among the other RBPs.

• Specificity of ARID5A targets in microglia: ARID5A is a known regulator of gene expression in peripheral immune cells. Moreover, ARID5A is observed in the cortical organoid (Fig. 1D). The authors claim that ARID5A is linked to specific microglia targets involved in disease. A comparison with a known ARID5A binding partner observed in peripheral immune cells and neuronal cells is essential to support this claim.

• Functional assays: While the reviewer appreciates the challenge of generating functional validation, some assays and statistical measures are below standards in the field. All experiments should be done in triplicate at least; individual data points should be shown, and similar quantification should be done across assays. For example, Fig. 5E and many panels of Fig. 6 only have 2 data points, Fig. 6G doesn’t have an individual datapoint, and phagocytosis readout varies between Fig. 6C and G. Figure 6A should include a loading control.

• The set of data presented includes a role of ARID5A in binding Alzheimer disease proteins (Fig. 2C), a role in the regulation of GBA (Fig. 3D), assays with amyloid, ferroptosis (Fig. 5), and TGFb (Fig. 1K). The picture drawn by the authors is not clear at the moment on the role of ARID5A in neurodegeneration because all these phenotypes could lead to neurodegeneration through diverse mechanisms. These data need to be tied together with a direct causal link. Some possibilities could be finding the initial step mediated by ARID5A and leading to microglia dysfunctions, showing that these mechanisms happen in the same cells, or validating the role of ARID5A in a mouse model in early stages of disease. Also, the ‘neuroprotective potential” of ARID5A and “therapeutic potential” mentioned in the abstract and introduction should be rephrased; these are overstatements.

Version 1:

Reviewer comments:

Reviewer #1

(Remarks to the Author)
The authors have addressed many of the major conceptual and technical issues raised in the previous review. In particular, they have strengthened the validation of their sequencing results and clarified the iron-dependent mechanism underlying ARID5A-mediated ferroptosis. The revised manuscript provides a more coherent narrative that links immune, lysosomal and iron metabolism pathways.

However, some limitations remain. For example, the separation of the RNA- and DNA-binding functions of ARID5A remains largely descriptive. Furthermore, there are still several presentation and data quality issues. For example, all Western blots should include clearly labelled molecular weight markers to enable the correct interpretation of band identity and specificity (see Fig. 2E, S1E, S2H). The quality of the confocal microscopy images (Fig. 2A, 5H, 6M,S4G, S5E, S5H), including the newly added repeats, remains below the standard typically seen in comparable publications for microglia and macrophages. Limited resolution and contrast make subcellular localisation difficult to assess with confidence (Fig. 2A). Furthermore, the cells appear unhealthy in multiple experiments, even under untreated or baseline conditions, raising concerns about culture quality and the potential impact of cellular stress on the reported phenotypes. Improving image quality, including acquiring higher-resolution confocal data with better controls and maintaining healthier baseline cultures, would substantially strengthen the manuscript.

Reviewer #2

(Remarks to the Author)
No more revision.

Reviewer #3

(Remarks to the Author)
The authors have addressed all previously raised comments. The manuscript is now written in a much clearer manner, highlighting the role of ARID5A in lysosome biology and iron metabolism.

Version 2:

Reviewer comments:

Reviewer #1

(Remarks to the Author)
I appreciate the additional clarifications, which help to better clarify the issues raised, as well as the revised figures. I also welcome the provision of high-resolution images to be deposited alongside the publication for future reference.

Open Access This Peer Review File is licensed under a Creative Commons Attribution 4.0 International License, which permits use, sharing, adaptation, distribution and reproduction in any medium or format, as long as you give appropriate credit to the original author(s) and the source, provide a link to the Creative Commons license, and indicate if changes were

made.
In cases where reviewers are anonymous, credit should be given to 'Anonymous Referee' and the source.
The images or other third party material in this Peer Review File are included in the article’s Creative Commons license, unless indicated otherwise in a credit line to the material. If material is not included in the article’s Creative Commons license and your intended use is not permitted by statutory regulation or exceeds the permitted use, you will need to obtain permission directly from the copyright holder.
To view a copy of this license, visit https://creativecommons.org/licenses/by/4.0/

Response to Reviewers
ARID5A RNA-binding Coordinates Microglial Defense and Ferroptosis

Overall Response

We thank the Reviewers for their positive comments that our manuscript "presents an ambitious and multifaceted study", "produced high quality sequencing data sets and proteomics", and is "unique and important to the field". We also acknowledge the Reviewers' appreciation for the importance of our study in our "aim to bridge the gap between the transcriptional signature and microglia functions", an area that "few other groups have tried to tackle". Here, we submit an extensively revised manuscript that takes into consideration the helpful suggestions by the Reviewers as detailed below, and we highlight how we responded to the main critiques regarding i) improved cohesion of diverse themes, ii) enhanced validation of sequencing data with orthogonal assays and lastly, iii) delineated the mechanism by which ARID5A regulates ferroptosis.

i). Improved cohesion of diverse themes: We extensively reshaped the manuscript to position ARID5A RNA-based regulation as a nexus of an interconnected network between immune, lysosomal, and iron metabolism as opposed to a regulator of three distinct pathways. These changes are evident through the text, namely with

a) Title change from " to "Modulation of the DNA/RNA Binding Protein ARID5A Regulates Microglial Activation and Response" to "ARID5A RNA-binding Coordinates Microglial Defense and Ferroptosis",
b) Highlighting the consistency of our results with previous studies through the lens of interconnected studies, as shown in the section "Depletion of ARID5A leads to enhanced translation of the defense response and increased ISGylation and cytokine secretion" in which our lysosomal dampening with increased cytokine secretion was consistent with a prior study in cytokine-stimulated macrophages.
c) Additional analyses connecting iron, immune, and lysosome pathways presented in **Figure 7** and **Supplemental Figure 8**

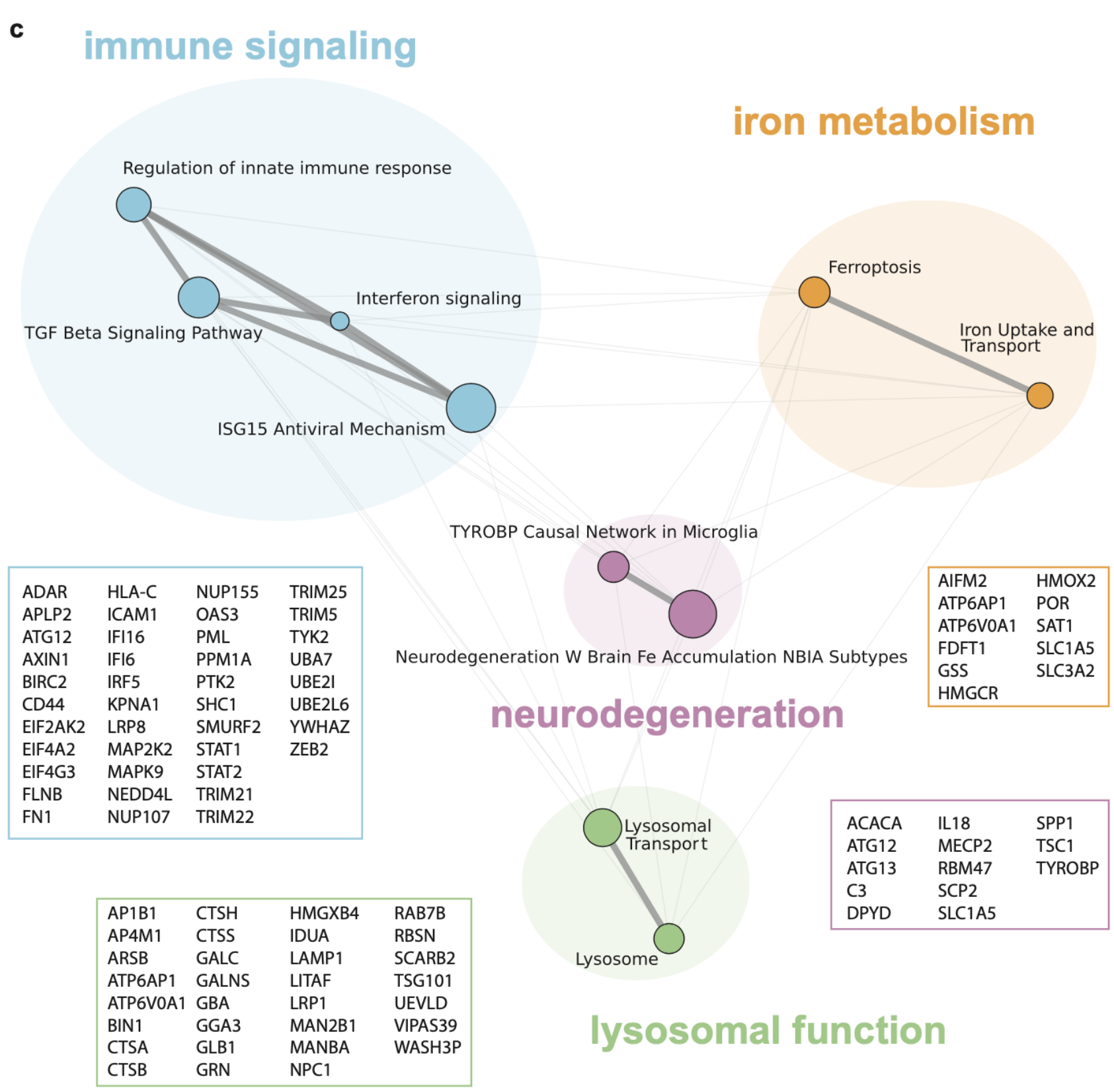

Figure 7c: Integration of immune signaling, iron metabolism, and lysosomal function with neurodegeneration. Boxed genes are regulated at the RNA level by ARID5A.

ii) Enhanced reproducibility and validation of sequencing data with orthogonal assays: We performed additional experiments to reproduce our findings as well strengthen findings from our sequencing data. These are detailed point by point below, but in summary:

a) We repeated the requested experiments (detailed below) to add replicates and enhance reproducibility. These are present throughout the manuscript, but are most evident in **Figure 5**.

b) We performed several Western blotting experiments as orthogonal validation of sequencing data/computational predictions as shown by validated GBA expression in **Supplemental Figure 6c-d** and ISGylation in **Figure 4i** and **Supplemental Figure 7f**. These are discussed more in depth in our point by point response below.

iii) Delineated the mechanism by which ARID5A regulates ferroptosis: We overexpressed ARID5A and induced ferroptosis in the presence of iron and ferroptosis inhibitors. As shown in **Figure 5j-k** and **Supplemental Figure 8b-c**, ARID5A regulation of ferroptosis is iron dependent and ameliorated with iron chelation.

Response to Reviewer #1 (Remarks to the Author):

The manuscript by Schafer et al presents an ambitious and multifaceted study exploring the RNA-binding protein ARID5A and its role in microglial function, combining eCLIP, RNA sequencing, ribosome profiling, protein interaction mapping with functional assays in microglia. The findings are of potential significance, particularly given the emerging interest in RBPs in their diverse regulatory roles and cell-type specific contexts.

The authors have produced high quality sequencing data sets and proteomics but several methodological, interpretational, and presentation issues limit the current impact of the study. I would strongly encourage the authors to focus on clarifying their mechanistic claims, and strengthen the narrative thread across the study. With these revisions, the manuscript would constitute a valuable contribution to the field and has the potential to advance understanding of a cell type-specific RBP in neuroimmune contexts. However, it currently lacks critical validation and cohesion. Below are major and minor points organized to guide revision.

We thank the reviewer for the helpful suggestions for our revision. We have focused on validation and cohesion for our revision as described in our overall response above and as detailed in our point by point response below.

Comment 1

The manuscript proposes several distinct functions for ARID5A (e.g., in lysosomal regulation, splicing, ferroptosis, and immune modulation) without adequately demonstrating causality for all of these functions. It appears that this protein has three very distinct modes of action (as an DNA or RNA-binding proteins active in the nuleus and cytoplasm). While this is not unknown for RBPs, it is not clear what specific feature qualifies the protein to be involved in all of these processes (just location, modification, solely interactors?).

Response 1

We thank the reviewer for this feedback and accordingly prioritized presenting ARID5A as a regulator of an integrated network rather than distinct networks, as described in our Overall Response. The reviewer raises an interesting question that is pertinent to the RBP field as a whole: what specific feature qualifies some RBPs (like ARID5A) to be more broadly involved? Consistent with other RBPs, there is not one specific feature that qualifies ARID5A to be involved in these processes. RBP activity is not static, and changes in localization and modification can influence their binding, as shown with stress granule proteins[1]. Further, many RBPs are multimodal, including the stress granule protein FUS that regulates translation as well as splicing. In addition to RNA regulation, FUS regulates DNA[2] as do several zinc finger RBPs[3]. Zinc fingers, FUS, and ARID5A differ greatly in domain and structure, which suggests that this specific feature is not likely to be due to protein structure. One potential feature is that ARID5A, like FUS, is not restricted to either the nucleus or the cytoplasm. We demonstrated nuclear and cytoplasmic expression of ARID5A (Supplemental Figure 4g-i), and this was consistent with other reports of ARID5A shuttling between the nucleus and cytoplasm to interact with UPF1[4]. Overall, our findings for ARID5A were in agreement with its unrestricted localization, previous reports, and findings for other RBPs that broadly regulate RNA through multiple mechanisms in addition to DNA.

Comment 2

Rescue experiments are essential across relevant functional assays to distinguish ARID5A-specific effects from off-target phenomena.

Response 2

We thank the reviewer for this comment and recognize the strength of delineating ARID5A regulation from off-target phenomena. To address this concern, we included the following appropriate controls:

i) Nontargeting CRISPRi guide compared to two independent ARID5A guide RNAs (sg2 and sg3). The cloning and selection of guide sequences was consistent with previous studies leveraging CRISPRi-mediated depletion[5–7]. All functional assays for iTF microglia were compared against the nontargeting control to control for any influence of CRISPRi machinery/introduction of guides as shown in **Figures 3-5** and **Supplemental Figures 5-8.**

ii) Transfection of ARID5A full-length and domain deleted constructs in HEK293T and THP-1 cells. We designed these constructs to contain a V5 in order to avoid biases with the ARID5A antibody for the ARID domain, as evident in **Supplemental Figure 2**

iii) Mock transfection of HeLa cells to control for the effects of Lipofectamine as shown in **Figure 5** and **Supplemental Figures 7-8**.

iv) Isogenic control compared to T66M mutation. In order to control for genetic background, we used T66M mutant lines generated by the iNDi consortium to eliminate off-target effects. Both of these lines are on the KOLF 2.1 background.

v) Nontargeting shRNA compared to ARID5A-targeted shRNA in iMGs. To control for effects of shRNAs, we included a nontargeting control and performed all functional studies against this control in **Figure 6** and **Supplemental Figure 9**.

To provide further evidence, we attempted to overexpress ARID5A in the iTF microglia as a "rescue" experiment. However, these experiments resulted in rampant cell death. We tried multiple approaches, including RNA-mediated overexpression (Horizon Biosciences CRISPRa-VPR mRNA co-delivery with sgRNA) as well as conventional plasmid overexpression at different timecourses. Microglia are well-known to be resistant to introducing overexpression systems[8,9], and this was no different in our hands. Beyond experimental challenges, overexpression of ARID5A can be disastrous for the cells, as elevated ARID5A has been implicated in severe inflammation such as sepsis and EAE[10,11]. With those considerations in mind, we recognized the importance of this question, so as an alternative we provided additional data in HeLa cells with ARID5A overexpression, as these immortalized cell lines are more resistant. This data supported both the ISGylation findings as well as iron metabolism findings. As an additional line of supporting evidence, the knockdown in the T66M iMGs was performed using shRNA rather than CRISPRi and recapitulated many of our findings in iTF microglia. In total, our interrogation of ARID5A functions was robust as many of these functions were replicated across two different knockdown modalities in two separate microglia models as well as an overexpression model in an immortalized cell line.

Comment 3

Figure 3: For example, considering that nearly 9000 mRNAs were bound and 300 mRNAs appear to be dependent on ARID5A expression, the rationale for narrowing down on lysosomal function does not appear very straight-forward. If the ARID5A-mediated GBA splicing is indeed causing differential lysosomal activity, a rescue by providing an intact GBA version should be provided. This would be a necessary experiment to confirm that GBA is the key gene here.

Response 3

We thank the reviewer for this suggestion and recognize the need for clarification. In revision of our manuscript we identified an inconsistency with the file to generate Figure 3d vs Figure 3b and c, which has since been corrected. The rationale to focus on the lysosome is motivated first and foremost by the splicing event results. In Figure 3c, lysosome related pathways were enriched for direct (bound in eCLIP) skipped exon events. While pathways like chromatin modifying enzyme were also enriched, we probed lysosomal function because it is of stronger biological interest in regards to microglial function. We focused on GBA over other lysosomal genes as it has one of the largest changes in delta percent spliced in for ARID5A knockdown (**Figure 3d**). In our revision, we provide an additional GBA WB to show

the canonical GBA is still expressed (**Supplemental Figure 6c-d**). Our data suggest translation of GBA may be less efficient. In this case, reintroducing intact GBA would not be appropriate as these are not a complete loss of GBA, but rather a significant percentage of GBA transcripts contain exon 2 and result in a noncoding variant, as predicted by Ensembl. We highlight lysosome function due to the importance of GBA for the lysosome in the literature, but modify our language to clarify that other lysosomal genes are likely involved.

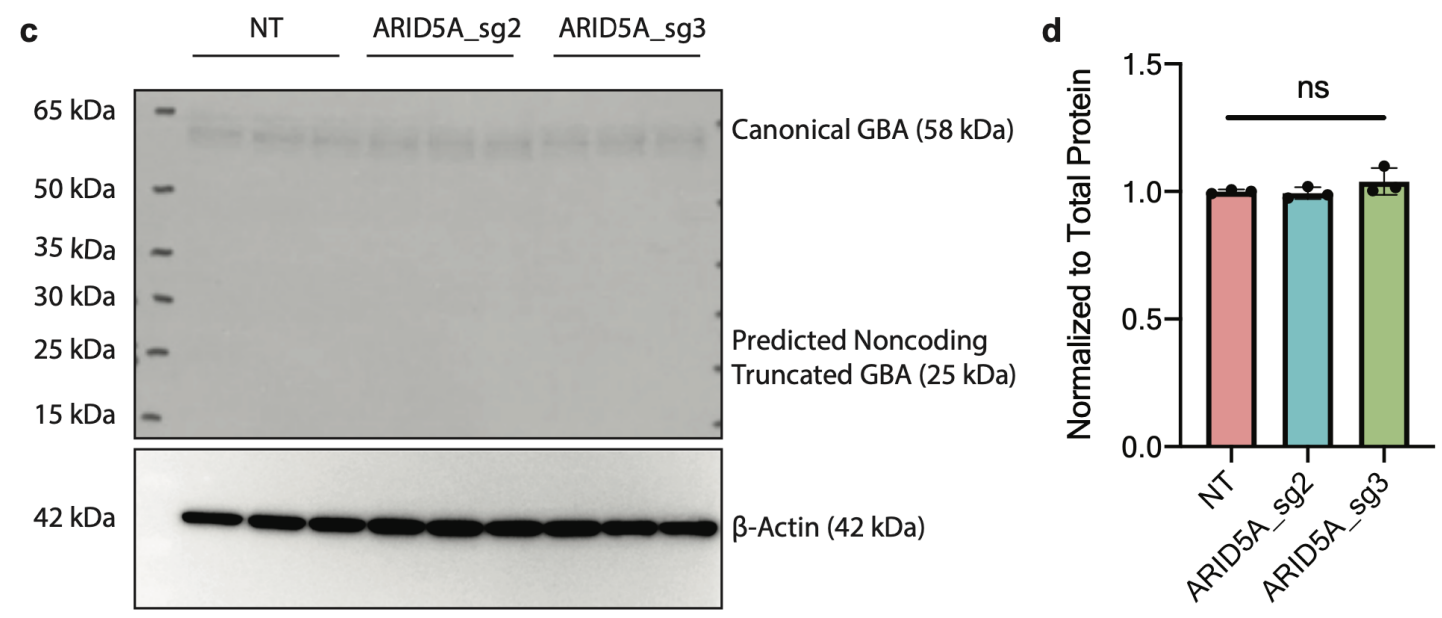

Supplemental Figure 6c-d: Consistent with Ensembl prediction that the truncated version was noncoding, canonical GBA antibodies detect only the full length variant

Comment 4

In addition, one should consider generating RNA or DNA-binding mutants that can disentangle the different roles. Otherwise, the paper remains somewhat descriptive. Figure 1: For example, can the authors see differences in iron metabolism when they generate RNA-binding deficient mutants.

Response 4

We thank the reviewer for this suggestion and provide additional experiments and analysis to alter the ARID5A protein structure (**Supplemental Figure 2)**. The ARID5A protein is composed of the ARID domain and IDR, the latter of which has high affinity for nucleic acids. While a discrete RNA vs DNA binding mutant is unlikely, we probed the effect of the ARID domain on RNA binding. RNA binding persisted regardless of the ARID domain, which was in line with a prior study[12]. Further, domain deletion resulted in increased RNA binding, in agreement with IDR affinity for nucleic acids. Our RIP-seq demonstrated changes in RNA-bound targets upon domain deletion, so while mutating ARID5A can result in binding targets, it is unlikely to ablate RNA binding completely due increased disordered regions that bind RNA with high affinity.

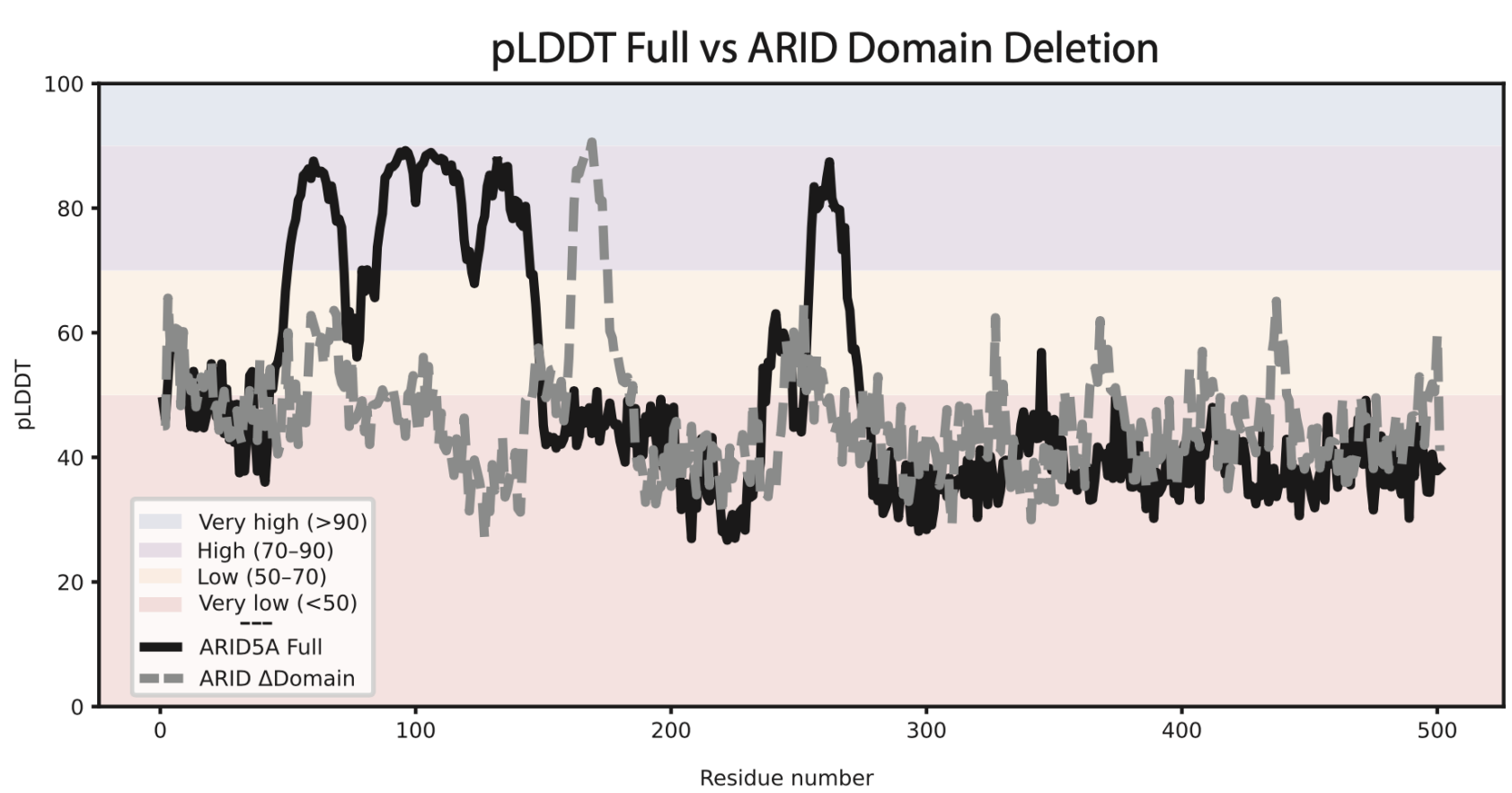

Supplemental Figure 2f: pLDDT scores calculated by Alphafold2 demonstrate increased disorder (lower pLDDT) upon ARID domain deletion

Comment 5

Figure 4: Given that the effect on mRNA splicing was comparatively mild (see comment below on Figure 3F), it might have implications for the translational efficiency measurements. Therefore, it would be helpful to see a validation of one or more of the candidate for the translational regulation of genes using orthogonal assays (de novo translation proteomics) or at least a Western blot to show that protein levels are affected.

Response 5

We thank the reviewer for this suggestion and added a Western blot as validation to show total ISGylation levels and free ISG15 (a key translational target) were affected by ARID5A knockdown (**Figure 4i**). We further validated this in an ARID5A overexpression model in HeLa cells (**Supplemental Figure 7f**).

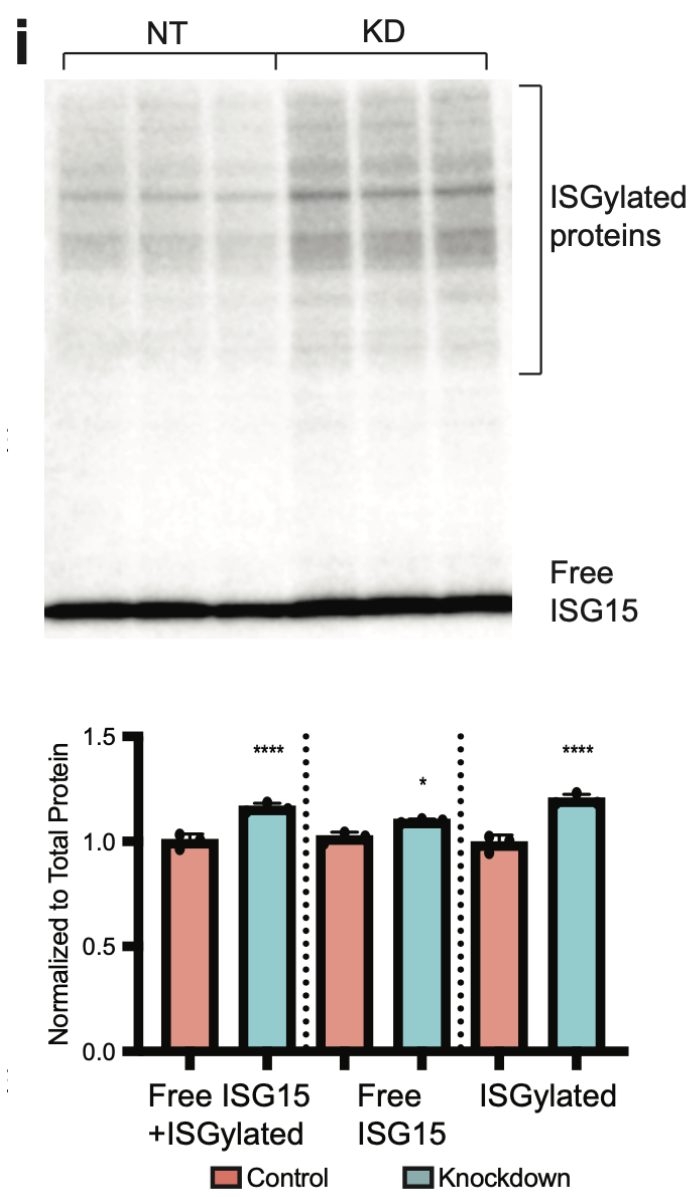

Left: **Figure 4i**: Validation of ISG15 enhanced translation and ISGylation in microglia

Right: **Supplemental Figure 7f:** Validation of enhanced ISGylation in HeLa cells

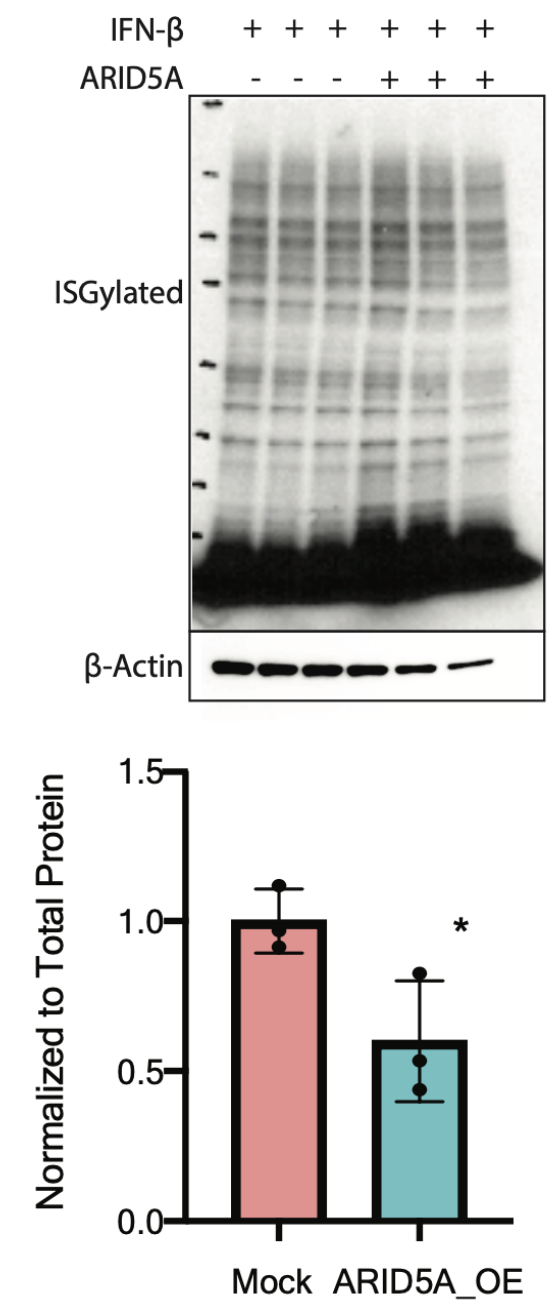

Comment 6

Also ARID5A is mainly in the nucleus, as per their microscopy imaging, how do the authors envision the mechanism of translational regulation?

Response 6

The reviewer is correct to point out that the quality of our images suggested ARID5A was mainly in the nucleus. To address this concern, we provided higher resolution images and quantification (**Figure 2a** and **Supplemental Figure 4g-i**) to show that while ARID5A is highly expressed in the nucleus, it is also expressed in the cytoplasm. Cytoplasmic expression supports a ARID5A involvement in translation, and was reinforced by its PPIs with translation-associated RBPs (**Figure 2d**, **Figure 4a**, **Supplemental Figure 7c**) and prior literature demonstrating ARID5A regulates IL17 translation[13] and interacts with UPF1 in the cytoplasm to regulate proteasome degradation[4].

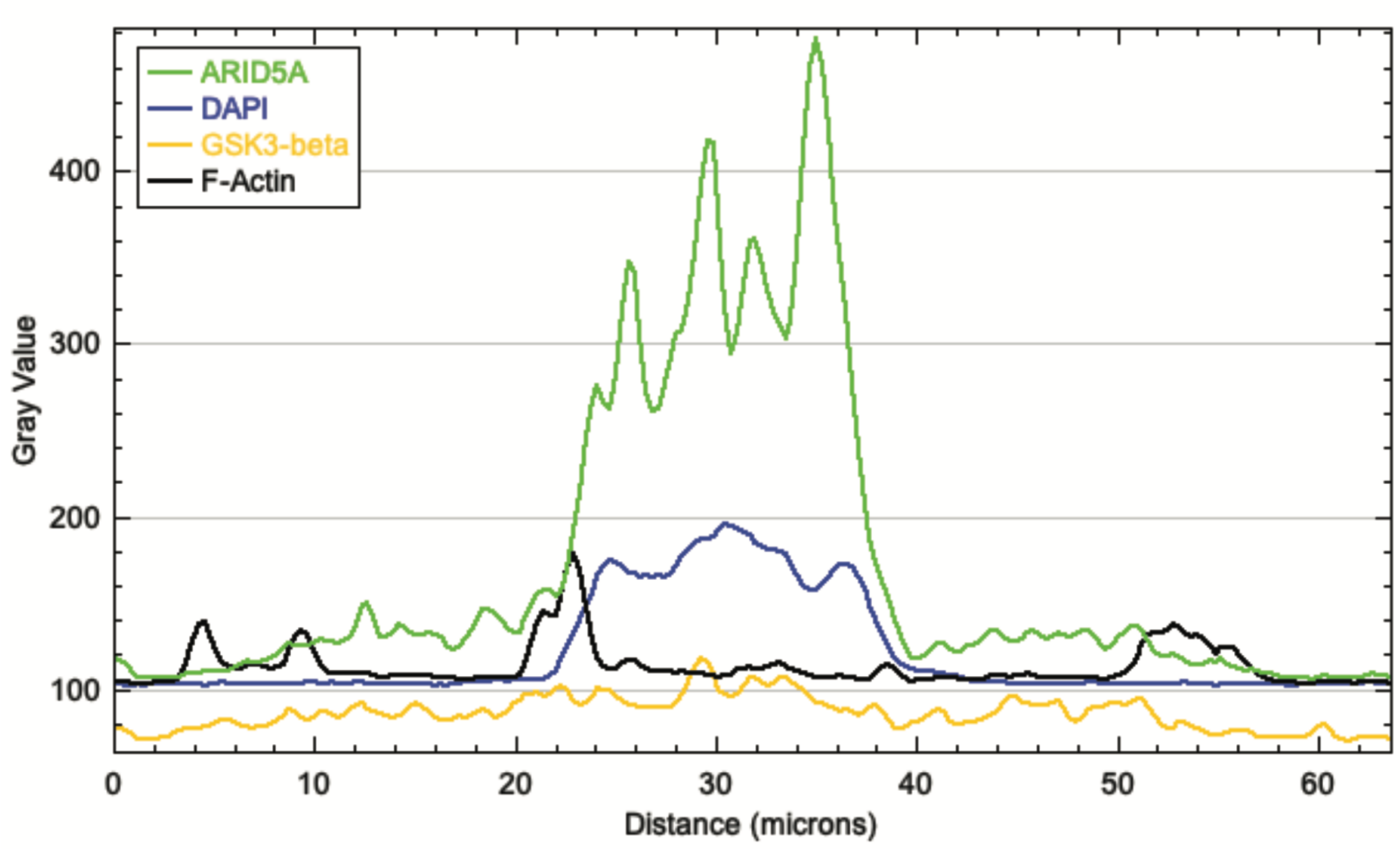

Supplemental Figure 4i: ARID5A is highly expressed in the nucleus but also cytoplasmic when compared to DAPI signal

Comment 7

The rationale for performing eCLIP in non-microglial cells (e.g., THP-1) is unclear given the microglial focus. A justification or data from microglia-derived cells would strengthen relevance.

Response 7

The reviewer is correct to point out the strength of performing eCLIP in microglia and thank them for the opportunity to clarify our rationale. Microglia and macrophages are both myeloid cells and as a result microglia share more similarity with macrophages than other CNS cell types. In **Figure 1**, RBPs, including ARID5A were classified by the Human Protein Atlas in Microglia/Macrophages due to their shared lineage. It is true that naive THP-1s are more monocyte-like, however we differentiate THP-1s to macrophages using well-established protocols[14]. To clarify THP-1s were differentiated, we included the following in the main text:

"we performed an immunoprecipitation assay in THP-1 cells differentiated to macrophages"

Similar to how neuronal studies use SY5Y cells in conjunction with iPSC-derived neurons, we used an immortalized cell line model that closely captures microglial genes to model protein-RNA interactions. Many of these studies such as eCLIP require tens of millions of cells and this grows exponentially when working with small cells such as iPSC-derived microglia. To address the relevancy to microglia, we validated ARID5A-RNA interactions mapped in THP-1 macrophages in iTF microglia by examining two different aspects of RNA metabolism (splicing and translation) and overlapping bound targets and differentially spliced/translated targets.

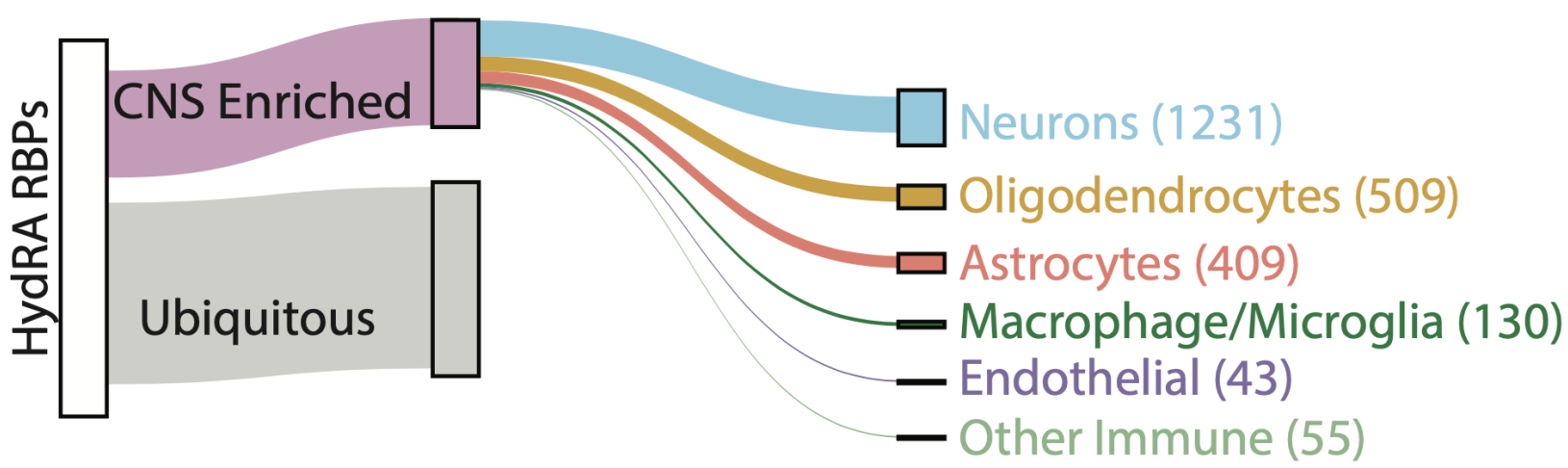

Figure 1a: Microglia and Macrophages are classified together by HPA due to their shared myeloid lineage

Comment 8

Supplementary Figure 2A: The analysis does not convincingly account for cell-type-specific transcriptomes. ENCODE libraries derived from cancer lines may not represent macrophage/microglia expression profiles, complicating interpretation of enrichment patterns. Specifically, if THP-1 macrophages just express more immune-associated transcripts compared the K563/HepG2 cells then detecting it more in the ARID5A eCLIP library would not be too surprising however not really supporting their argument.

Response 8

The reviewer is correct to point this out and as such we have amended our manuscript to reflect this as a benchmark in line with ARID5A's known role in binding immune transcripts. That section of the text has been reduced and now reads as:

"Skipper-defined[24] ARID5A binding targets were enriched in immune-associated transcripts (Supplemental Figure 2a), consistent with its known RNA binding targets[12–14,21]. "

Comment 9

The authors claim that ARID5 regulates splicing and translation of genes critical for microglial function. However, the microglia functional assays used by themselves—cytokine secretion, lysosomal activity, and iron accumulation—do not fully support this. Key functional outputs like motility or process dynamics are missing and should be included.

Response 9

We thank the reviewer for this comment. We provided additional citations and discussion throughout the text to clarify cytokine secretion, lysosomal activity, and iron accumulation are important microglial functions. To address the point of including motility/process dynamics, we added a migration to amyloid beta experiment in **Supplemental Figure 7j-k t**hat was in agreement with our ribosome profiling data (**Figure 4**) and findings in T66M microglia (**Figure 6**, **Supplemental Figure 9**).

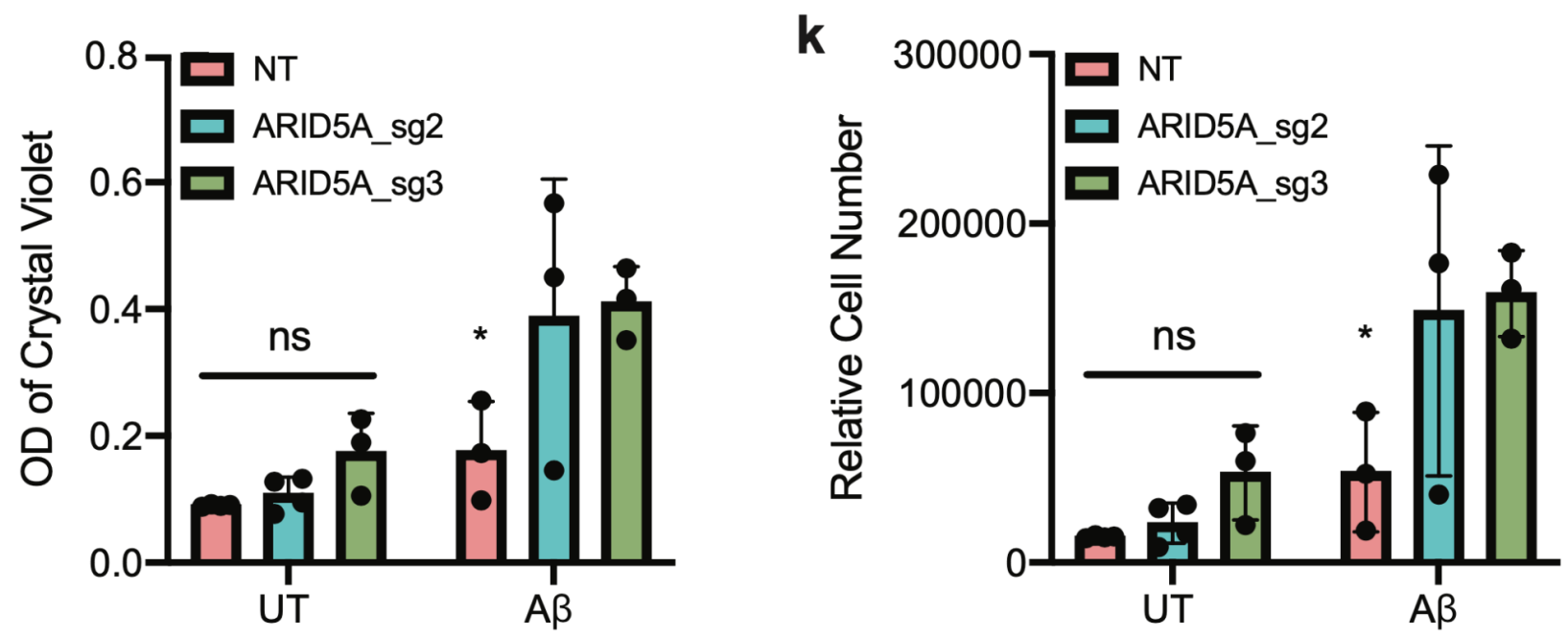

Supplemental Figure 7h-i:
Transwell assay measuring microglial migration

Comment 10
The potential separation of RNA- and DNA-binding roles is intriguing but requires direct testing (e.g., RNA-binding mutant assays or functional data from orthogonal data).

Response 10
We agree with the reviewer that delineating RNA vs DNA binding is intriguing. In our revision, we approached this question by deleting the ARID domain, which is well-known to bind DNA but also implicated in RNA binding. We probed changes in ARID5A protein structure, RNA binding levels, and RNA targets upon ARID domain deletion (**Supplemental Figure 2**). As discussed in **Comment 4**, the increased IDR and use of the domain for DNA and RNA binding hinders the possibility of generating a completely RNA-deficient mutant.

Comment 11
Figure 2d: The authors should include an IgG control for their IP+WB experiments to show specific enrichment, as it is somewhat surprising that while ARID5A was not notably enriched proteins such as SRSF7 and MAGOH were much stronger enriched. An IgG control would be required here to ensure that these proteins do not unspecifically bind to IgG or the beads. How can these stoichiometric differences be explained? Were the IPs RNase treated or not?

Response 11
The reviewer is correct to point out the need for an IgG control and the ambiguity in the experimental conditions. To address these concerns, we performed an additional co-IP experiment with a subset of targets and included an IgG control (**Supplemental Figure 4e**). We clarified in the methods that these lysates were not RNAse treated. Regarding stoichiometric differences, this can be readily explained by differences in expression levels and that these are known to not necessarily be a 1:1 ratio.

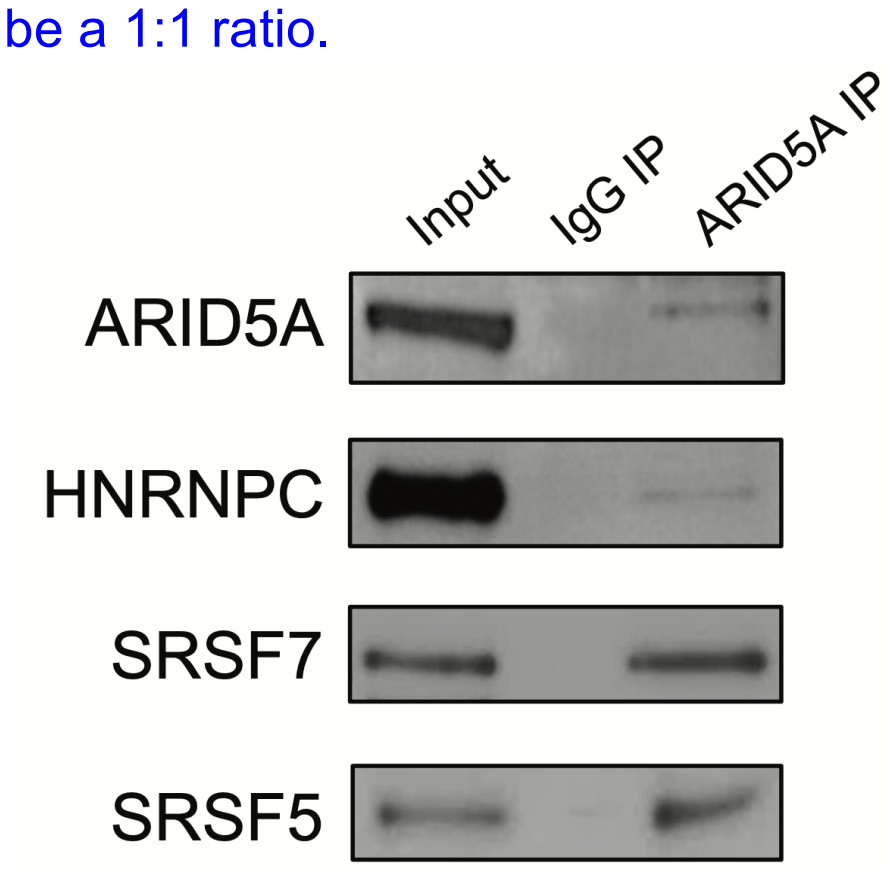

Supplemental Figure 4e:
Inclusion of IgG control in co-immunoprecipitation

Comment 12
Supplementary Figure 1e: The biotinylation assay is not of very high quality. No replicate data are provided and no western blot. At the moment, the data are not very convincing evidence of RNA binding and it would be recommended to either remove this figure and statement from the paper or prepare replicate data with Western blot controlling for the IP efficiency.

Response 12

The reviewer is correct to point out the quality of our initial biotinylation assay, and as a result we provided a higher quality biotin assay with replicates and no crosslinked control (**Supplemental Figure 1e**). This was further validated in the added V5 domain vs no domain experiments in **Supplemental Figure 2** alongside a V5 WB.

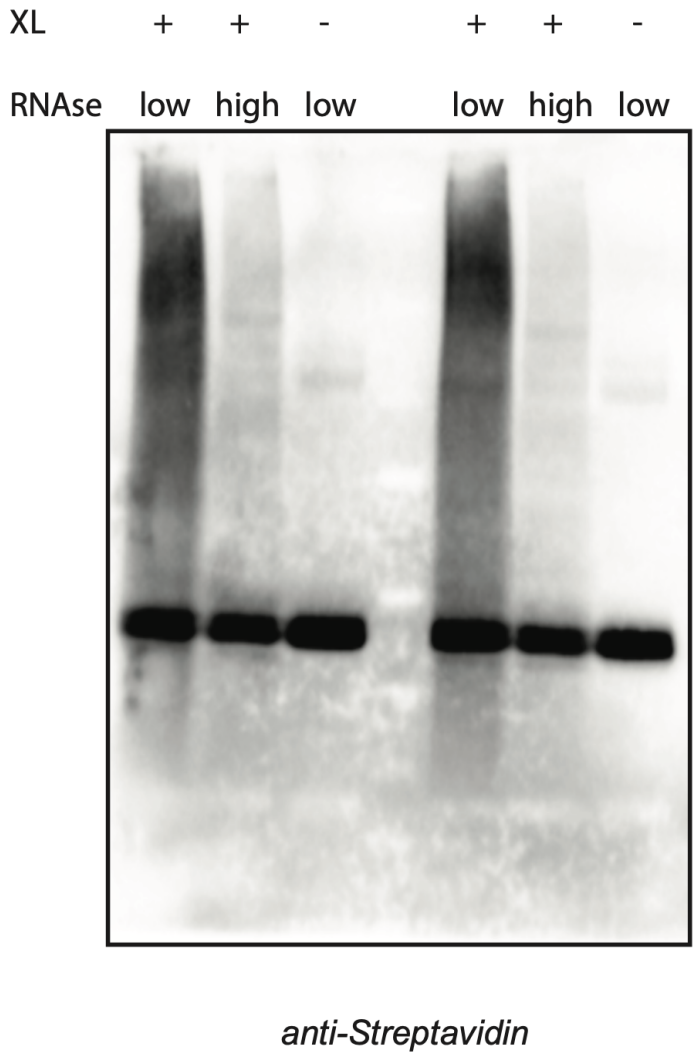

Left: Supplemental Figure 1e: Repeat of biotinylation assay for ARID5A RNA binding

Right: Supplemental Figure 2f: Biotinylation assay with corresponding V5 Western blot.

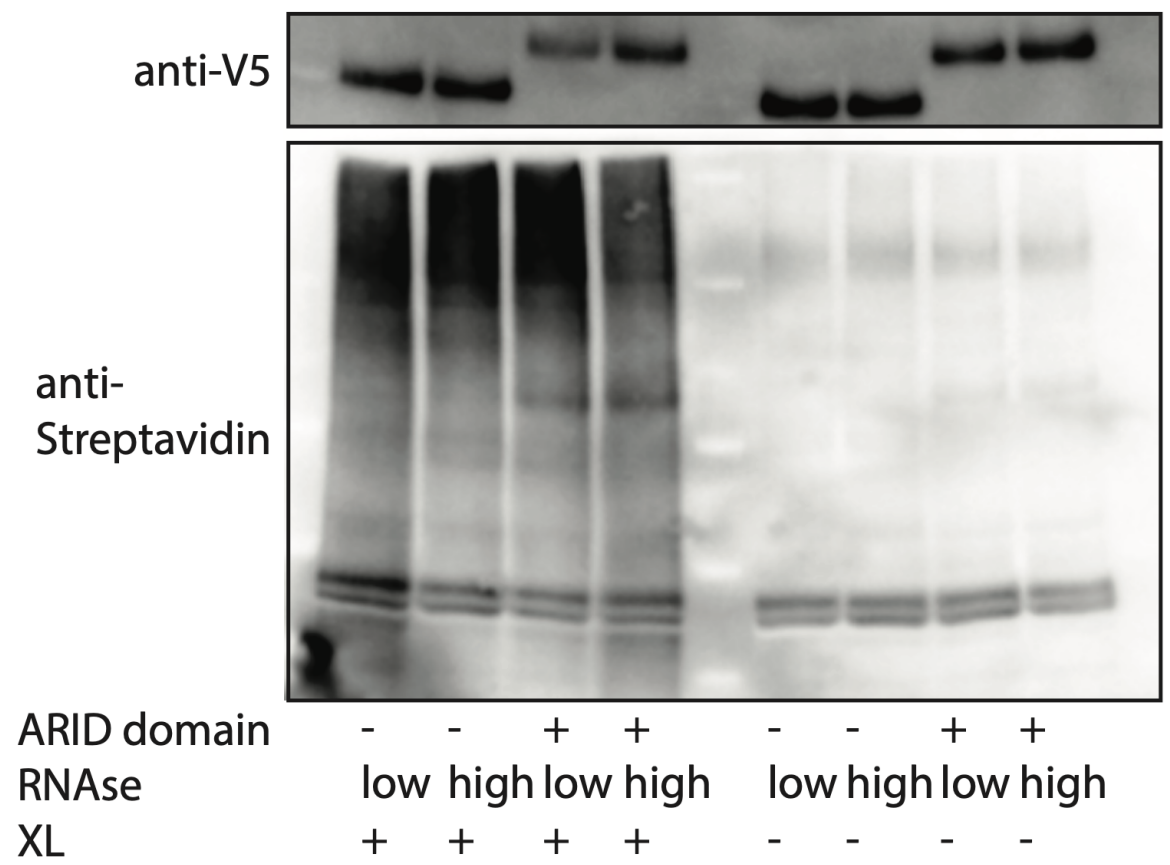

Comment 13

Figure 3F: It is very hard to see the differences on the gel, as both of the bands appear to change between conditions. Could the authors represent the individual clones separately in their quantification of the gel and then perform statistical testing to ensure the same number of replicates is being compared? Overall, the effect appears very mild how does this compare to the RNAseq (PSI) results from Figure 3d, where GBA splicing appears to be one of the top hits when knocking down ARID5A. Are all of the changes expected to be this mild? Would this explain very little overlap with alternative translational output?

Response 13

We thank the reviewer for this suggestion and have amended our quantification to represent the individual guides (**Figure 3g**). In regards to the effect appearing mild, these assays are highly dependent on primer design and thus may not capture the same sensitivity as the RMATs analysis. As an orthogonal validation, we validated changes to exon 2 inclusion using RT-qPCR with a different set of primers (**Figure 3h**). As for the differences with translation, these are connected but not completely linear changes.

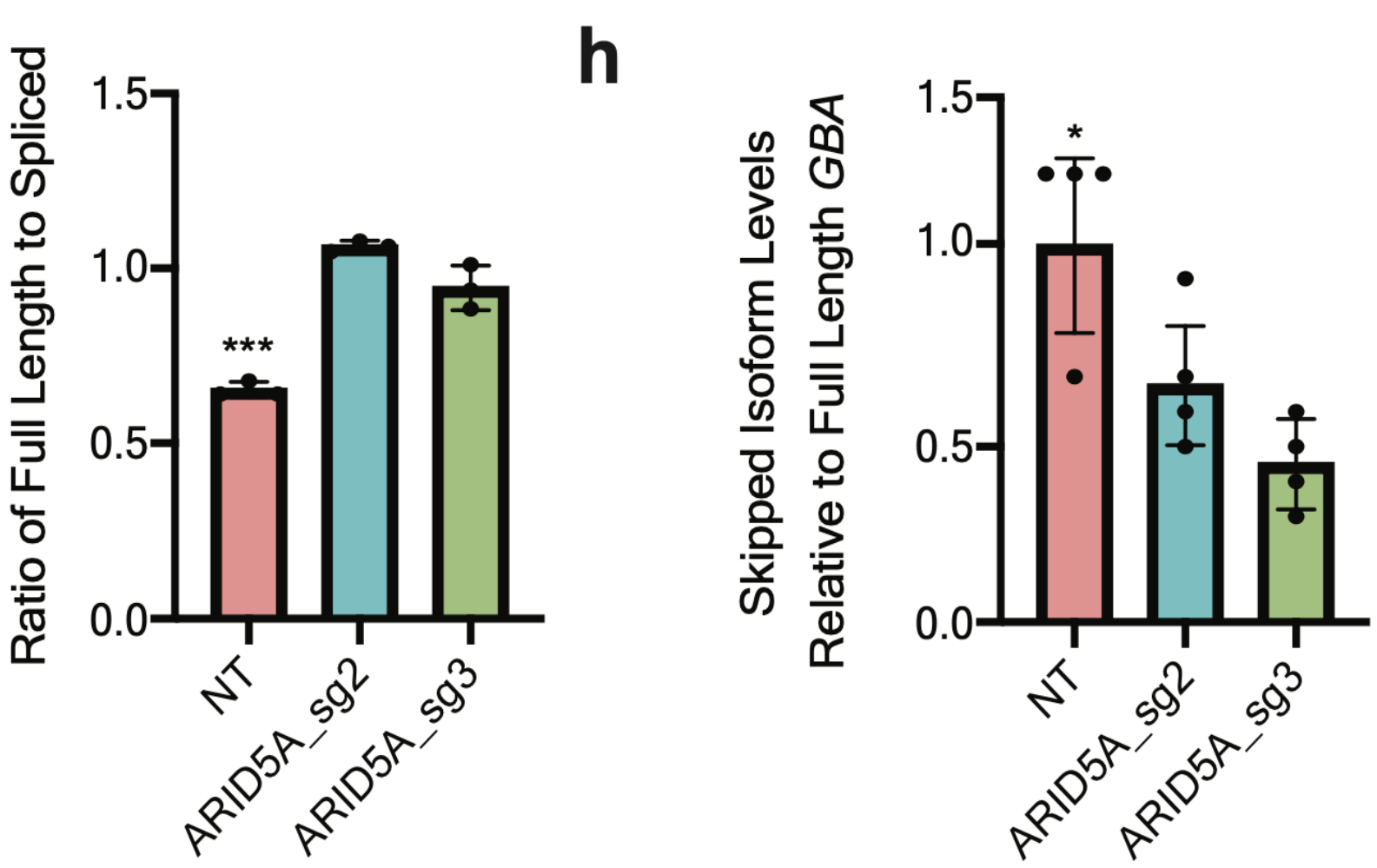

Figure 3g-h: Quantification of splicing gel by individual guides (3g) and secondary validation with RT-PCR (3h)

Comment 14
Figure 3i: Predicted truncation should be validated (e.g., WB or proteomics).

Response 14
We thank the reviewer for this suggestion and amended our text to clarify that this truncated variant is predicted to be noncoding and provided a Western blot validation, as shown in **Comment 3**. The text now reads:
"Predicted by Ensembl to be noncoding, the truncated version was not detected via Western blot with canonical anti-GBA antibodies (Supplemental Figure 6c-d)".

Comment 15
Figure 4G-H: The authors propose ISGylation involvement solely on overlapping datasets without sufficient experimental backing; statistical analysis and functional validation are needed.

Response 15
The reviewer was correct to point out the limitations of our analysis. To address this concern, we performed additional functional validation and analyses. We performed Western blotting and validated ISGylation was upregulated upon ARID5A depletion in microglia (**Figure 4i**) and reproduced these results in HeLa cells (**Supplemental Figure 7f**). The ISGylation blots are shown in **Comment 5**. We provided additional analysis linking ISGylation with lysosomal activity and iron accumulation (**Supplemental Figure 8a**).

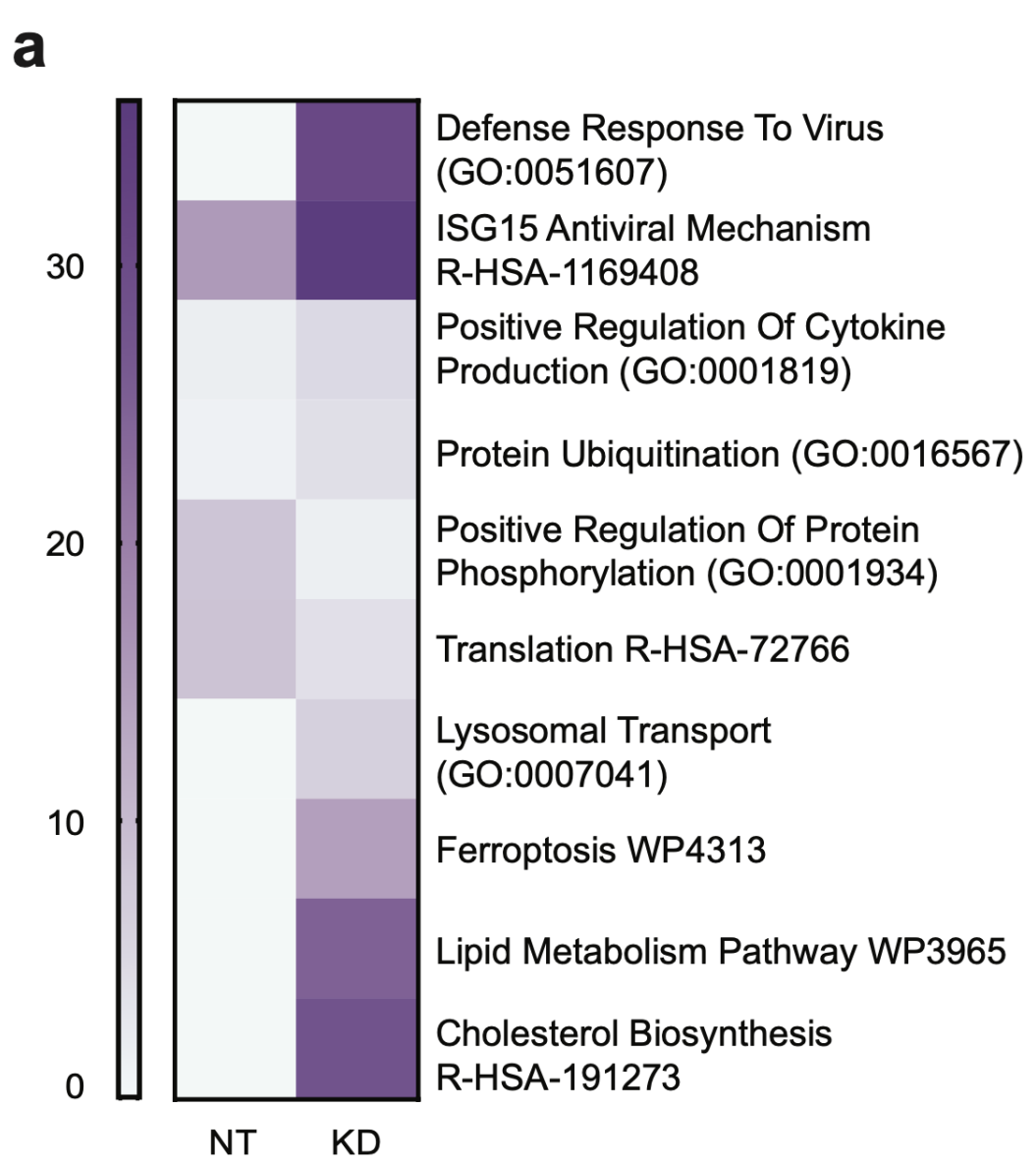

Supplemental Figure 8a: Enrichment of overlapping ARID5A-depleted enhanced TE

Comment 16
Figure 4I: Cytokine secretion was assessed via cytokine array, which is semi-quantitative and depends on internal standards. For reliable data, key cytokines such as IL-1β and TNF-α should be validated by ELISA.

Response 16
We thank the reviewer for this suggestion and accordingly provided ELISA validation in **Supplemental Figure 7i**. To clarify the quantification, samples were normalized to their total protein positive control and reported in ratio-based intensities per manufacturer's instructions.

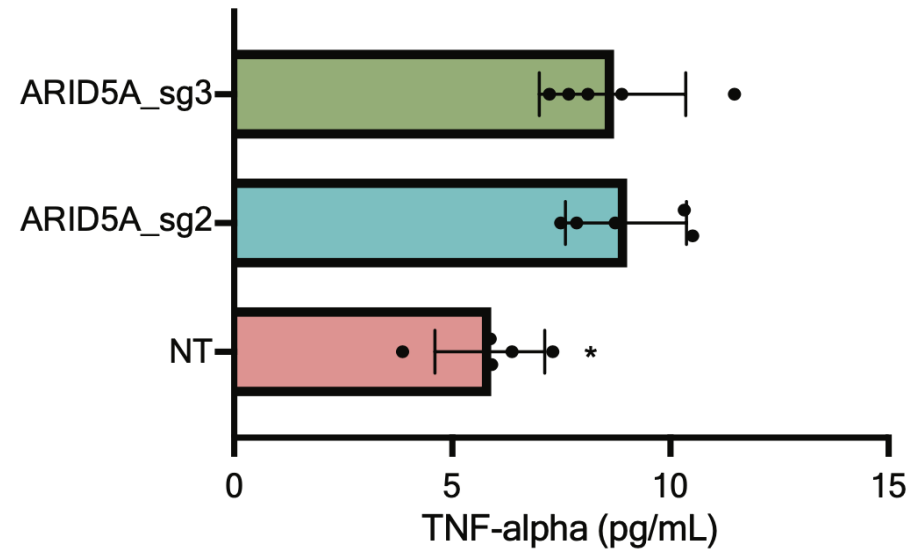

Supplemental Figure 7i: ELISA validation of TNF alpha secretion

Comment 17

Figure S5c: No clear effect on phagocytosis or degradation is shown. The claim of neuroprotection is not corroborated here. How do the authors rationalize that?

Response 17

We thank the reviewer for this comment as we do not wish to overstate our findings. We amended our text to exclude the term "neuroprotection" entirely and confine our results and discussion to our observed results.

Comment 18

Figure 6e: The cells appear apoptotic. These data should be interpreted with caution and ideally validated by a dedicated apoptosis assay.

Response 18

We thank the reviewer for this suggestion and accordingly provided an apoptosis assay (Supplemental Figure 9c, g). For consistency with our other assays, we opted for a dedicated apoptosis dye measuring Caspase 3/7 and quantified with flow cytometry.

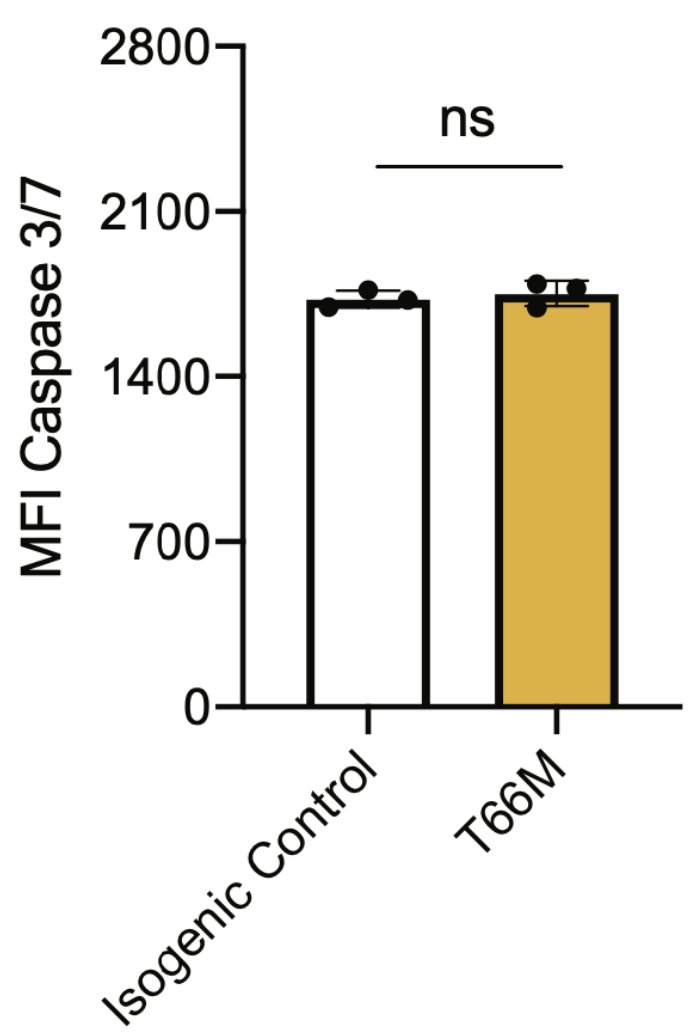

Left: Supplemental Figure 9c: T66M cells are not apoptotic as measured by live cell apoptosis dye (Caspase 3/7)

Right: Supplemental Figure 9g: ARID5A depletion does not lead to significant apoptosis as measured by Caspase 3/7 dye

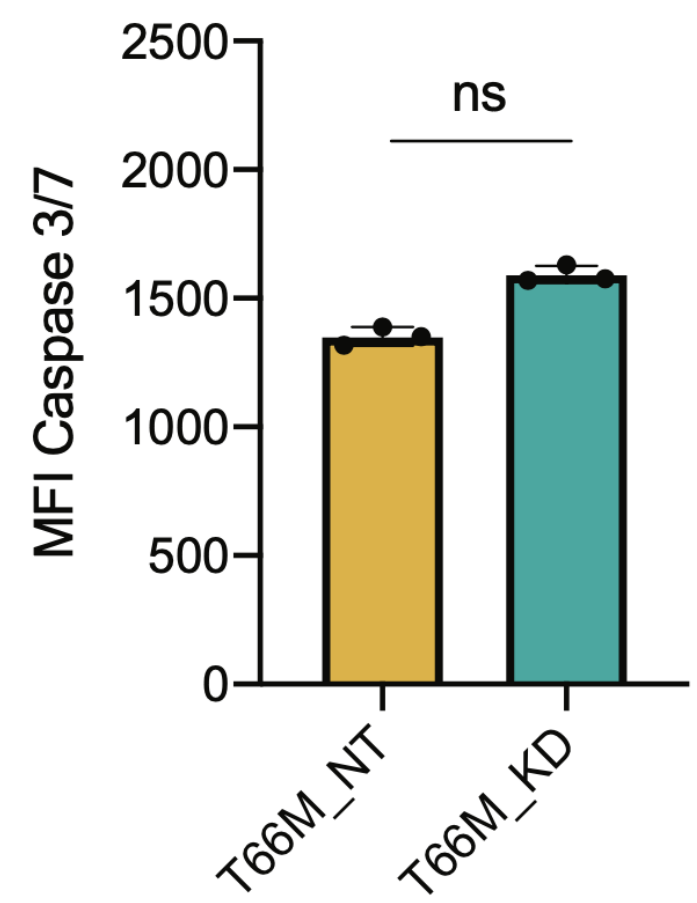

Comment 19

Figure 6i: The absence of LysoTracker signal in NT groups is unlikely, given microglia's phagocytic nature even in the T66M line.

Response 19

The reviewer is correct to point out complete absence is unlikely. To address this we repeated this experiment with flow cytometry, which is more sensitive than the Incucyte. We detected a reduced, but clear Lysotracker signal (Figure 6o). As further validation, we also included DQ-BSA and observed the same trend (Figure 6n).

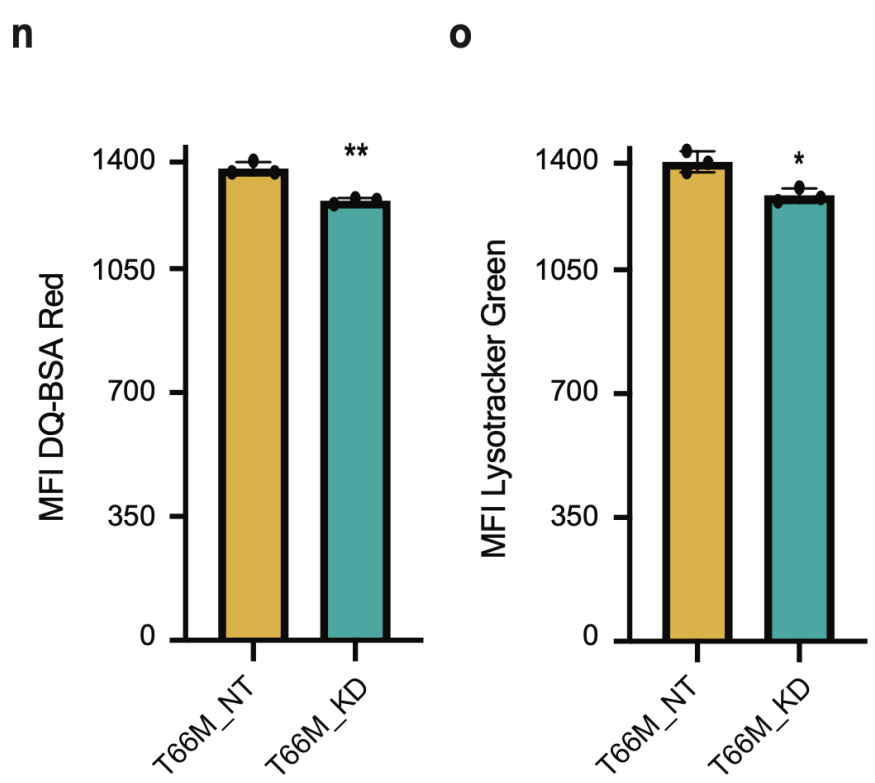

Figure 6n-o:Lysotracker and DQ-BSA readouts validating ARID5A-depletion effects on lysosomal activity

Comment 20

o Figure 6k: Data imply that T66M_NT cells lack F-actin, which is implausible. An additional WB should be performed here to validate this or staining needs to be questioned.

Response 20

We thank the reviewer for this comment and agree. In our revision we decided this data was confusing and tangential at best to our manuscript. To avoid confusion and streamline the narrative we removed the F-actin data altogether.

Comment 21

A clear description of cell types used in each experiment is missing in several figure legends and the main text, impairing interpretation of genotype-dependent effects.

Response 21

We thank the reviewer for this feedback and accordingly updated the main text and figure legends to clarify cell types and genotypes.

Comment 22

- Critical experimental details are missing:
- Concentration of Aβ used.
- QC of Aβ aggregates (e.g., EM or Thioflavin T).
- Normalization approaches (e.g., in viability assays).

Response 22

We thank the reviewer for the suggestion to include Thioflavin T validation of our Aβ aggregates, and this can now be found in **Supplemental Figure 5c**. The critical experimental details are all present in the methods, with normalization approaches grouped with the corresponding assay. The concentration of Aβ can be found under "*Treatments*"; "Cells were treated with 3.3 µg/mL of amyloid beta"

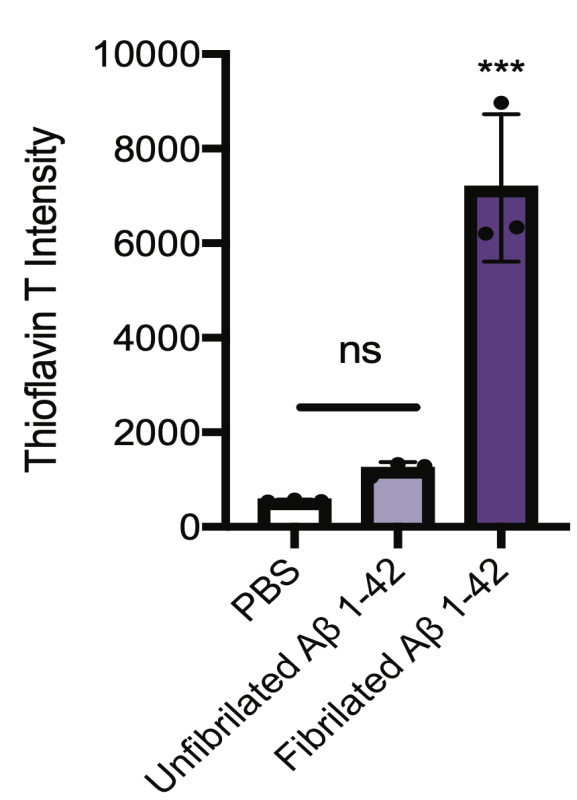

Supplemental Figure 5c: Validation of amyloid beta aggregates as measured by Thioflavin T incorporation

Comment 23
Indication of biological replicate number and presentation of individual data points across figures.
For example: The sample size in Figures 6a and 6c appears limited (possibly only n=2). I would ask the authors to show individual data points across all graphs for transparency.

Response 23
We thank the reviewer for this comment. All of the biological replicate information is included in the methods, and graphs missing individual data points were added.

Comment 24
The manuscript presents multiple disconnected hypotheses (lysosomal function, ferroptosis, ISGylation, etc.) without clear experimental resolution.
o Stronger integration and prioritization of mechanistic themes are necessary to clarify ARID5A's core function.
o Each line of speculation should be explicitly linked to testable hypotheses and validated findings, or the speculation should be tempered accordingly.

Response 24
We thank the reviewer for this feedback and prioritized cohesion in our revision. To improve cohesion, we connected these hypotheses with additional data, validation, and discussion. These are highly interconnected pathways that we admittedly did not convey how they relate in our prior version. This led us to create **Figure 7**, in order to underscore both the interconnected nature of these pathways and the role of ARID5A. We have tempered speculation where appropriate, and added additional validations and findings to **Figure 3**, **Figure 4, Figure 5**, and **Figure 6.**

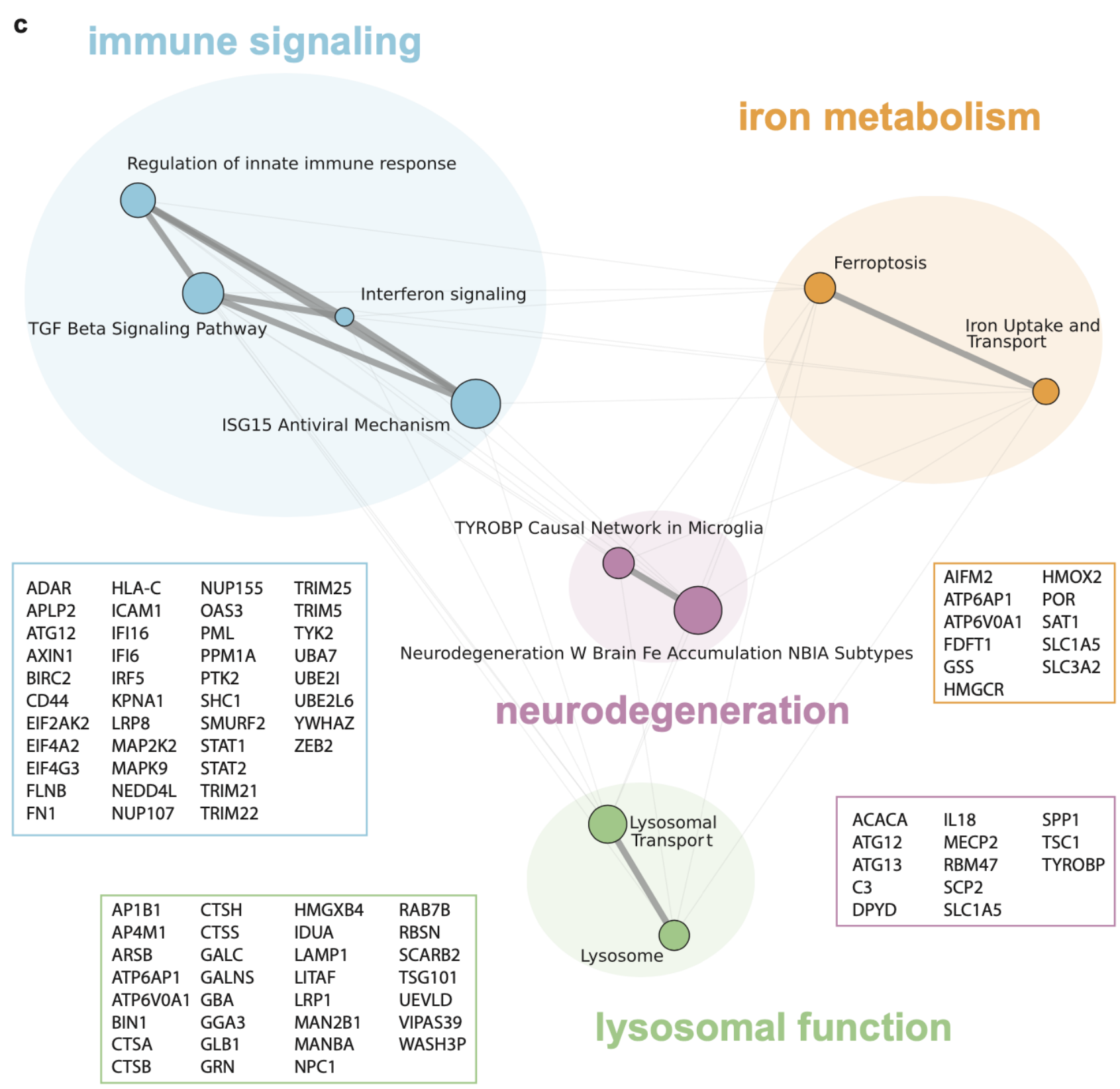

Figure 7c: ARID5A regulates an interconnected network of lysosomal, immune, and iron pathways that contribute to neurodegeneration. Boxed genes are regulated by ARID5A at the RNA level.

Comment 25
The abstract overstates the novelty of CNS-specific RBP functon("remain uncharacterized") and should be revised to acknowledge the growing body of literature.

Response 25

We thank the reviewer for this comment. We do not wish to disregard the growing body of work and as such changed the abstract to say "poorly understood" since it is still a relatively new and growing field. Relevant studies contributing to this field are cited in the Introduction.

Comment 26

Supplementary Figure 1c: The figure legend lacks clarity, particularly regarding ARID5A's apparent significance in only one replicate of the Olah_activation dataset.

Response 26

We thank the reviewer for this feedback and accordingly provided clarifying details that this figure is binary (present vs absent) in published datasets that determined their microglia population gene lists from multiple replicates. The figure legend now reads "Presence of Macrophage/Microglial RBPs in microglial transcriptomic signatures derived from published datasets (Olah et al. 2020, Silvlin et al. 2022, Millet et al. 2024)". For the Olah publication, Activation_1 and Activation_2 are two different populations.

Comment 27

Supplementary Figure 4F: Show replicate data and quantification to support the reported findings.

Response 27

We thank the reviewer for this suggestion and have included new higher resolution immunofluorescence images with replicates and quantification (**Figure 2a, Supplemental Figure 4g-i**).

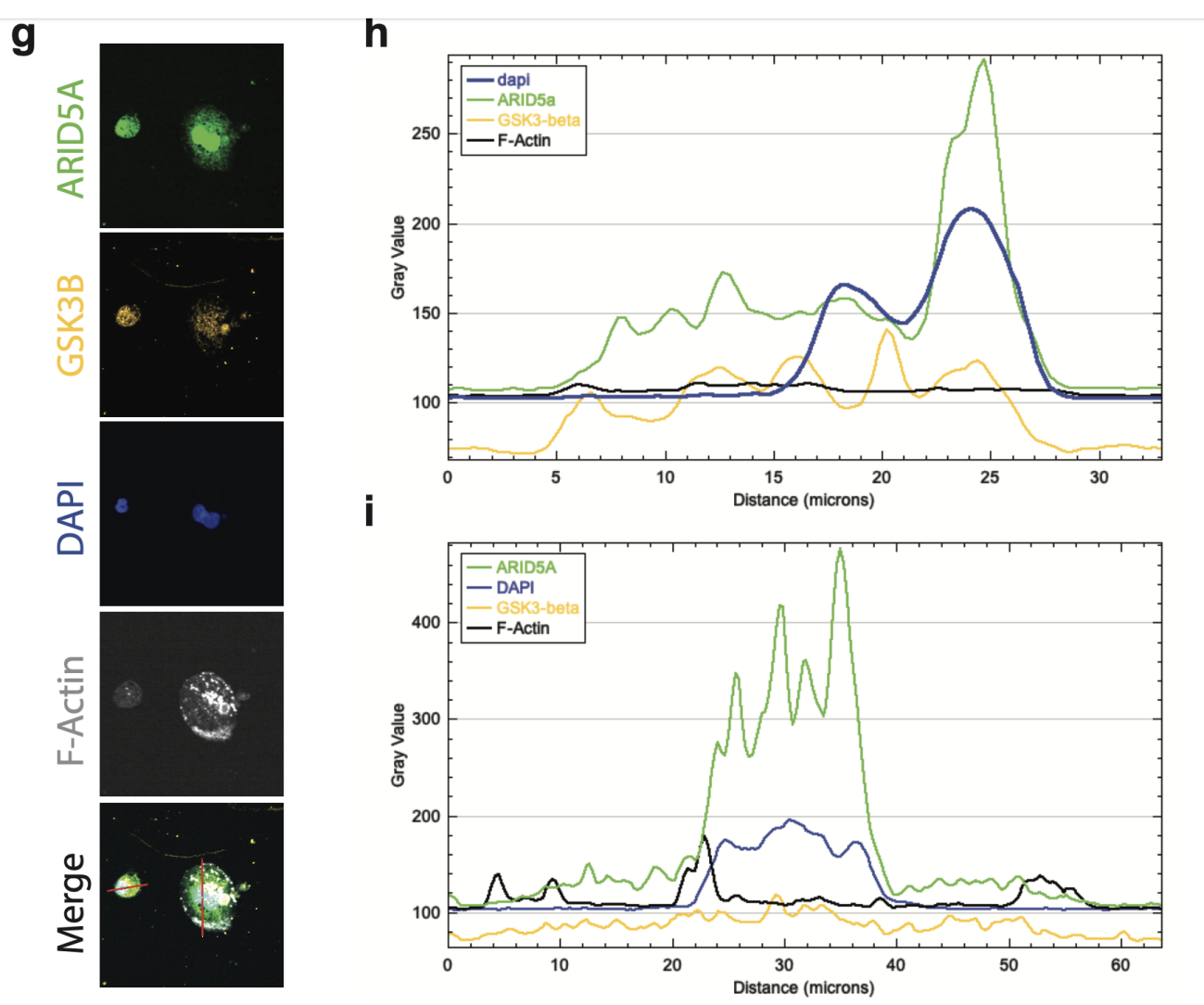

Supplemental Figure 4g-i: ARID5A is expressed primarily in the nucleus, but also in the cytoplasm relative to DAPI

Comment 28

Figure 6f–i: The method switches from flow cytometry (Fig. 6c) to Incucyte without explanation.

Response 28

We thank the reviewer for this comment and to address this we repeated the experiments solely with flow cytometry.

Comment 29

NT groups consistently have fewer cells, and since data are shown as RCU/μm2 (incorrectly labeled as "μM"), it likely is not normalized to cell count.

Response 29
We thank the reviewer for bringing this to our attention and apologize for the oversight. We have corrected this and ensured all Incucyte data is properly labeled. The data was normalized to cell count in line with standard Incucyte analysis protocols, as described in the methods, under "*Incucyte live cell imaging*";
"Integrated intensity was calculated by normalizing fluorescence to phase cell count, and standard deviation was calculated by image per condition."

Comment 30
Figure 6I: Cytokine levels should be reported in pg/mL for standardization.

Response 30
We thank the reviewer for this suggestion. Due to differences in viability, we find these cells to have markedly different protein levels. We normalized to total protein to ensure this data was not due to differences in cell count and/or protein.

Comment 31
Terminology: Clarify use of "sg2" and "sg3" for shRNA knockdowns to avoid confusion with CRISPR nomenclature.

Response 31
Sg2 and sg3 are CRISPRi guides, not shRNA knockdowns, as shown in the manuscript:

i) Results: "ARID5A-dependent splicing events were profiled in microglia differentiated from hiPSCs through inducible transcription factor expression (iTF) and CRISPRi-mediated depletion"
ii) **Supplemental Figure 5a** legend: "a. Schematic of iTF Microglia differentiation from iPSCs containing a doxycycline-inducible transcription factors system and CRISPRi machinery. Incucyte images (20x) of nontargeting control (NT), ARID5A-targeting CRISPRi guide 2 (sg2) and guide 3 (sg3) on Day 8"
iii) Methods:

a) Section "*Cloning of CRISPRi guides*"; CRISPRi sgRNA lentivirus plasmids were generated as previously described[50,90,91]. In brief, sequences for CRISPRi guides were obtained from the CRISPRi v2 H1 library[90]. Guides were annealed and ligated into the pMK1334 backbone (Addgene, #127965)"
b) Section "*Generation of Induced Microglia (iTFs)*"; iPSCs harboring inducible microglial transcription factors and constitutive CRISPRi machinery[50] were transduced with CRISPRi sgRNA lentiviruses using TransIT (Mirus Bio, #MIR 6600).

Response to Reviewer #2 (Remarks to the Author):

Comment 1

This manuscript (NCOMMS-25-29083-T) demonstrated the machinery of Arid5a in microglia activation as DNA/RNA binding protein. The authors clarified that Arid5a has both activities to bind RNA and DNA directly and indirectly in microglia, although a distinct repertoire, and they suggested the potential role in splicing. In the last part, the authors identified the function of Arid5a by knockdown in microglia, and they demonstrated that knockdown of Arid5a has several functions to have regulation of splicing of neurodegenerative proteins and the alteration of Aβ-induced GCase activity and lysosome response in microglia. In addition, knockdown of Arid5a in microglia augmented the immune responses. Next, knockdown of Arid5a decreased ferroptosis by inhibition of lipid peroxidation in microglia, indicating protective function. Last, under Aβ stress, depletion of Arid5a also exerts the protective function in TREM-T66M mutant microglia which impaired in phagocytosis and neuroinflammation. However, there are many concerns to be link the machinery of Arid5a function as RNA/DNA binding protein and the phenotypes of Arid5a knockdown microglia.

Response 1

We thank the reviewer for this comment. To address concerns linking ARID5A RNA binding activity with functional phenotypes in microglia, we performed additional experiments. This included the added ferroptosis inhibitor data in which the effects of ARID5A overexpression on erastin-induced cell death were mitigated by iron chelator DFO, but not other ferroptosis inhibitors that go through oxidative pathways such as liproxstatin and ferrostatin (**Figure 5, Supplemental Figure 8**).This provided further mechanistic insight, and to bridge this with RNA binding we examined ARID5A RNA binding sites for iron-responsive elements (IREs). IREs are found within RNA sequences and sensitive to changes in iron. In agreement with our functional data, ARID5A RNA binding sites contained many IREs (**Supplemental Figure 8**). Notably, these IREs were within transcripts known to regulate ISGylation, another key phenotype we described for ARID5A (**Figure 4**, **Supplemental Figure 7**). Collectively these findings not only point to iron metabolism as a key component of ARID5A regulation of ferroptosis, but also tie together ARID5A regulation of ISGylation and downstream immune processes. In addition to supplementing our manuscript with additional validations and mechanistic experiments, we refined the text to further highlight the interconnected nature of ferroptosis, lysosomal function, and immune modulation and how this can manifest in a model of neuroinflammation in **Figure 7**.

c

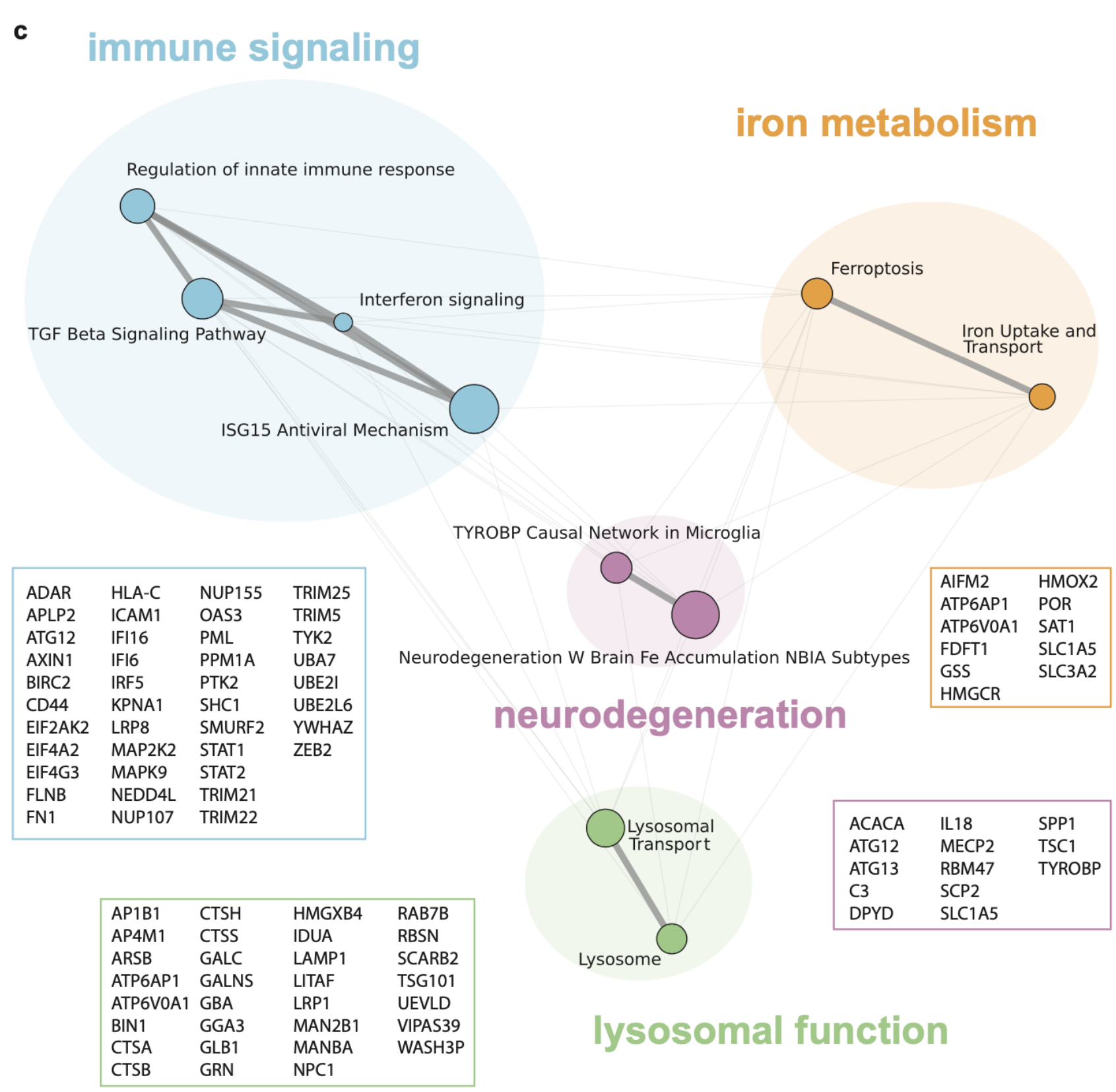

Figure 7c: ARID5A regulates an interconnected network of lysosomal, immune, and iron pathways that contribute to neurodegeneration. Boxed genes are regulated by ARID5A at the RNA level.

Response to Reviewer #3 (Remarks to the Author):

Comment 1

Identification of ARID5A: The authors perform extensive analysis to pinpoint the importance and specificity of ARID5A; however, they do not provide details about their analyses. A supplementary table with more information on Figures 1A and B is necessary to understand the importance of ARID5A among the other RBPs.

Response 1

We thank the reviewer for this feedback and sincerely apologize for not providing sufficient details. To address this concern we have provided Supplemental Table 1 and more details within the Methods:
"Candidate RBPs were defined with a HydRA score of greater than or equal to 0.83. Cell type annotation was assigned based on the Human Protein Atlas, and further binned into the categories "Neurons", "Astrocytes", "Oligodendrocytes", "Macrophages and Microglia", "Endothelial Cells", "Other Immune Cells", and "Ubiquitous". Percentage of RBPs was determined by dividing the number of HydRA predicted RBPs over the total number of cell-type defining genes (Supplemental Table 1)."

Comment 2

Specificity of ARID5A targets in microglia: ARID5A is a known regulator of gene expression in peripheral immune cells. Moreover, ARID5A is observed in the cortical organoid (Fig. 1D). The authors claim that ARID5A is linked to specific microglia targets involved in disease. A comparison with a known ARID5A binding partner observed in peripheral immune cells and neuronal cells is essential to support this claim.

Response 2

We thank the reviewer for this comment and to address the specificity of microglia genes we included enrichment in **Figure 3c** depicting that directly bound and differentially spliced transcripts were enriched for "Top Microglial Expressed Genes in Humans and Mice". For disease-relevance, we examined established disease-datasets for ARID5A-dependent RNA regulation. These are shown in **Supplemental Figure 10**. Some of these include immune-specific transcripts such as *TREM2* whereas others are widely expressed in CNS cell types such as *BIN1*. For Western blotting, ARID5A is enriched in microglia, but can be detected at lower levels in astrocytes. Based on the Human Protein Atlas, ARID5A is expressed six-fold higher in CNS macrophages/microglia than astrocytes (43.9 vs 7.3 tpm). As such, the lower yet detected expression of ARID5A in the organoids can likely be attributed to the presence of astrocytes.

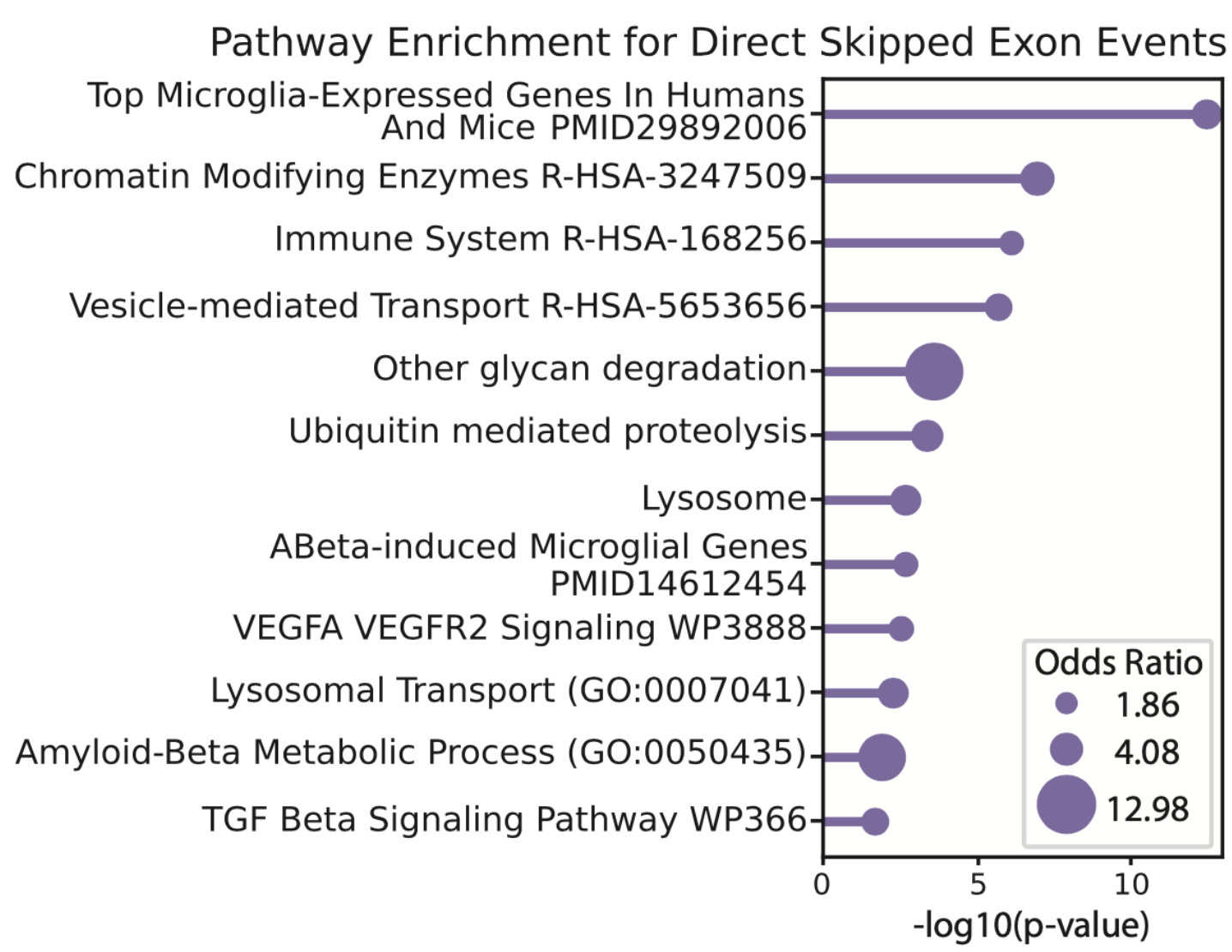

Figure 3c: ARID5A bound and differential skipped exon events were enriched for microglial genes

Comment 3

Functional assays: While the reviewer appreciates the challenge of generating functional validation, some assays and statistical measures are below standards in the field. All experiments should be done in triplicate at least; individual data points should be shown, and similar quantification should be done across assays. For example, Fig. 5E and many panels of Fig. 6 only have 2 data points, Fig. 6G doesn't have an individual datapoint, and phagocytosis readout varies between Fig. 6C and G. Figure 6A should include a loading control.

Response 3

We thank the reviewer for this feedback and have accordingly repeated the experiments and show individual datapoints, especially within Figures 5-6.We repeated experiments to consistently use flow cytometry as a readout as opposed to switching between the two methods. We repeated the Western blot and added a loading control and this data is now in **Supplemental Figure 9a-b**.

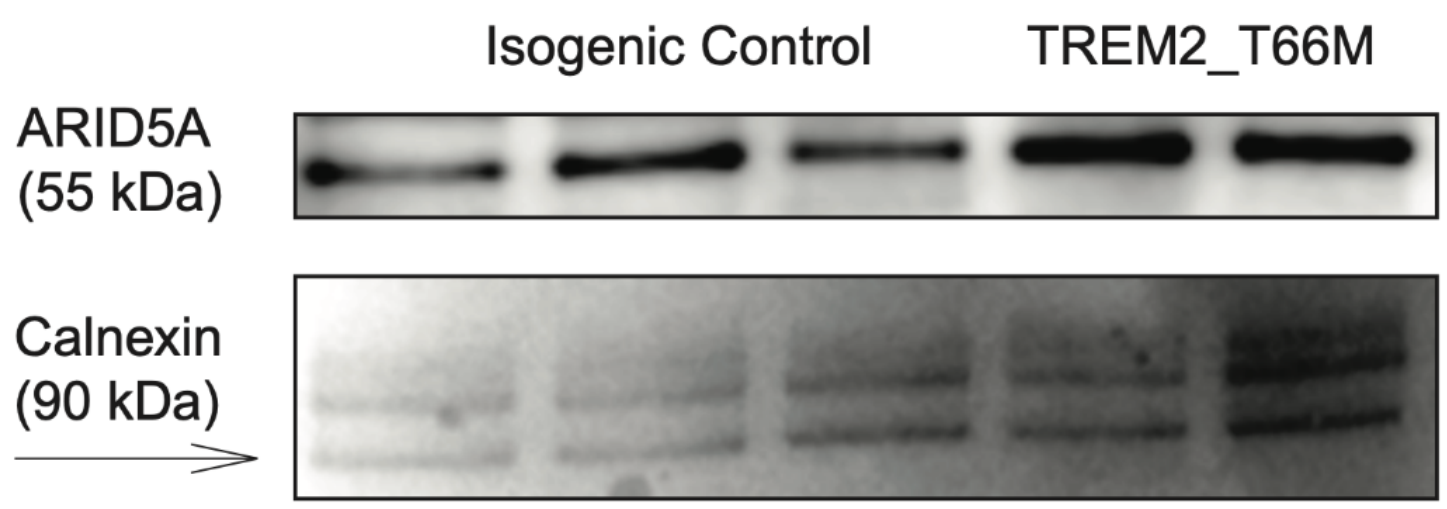

Supplemental Figure 9a: Western blot of ARID5A and loading control (Calnexin) in T66M vs isogenic control microglia

Comment 4

The set of data presented includes a role of ARID5A in binding Alzheimer disease proteins (Fig. 2C), a role in the regulation of GBA (Fig. 3D), assays with amyloid, ferroptosis (Fig. 5), and TGFb (Fig. 1K). The picture drawn by the authors is not clear at the moment on the role of ARID5A in neurodegeneration because all these phenotypes could lead to neurodegeneration through diverse mechanisms. These data need to be tied together with a direct causal link. Some possibilities could be finding the initial step mediated by ARID5A and leading to microglia dysfunctions, showing that these mechanisms happen in the same cells, or validating the role of ARID5A in a mouse model in early stages of disease.

Response 4

The reviewer is correct to point out that all of these phenotypes can lead to neurodegeneration and we thank them for this feedback. As a result, we prioritized cohesion and better integration of these phenotypes, as described in Overall Response. We added **Figure 7** to the manuscript to highlight the interconnected nature of these pathways and relevance to neurodegeneration. We provided additional experimental data, validations, and analyses to better tie together lysosomal, ferroptosis, and immune phenotypes and their importance in a model of neuroinflammation/neurodegeneration.

We show ARID5A overexpression in ferroptosis can be mitigated with the iron chelator DFO, but not with other ferroptosis inhibitors that act through oxidative pathways (**Figure 5j-k**, **Supplemental Figure 8b-c**). This points to iron accumulation as the key mechanism for ARID5A regulation of ferroptosis and that ARID5A sensitizes cells to ferroptosis. Further, we found that ARID5A eCLIP binding sites contain known and predicted iron response elements which not only further suggest a role in iron metabolism, but were also detected in transcripts involved in ISGylation (**Supplemental Figure 8d-h**). We further provide evidence that ARID5A modulates ISGylation in both the knockdown microglia as well as an overexpression HeLa model (**Figure 4i** ,**Supplemental Figure 7f**. Exploration of known ISGylation targets that were differentially translated upon ARID5A knockdown were also enriched for ferroptosis and lysosome activity, suggesting these pathways are tightly interconnected (**Supplemental Figure 8a**). In addition to our new data and analyses, we also included discussion and citation of studies that further demonstrate connections among these pathways that were in agreement with our results.

We agree with the reviewer on the importance of mouse models, and as such is an ongoing direction in the lab. Mouse microglia and human microglia differ substantially[15], and human ARID5A only shares about 50% similarity with mouse

ARID5A. As such, we believe these should be studied thoroughly but separately. Our future work will build on the foundation we provided in this manuscript as well as capture novel insights on ARID5A function within the *in vivo* context.

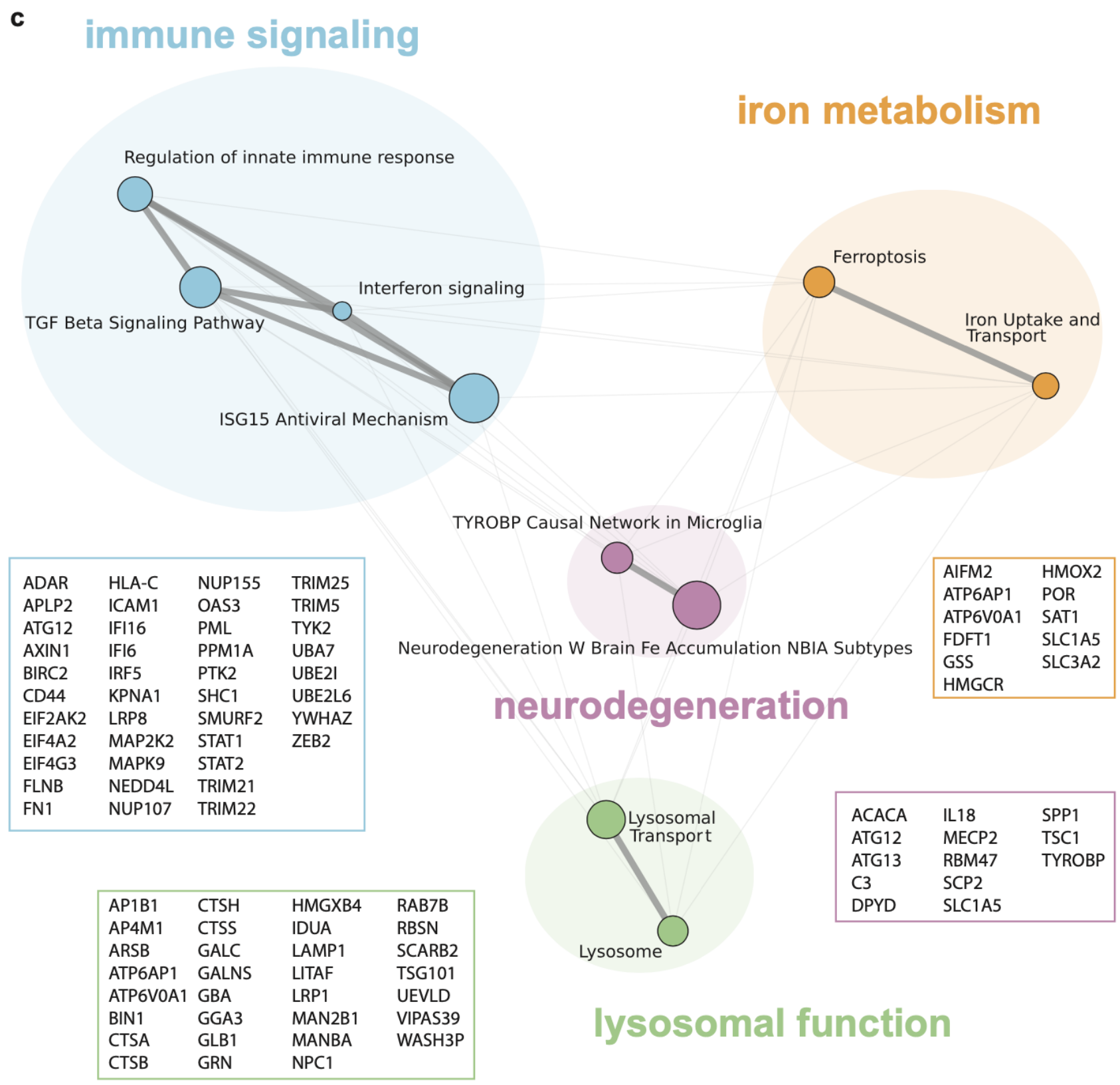

Figure 7c: ARID5A regulates an interconnected network of lysosomal, immune, and iron pathways that contribute to neurodegeneration. Boxed genes are regulated by ARID5A at the RNA level.

Comment 5

Also, the 'neuroprotective potential" of ARID5A and "therapeutic potential" mentioned in the abstract and introduction should be rephrased; these are overstatements.

Response 5

We thank the reviewer for this comment and sincerely apologize, as we do not wish to overstate our results. To address this concern, the terms "neuroprotection/neuroprotective potential" and "therapeutic potential" have been removed from the manuscript altogether.

References

1. Markmiller, S., Sathe, S., Server, K.L., Nguyen, T.B., Fulzele, A., Cody, N., Javaherian, A., Broski, S., Finkbeiner, S., Bennett, E.J., et al. (2021). Persistent mRNA localization defects and cell death in ALS neurons caused by transient cellular stress. Cell Rep *36*, 109685. https://doi.org/10.1016/j.celrep.2021.109685.

2. Yang, L., Gal, J., Chen, J., and Zhu, H. (2014). Self-assembled FUS binds active chromatin and regulates gene transcription. Proceedings of the National Academy of Sciences *111*, 17809–17814. https://doi.org/10.1073/pnas.1414004111.

3. Gosztyla, M.L., Zhan, L., Olson, S., Wei, X., Naritomi, J., Nguyen, G., Street, L., Goda, G.A., Cavazos, F.F., Schmok, J.C., et al. (2024). Integrated multi-omics analysis of zinc-finger proteins uncovers roles in RNA regulation. Molecular Cell *84*, 3826-3842.e8. https://doi.org/10.1016/j.molcel.2024.08.010.

4. Higa, M., Oka, M., Fujihara, Y., Masuda, K., Yoneda, Y., and Kishimoto, T. (2018). Regulation of inflammatory responses by dynamic subcellular localization of RNA-binding protein Arid5a. Proceedings of the National Academy of Sciences *115*, E1214–E1220. https://doi.org/10.1073/pnas.1719921115.

5. Horlbeck, M.A., Gilbert, L.A., Villalta, J.E., Adamson, B., Pak, R.A., Chen, Y., Fields, A.P., Park, C.Y., Corn, J.E., Kampmann, M., et al. (2016). Compact and highly active next-generation libraries for CRISPR-mediated gene repression and activation. Elife *5*, e19760. https://doi.org/10.7554/eLife.19760.

6. Tian, R., Gachechiladze, M.A., Ludwig, C.H., Laurie, M.T., Hong, J.Y., Nathaniel, D., Prabhu, A.V., Fernandopulle, M.S., Patel, R., Abshari, M., et al. (2019). CRISPR Interference-Based Platform for Multimodal Genetic Screens in Human iPSC-Derived Neurons. Neuron *104*, 239-255.e12. https://doi.org/10.1016/j.neuron.2019.07.014.

7. Dräger, N.M., Sattler, S.M., Huang, C.T.-L., Teter, O.M., Leng, K., Hashemi, S.H., Hong, J., Aviles, G., Clelland, C.D., Zhan, L., et al. (2022). A CRISPRi/a platform in human iPSC-derived microglia uncovers regulators of disease states. Nat Neurosci *25*, 1149–1162. https://doi.org/10.1038/s41593-022-01131-4.

8. Dolan, M.-J., Therrien, M., Jereb, S., Kamath, T., Gazestani, V., Atkeson, T., Marsh, S.E., Goeva, A., Lojek, N.M., Murphy, S., et al. (2023). Exposure of iPSC-derived human microglia to brain substrates enables the generation and manipulation of diverse transcriptional states in vitro. Nat Immunol *24*, 1382–1390. https://doi.org/10.1038/s41590-023-01558-2.

9. Smolders, S., Kessels, S., Smolders, S.M.-T., Poulhes, F., Zelphati, O., Sapet, C., and Brône, B. (2018). Magnetofection is superior to other chemical transfection methods in a microglial cell line. J Neurosci Methods *293*, 169–173. https://doi.org/10.1016/j.jneumeth.2017.09.017.

10. Masuda, K., Ripley, B., Nishimura, R., Mino, T., Takeuchi, O., Shioi, G., Kiyonari, H., and Kishimoto, T. (2013). Arid5a controls IL-6 mRNA stability, which contributes to elevation of IL-6 level in vivo. Proc Natl Acad Sci U S A *110*, 9409–9414. https://doi.org/10.1073/pnas.1307419110.

11. Nyati, K.K., and Kishimoto, T. (2021). Recent Advances in the Role of Arid5a in Immune Diseases and Cancer. Front Immunol *12*, 827611. https://doi.org/10.3389/fimmu.2021.827611.

12. von Ehr, J., Oberstrass, L., Yazgan, E., Schnaubelt, L.I., Blümel, N., McNicoll, F., Weigand, J.E., Zarnack, K., Müller-McNicoll, M., Korn, S.M., et al. (2024). Arid5a uses disordered extensions of its core ARID domain for distinct DNA- and RNA-recognition and gene regulation. J Biol Chem *300*, 107457. https://doi.org/10.1016/j.jbc.2024.107457.

13. Taylor, T.C., Li, Y., Li, D.-D., Majumder, S., McGeachy, M.J., Biswas, P.S., Gingras, S., and Gaffen, S.L. (2022). Arid5a Mediates an IL-17-Dependent Pathway That Drives Autoimmunity but Not Antifungal Host Defense. J Immunol *209*, 1138–1145. https://doi.org/10.4049/jimmunol.2200132.

14. Baxter, E.W., Graham, A.E., Re, N.A., Carr, I.M., Robinson, J.I., Mackie, S.L., and Morgan, A.W. (2020). Standardized protocols for differentiation of THP-1 cells to macrophages with distinct M(IFNγ+LPS), M(IL-4) and M(IL-10) phenotypes. J Immunol Methods *478*, 112721. https://doi.org/10.1016/j.jim.2019.112721.

15. Gosselin, D., Skola, D., Coufal, N.G., Holtman, I.R., Schlachetzki, J.C.M., Sajti, E., Jaeger, B.N., O'Connor, C., Fitzpatrick, C., Pasillas, M.P., et al. (2017). An environment-dependent transcriptional network specifies human microglia identity. Science *356*, eaal3222. https://doi.org/10.1126/science.aal3222.

Response to Reviewer 1

ARID5A RNA-binding Coordinates Microglial Defense and Ferroptosis

Response to Reviewer #1 (Remarks to the Author):

The authors have addressed many of the major conceptual and technical issues raised in the previous review. In particular, they have strengthened the validation of their sequencing results and clarified the iron-dependent mechanism underlying ARID5A-mediated ferroptosis. The revised manuscript provides a more coherent narrative that links immune, lysosomal and iron metabolism pathways.

We thank the reviewer for recognizing the improvements made to the conceptual and technical issues to produce a more coherent narrative and for their initial feedback that greatly aided our revision

Comment 1

However, some limitations remain. For example, the separation of the RNA- and DNA-binding functions of ARID5A remains largely descriptive.

Response 1

We thank the Reviewer for this comment. We agree that separation of RNA and DNA binding functions is an exciting direction, but opine that a deeper dive into this is beyond the scope of the current manuscript. We have already generated novel datasets of ARID5A-RNA and ARID5A-DNA interactions using state-of-the-art genome wide measurements, and sharing these foundational datasets propels the field as a whole forward. As an RNA binding protein lab, we leveraged our expertise in splicing and translation to build upon ARID5A-RNA interactions and paired this with functional assays such as live cell imaging and flow cytometry to connect ARID5A-RNA interactions with readouts focused on ferroptosis, lysosome function, and immune signaling. The undertaking to separate out DNA vs RNA binding remains a challenge for the field at large, as separation-of-function mutations are not easy to decipher for many proteins. Also, it might be impossible. As demonstrated by von Ehr et al 2024 and ourselves in the revision, the ARID domain binds both DNA and RNA. As we demonstrated, domain deletion still results in RNA binding, as the IDR binds RNA with high affinity. The mechanistic undertaking to fully disentangle DNA vs RNA binding is ambitious and has the potential to amount to an entirely separate manuscript exclusively dedicated to the biochemistry of these interactions.

Comment 2

Furthermore, there are still several presentation and data quality issues. For example, all Western blots should include clearly labelled molecular weight markers to enable the correct interpretation of band identity and specificity (see Fig. 2E, S1E, S2H).

Response 2

We thank the Reviewer for this comment and apologize for the oversight. As shown below, Figures 2E, S1E, S2H were updated to include molecular weight labels. We also caught this error in Figure S4E, which we also corrected.

2E

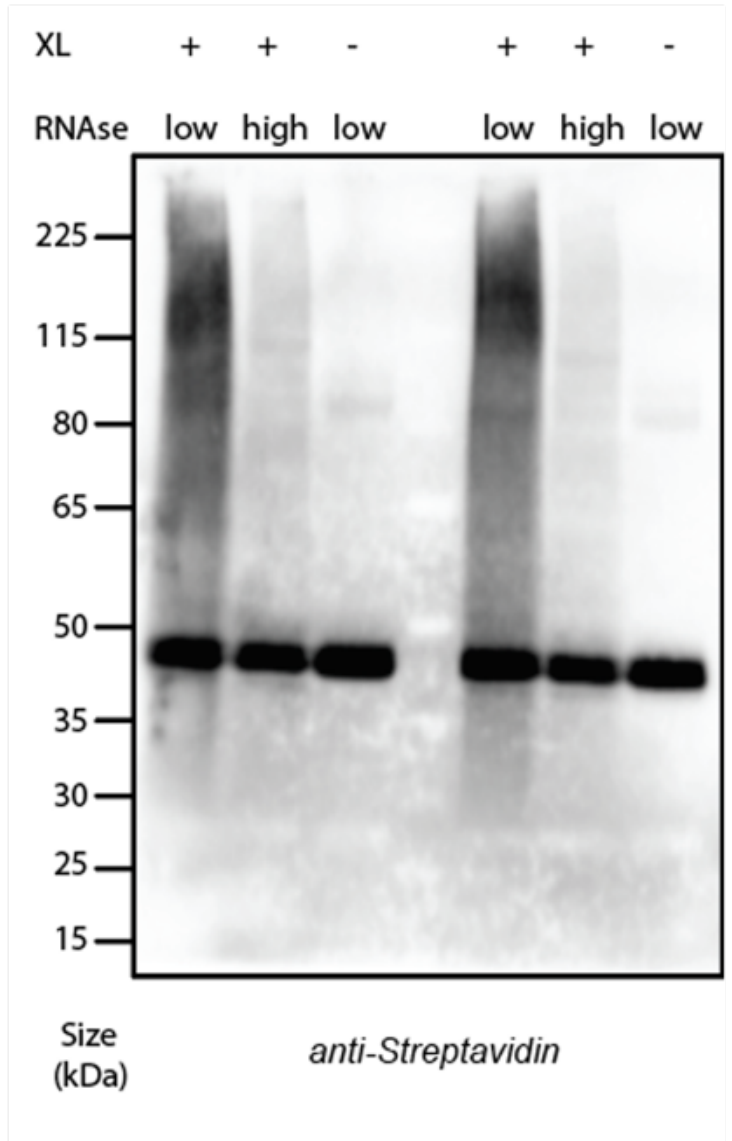

S1E

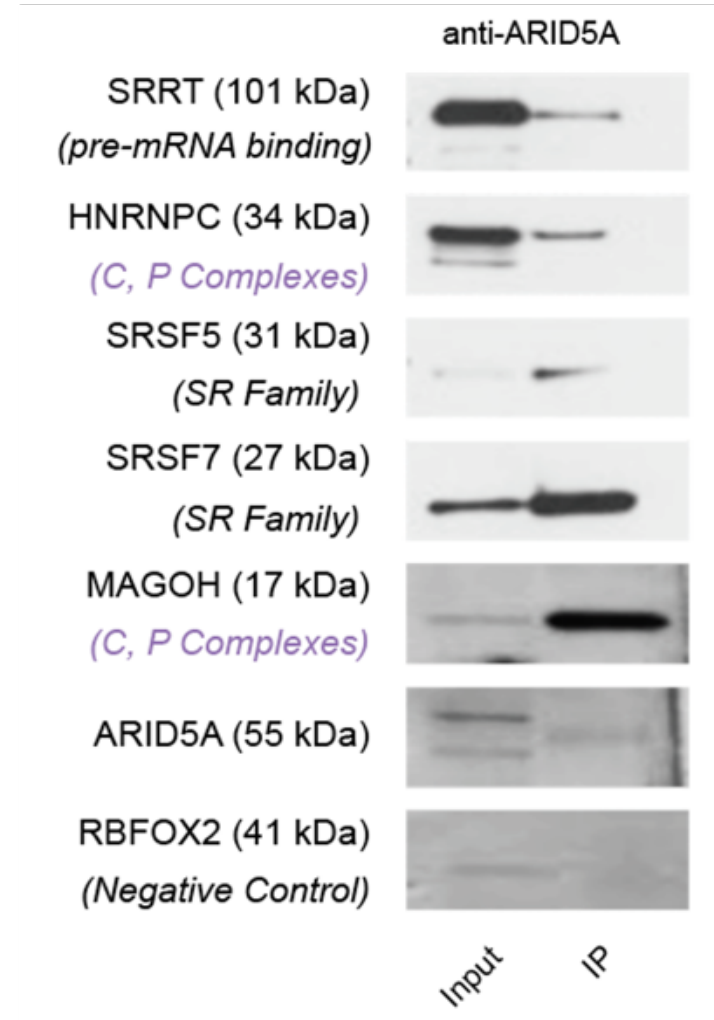

S4E

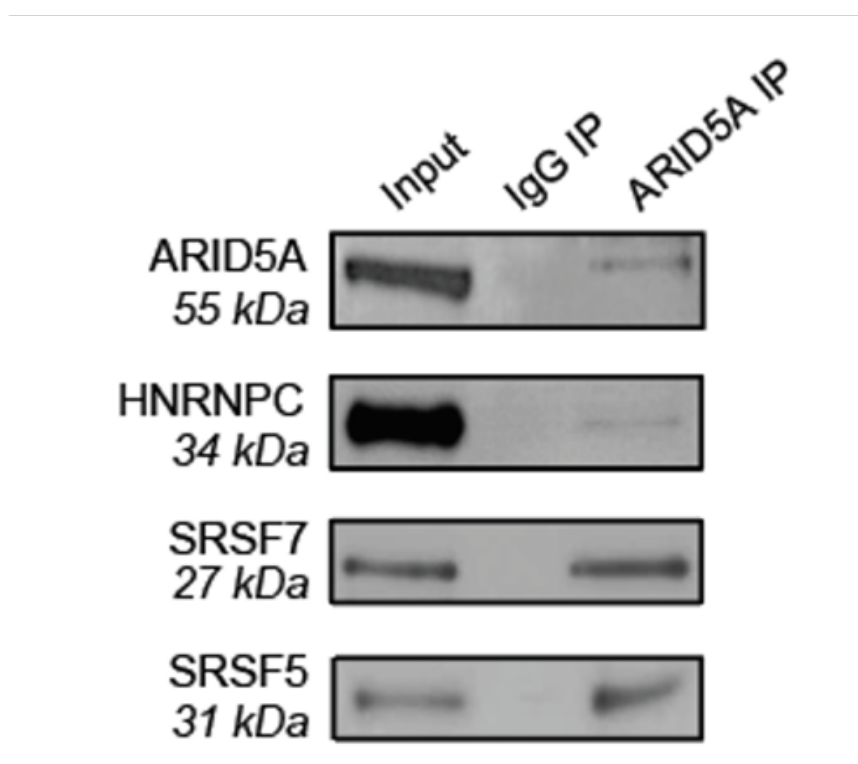

S2H

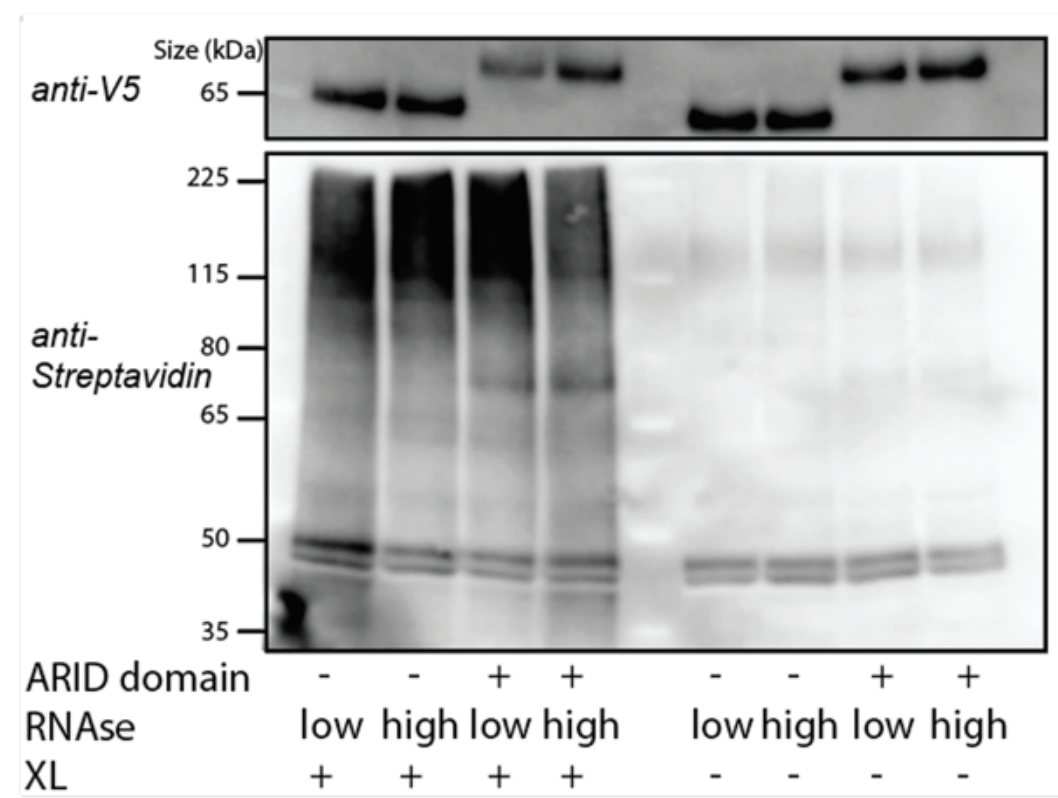

Figure 1 Blots Updated to Include Molecular Weight Labels

Comment 3
The quality of the confocal microscopy images (Fig. 2A, 5H, 6M, S4G, S5E, S5H), including the newly added repeats, remains below the standard typically seen in comparable publications for microglia and macrophages. Limited resolution and contrast make subcellular localisation difficult to assess with confidence (Fig. 2A).

Response 3
We thank the Reviewer for this comment and apologize for the reduced resolution. Due to size limitations for submission, images were compressed. We have generated a Google Drive link for the reviewer to view the uncompressed images (Fig. 2A, 5H, 6M,S4G, S5E, S5H) at the highest resolution possible. They can be viewed at this link: https://drive.google.com/drive/u/0/folders/1Q54IaLHCXnnITOhqJ_zxBnpwj9xe7_AS

It is important to note that the Incucyte is not a confocal microscope and only capable of imaging at 20x in our facility. Image quality of Incucyte images is consistent with other publications and importantly were not used for quantification in our resubmission. Quantification was measured by orthogonal flow cytometry assays were appropriate.

Comment 4
Furthermore, the cells appear unhealthy in multiple experiments, even under untreated or baseline conditions, raising concerns about culture quality and the potential impact of cellular stress on the reported phenotypes.

Response 4
We agree with the Reviewer that cell culture quality is important in discerning results. We respectively disagree, however, that cell culture conditions can or needs to be reasonably improved because our morphologies are consistent with other publications and our culture practices were optimized to the highest possible standard. We have highlighted the publications as well as show a side by side of our morphologies with others' images, and provide in depth details of our cell culture practices.
Benchmarking against other publications.

- For iTF lines, the cell morphology (Figure 5c) is consistent with the morphology reported in the original publication (Figure 1b of Dräger et al 2022).
- Regarding the iMG cells, the morphology of the isogenic line is consistent with what is regularly observed in differentiation (Figure 1, McQuade et al 2018). These cells were not replated for imaging, yet exhibit a healthy ramified morphology and bright cell bodies. This protocol was commercialized by StemCell Technologies, and the image from their website (Figure 2c in documentation) shows a morphology for their healthy control lines that is a blend of what we observed in our isogenic controls and our severely inflamed mutant line, the T66M homozygotes.
- Regarding the T66M cells, this mutation is very severe as evident by our study and others' publications. Our morphology was consistent with those reported by Garcia-Reitboeck et al 2018, and strongly indicates this is a phenotype of this mutation given that these cells were differentiated in two separate labs with two separate protocols and differentiated from different iPSC lines (Figure 2, Garcia-Reitboeck et al 2018). These cells were consistently differentiated alongside a healthy control to ensure proper differentiation multiple times over the course of two years. As such, this morphology is a core phenotype and thus no change in culture conditions is likely to change it.

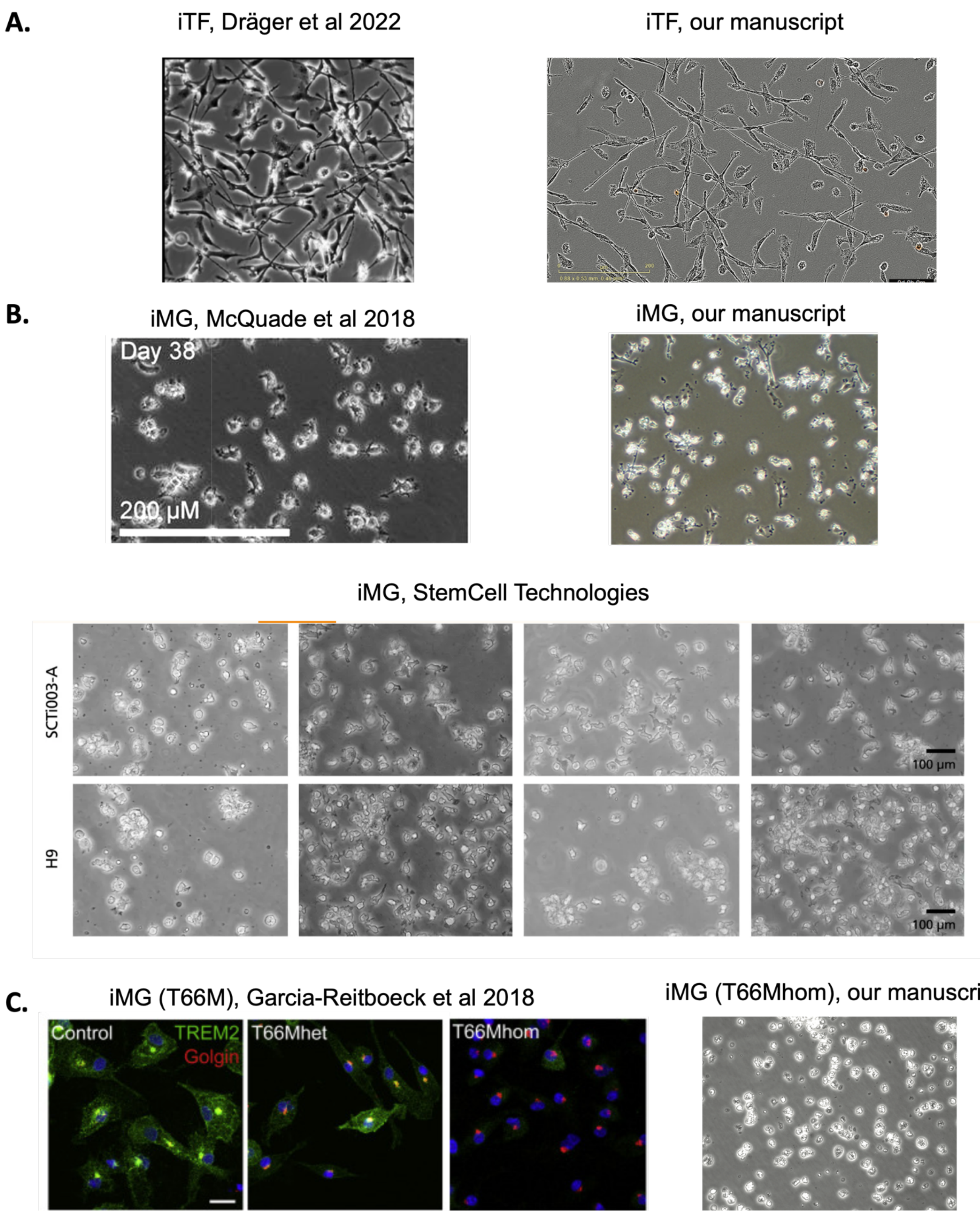

Figure 2: Cell Quality for this Manuscript is Consistent and Robust with Field Standards*:*
a. Comparison of iTF microglia from original publication Dräger et al 2022 (left) with iTF microglia generated for our manuscript (right **b.** Comparison of our iMG (iPSC derived microglia) generated for this manuscript (right) alongside iMGs from the original publication McQuade et al 2018 (left) and iMGs from StemCell Technologies' documentation, who commercialized the McQuade protocol into a kit (bottom). **c.** Healthy, T66Mhet, and T66Mhom microglia generated in Garcia-Reitboeck et al 2018 from a different iPSC-derived microglia protocol (left) show the same rounded morphology as observed in our T66Mhom microglia generated for our manuscript (right).

Cell culture practices and optimization

- Cells were cultured in a sterile room dedicated to iPSC differentiations and regularly tested for mycoplasma contamination. The incubator was regularly calibrated for CO2 and the water bath changed weekly.
- iPSCs were cultured without antibiotics. Alongside the mycoplasma testing, this ensured that starting iPSCs were pristine.
- Spontaneous differentiation was rare due to routine passaging with Relesr and fresh Cultrex. In our hands, differentiation of hematopoietic progenitors failed to produce viable cells if iPSCs were unhealthy, so as a result all of our microglia were differentiated from healthy iPSCs. TREM2 is specific to myeloid cells, so we do not observe the effects of T66M mutation at the iPSC stage.
- Basal media was made fresh every two weeks with growth factors added fresh each media change to prevent degradation.
- Growth factors were aliquoted for individual use to avoid freeze thaw cycles.
- Media changes were performed in the same time window every day (10 am-12 pm PST) for two years. Aliquots of media were first warmed to 37°C, and growth factor aliquots were subsequently added and filtered.
- Cell plating was optimized both in regards to density and plating substrate.
    - Density of initial hematopoietic colonies and microglia for maturation was optimized under the guidance of StemCell Technologies' representatives, who as stated above commercialized the protocol. Plating was optimized over the course of two years prior to the experiments of this study to minimize any cell death.
    - In collaboration with another lab, we observed that different plate coatings dramatically altered microglia morphology and markers. Cultrex resulted in the most consistent microglia differentiations and was used fresh. Cells were replated onto freshly prepared Cultrex three days prior to experiments to ensure adherence and time to settle. We found overnight to be the optimal coating time for microglia adherence to Cultrex.

Overall our cell morphologies are consistent with StemCell Technologies' and others' publications, and our cell culture practices are in agreement with best practices. All phenotype comparisons were made between cells differentiated in parallel with appropriate controls. While ARID5A depletion improved T66M phenotypes, additional modulations are required to fully restore these cells to health. Nevertheless, our findings provide novel insights into a mutation that is incredibly damaging to microglia cells. We are confident that these cells were differentiated in the best possible conditions with the appropriate controls.

References

1. von Ehr, J., Oberstrass, L., Yazgan, E., Schnaubelt, L.I., Blümel, N., McNicoll, F., Weigand, J.E., Zarnack, K., Müller-McNicoll, M., Korn, S.M., et al. (2024). Arid5a uses disordered extensions of its core ARID domain for distinct DNA- and RNA-recognition and gene regulation. J Biol Chem 300, 107457. https://doi.org/10.1016/j.jbc.2024.107457.

2. Dräger, N.M., Sattler, S.M., Huang, C.T.-L., Teter, O.M., Leng, K., Hashemi, S.H., Hong, J., Aviles, G., Clelland, C.D., Zhan, L., et al. (2022). A CRISPRi/a platform in human iPSC-derived microglia uncovers regulators of disease states. Nat Neurosci 25, 1149–1162. https://doi.org/10.1038/s41593-022-01131-4.

3. McQuade, A., Coburn, M., Tu, C.H., Hasselmann, J., Davtyan, H., and Blurton-Jones, M. (2018). Development and validation of a simplified method to generate human microglia from pluripotent stem cells. Mol Neurodegener 13, 67. https://doi.org/10.1186/s13024-018-0297-x.

4. Garcia-Reitboeck, P., Phillips, A., Piers, T.M., Villegas-Llerena, C., Butler, M., Mallach, A., Rodrigues, C., Arber, C.E., Heslegrave, A., Zetterberg, H., et al. (2018). Human Induced Pluripotent Stem Cell- Derived Microglia-Like Cells Harboring TREM2 Missense Mutations Show Specific Deficits in Phagocytosis. Cell Rep 24, 2300–2311. https://doi.org/10.1016/j.celrep.2018.07.094.
